# A new route for the efficient metalation of unfunctionalized aromatics†
†Electronic supplementary information (ESI) available. CCDC 1869336–1869340. For ESI and crystallographic data in CIF or other electronic format see DOI: 10.1039/c8sc04325f


**DOI:** 10.1039/c8sc04325f

**Published:** 2019-02-05

**Authors:** Andrew J. Peel, Noriyuki Tezuka, James M. D'Rozario, Masanobu Uchiyama, Andrew E. H. Wheatley

**Affiliations:** a Department of Chemistry , University of Cambridge , Lensfield Road , Cambridge , CB2 1EW , UK . Email: aehw2@cam.ac.uk ; Fax: +44 (0)1223 336362; b Cluster of Pioneering Research (CPR) , Advanced Elements Chemistry Laboratory , RIKEN , 2-1 Hirosawa, Wako-shi , Saitama 351-0198 , Japan; c Graduate School of Pharmaceutical Sciences , The University of Tokyo , 7-3-1 Hongo, Bunkyo-ku , Tokyo 113-0033 , Japan . Email: uchiyama@mol.f.u-tokyo.ac.jp

## Abstract

The synthesis and isolation of a novel bimetallic species formed by reacting two equivalents of TMPLi with CuCl in the presence of Et_2_O are reported. X-ray crystallography reveals the Et_2_O-free tetranuclear aggregate (TMPCu)_2_(TMPLi)_2_**1**, which formally results from the catenation of dimers of TMPLi and TMPCu. NMR spectroscopy confirms that, upon dissolution in hydrocarbon media, the crystals fail to form a conventional Gilman cuprate dimer. Instead they exhibit a spectrum which is consistent with that recently proposed for an *isomer* of dimeric Gilman cuprate. Moreover, while pre-isolated Gilman cuprate is inert to benzene solvent, this new isomer smoothly affects aromatic deprotonation to give mainly Ph(TMP)_3_Cu_2_Li_2_**3**, which is formally a heterodimer of Gilman cuprate TMPCu(μ-TMP)Li **2** and PhCu(μ-TMP)Li **4**. Attempts to synthesise **3** through explicit combination of pre-isolated **2** and **4** were successful; additionally, this permitted the preparation of Ph(TMP)_3_Cu_3_Li **5** and Ph(TMP)_3_CuLi_3_**7** when **4** was combined in 1 : 2 ratios with TMPCu or TMPLi, respectively. **5** was characterised as metallacyclic in the solid-state, its structural features resembling those in **3** but with reduced Li–π interactions. It also proved possible to perform Cu/Li exchange on **5** (using ^*t*^BuOCu) to give a novel mixed organo(amido)copper species Ph(TMP)_3_Cu_4_**6**. Remarkably, the unprecedented reactivity of **1** towards benzene is reproduced by heating a 1 : 1 mixture of TMPLi and TMPCu in the same solvent; this gives predominantly **3**. On the other hand, mixtures which are rich in either Cu or Li can lead to the selective *in situ* formation of **5** or **7**. Though crystallographic data on **7** could not be obtained, DFT calculations accurately corroborated the observed structures of **3** and **5** and could be used to support **7** having the same structure type, albeit with enhanced Li–π interactions. This was consistent with NMR spectroscopic data. However, in contrast to **3** and **5**, for which 2D NMR spectroscopy indicated only conformational changes, **7** was additionally found to exhibit fluxionality in a manner consistent with a dissociative process.

## Abstract 2

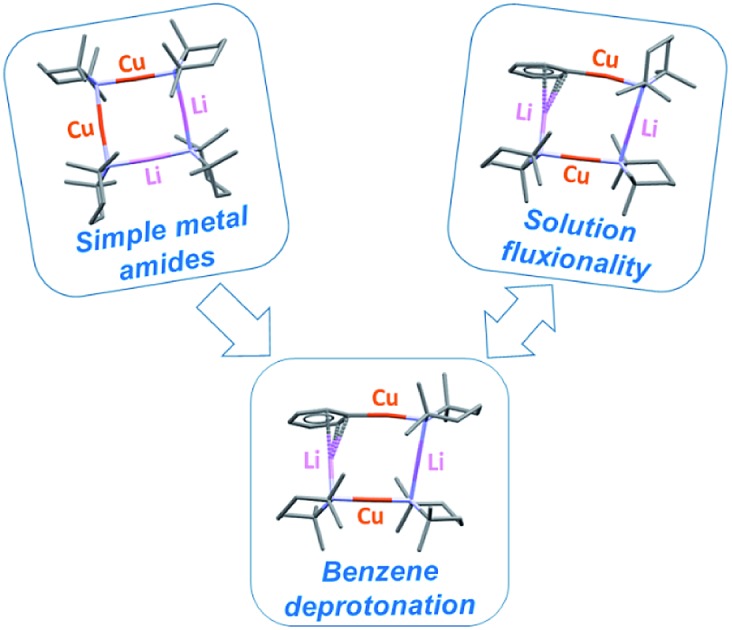
Efficient metalation of benzene is reported for the first time using reagents based only upon a mixture of a copper amide and a lithium amide.

## Introduction

The elaboration of functionalized aromatic compounds underwent little short of a revolution when it was reported, in 1999, that simply prepared lithium zincates could selectively deprotonate these rings whilst exhibiting remarkable levels of ancillary group tolerance.^1^ These data led to an explosion of interest in what have become known as ‘synergic bases’,^2^ heterobimetallic reagents of the type 
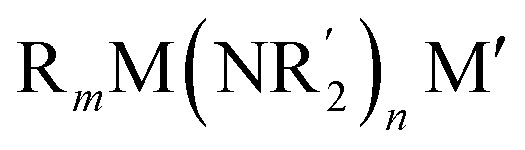
 (R, R′ = organyl; *m* = 0–3; M = more electronegative metal; 
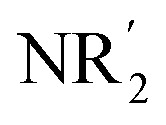
 = amide; *n* = 1–3; M′ = less electronegative metal). Over the subsequent two decades these bases have afforded levels of reactivity,^3^ regioselectivity^4^ and functional group tolerance^5^ not previously available using traditional main group organometallic bases, and they continue to evolve new applications.^6^ Most work in this field has focused on the directed *ortho* metalation of functional aromatics, in which M = Zn,^1^ Cr,^7^ Fe,^8^ Mg,^9^ Al,^10^ and Mn.^11^ Often, in these reactions M′ = Li and while organyl elimination has been observed and rationalized on thermodynamic grounds,^12^ experimental,^13^ spectroscopic^14^ and computational^15^ evidence points to a preference for *kinetic* amido basicity. Recourse to higher alkali metals (for M′) has allowed other major advances, for example synergic bases capable of offering non-traditional *meta*^16^ and *para*16c,^17^ directed metalation. In particular, whilst synergic bases incorporating Li afford new opportunities in directed deprotonation, much more reactive alkali metals^18^ have been required to achieve the highly desirable target of efficiently metalating *unfunctionalized* aromatic hydrocarbons, such as benzene,^19^ toluene^19^,^20^ and naphthalene.^21^ Taken together with the inability of traditional main group metal bases to efficiently activate such simple aromatics,^22^ it has become clear that opportunities remain for the development of metalating agents capable of activating unfunctionalized feedstock.

**Fig. 1:**
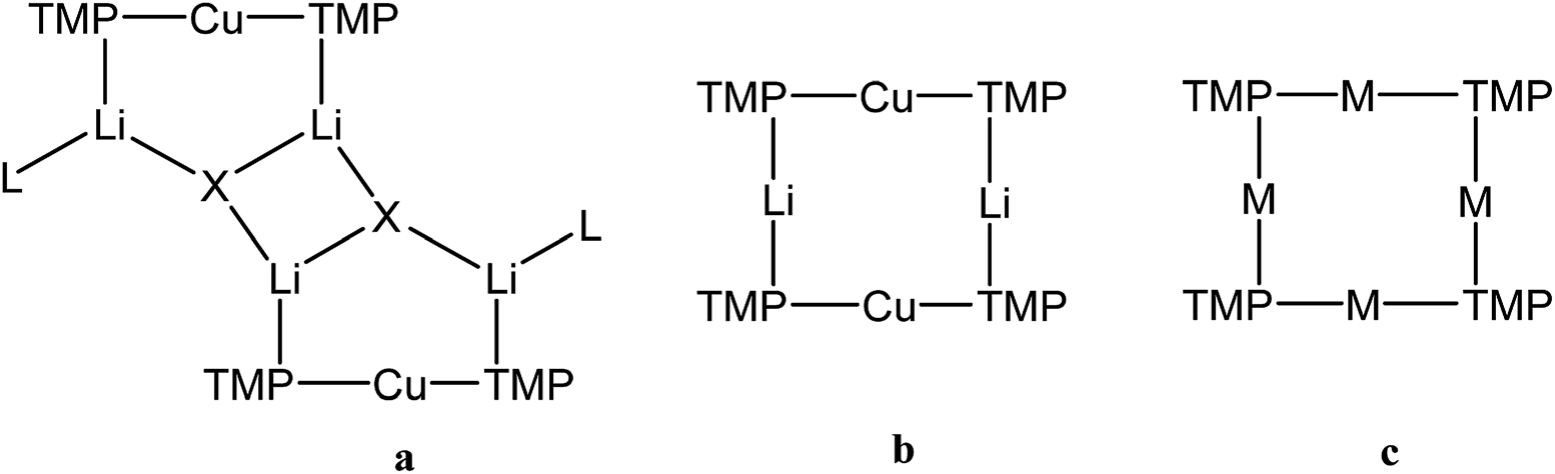
Structure-types known for bis(TMP)cuprates. (a) Lipshutz-type cuprates (X = CN, L = THF,^28^ Et_2_O,^23^ THP^27^ (THP = tetrahydropyran); X = halide, L = THF,^25^,^26^,^30^ L = Et_2_O;^23^ X = SCN, L = THF, Et_2_O, THP;^27^ X = OCN, L = THF^36^), (b) Gilman cuprate (TMP)_2_CuLi,^30^ and (c) mixed TMPLi-TMPCu aggregate (M = Li, Cu).^36^

One of the most fruitful heterobimetallic combinations employed in the selective activation of functionalized benzenoids^23^,^24^ and heteroaromatics^25^–^27^ is that of Cu with Li. This use of so-called lithium cuprates led to the emergence of directed *ortho* cupration (DoCu) as a synthetic strategy.^28^–^30^ These systems have been the subject of recent review,^31^ with the importance of employing a sterically demanding amide such as TMP (= 2,2,6,6-tetramethylpiperidide) having been noted.^23^,^32^ Though bis(TMP)cuprates themselves have been documented as being capable of DoCu, the inclusion of Li-salts (such as LiCN) to form what have been coined Lipshutz-type cuprates (Fig. 1a) has proven essential for efficient reactivity.^29^ This observation, combined with a debate over bonding of the salt anion,^33^ has meant that while Li-salt incorporating cuprates have been studied in some detail,^34^ Li-salt free cuprates have been subject to less exploration.^35^,^36^ To the best of our knowledge, only two examples of unsolvated bis(amido) Gilman cuprates, (amido)_2_CuLi, have been characterised in the solid state.^30^,^37^ In contrast to its Lipshutz-type analogues (TMP)_2_Cu(X)Li_2_ (X = CN, halide, SCN, OCN; Fig. 1a),^23^,^25^–^28^,^30^,^36^ the pre-isolated dimer of this Gilman cuprate is considered not to be reactive in DoCu.^30^

We recently reported the isolation of a *non-stoichiometric* cuprate (TMP)_4_Cu_2.7_Li_1.3_,^36^ which led us to suspect the existence, in solution, of a series of aggregates of TMPLi and TMPCu described by the general formula (TMP)_*m*+*n*_Cu_*m*_Li_*n*_ (*m* + *n* = 4). By extension, these data caused us to propose the existence of an isomeric variant on the dimer of a previously reported^30^ Gilman cuprate (Fig. 1b) in which metal ions of the same type, instead of being located *opposite* to one another in the 8-membered metallacyclic core, reside *adjacent* to one another.^36^ Herein we report on the isolation of this species and its characterisation in the solid state. In contrast to the previously reported Gilman cuprate, this new isomer exhibits unexpected reactivity with benzene. The ability to replicate this behaviour using *mixtures* of TMPLi and TMPCu, but not using either monometallic compound in isolation, points to cooperativity in solution. This process is studied by elucidating a series of Ph(TMP)_3_Cu_*m*_Li_*n*_ (*m* + *n* = 4) complexes, with results opening up the possibility of deploying easy-to-handle metal amides^38^ to smoothly deprotonate unfunctionalized aromatic feedstock.

## Results and discussion

### Synthesis and characterization of (TMPCu)_2_(TMPLi)_2_**1**

**Fig. 2:**
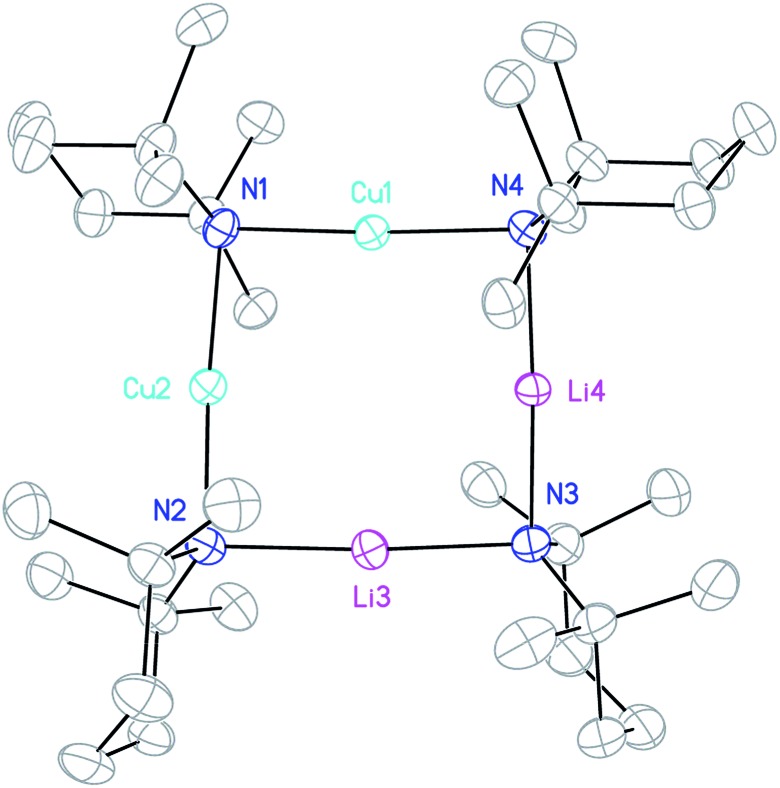
Thermal ellipsoid plot of **1** (30% probability). H-atoms and metal disorder omitted for clarity. Mean selected bond lengths (Å) and angles (°): N–Cu1 1.924, N–M2 1.978, N–Li3 2.065, N–M4 1.969, M–N–M 89.11, N–M–N 176.84.

In previous work, Lipshutz-type cuprates have typically been the focus of study due to their superior reactivity in DoCu when compared to pre-isolated Gilman cuprate.^29^ Accordingly, reaction conditions have been sought which favour their formation. These have been found to vary substantially with the choice of Cu(i) precursor. In the case of Lipshutz-type cuprate (TMP)_2_Cu(Cl)Li_2_(Et_2_O), appropriate conditions have been reported to be 2 : 1 LiTMP : CuCl in toluene followed by the introduction of Et_2_O prior to recrystallisation.^23^ We have now found that when the same reaction is performed in toluene containing limited Et_2_O (1 eq. wrt Li), a different crystalline material can be obtained. X-ray diffraction reveals a cyclic mixed-metal aggregate **1** which is isomorphous with previously reported mixed-metal aggregate (TMP)_4_Cu_2.7_Li_1.3_,^36^ but with an overall composition of (TMP)_4_Cu_2_Li_2_ (*i.e.* that of a Gilman cuprate) (Fig. 2). Elemental analysis indicated that the bulk composition of the product was consistent with the empirical formula (TMP)_2_CuLi.

Crystallographic refinement of **1** revealed that Cu2 and Li4 in Fig. 2 were almost perfectly disordered according to the pseudo two-fold symmetry of the molecule. This suggests an aggregate best formulated as (TMPCu)_2_(TMPLi)_2_ (Fig. 1c), which can be reasonably interpreted as an isomer of the previously characterized dimer of Gilman cuprate TMPCu(μ-TMP)Li **2** (Fig. 1b).^30^ In recent work, we have proposed the existence of this isomer from solution-state data.^36^ Evidence that this species corresponds to **1** as isolated in this work was gathered from ^1^H NMR spectroscopy. When crystalline **1** was dissolved in C_6_D_6_, the key spectroscopic features observed previously^36^ – namely the presence of three TMP-Me resonances, at *δ* 1.76, 1.57 and 1.39 ppm, in a 1 : 2 : 1 integral ratio – were reproduced (in addition to minor reformed TMPH, identified by a TMP-Me resonance at *δ* 1.06 ppm; see ESI, Fig. S1†). ^1^H,^1^H-NOESY reinforces the view that the solid-state structure of **1** is robust in solution by demonstrating a lack of exchange peaks between Me-groups at room temperature. ^7^Li NMR spectroscopy revealed a dominant singlet, at *δ* 1.64 ppm, alongside a very minor resonance, at *δ* 0.95 ppm. The former of these is consistent with the single Li-environment in **1** whereas the minor resonance has previously been interpreted as belonging to a Cu-rich species such as (TMP)_4_Cu_3_Li.^36^^13^C NMR spectroscopy revealed a spectrum consistent with the three different TMP environments in **1**, most clearly evidenced by the TMP-2,6 carbon resonances, at *δ* 56.9, 54.2 and 52.0 ppm. These correspond to TMP ligands bonded to two Cu centres, one Cu and one Li centre and two Li centres, respectively.

### Reactivity of **1** towards unfunctionalized aromatics

**Fig. 3:**
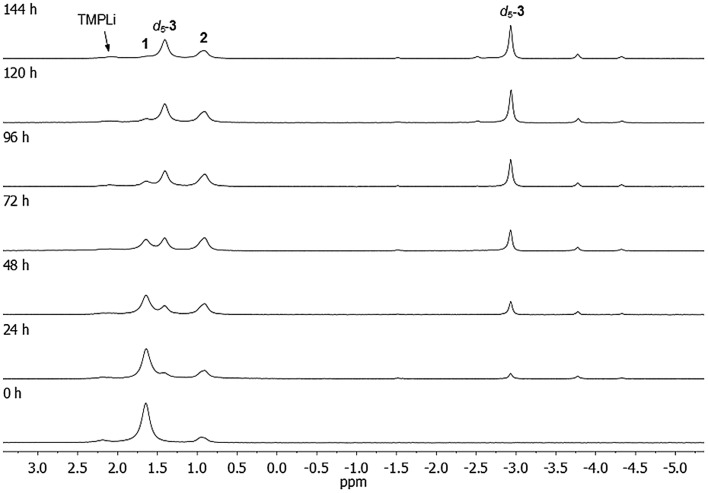
^7^Li NMR spectra of **1** in C_6_D_6_, heated to 50 °C for 0–144 h. For details on minor, unlabelled peaks, see text.

In previous work, it was hypothesised that **1** might be the kinetic form of (TMP)_4_Cu_2_Li_2_ and that it should convert to the thermodynamic isomer (Gilman dimer **2**_2_) under suitable conditions.^36^ To test this, crystalline **1** was dissolved in C_6_D_6_ and heated to 50 °C. The composition of the mixture was monitored at regular intervals by *in situ* NMR spectroscopy. This established the expected progressive loss of **1**. However, instead of revealing the anticipated proportional growth of Gilman cuprate, a number of additional Li-containing species were observed, characterised by unusual high-field ^7^Li resonances (Fig. 3). Furthermore, the quantity of free amine present in the reaction mixture (observed by ^1^H NMR spectroscopy) was far in excess of what could reasonably be attributed to hydrolysis by adventitious moisture. In any case, the absence of an N–H signal (typically observed by ^1^H NMR at *δ* 0.3 ppm) suggested that this may be *deuterated* free amine TMP*D* rather than TMP*H*. These observations suggested the intriguing possibility of a metal–deuterium exchange reaction occurring between **1** and the NMR solvent. Logically, this would result in the generation of aryl anions (C_6_D_5_^–^), whose π-type interaction with Li^+^ could then account for the observed high-field ^7^Li NMR resonances (as previously reported in aryl(amido)cuprates).^39^–^41^

The ^7^Li NMR spectrum of the final reaction mixture (Fig. 3, top) reveals a subsidiary peak at *δ* 0.90 ppm, identified as **2**, and two dominant resonances, at *δ* 1.41 and –2.93 ppm (in a 1 : 1 integral ratio). The former of these two signals falls within the chemical shift range expected for a Li^+^ center bridging two amido ligands (as observed previously in amidocuprates).^36^ On the other hand, the resonance *δ* –2.93 ppm indicates some degree of Li···π interaction.^39^–^41^ These data point towards the presence of a well-defined organo(amido)cuprate, though symmetry indicates this not to be a simple homodimer of the type [ArCu(μ-TMP)Li]_2_ (Ar = C_6_D_5_). This view is substantiated by data obtained from the reaction of **1** with C_6_H_6_ solvent under similar conditions, for which ^1^H NMR spectroscopy on the reaction mixture shows both TMP ligands and a number of aromatic hydrogens (*δ* 7.9–6.7 ppm), the splitting pattern of the latter suggesting a Ph group. Integration suggests an amido : phenyl ratio of 3 : 1. Furthermore, removal of the volatiles, followed by recrystallisation from toluene provided material free from TMPH, establishing that this potentially Lewis basic by-product of aromatic deprotonation does not coordinate the organometallic product in this system. ^7^Li NMR spectroscopy on the same material reveals singlets, at *δ* 1.41 and –2.86 ppm, in a 1 : 1 integral ratio – nearly identical to those observed in the deuterated system (Fig. 3) – overall suggesting the formula of the major product to be Ph(TMP)_3_Cu_2_Li_2_**3**.

### Probing Ph(TMP)_3_Cu_2_Li_2_**3**: synthesis and characterization of PhCu(μ-TMP)Li **4**

**Fig. 4:**
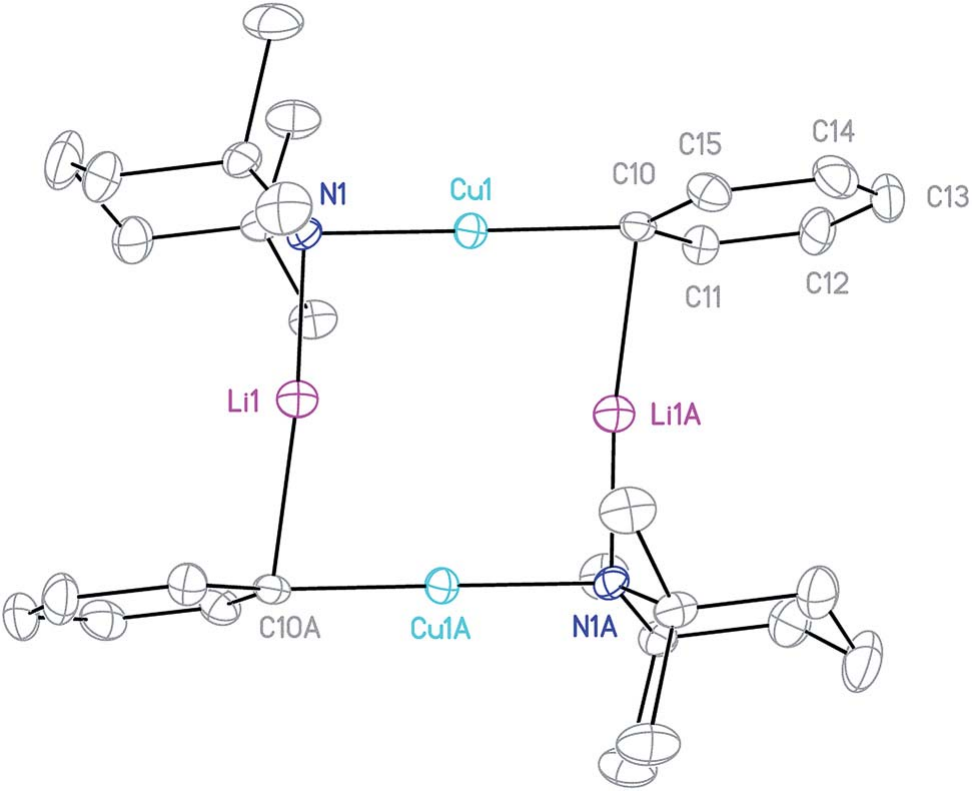
Thermal ellipsoid plot (30% probability) of **4**_2_. H-atoms omitted for clarity. Selected bond length (Å) and angles (°): Cu1–N1 1.892(2), Li1–N1 1.932(4), Cu1–C10 1.904(2), Li1–C10A 2.153(4); Cu1–N1–Li1 92.55(14), C10–Cu1–N1 178.71(8), Cu1–C10–Li1A 81.33(13), and N1–Li1–C10A 169.4(2).

Crystals obtained directly from the reaction mixture described above proved to be of poor quality. An alternative method of preparing **3** for structural characterisation was therefore sought. The realisation that **3** is a heterodimer of TMPCu(μ-TMP)Li and PhCu(μ-TMP)Li suggested that the combination of these reagents might result in equilibration to the desired product. Testing this hypothesis necessitated the synthesis of solvent-free PhCu(μ-TMP)Li. This compound has been isolated previously as a monomeric tris-(THF) solvate, synthesised through the combination of PhCu in THF with TMPLi.^29^ Given the known difficulty of removing THF from such preformed complexes, an alternative method was devised. Solvent- and salt-free PhCu is known to be very difficult to isolate, so we opted to synthesise PhLi and then combine it with *in situ*-generated TMPCu in a hydrocarbon solvent. Remarkably, the insoluble PhLi dissolved very rapidly to give a yellow solution, which, after concentration and chilling, yielded prismatic orthorhombic crystals. X-ray crystallography revealed a homodimer of the expected heterocuprate, [PhCu(μ-TMP)Li]_2_**4**_2_ (Fig. 4). The coordination of Li differs significantly from that seen in the small number of previously reported cuprates of this type. While homodimer cuprates [MesCu(NR_2_)Li]_2_ (R_2_N = TMP, N(CH_2_Ph)_2_) exhibit exclusively η^6^-hapticity towards Li,^39^–^41^ this is not observed in **4**_2_. In this complex, Li1A is canted away from the Ph-group (Cu1–C10–Li1A 81.33(13)°) and appears to interact primarily with *ipso* carbon C10 (Li1A–C10 2.153(4) Å). Meanwhile, interaction with the *ortho* carbons C11 and C15 is weaker and distinctly asymmetrical (Li1A–C11 2.594(4), Li1A–C15 2.826(4) Å), suggesting that the Ph-group in **4**_2_ is best regarded as η^1^. Though unusual, DFT calculations (see later) nonetheless confirm that η^1^-arene coordination by lithium is energetically preferred in this case.

^1^H NMR spectroscopy on **4** in C_6_D_6_ solution revealed a single set of resonances attributable to Ph and TMP moieties, though the observation of two TMP-Me resonances (1 : 1 integral ratio) suggested retention of a static conformation for the amido ligand. Meanwhile, a dominant resonance was observed at *δ* –2.43 ppm by ^7^Li NMR spectroscopy, consistent with retention of the low-hapticity Li···π interactions found in the solid-state dimer.

**Fig. 5:**
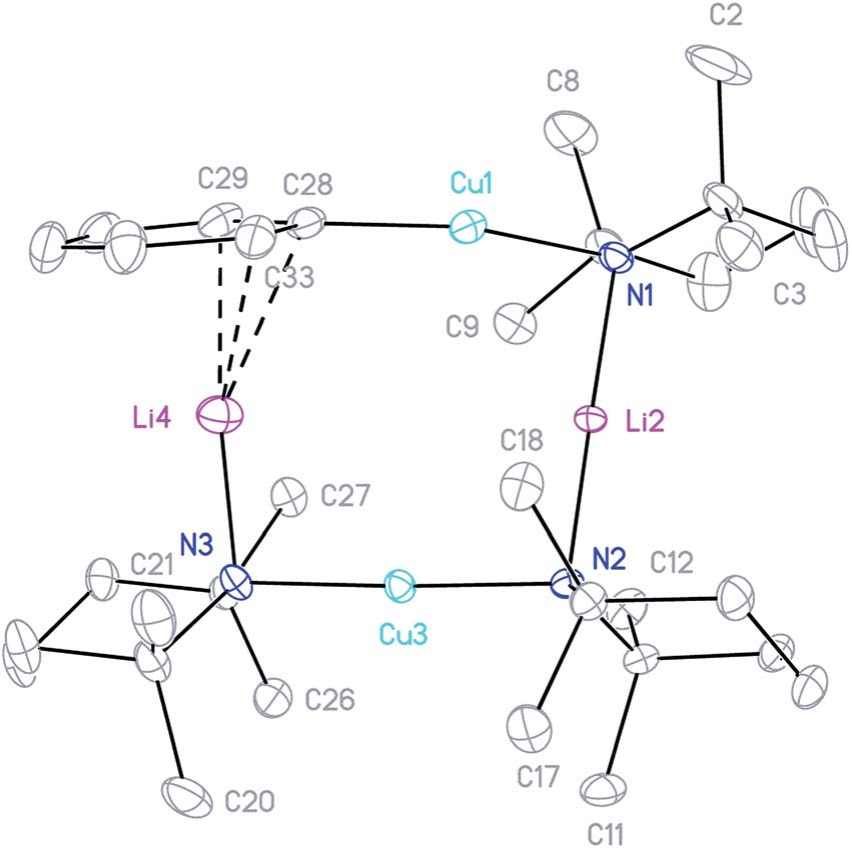
Thermal ellipsoid plot (30% probability) of **3**. H-atoms omitted for clarity. Selected bond lengths (Å) and angles (°): Cu1–C28 1.895(2), Cu1–N1 1.907(2), Li2–N1 2.047(3), Li2–N2 2.022(3), Cu3–N2 1.913(2), Cu3–N3 1.915(2), Li4–N3 1.971(4), Li4–C28 2.348(4), Li4–C29 2.380(4), Li4–C33 2.431(4); C28–Cu1–N1 168.97(8), Cu1–N1–Li2 81.53(10), N1–Li2–N2 176.45(18), Li2–N2–Cu3 94.04(10), N2–Cu3–N3 177.11(7), and Cu3–N3–Li4 91.48(13).

The combination of crystalline **4** with TMPCu(μ-TMP)Li **2** (1 : 1 molar ratio) in C_6_D_6_ was followed by screening of the reaction mixture for the formation of Ph(TMP)_3_Cu_2_Li_2_**3** using *in situ* NMR spectroscopy. This failed to reveal any changes upon mixing at room temperature, but indicated substantial levels of conversion after 24 h at 50 °C. As a result, the same reaction was then attempted on a larger scale, using toluene as the solvent. Replacement of this solvent with hexane, and chilling of the resulting solution to 5 °C gave a crop of block-like crystals. X-ray diffraction revealed a metallacycle, featuring Ph and TMP ligands in the expected ratio, confirming the successful fabrication of **3** (Fig. 5).

The crystal structure of **3** supports its interpretation as a heterodimer and establishes a higher arene coordination mode than observed in **4**_2_. Li4 interacts in a π-fashion with Ph-carbons in an asymmetrical manner best described as η^3^. Retention of this feature in solution would explain the spectroscopic observation of reduced shielding of the arene-bound Li in **3** (^7^Li NMR: *δ* –2.86 ppm) relative to the η^6^-coordination seen previously in lithium aryl(amido)cuprates.^39^–^41^ The Li–π interactions in **3** have a wide-reaching influence upon the structure. In contrast to **4**_2_, the C–Cu–N unit deviates significantly from linearity, allowing the Ph to incline towards Li4. The framework twists to accommodate this geometry, with a mean deviation from the plane of the metallacyclic ring (which is taken to incorporate C28) of 0.3046 Å (ESI, Fig. S3a†). This contrasts significantly with **4**_2_, which was much more planar (mean deviation from the plane of the metallacycle of 0.0565 Å). Lastly, it was noted previously that there is a preference for the so-called *endo* orientation of TMP in cuprates incorporating this ligand^37^ and it is interesting to note that this feature is also preserved in the dimer of **4** and heterodimer **3**.

### Beyond Ph(TMP)_3_Cu_2_Li_2_**3**: synthesis and characterization of Ph(TMP)_3_Cu_*m*_Li_*n*_ (*m* = 3, *n* = 1 5; *m* = 4, *n* = 0 6; *m* = 1, *n* = 3 7)

We next considered whether other species with the same general structure as **3**, but with different metal content, might exist and whether these might potentially account for some of the minor products present in the **1**–C_6_H_6_ reaction mixture. Logically, this could be achieved by combining TMPCu or TMPLi with PhCu(μ-TMP)Li **4** in a 2 : 1 ratio. Preliminary *in situ* NMR experiments confirmed that re-aggregation took place. When TMPCu was used, heating to 50 °C for 24 h was necessary to achieve this. However, heating was not necessary for the analogous reaction using TMPLi.

**Fig. 6:**
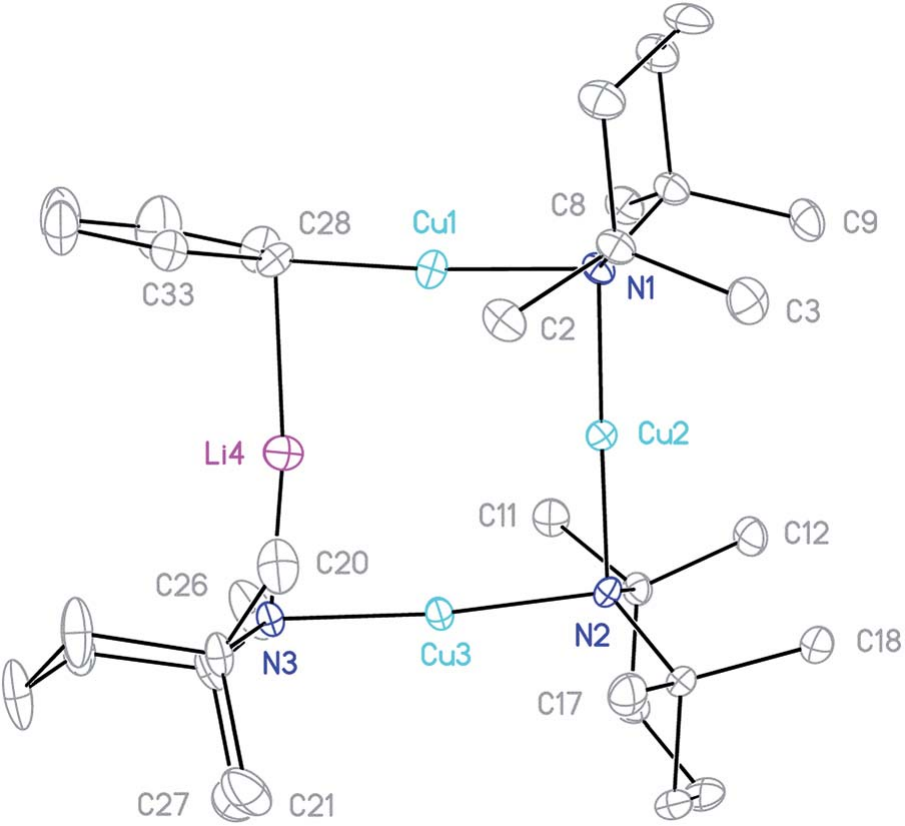
Thermal ellipsoid plot (30% probability) of **5**. H-atoms and minor metal disorder omitted for clarity. Selected bond lengths (Å) and angles (°): Cu1–C28 1.915(4), Cu1–N1 1.934(3), Cu2–N1 1.933(3), Cu2–N2 1.916(3), Cu3–N2 1.940(3), Cu3–N3 1.918(3), Li4–N3 1.888(5), Li4–C28 2.204(5); C28–Cu1–N1 176.35(17), Cu1–N1–Cu2 86.40(13), N1–Cu2–N2 178.23(14), Cu2–N2–Cu3 91.31(13), N2–Cu3–N3 174.31(14), Li4–N3–Cu3 83.89(17), N3–Li4–C28 172.4(3), and Cu1–C28–Li4 81.85(17).

In the same manner as described above for the synthesis of **3**, a 2 : 1 combination of TMPCu and **4** in toluene provided a crystalline material after concentration of the solution. X-ray diffraction revealed a Cu-rich cuprate with composition Ph(TMP)_3_Cu_3.12_Li_0.88_, which can be regarded as Ph(TMP)_3_Cu_3_Li **5**, subject to a small amount of Cu–Li substitution^42^ at the sole Li-containing site (Fig. 6).

**Scheme 1:**
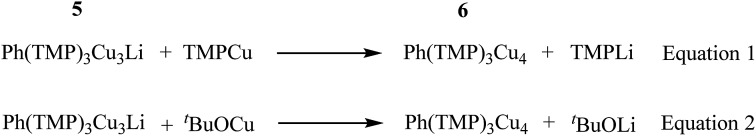
Cu–Li exchange reactions leading to the formation of **6**.

The metallacycle in **5** is similar to that in **3**, whilst the Ph coordination mode is clearly more akin to that of **4**_2_. In **5**, C–Li bond distances (Li4–C28 2.204(5) and Li4–C33 2.656(6) Å) suggest η^1^-coordination to the *ipso* carbon (*cf.***4**_2_). Regarding substitutional Cu–Li disorder, even though site Li4 in the present structure contains only a small amount of Cu (12% by crystallography with NMR spectroscopy suggesting similar levels in bulk samples), its presence implies the existence of a purely Cu-based homologue: Ph(TMP)_3_Cu_4_**6**. It was speculated that trace amounts of **6** could arise from Cu–Li exchange between **5** and TMPCu, expelling TMPLi (Scheme 1, eqn (1)). To attempt to prepare **6** in isolable quantities, we opted to conduct this exchange using the previously reported strategy of Cu^I^–O/Li–O bond metathesis.^43^*In situ* NMR spectroscopy on a 1 : 1 mixture of ^*t*^BuOCu with **5** in C_6_D_6_ (which developed a green colouration upon heating to 50 °C) revealed formation of **6** and ^*t*^BuOLi (Scheme 1, eqn (2); see ESI, Fig. S5j–S5l†). Scaling-up this reaction in toluene gave a dark green reaction mixture from which colourless crystals of **6** could be isolated in low yield.

**Fig. 7:**
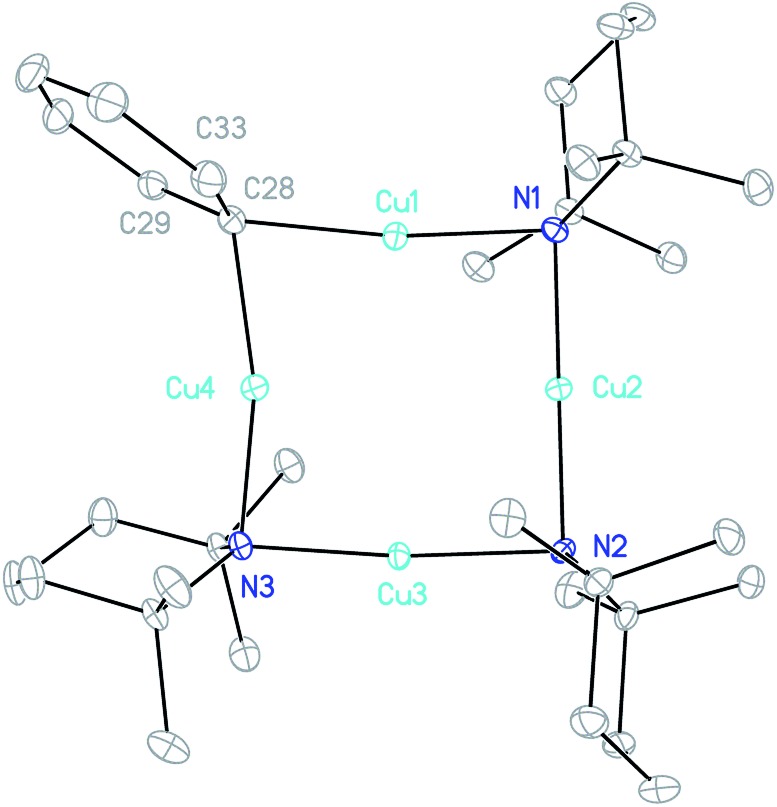
Thermal ellipsoid plot (30% probability) of **6**. Selected bond lengths (Å) and angles (°): Cu1–N1 1.948(3), N1–Cu2 1.934(4), Cu2–N2 1.929(3), N2–Cu3 1.939(4), Cu3–N3 1.921(4), N3–Cu4 1.948(4), Cu4–C28 1.992(4), C28–Cu1 1.971(4); C28–Cu1–N1 171.81(17), Cu1–N1–Cu2 84.97(14), N1–Cu2–N2 178.64(15), Cu2–N2–Cu3 89.16(14), N2–Cu3–N3 84.25(15), N3–Cu4–C28 167.12(17), and Cu4–C28–Cu1 75.45(15).

X-ray crystallography on **6** (Fig. 7) reveals a structure that is nearly superimposable with that of **5**. The major difference between the two lies in the position of Ph, which adopts a more symmetrical bridging mode in **6** than in **5**. The M1–C28–M4 (M1 = Cu; M4 = Li **5**, Cu **6**) angle is correspondingly reduced (Cu1–C28–Li4 81.28(11)° in **5** and Cu4–C28–Cu1 75.45(15)° in **6**), bringing it well within the range known for tetrameric arylcopper species (70.1–77.5°).^44^ This presumably reflects a greater level of covalency in the C–Cu bond compared to that of C–Li, a suggestion which is supported by the significant shortening of the Cu1–C28 interaction when Ph bridges Cu and Li as opposed to Cu only (Cu1–C28 1.895(2) Å in **3**, 1.915(4) Å in **5** and 1.971(4) Å in **6**).

In contrast to the previous reactions, when TMPLi was combined with PhCu(μ-TMP)Li **4** at room temperature in hexane a viscous oil was obtained which repeatedly failed to deposit crystals; multiple attempted crystallisations from pentane also failed. However, removal of all volatiles gave a white solid which was confirmed to be Ph(TMP)_3_CuLi_3_**7** by ^1^H NMR spectroscopy through the observation of Ph and TMP resonances in a 1 : 3 ratio, in addition to ^7^Li NMR signals at *δ* 2.09 and –4.26 ppm in a 2 : 1 ratio.

### *In situ* synthesis of **3**, **5** and **7**

**Fig. 8:**
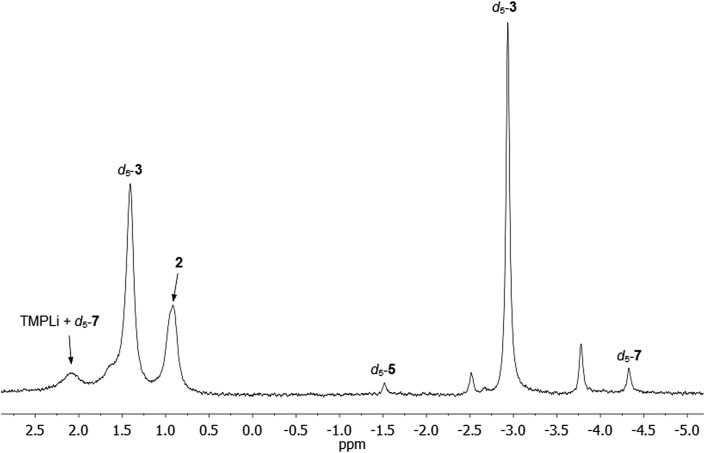
^7^Li NMR spectrum of **1** in C_6_D_6_, heated to 50 °C for 144 h. d_5_-**5** and d_5_-**7** can be identified as minor products.

Comparison of the NMR spectra of **3**, **5**, and **7** with the *in situ* data for the final reaction mixture of **1** in C_6_D_6_ revealed that d_5_-**5** and d_5_-**7** constitute minor products in this system. In addition, there were two other Ph-containing species, though the proportion of these species is too small to permit confident assignment from NMR data (Fig. 8).

**Fig. 9:**
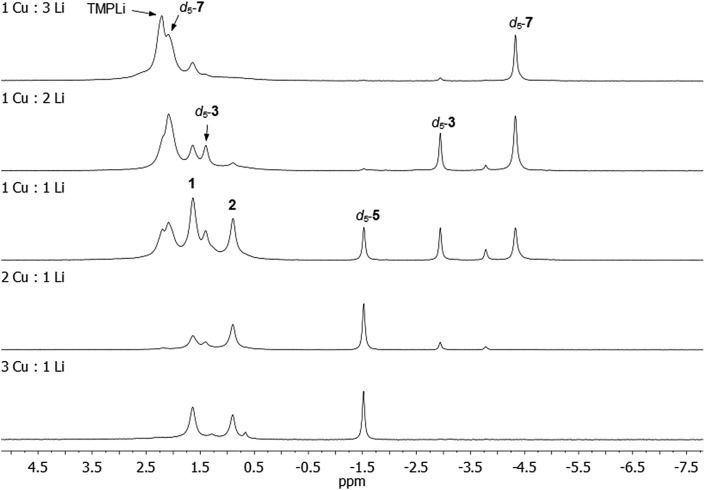
^7^Li NMR spectra in C_6_D_6_ of reaction mixtures containing TMPCu and TMPLi in the ratios specified. Reaction mixtures were heated in C_6_D_6_ to 50 °C for *ca.* 24 h in a sealed NMR tube before data acquisition.

Our attention next turned to eliciting whether **3**, **5** and/or **7** could be generated selectively *via* deprotonative means. It was clear from *in situ* work that the Cu : Li ratio (of 1 : 1) in **3** reflected that of the starting material **1**. Moreover, our interpretation of **1** as a molecular mixed aggregate of TMPLi and TMPCu raised the possibility that reactivity akin to that shown by **1** might be exhibited by a physical mixture of these two amides. Such an approach would remove the restrictions in the Cu : Li stoichiometry imposed by **1**. To test this hypothesis, pre-isolated, crystalline TMPCu and TMPLi^45^ were combined in C_6_D_6_ (in a sealed NMR tube) and heated to 50 °C for *ca.* 24 h. The TMPCu : TMPLi molar ratio in the starting mixture was varied in each of the five experiments, 3 : 1, 2 : 1, 1 : 1, 1 : 2, and 1 : 3. Analysis of the resulting reaction mixtures by ^7^Li NMR spectroscopy revealed multiple products – including d_5_-**3**, d_5_-**5**, and d_5_-**7** – and a gradual transition of the dominant organo(amido)cuprate from d_5_-**5**, through d_5_-**3**, to d_5_-**7** as Cu was replaced by Li in the reaction mixture (Fig. 9; see also ESI, Fig. S17a and b†). While **1** in C_6_D_6_ showed low levels of d_5_-**3** after 24 h at 50 °C, all physical mixtures of TMPCu and TMPLi generated much higher levels of organometallic products d_5_-**3**, d_5_-**5** and/or d_5_-**7** after the same period of time at 50 °C, implying a faster rate of reaction. Nonetheless, the presence of residual TMPCu–TMPLi aggregates suggested that longer reaction times might be beneficial to achieve optimal conversion (especially in deuterated solvents, which might be expected to react more slowly than their protic counterparts).

Further experiments on a larger scale reinforced our hypothesis: equimolar TMPCu and TMPLi were combined in C_6_H_6_ and heated to 50 °C for *ca.* 5 days, after which time the volatiles were removed. ^7^Li NMR spectroscopy on the residue (in C_6_D_6_) indicated a composition of mainly **3** (85%), the remainder being **2** (4%) and other Ph-containing species (11%), including **4** and **5** (see ESI, Fig. S18c†). This contrasts with the 1 : 1 reaction in Fig. 9, where the levels of d_5_-**3**, d_5_-**5** and d_5_-**7** were much more evenly distributed.

**Scheme 2:**
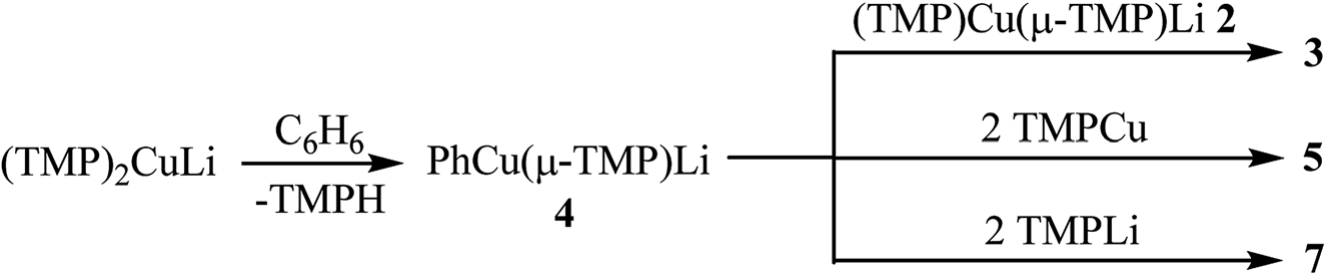
Reactions explaining the production of **3**, **5** and **7**.

Considering the observations outlined above and in the preceding sections, it seems likely that the reaction pathways leading to the formation of **3**, **5** and **7** involve *at least* two steps. This is suggested by the fact that **4** is explicitly demonstrated to react with TMPLi–TMPCu aggregates but is not itself observed in significant quantities in the *in situ* reaction mixtures examined. A plausible sequence of reactions would therefore involve (1) reaction of **1** or components thereof with benzene and (2) subsequent re-equilibration with the remaining **2**, TMPCu or TMPLi to generate **3**, **5**, or **7**, respectively (Scheme 2). Though the nature of the active base in these reactions is not certain, control experiments in which either TMPCu or TMPLi was heated in C_6_D_6_ to 50 °C revealed no substantial changes by NMR spectroscopy after 24 h (see ESI, Fig. S14a–c and S15a–b†), suggesting synergistic activity instead. Furthermore, pre-isolated **2** (known to be a dimer in the solid state) was also inactive under these conditions (see ESI, Fig. S16a–c†). In light of this, it is suggested that a smaller cuprate aggregate (possibly a monomer of **2**: TMPCu(μ-TMP)Li or TMPLi(μ-TMP)Cu) could act as the base, as has been suggested previously by DFT calculations in relation to DoCu chemistry.^30^

### DFT calculations

DFT calculations (M06/6-31+G* & SVP(Cu)) were undertaken to assess the energies of structures for (TMP)_4_Cu_2_Li_2_ isomers **1** and **2**_2_, Ph(TMP)_3_Cu_2_Li_2_**3**, [PhCu(μ-TMP)Li]_2_**4**_2_, Ph(TMP)_3_Cu_3_Li **5**, and expected Ph(TMP)_3_CuLi_3_**7**. This was done with a view to predicting the structural features of **7**, for which no crystals could be obtained. Efforts focussed first on the two structural isomers of the formulation (TMP)_4_Cu_2_Li_2_, amide aggregate **1** and Gilman cuprate dimer **2**_2_. Analysis confirmed the Gilman cuprate dimer to be the thermodynamically preferred isomer, but by only 2.5 kcal mol^–1^ (see ESI, Fig. S8 and S9†).

Subsequently, the remainder of the structures were assessed, whereby the energy of all possible conformers (generated by individually flipping each amido ligand from *exo* to *endo* or *vice versa*)^23^ was evaluated (see ESI, Fig. S10 to S13†). For models based on the formulation Ph(TMP)_3_Cu_3_Li (**5**), the preferred conformer was found to be 6.3 kcal mol^–1^ lower in energy than the most unstable one, and corresponded exactly to that observed experimentally by X-ray diffraction. The experimentally seen low-hapticity coordination mode of the arene is reproduced satisfactorily and differs little between Ph(TMP)_3_Cu_3_Li conformers. In a similar vein, for models based on the formulation Ph(TMP)_3_Cu_2_Li_2_ (**3**), the energy difference between the conformers was relatively small (5.5 kcal mol^–1^). Again, the conformer observed in the solid-state proved to be the most stable calculated one. As for attempts to model **5**, modelling of **3** led to the coordination mode of the arene (approximately η^3^) again being quite accurately reproduced by theory.

In spite of the demonstrable ability of DFT calculations to replicate the experimentally observed structures of **1**, **3** and **5**, attempts to predict the conformation of Ph(TMP)_3_CuLi_3_ (**7**) proved difficult. In this case, DFT work revealed a much smaller range of energies associated with the different conformers (only a 2.0 kcal mol^–1^ difference between the highest and lowest energy forms). However, in all modelled conformers, a high hapticity arene coordination mode was predicted (approximately η^6^) and this is consistent with the significant shielding of the Li-centre seen by NMR spectroscopy.

### NMR spectroscopy

Further insights into the structures of **3**, **5** and **7** in hydrocarbon solution have been gathered using 2D NMR spectroscopy. Analysis of ^1^H,^1^H-NOESY and ^1^H,^7^Li-HOESY data corroborates the maintenance, in solution, of structure-types exemplified by **3** and **5** in the solid-state. However, extension of this analysis to **7** (for which solid-state data could not be obtained) suggests a degree of fluxionality not observed in **3** or **5**.

**Fig. 10:**
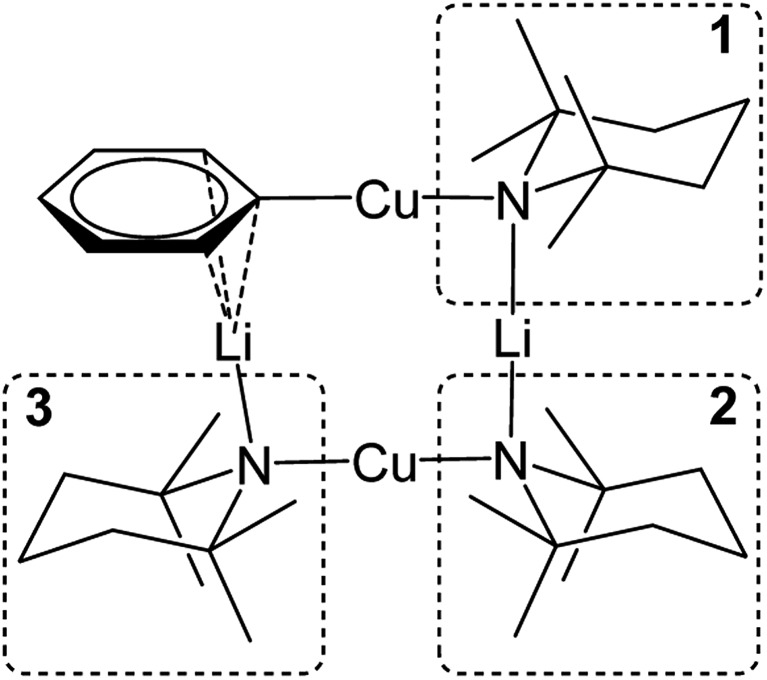
Labelling scheme for **3** in solution, based upon the solid-state structure for the same compound.

**Fig. 11:**
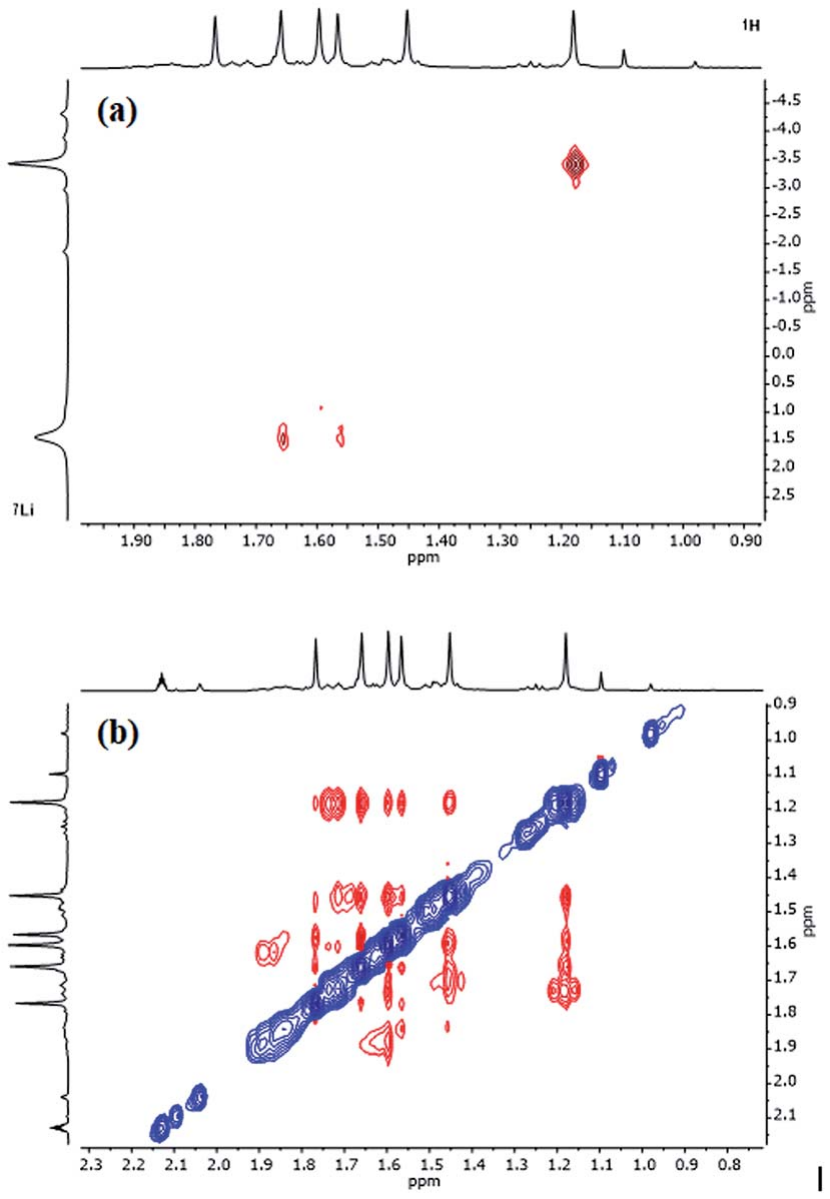
Selected NMR data for **3** in C_7_D_8_ at –10 °C. (a) ^1^H,^7^Li-HOESY spectrum (*τ* = 0.05 s); (b) ^1^H,^1^H-NOESY spectrum (*τ* = 0.6 s).

^1^H,^1^H-NOESY data on **3** (see Fig. 10 for the labelling scheme) in C_6_D_6_ at ambient temperature revealed exchange correlations. However, these were eliminated at –10 °C, at which temperature in C_7_D_8_, ^1^H,^7^Li-HOESY data (Fig. 11a) revealed correlations at *δ*(^1^H, ^7^Li) = (1.66, 1.44), (1.56, 1.44) and (1.18, –3.43) ppm (see ESI, Fig. S3f and S3g†). The last of these is consistent with the short TMP^3^-Me_eq_···Li contact expected from the solid-state data (*viz.* C21···Li4 2.874(5) Å in Fig. 5), whilst the remaining correlations must arise from TMP^1^-Me_eq_···Li contacts (C3···Li2 2.775(4) Å) and TMP^2^-Me_eq_···Li contacts (C12···Li2 2.707(3) Å in Fig. 5). ^1^H,^1^H-NOESY data obtained at –10 °C confirmed the spatial proximity of the hydrogen atoms of TMP^3^-Me_eq_ to *both o*- and *m*-hydrogens of the Ph moiety (C27···C29 3.803(3) Å, C21···C32 3.897(3) Å in Fig. 5; see ESI, Fig. S3f†). Based upon the conformation observed in the solid-state, further NOEs are predicted between the following pairs of Me-groups (in addition to NOEs between inequivalent pairs of Me-groups within each amido ligand): TMP^1^-Me_eq_···TMP^2^-Me_eq_ (C3···C18 3.850(3) Å), TMP^2^-Me_eq_···TMP^3^-Me_eq_ (C21···C18 4.131(3) Å), TMP^2^-Me_eq_···TMP^3^-Me_ax_ (C12···C26 4.458(3) Å), TMP^2^-Me_ax_···TMP^3^-Me_eq_ (C17···C21 4.112(3) Å), TMP^2^-Me_ax_···TMP^3^-Me_ax_ (C20···C17 4.324(3) Å), and TMP^1^-Me_eq_···TMP^3^-Me_eq_ (C9···C27 3.549(3) Å). Analysis of ^1^H,^1^H-NOESY data obtained at –10 °C revealed these predicted correlations (Fig. 11b), allowing the ^1^H NMR resonance at *δ* 1.66 ppm to be assigned to TMP^1^-Me_eq_, and that at *δ* 1.56 ppm to TMP^2^-Me_eq_ (see ESI, Fig. S3f†).

**Fig. 12:**
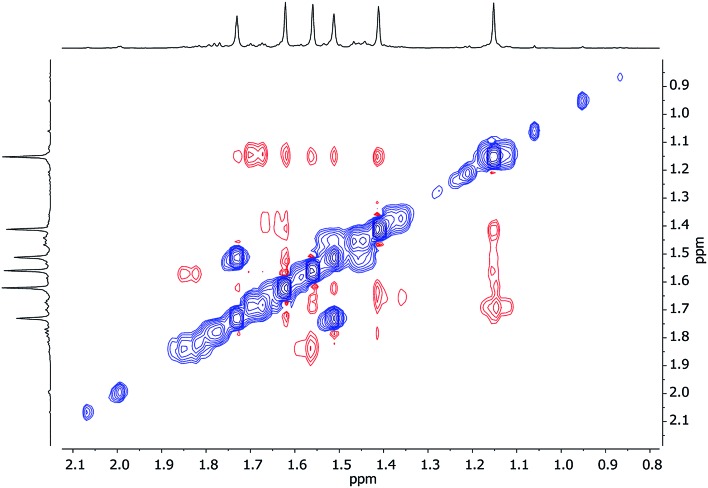
^1^H,^1^H-NOESY spectrum of **3** in C_6_D_6_ at 25 °C; exchange correlations in blue (*τ* = 0.6 s).

In contrast to the low-temperature data, ^1^H,^1^H-NOESY results on **3** at ambient temperature (25 °C) revealed exchange correlations between the axial and equatorial hydrogens of TMP^1^ (in both its Me groups and its ring; Fig. 12 and S3h†). This required (at least in part) ring inversion of TMP^1^, which is notable since DFT calculations predicted the resulting conformation of **3** to be the most energetically accessible conformer above the ground state (see ESI, Fig. S10a†).

**Fig. 13:**
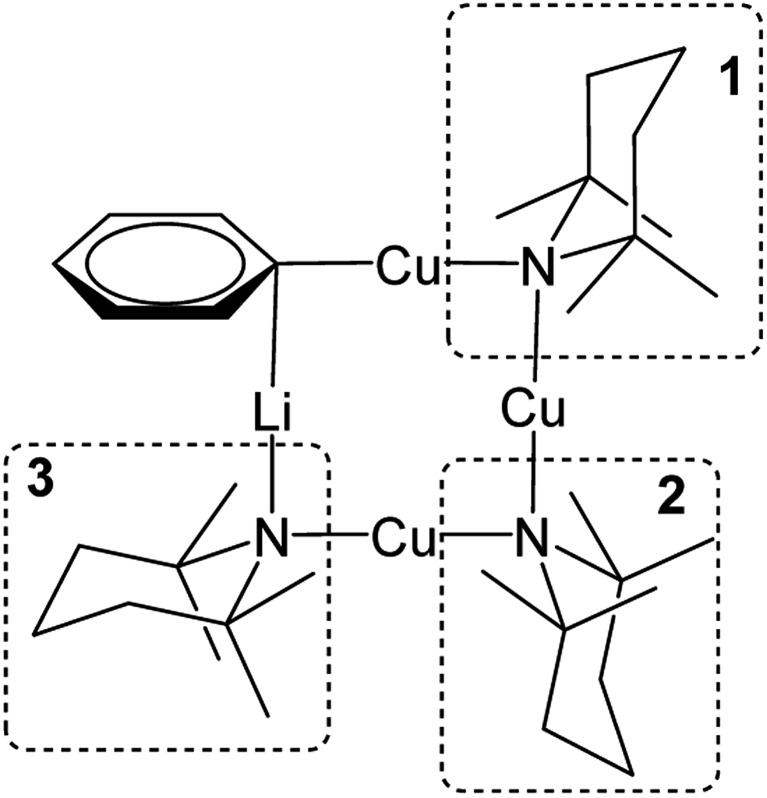
Labelling scheme for **5** in solution, based upon the solid-state structure for the same compound.

**Fig. 14:**
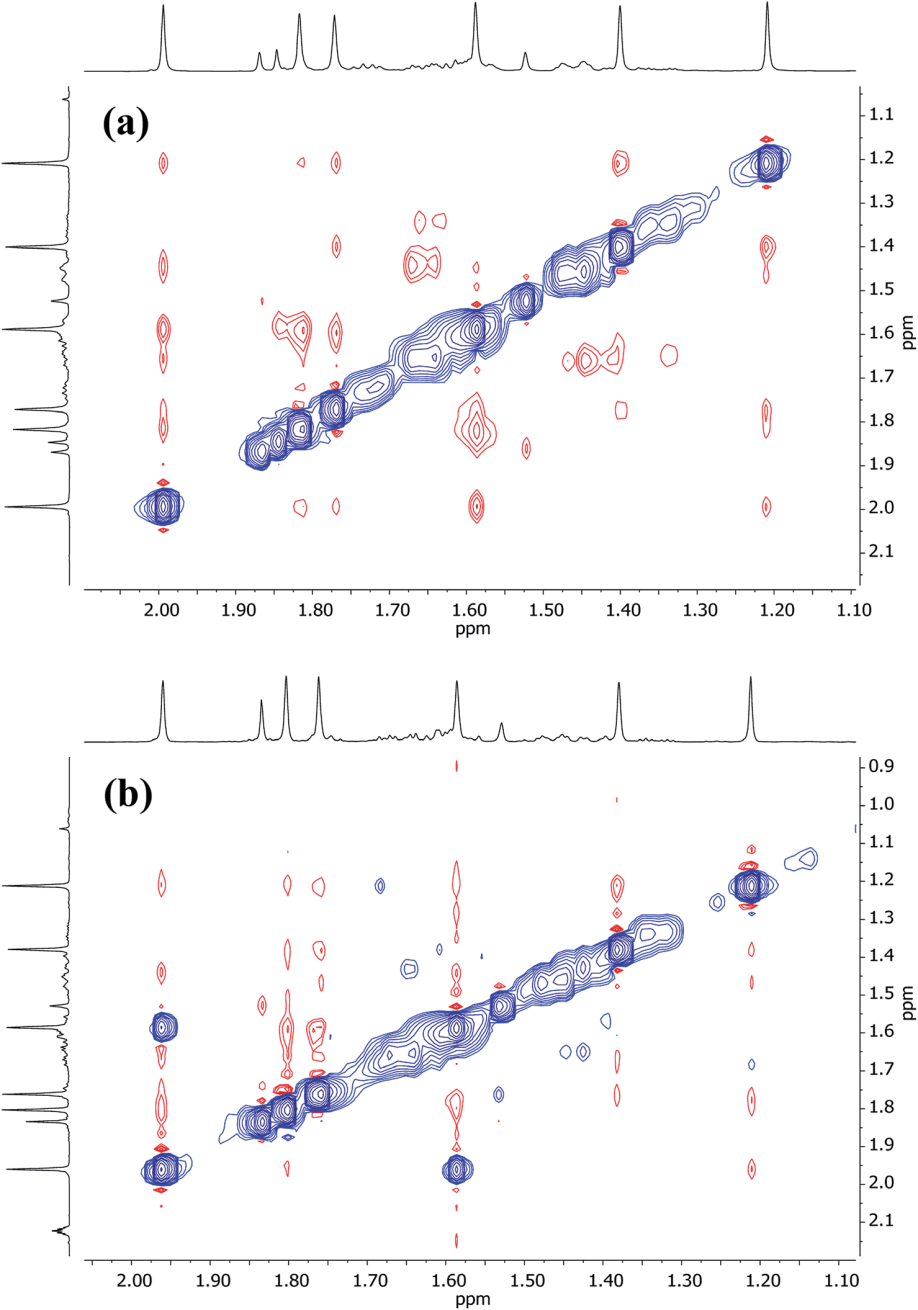
Expansions of ^1^H,^1^H-NOESY spectra for **5** in (a) C_6_D_6_ at 25 °C, and (b) C_7_D_8_ at 80 °C; exchange correlations in blue (*τ* = 0.6 s).

Turning to Cu-rich **5** (Fig. 13 and S5d†), ^1^H,^7^Li-HOESY data obtained in C_6_D_6_ at room temperature revealed one correlation at *δ*(^1^H, ^7^Li) = (1.21, –1.46) ppm (see ESI, Fig. S5g†), allowing assignment of TMP^3^-Me_eq_ (*viz.* C26···Li4 2.801(6) Å in Fig. 6). The remaining Me-groups could be assigned by their proximity to the *o*-Ph hydrogens and to each other, using ^1^H,^1^H-NOESY data (Fig. 14a and ESI Fig. S5e†). Aside from TMP^3^-Me_eq_ (C26···C33 4.097(6) Å), one other TMP-Me group showed a *strong* correlation to *o*-Ph, at *δ*(^1^H, ^1^H) = (7.88, 1.99) ppm, which arose from TMP^1^-Me_eq_ (C8···C29 4.357(6) Å). Unlike in **3**, this ligand adopted an *exo* orientation with respect to the aryl-bonded Cu center (in the solid state), leaving TMP^1^-Me_ax_ unsuitably orientated to generate a nOe with the aromatic ring. This is consistent with the failure to observe exchange correlations for TMP^1^ at room temperature (by which means a transient nOe to *o*-Ph might arise from Me_ax_/Me_eq_ interconversion). In contrast, both TMP^2^-Me_eq_ and TMP^2^-Me_ax_ (^1^H NMR, *δ* 1.82 and 1.77 ppm) gave rise to *weak* NOEs to *o*-Ph, suggesting ring inversion of TMP^2^ to bring both Me groups transiently close to *o*-Ph (*viz.* C11···C29 4.853(7) Å). Any potential correlations between TMP^2^-Me_eq_ and TMP^2^-Me_ax_ are obscured by the diagonal. The observation of a greater number of cross-peaks from TMP^2^-Me_eq/ax_ than expected (in fact, to *all* other TMP-Me resonances, and additionally to *o*-Ph; see ESI, Fig. S5e†) could be explained by this phenomenon. On the other hand, the observation of a single correlation, at *δ*(^1^H, ^1^H) = (1.99, 1.21) ppm, between the Me groups of TMP^1^ and TMP^3^ suggested that these amido ligands were not conformationally mobile.

At higher temperatures (80 °C in C_7_D_8_, Fig. 14b), exchange correlations between the Me hydrogens of TMP^1^ are revealed by ^1^H,^1^H-NOESY, at *δ*(^1^H, ^1^H) = (1.96, 1.59) ppm (ESI, Fig. S5f†), suggesting thermally induced ring inversion (*cf.*^1^H,^1^H-NOESY on **3** at room temperature). Interestingly, DFT calculations suggest that the molecular conformer in which TMP^2^ is ring-flipped with respect to the ground-state structure shown in Fig. 13 is the second lowest energy structure, whilst the third lowest in energy is generated by inverting TMP^1^ instead (see ESI, Fig. S12a and S12g†). In other words, spectroscopic data suggest that increasing the temperature experimentally accesses higher energy conformers, and these appear in the order predicted by DFT calculations.

**Fig. 15:**
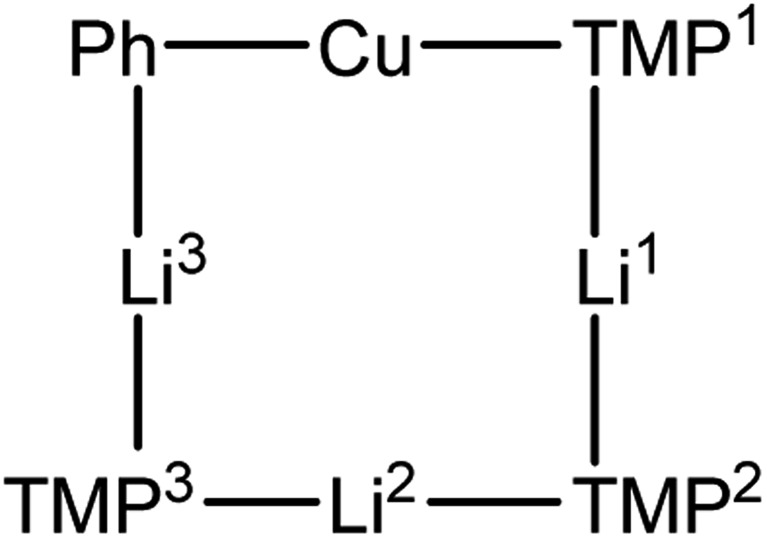
Labelling scheme for **7**.

**Fig. 16:**
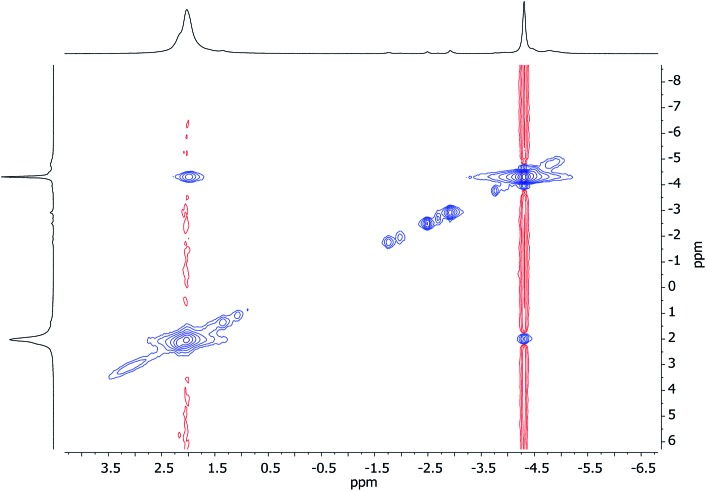
^7^Li,^7^Li-NOESY spectrum of **7** in C_7_D_8_, recorded at 25 °C; exchange correlations in blue (*τ* = 0.05 s). The shoulder at *δ* 2.23 ppm is TMPLi.

Unlike for **3** and **5**, solid-state structural data on **7** could not be obtained. At ambient temperature, ^1^H NMR spectroscopy on **7** revealed broadly similar features to those of **3** and **5**, namely, six TMP-Me resonances. These could therefore reasonably be grouped by comparison with spectra of **3** and **5** (Fig. 15, ESI Fig. S7d†). Additionally, ^1^H,^7^Li-HOESY of **7** revealed a *δ*(^1^H, ^7^Li) = (0.93, –4.25) ppm correlation presumably originating from TMP^3^-Me. However, rapid exchange of Li^1^ and Li^2^ was implied by the observation of not three but of only two ^7^Li resonances (*δ* 2.09 (br), –4.26 ppm) in a 2 : 1 integral ratio. More surprisingly, ^7^Li,^7^Li-NOESY revealed exchange of Li^1^ and Li^2^ centers with phenyl-bound Li^3^ (Fig. 16), implying that, overall, all Li sites are exchangeable in solution.

**Fig. 17:**
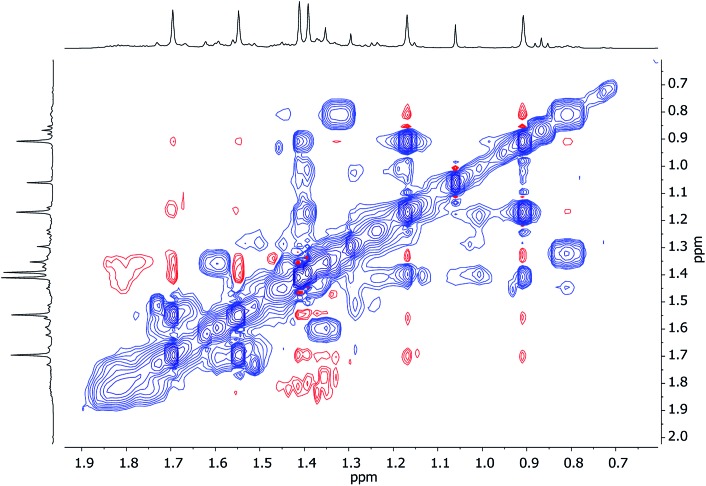
^1^H,^1^H-NOESY spectrum of **7** in C_6_D_6_, at 25 °C; exchange correlations in blue (*τ* = 0.6 s).

**Scheme 3:**
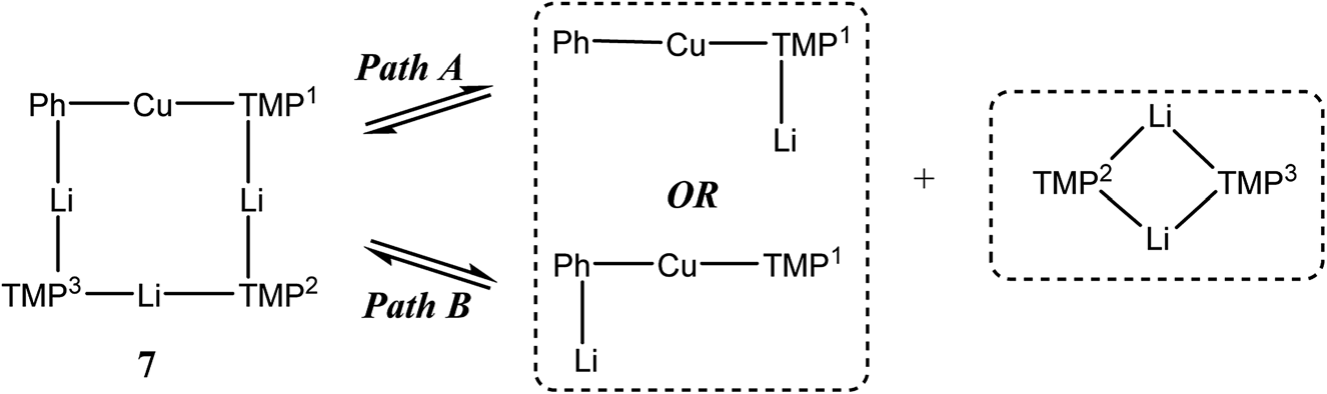
Dissociative interchange of Li sites and amido ligands, mediated by the symmetrical amidolithium dimer (TMPLi)_2_.

^1^H,^1^H-NOESY data reinforce the view that **7** is fluxional by revealing substantial exchange correlations between TMP-Me groups at *δ*(^1^H, ^1^H) = (1.70, 1.55), (1.41, 0.91), (1.39, 1.17) and (1.17, 0.91) ppm (Fig. 17). The first and last pairs of correlations indicate conformational fluxionality associated with each of TMP^1^ and TMP^3^ (*i.e.* ring inversion-type behaviour, as seen in **3** at 25 °C). Whilst it is likely that TMP^2^ also behaves in this way, any potential correlations are obscured by the diagonal. The remaining pairs of correlations indicate that TMP^2^ and TMP^3^ undergo chemical exchange; this necessitates the involvement of a dissociative pathway. Most probably, exchange proceeds *via* a metallacyclic (TMPLi)_2_ intermediate of a type well established in amidolithium chemistry.^46^ Its symmetry would permit exchange of all Li centers and of TMP^2^ and TMP^3^ (but not TMP^1^, see Scheme 3). This thesis also explains the appearance of the ^7^Li NMR spectrum of **7** if it is suggested that the two exchange paths illustrated proceed at different rates, with path B occurring faster and causing the broadness of the singlet at *δ* 2.09 ppm. Upon cooling to –20 °C, ^1^H,^1^H-NOESY fails to show exchange correlations, whilst at –10 °C only those associated with TMP^3^-Me_eq_/TMP^3^-Me_ax_ interconversion are seen (see ESI, Fig. S7g and f†). Clearly therefore, TMP^2^/TMP^3^ exchange is not established at either temperature. Similarly, ^7^Li,^7^Li-NOESY at these temperatures fails to reveal exchange correlations (ESI, Fig. S7j and k†). Taken together, these data suggest that the exchange of inequivalent amido ligands and Li sites in **7** is mediated by a common intermediate, and this lends support to the mechanism proposed in Scheme 3.

## Conclusions

Recently, the importance of Li–Cu interchange has been established in lithium amidocuprates. The relevance of this to their reactivity has now been substantiated through the observation of the dramatically different action of two metal-interchange isomers, metal amide aggregate (TMPCu)_2_(TMPLi)_2_**1** and Gilman lithium cuprate [TMPCu(μ-TMP)Li]_2_**2**_2_. While isolated **2**_2_ proves incapable of metalating benzene, **1** is able to do so smoothly under mild conditions, yielding predominately Ph(TMP)_3_Cu_2_Li_2_**3**, a novel mixed organo(amido)cuprate aggregate. This reactivity is not limited to preformed **1**: simple mixtures of TMPLi and TMPCu also exhibit this behaviour in benzene solution, and in so doing offer both ease of handling and greater possibilities in terms of the products accessible. Critically, the failure of either monometallic amide to efficiently metalate benzene confirms that ring metalation has a synergistic origin. Manipulating the reactant Cu : Li stoichiometry when combining monometallic amides leads to the additional formation of either Ph(TMP)_3_Cu_3_Li **5** or Ph(TMP)_3_CuLi_3_**7**, whose presence dominates in Cu- or Li-rich reaction mixtures, respectively. Additionally, the formation of **5** is accompanied by that of minor amounts of Ph(TMP)_3_Cu_4_**6**, which is believed to result from Cu–Li exchange between **5** and TMPCu. In the reaction pathway that leads to **3**, **5** and **7,** the generation of putative PhCu(TMP)Li **4** from reaction of a base of formula (TMP)_2_CuLi with benzene is suggested. The absence of **4** from the final reaction mixtures is explained by its rapid *in situ* combination with metal amides. This notion is borne out by the observation that pre-isolated **4** equilibrates with **2**, TMPCu or TMPLi to give **3**, **5** and **7**, and that these can subsequently be isolated independently of one another. Both solid- (**3–5**) and solution-state (**3–5**, **7**) measurements expose Li···π interactions as a prominent structural feature and these interactions are reproduced satisfactorily by DFT calculations. Additionally, DFT analysis predicts the ground-state molecular conformations of **3–5**, each of which was also exposed by crystallography. The relatively small differences in energy associated with the molecular conformers of each lead ostensibly to the emergence of exchange correlations in ^1^H,^1^H-NOESY experiments, while Li-rich **7** exhibits additional exchange that is suggested by ^7^Li,^7^Li-NOESY and requires the dissociation of a lithium amide moiety.

These results expose significant new possibilities for functionalising weakly acidic aromatic hydrocarbons using easy-to-handle and readily accessible lithium and copper amide reagents. Investigations are currently underway to assess the suitability of different solvents for handling and applying **1** and the substrate scope of the deprotonation reaction, particularly as applied to hydrocarbons such as toluene and naphthalene, which could offer more complex regioselectivity. Meanwhile, further DFT studies have been initiated to probe the reaction pathway with a view to explaining the substantially different behaviour of **1** and **2**_2_ and to shed light on reaction intermediates accessible from both **1** and mixtures of TMPLi and TMPCu.

## Experimental section

### General synthetic and analytical details

Reactions were carried out under dry nitrogen, using double manifold and glove-box methods. Solvents were distilled off sodium (toluene) or a sodium-potassium amalgam (THF, Et_2_O, and hexane) immediately before use. 2,2,6,6-tetramethylpiperidine (TMPH) was purchased from Alfa Aesar and stored over molecular sieves (4 Å). Other chemicals were used as received. Copper(i) chloride and *^n^*BuLi (1.6 M in hexane) were purchased from Acros and used as received. The syntheses of TMPCu(μ-TMP)Li **2** (ref. 27) and ^*t*^BuOCu^47^ were based on the literature. NMR data were collected on a Bruker Avance III HD 500 MHz Smart Probe FT NMR spectrometer (500.200 MHz for ^1^H, 125.775 MHz for ^13^C, and 194.397 for ^7^Li). Spectra were obtained at 25 °C (unless otherwise stated) using deuterated solvent stored over molecular sieves (3 Å). For ^1^H and ^13^C, chemical shifts were internally referenced to deuterated solvent and calculated relative to TMS. For ^7^Li, an external reference was used (1 M LiCl in D_2_O). Chemical shifts are expressed in *δ* ppm. The following abbreviations are used: br = broad, m = multiplet, s = singlet, sh = shoulder.

### Crystallographic details

For details of data collections see Table 1. Crystals were transferred from the mother liquor to a drop of perfluoropolyether oil mounted upon a microscope slide under cold nitrogen gas.^48^ Suitable crystals were attached to the goniometer head *via* a MicroLoop™, which was then centred on the diffractometer. Data were collected on a Bruker D8 Quest (Cu-Kα, *λ* = 1.54184 Å) or a Nonius Kappa CCD diffractometer (Mo-Kα, *λ* = 0.71073 Å), each equipped with an Oxford Cryosystems low-temperature device (*T* = 180(2) K). Structures were solved using SHELXT,^49^ and refinement (based on *F*^2^, by the full-matrix least squares method) was performed using SHELXL.^50^ Non-hydrogen atoms were refined anisotropically (for disorder, standard restraints and constraints were employed as appropriate) and a riding model with idealized geometry was employed for the refinement of H-atoms. Data have been deposited with the Cambridge Crystallographic Data Centre as supplementary publications CCDC ; 1869336–1869340.†

**Table 1:** Crystallographic data

	**1**	**4**_2_	**3**	**5**	**6**
Formula	C_36_H_72_Cu_2.01_Li_1.99_N_4_	C_30_H_46_Cu_2_Li_2_N_2_	C_33_H_59_Cu_2_Li_2_N_3_	C_33_H_59_Cu_3.12_Li_0.88_ N_3_	C_33_H_59_Cu_4_N_3_
*M*	702.64	575.65	638.79	702.39	751.99
Crystal system	Monoclinic	Orthorhombic	Monoclinic	Orthorhombic	Orthorhombic
Space group	*P*2_1_/*c*	*Pbca*	*P*2_1_/*n*	*Pbca*	*Pca*2_1_
*a* (Å)	11.679(2)	8.6647(2)	15.4346(5)	11.3882(2)	11.2175(4)
*b* (Å)	22.808(5)	16.1964(4)	12.0863(4)	18.8747(4)	15.9250(6)
*c* (Å)	15.234(3)	21.7364(6)	19.5308(6)	31.9081(7)	18.9824(7)
*α* (°)	90	90	90	90	90
*β* (°)	108.90(3)	90	110.2960(10)	90	90
*γ* (°)	90	90	90	90	90
*V* (Å^3^)	3839.1(15)	3050.42(13)	3417.21(19)	6858.7(2)	3391.0(2)
*Z*	4	4	4	8	4
*ρ*_calcd_ (Mg m^–3^)	1.216	1.253	1.242	1.360	1.473
*λ* (Å)	0.71073	1.54184	1.54184	1.54184	1.54184
*μ* (mm^–1^)	1.143	1.839	1.694	2.405	2.997
Data	32 751	20 457	28 978	38 994	23 738
Unique data	7474	2678	6023	6044	5601
*R*_int_	0.0384	0.0366	0.0406	0.0415	0.0252
*θ* (°)	3.778–26.017	4.064–66.424	3.167–66.751	2.770–66.634	2.775–66.611
*wR*_2_	0.1051	0.0806	0.0870	0.1063	0.0715
*R*	0.0478	0.0326	0.0319	0.0491	0.0234
GoF	1.242	1.062	1.013	1.280	1.146
Flack parameter	n/a	n/a	n/a	n/a	–0.03(2)
Parameters	417	167	373	374	373
Peak/hole (eÅ^–3^)	0.405/–0.273	0.258/–0.330	0.368/–0.434	0.404/–0.380	0.306/–0.377

### Synthesis and characterisation of **1**

To a stirred solution of TMPH (0.34 mL, 2 mmol) and Et_2_O (0.21 mL, 2 mmol) in toluene (2 mL) at –78 °C was added ^*n*^BuLi (1.6 M in hexanes, 1.25 mL, 2 mmol). The solution was warmed to room temperature and then added dropwise to a suspension of CuCl (0.1 g, 1 mmol) in toluene (1 mL) at –78 °C. The mixture was warmed to room temperature whereupon a grey discolouration occurred. LiCl was removed by filtration to give a yellow solution, the storage of which at 5 °C for 48 h yielded a small crop of colourless block-like crystals. Yield 50 mg (14%). Melting point 187–189 °C. Elemental analysis: C_18_H_36_CuLiN_2_ requires (%) C, 61.60; H, 10.34; N, 7.98. Found (%) C, 60.86; H, 10.49; N, 7.85. ^1^H NMR (500 MHz, 298 K, C_6_D_6_): *δ* 1.89–1.78 (m, 4H, TMP-4), 1.76 (s, 12H, TMP-Me), 1.74–1.69 (m, 2H, TMP-4), 1.66 (m, 4H, TMP-3,5), 1.59 (m, 4H, TMP-3,5), 1.57 (s, 24H, TMP-Me), 1.53 (m, 2H, TMP-4), 1.39 (s, 12H, TMP-Me), 1.37 (m, 4H, TMP-3,5), 1.08 (m, 4H, TMP-3,5), 1.06 (s, 2H, TMPH-Me). ^13^C NMR (125 MHz, 298 K, C_6_D_6_): *δ* 56.9 (TMP-2,6), 54.2 (TMP-2,6), 52.0 (TMP-2,6), 42.6 (TMP-3,5), 42.5 (TMP-3,5), 42.1 (TMP-3,5), 39.7 (TMP-Me), 37.0 (br, TMP-Me), 36.8 (TMP-Me), 34.2 (TMP-Me), 19.6 (TMP-4), 19.3 (TMP-4), 19.1 (TMP-4). ^7^Li NMR (194 MHz, 298 K, C_6_D_6_): *δ* 1.64 (s, 2Li), 0.96 (s, 0.3Li).

### Synthesis and characterisation of **3**

PhCu(TMP)Li **4** (290 mg, 1 mmol) and (TMP)_2_CuLi **2** (350 mg, 1 mmol) were combined in toluene (6 mL) and heated to 50 °C for *ca.* 24 h. The toluene was removed *in vacuo*, and the residue was dissolved in hexane (*ca.* 5 mL). This was filtered and the resulting colourless solution was stored at 5 °C for 24 h, after which time block-like crystals formed. Yield 126 mg (20%). Melting point 151–153 °C. Elemental analysis: C_33_H_59_Cu_2_Li_2_N_3_ requires (%): C, 62.05; H, 9.31; N, 6.58. Found (%): 62.14; H, 9.35; N, 6.62. ^1^H NMR spectroscopy (500 MHz, 298 K, C_6_D_6_): *δ* 7.85 (d, *J* = 7 Hz, 2H, *o*-Ph), 7.01 (t, *J* = 7 Hz, 2H, *m*-Ph), 6.93 (t, *J* = 7 Hz, 1H, *p*-Ph), 1.78 (m, 2H, TMP-4), 1.73 (s, 6H, TMP-Me), 1.69 (m, 2H, TMP-3,5), 1.63 (s, 6H, TMP-Me), 1.60 (m, 2H, TMP-4), 1.56 (s, 6H, TMP-Me), 1.54 (m, 2H, TMP-3,5), 1.51 (s, 6H, TMP-Me), 1.46 (m, 4H, TMP-3,5), 1.41 (s, 6H, TMP-Me), 1.36 (m, 2H, TMP-4), 1.17 (m, 2H, TMP-3,5), 1.15 (s, 6H, TMP-Me), 0.45 (m, 2H, TMP-3,5). ^13^C NMR spectroscopy (125 MHz, 298 K, C_6_D_6_): *δ* 164.2 (*i*-Ph), 140.8 (*o*-Ph), 128.9 (*m*-Ph), 125.5 (*p*-Ph), 54.1 (TMP-2,6), 54.0 (TMP-2,6), 53.0 (TMP-2,6), 42.5 (TMP-3,5), 41.9 (TMP-3,5), 41.1 (TMP-3,5), 39.5 (TMP-Me), 39.3 (TMP-Me), 38.2 (TMP-Me), 35.8 (TMP-Me), 34.1 (TMP-Me), 33.3 (TMP-Me), 19.6 (TMP-4), 19.2 (TMP-4), 18.8 (TMP-4). ^7^Li NMR spectroscopy (194 MHz, 298 K, C_6_D_6_): *δ* 1.41 (s, 1Li), –2.86 (s, 1Li).

### Synthesis and characterisation of **4**

To a stirred solution of TMPH (0.68 mL, 4 mmol) in hexane/THF (8 mL/8 mL) was added ^*n*^BuLi (1.6 M in hexanes, 2.5 mL, 4 mmol) at –20 °C. The pale-yellow solution was warmed to room temperature and transferred to a stirred suspension of CuCl (0.4 g, 4 mmol) in hexane/THF (4 mL/4 mL) at –20 °C. The mixture was warmed to room temperature and stirred for 15 min, whereupon a thick, cream-coloured suspension of TMPCu was formed. The solvents were removed *in vacuo* and the residue was treated with toluene (24 mL). Hot filtration of the resulting suspension to remove LiCl gave a tan coloured solution, which precipitated upon standing. PhLi was prepared by dropwise treatment of a solution of PhI (0.44 mL, 4 mmol) in hexane (6 mL) with ^*n*^BuLi (1.6 M in hexanes, 2.5 mL, 4 mmol) at room temperature. The solvent and BuI were removed *in vacuo* to leave a white powder. TMPCu was re-dissolved by gentle heating and transferred to the residue of PhLi at room temperature, whereupon the PhLi dissolved to give a bright yellow solution. This was concentrated (*ca.* 1/2 vol.), filtered and stored at 5 °C for 24 h to give a crop of colourless prismatic crystals. Yield 260 mg (23%). Melting point 195–197 °C (dec.). Elemental analysis: C_15_H_23_CuLiN requires (%): C, 62.59; H, 8.05; N, 4.87. Found (%) C, 62.38; H, 7.98; N, 4.86. ^1^H NMR spectroscopy (500 MHz, 298 K, C_6_D_6_): *δ* 7.87 (d, *J* = 7 Hz, 2H, *o*-Ph), 7.12 (t, *J* = 7 Hz, 2H, *m*-Ph), 6.97 (t, *J* = 7 Hz, 1H, *p*-Ph), 1.86–1.52 (m, 2H, TMP-4), 1.49 (m, 2H, TMP-3,5), 1.45 (s, 6H, TMP-Me), 1.24 (s, 6H, TMP-Me), 0.58 (m, 2H, TMP-3,5). ^13^C NMR spectroscopy (125 MHz, 298 K, C_6_D_6_): *δ* 164.0 (*i*-Ph), 140.7 (*o*-Ph), 128.8 (*m*-Ph), 125.7 (*p*-Ph), 52.7 (TMP-2,6), 40.7 (TMP-3,5), 38.2 (TMP-Me), 34.9 (TMP-Me), 19.2 (TMP-4). ^7^Li NMR spectroscopy (194 MHz, 298 K, C_6_D_6_): *δ* –0.58 (s, 0.04Li), –2.43 (s, 1Li), –2.63 (s, 0.1Li).

### Synthesis and characterisation of **5**

PhCu(TMP)Li **4** (145 mg, 0.5 mmol) and TMPCu (200 mg, 1 mmol) were combined in toluene (6 mL) and heated to 50 °C for *ca.* 12 h. The colourless solution was filtered and concentrated until precipitation occurred, at which point hexane (*ca.* 1 mL) was added. The solid was dissolved with gentle heating and allowed to cool to room temperature, whereupon colourless crystals were formed. Yield 161 mg (46%). Melting point 189–191 °C. Elemental analysis: C_33_H_59_Cu_3_LiN_3_ requires (%): C, 57.00; H, 8.55; N, 6.04. Found (%): C, 56.21; H, 8.75; N, 6.00. ^1^H NMR spectroscopy (500 MHz, 298 K, C_6_D_6_): *δ* 7.88 (d, *J* = 7 Hz, 2H, *o*-Ph), 7.15 (t, *J* = 7 Hz, 2H, *m*-Ph), 7.01 (t, *J* = 7 Hz, 1H, *p*-Ph), 1.99 (s, 6H, TMP-Me), 1.82 (s, 6H, TMP-Me), 1.77 (s, 6H, TMP-Me), 1.75–1.70 (m, 4H, TMP-4), 1.68–1.60 (m, 6H, TMP-3,5), 1.59 (s, 6H, TMP-Me), 1.46 (m, 4H, TMP-3,5), 1.40 (m, 6H, TMP-Me), 1.38 (m, 2H, TMP-4), 1.21 (s, 6H, TMP-Me), 0.48 (m, 2H, TMP-3,5). ^13^C NMR spectroscopy (125 MHz, 298 K, C_6_D_6_): *δ* 162.0 (*i*-Ph), 140.8 (*o*-Ph), 129.1 (*m*-Ph), 126.1 (*p*-Ph), 56.9 (TMP-2,6), 56.4 (TMP-2,6), 53.2 (TMP-2,6), 43.2 (TMP-3,5), 42.5 (TMP-3,5), 41.3 (TMP-3,5), 40.5 (TMP-Me), 39.1 (TMP-Me), 36.9 (br, TMP-Me), 36.7 (br, TMP-Me), 33.3 (TMP-Me), 32.5 (TMP-Me), 19.3 (TMP-4), 19.1 (TMP-4), 18.7 (TMP-4). ^7^Li NMR spectroscopy (194 MHz, 298 K, C_6_D_6_): *δ* –1.46 ppm.

### Synthesis and characterisation of **6**

Ph(TMP)_3_Cu_3_Li **5** (0.5 mmol) was prepared as described above in toluene (6 mL). ^*t*^BuOCu (70 mg, 0.5 mmol) was added and the solution was stirred at 50 °C for 12 h, during which time a green colouration developed. The solvent was evaporated *in vacuo* and the residue was treated with hexane (6 mL). The green supernatant was decanted and the white residue was re-dissolved in toluene (*ca.* 1 mL). Storage at 5 °C for 24 h yielded a crop of block-like crystals. Yield 25 mg (7%). Melting point 230 °C (dec.). Elemental analysis: C_33_H_59_CuLi_3_N_3_ requires (%): C, 52.71; H, 7.91; N, 5.59. Found (%): C, 53.01; H, 8.04; N, 5.77. ^1^H NMR spectroscopy (500 MHz, 298 K, C_6_D_6_): *δ* 7.92 (d, *J* = 7 Hz, 2H, *o*-Ph), 7.20–7.10 (m, 3H, *m*-Ph + *p*-Ph), 1.87 (s, 12H, TMP-Me), 1.85 (s, 12H, TMP-Me), 1.72 (m, 2H, TMP-4), 1.65 (m, 2H, TMP-4), 1.58 (m, 4H, TMP-3,5), 1.52 (s, 12H, TMP-Me), 1.46 (m, 4H, TMP-3,5), 1.31 (m, 2H, TMP-4), 1.01 (m, 4H, TMP-3,5). ^13^C NMR spectroscopy (125 MHz, 298 K, C_6_D_6_): *δ* 145.0 (*o*-Ph), 137.4 (*i*-Ph), 129.8 (*p*-Ph), 127.1 (*m*-Ph), 57.1 (TMP-2,6), 56.3 (TMP-2,6), 42.6 (TMP-3,5), 42.4 (TMP-3,5), 40.5 (TMP-Me), 37.1 (TMP-Me), 32.6 (TMP-Me), 19.3 (TMP-4), 18.8 (TMP-4). For the ordering of ^13^C NMR spectroscopic resonances in arylcopper species see ref. 51.

### Synthesis and characterisation of **7**

PhCu(TMP)Li (143 mg, 0.5 mmol) and TMPLi (147 mg, 1 mmol) were combined in hexane (6 mL) and stirred for 48 h at room temperature. The solvent was removed *in vacuo* to give a white microcrystalline solid, which was used without further purification. Yield 140 mg (48%). Melting point 146–148 °C. Elemental analysis: C_33_H_59_CuLi_3_N_3_ requires (%): C, 68.08; H, 10.21; N, 7.22. Found (%): C, 67.45; H, 9.79; N, 7.44. ^1^H NMR spectroscopy (500 MHz, 298 K, C_6_D_6_): *δ* 7.84 (d, *J* = 7 Hz, 2H, *o*-Ph), 6.98 (t, *J* = 7 Hz, 2H, *m*-Ph), 6.80 (m, 1H, *p*-Ph), 1.88–1.71 (m, 4H, TMP-4), 1.39 (s, 6H, TMP-Me), 1.64–1.58 (m, 4H, TMP-3,5 + TMP-4), 1.55 (s, 6H, TMP-Me), 1.49–1.43 (m, 4H, TMP-3,5), 1.41 (s, 6H, TMP-Me), 1.39 (s, 6H, TMP-Me), 1.38 (m, 2H, TMP-3,5), 1.35–1.32 (m, 2H, TMP-3,5), 1.17 (s, 6H, TMP-Me), 0.91 (s, 6H, TMP-Me), 0.81 (m, 2H, TMP-3,5). ^13^C NMR spectroscopy (125 MHz, 298 K, C_6_D_6_): *δ* 167.8 (*i*-Ph), 141.3 (*o*-Ph), 127.9 (*m*-Ph), 124.1 (*p*-Ph), 53.9 (TMP-2,6), 51.9 (TMP-2,6), 50.9 (TMP-2,6), 42.2 (TMP-3,5), 42.0 (TMP-3,5), 41.4 (TMP-3,5), 37.3 (TMP-Me), 37.0 (TMP-Me), 36.7 (TMP-Me), 36.1 (TMP-Me), 35.1 (TMP-Me), 19.7 (TMP-4), 19.6 (TMP-4), 19.5 (TMP-4). ^7^Li NMR spectroscopy (194 MHz, 298 K, C_6_D_6_): *δ* 2.09 (s, 2Li), –4.26 (s, 1Li).

## Conflicts of interest

There are no conflicts to declare.

## Supplementary Material

Supplementary information

Crystal structure data
