# Supplementary material for: A new route for the efficient metalation of unfunctionalized aromatics
Source: Chem Sci. 2019 Feb 5;10(11):3385–400. doi: 10.1039/c8sc04325f (PMC6429619; doi:10.1039/c8sc04325f)
Supplement: Supplementary file 1 [file SC-010-C8SC04325F-s001.pdf]

Electronic Supplementary Material (ESI) for Chemical Science.
This journal is © The Royal Society of Chemistry 2019

Electronic Supplementary Information

A new route to the efficient metalation of unfunctionalized aromatics

Andrew J. Peel,[a] Noriyuki Tezuka,[b,c] James M. D'Rozario,[a] Masanobu Uchiyama*[b,c] and Andrew E. H. Wheatley*[a]

[a]*Department of Chemistry, University of Cambridge, Lensfield Road, Cambridge, CB2 1EW (UK);*

Fax: (+) 44 1223 336362; e-mail: aehw2@cam.ac.uk

[b]*Cluster of Pioneering Research (CPR), Advanced Elements Chemistry Laboratory, RIKEN, 2-1 Hirosawa, Wako-shi, Saitama 351-0198 (Japan)*

[c]*Graduate School of Pharmaceutical Sciences, The University of Tokyo, 7-3-1 Hongo, Bunkyo-ku, Tokyo 113-0033 (Japan); e-mail: uchiyama@mol.f.u-tokyo.ac.jp*

Additional characterisation of 1

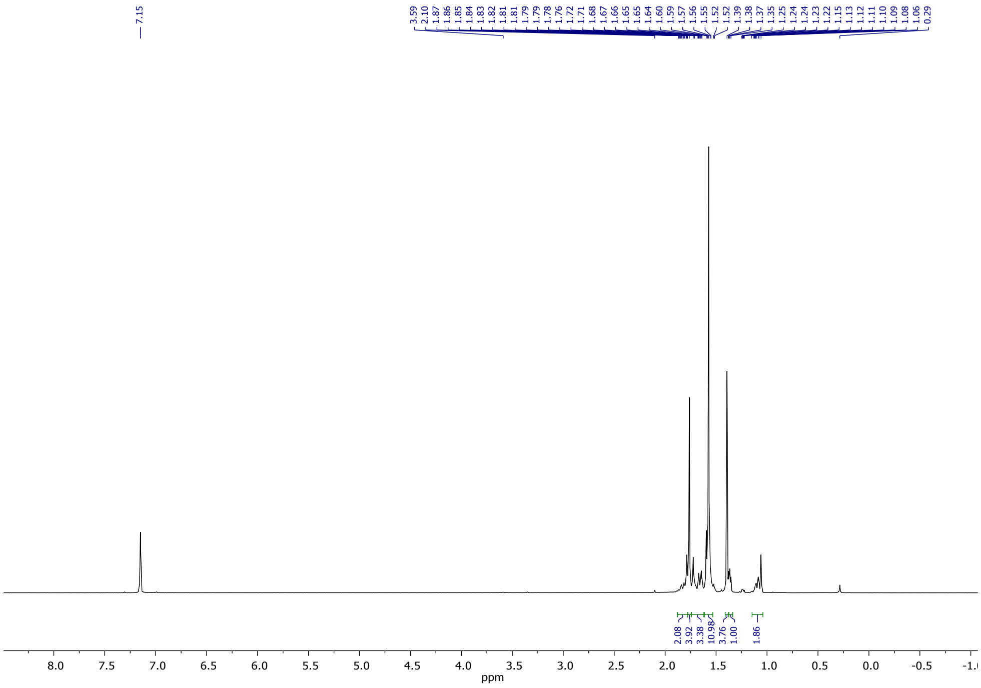

Figure S1a ^{1}H NMR spectrum of **1** (500 MHz, 298 K, C_6D_6).

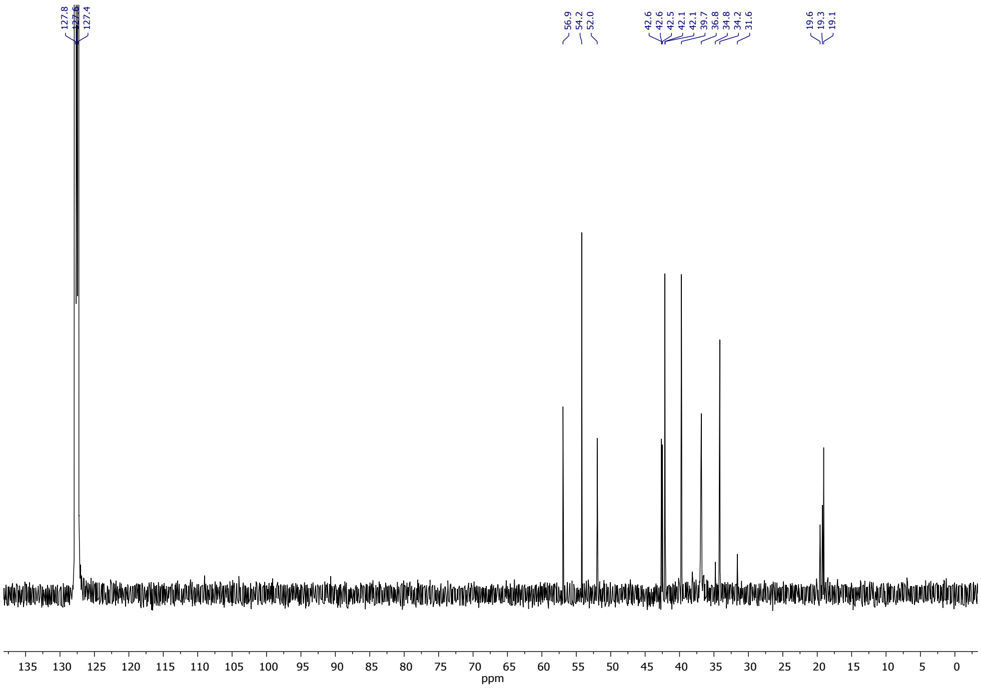

Figure S1b ^{13}C NMR spectrum of **1** (125 MHz, 298 K, C_6D_6).

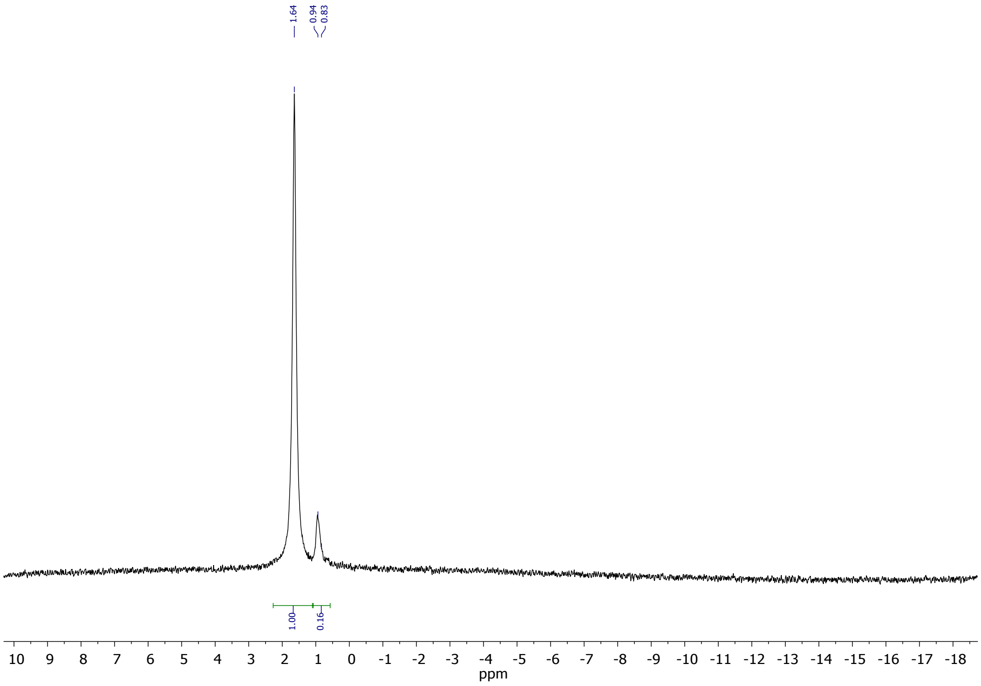

Figure S1c ^{7}Li NMR spectrum of **1** (194 MHz, 298 K, C_6D_6).

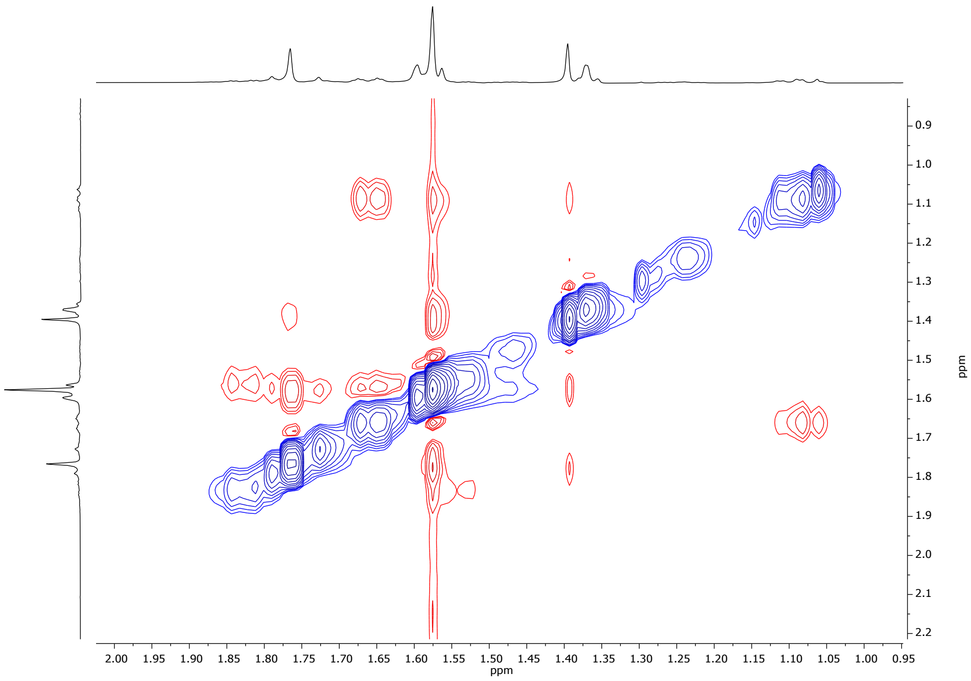

Figure S1d ^{1}H,^{1}H-NOESY spectrum of **1** (500 MHz, 298 K, C_6D_6, τ = 0.6 s).

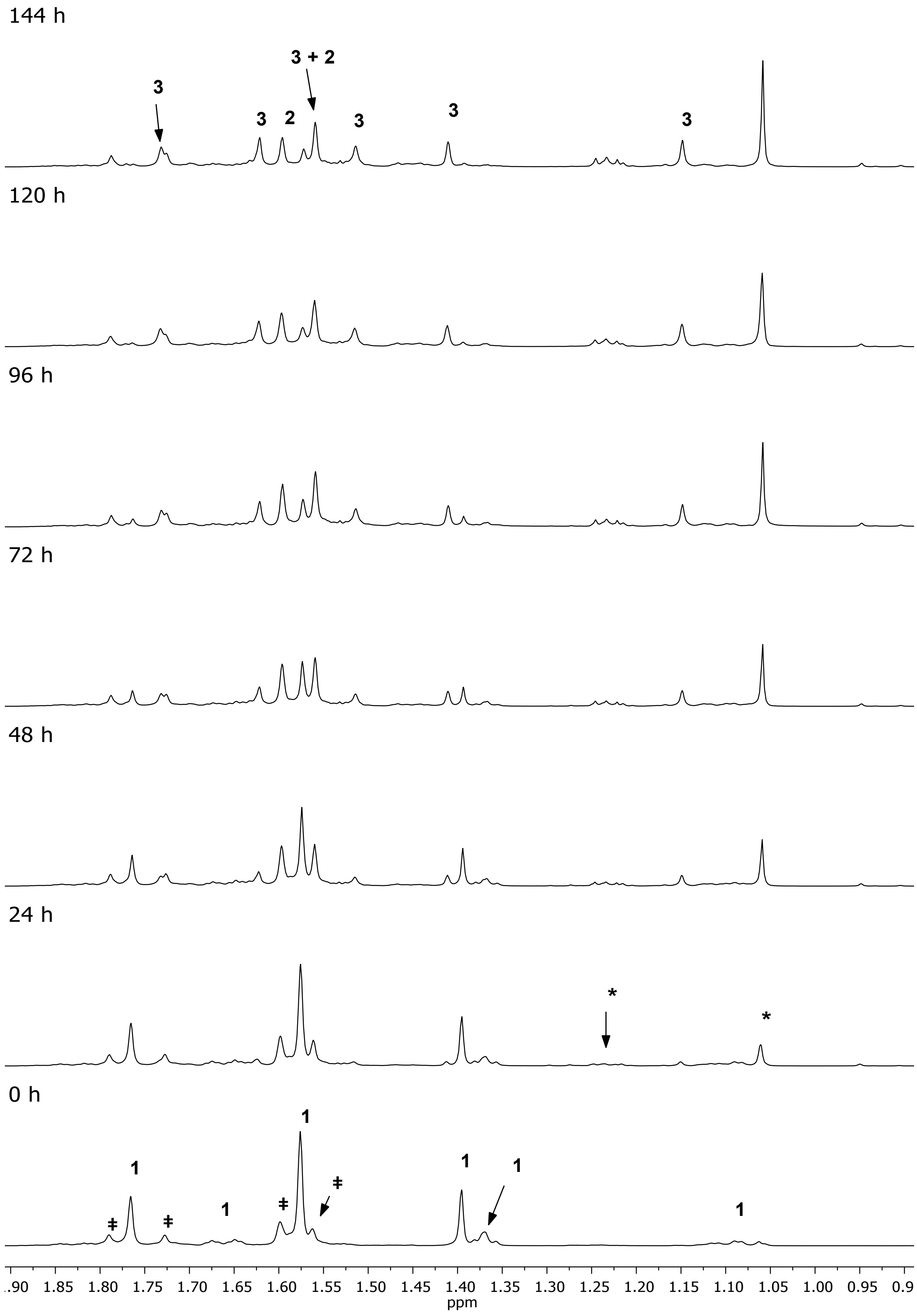

Figure S1e ^{1}H NMR spectra (500 MHz, 298 K, C_6D_6) of **1** heated to 50°C for the time specified. * = TMPH, ‡ = Cu-rich TMP-aggregate.

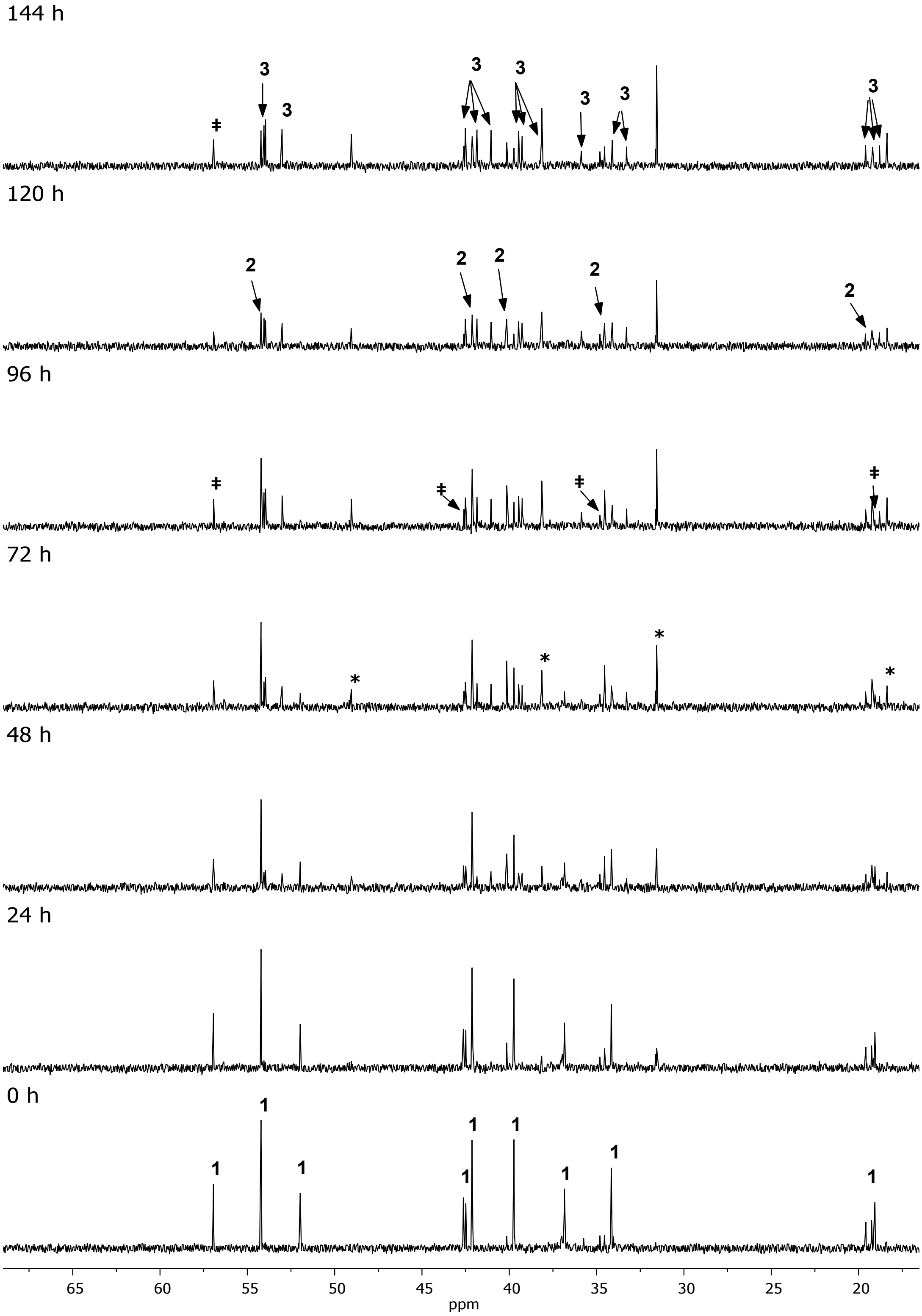

Figure S1f ^{13}C NMR spectra (125 MHz, 298 K, C_6D_6) of **1** heated to 50°C for the time specified. * = TMPH, ‡ = Cu-rich TMP-aggregate.

Additional characterisation of 2

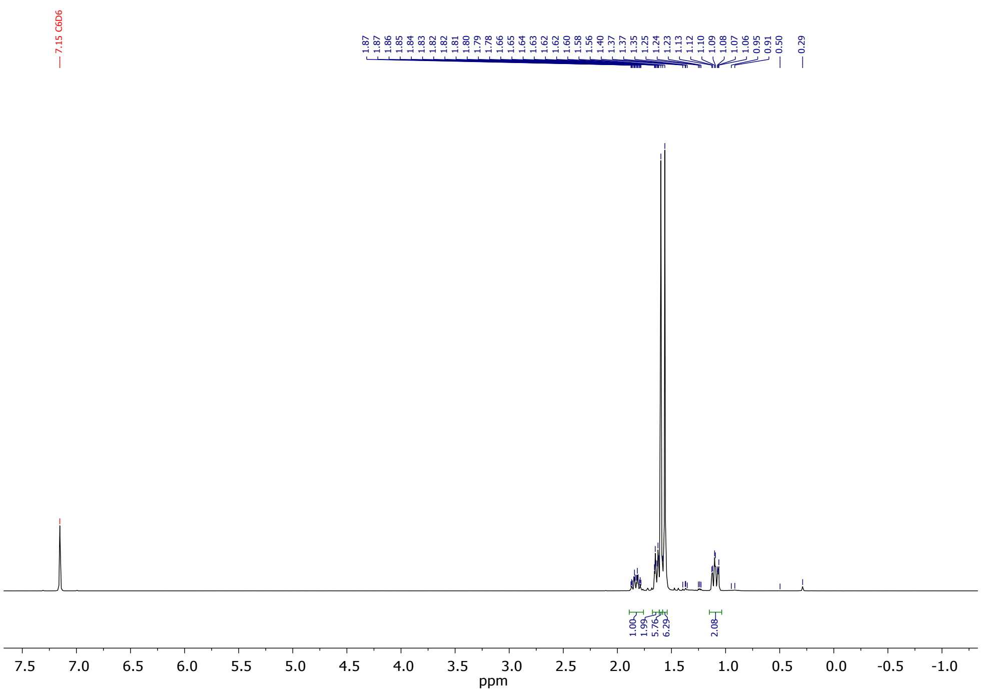

Figure S2a ^{1}H NMR spectrum of **2** (500 MHz, 298 K, C_6D_6).

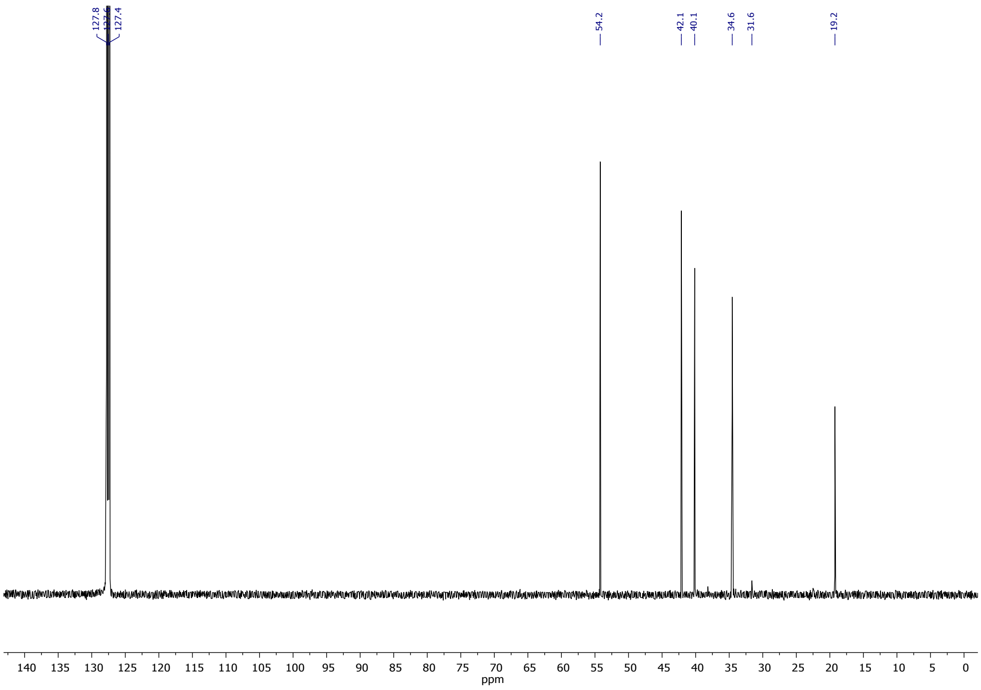

Figure S2b ^{13}C NMR spectrum of **2** (125 MHz, 298 K, C_6D_6).

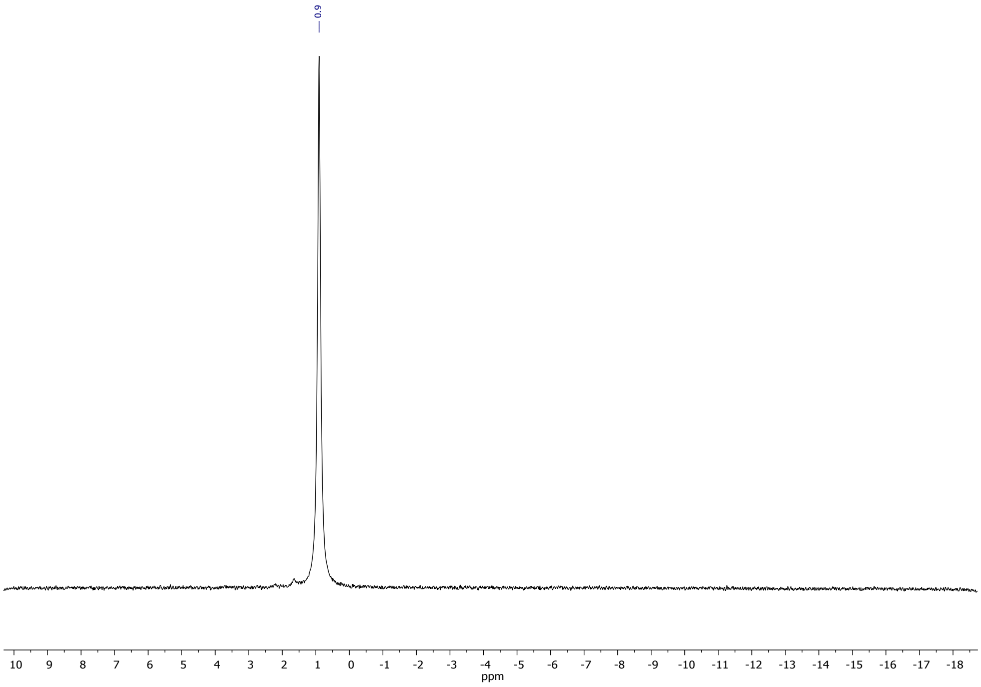

S2c ^{7}Li NMR spectrum of **2** (194 MHz, 298 K, C_6D_6).

Additional characterisation of 3

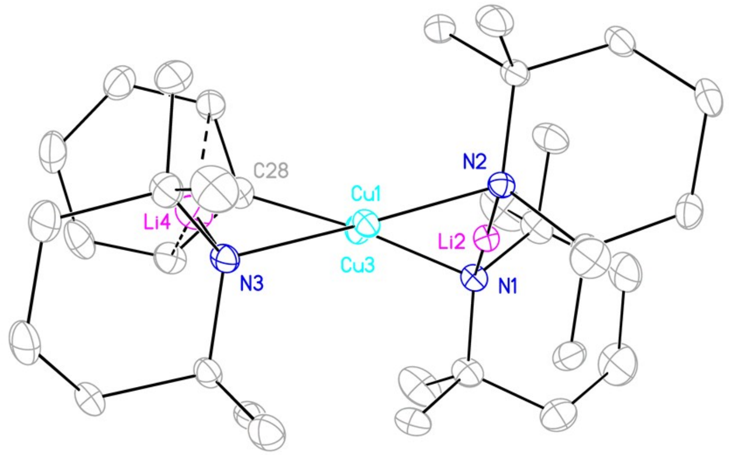

Figure S3a Side-view of **3**, showing deviation of the core metallacycle from planarity.

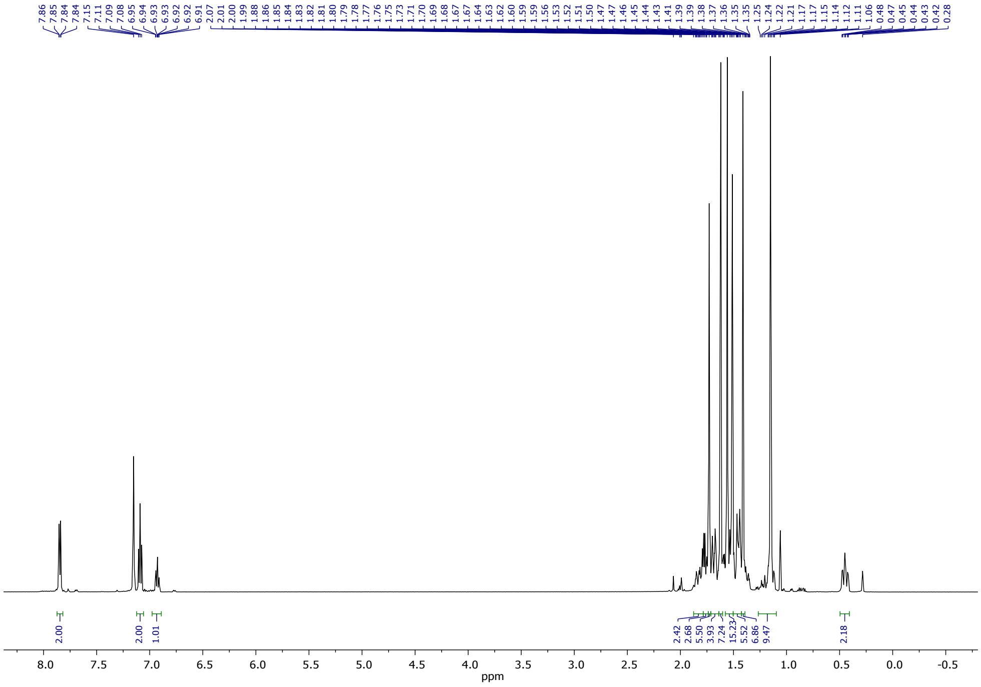

Figure S3b ^{1}H NMR spectrum of **3** (500 MHz, 298 K, C_6D_6).

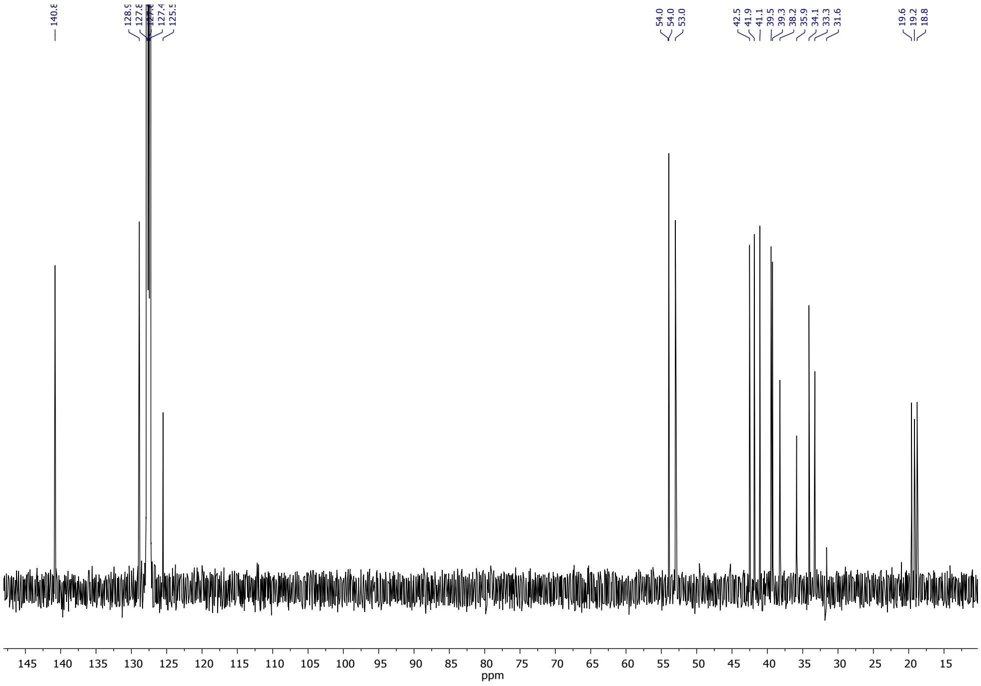

Figure S3c ^{13}C NMR spectrum of **3** (125 MHz, 298 K, C_6D_6).

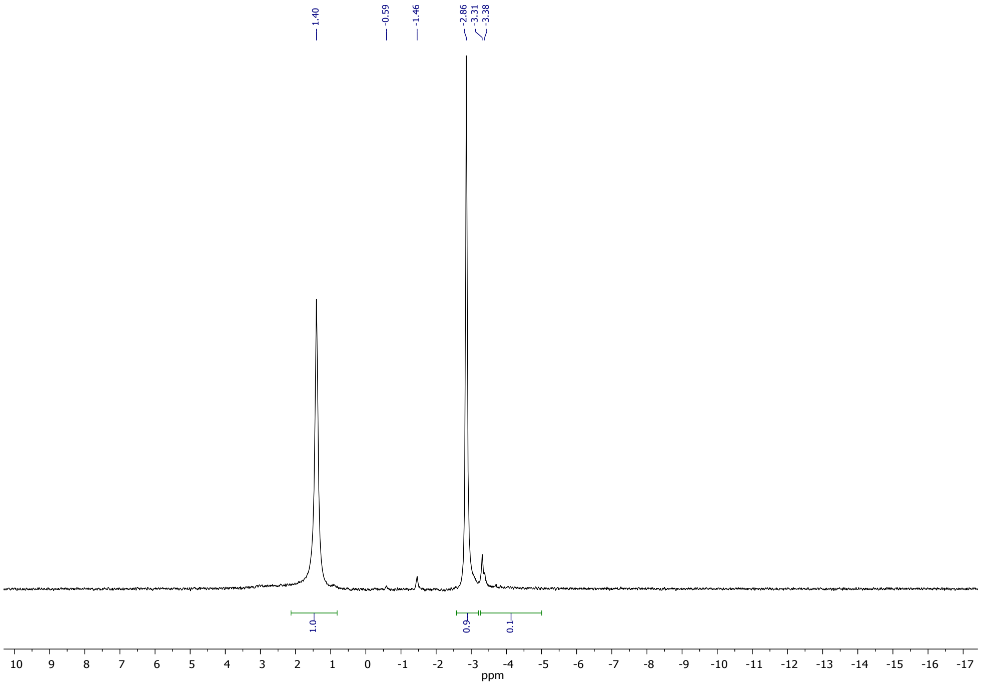

Figure S3d ^{7}Li NMR spectrum of **3** (194 MHz, 298 K, C_6D_6).

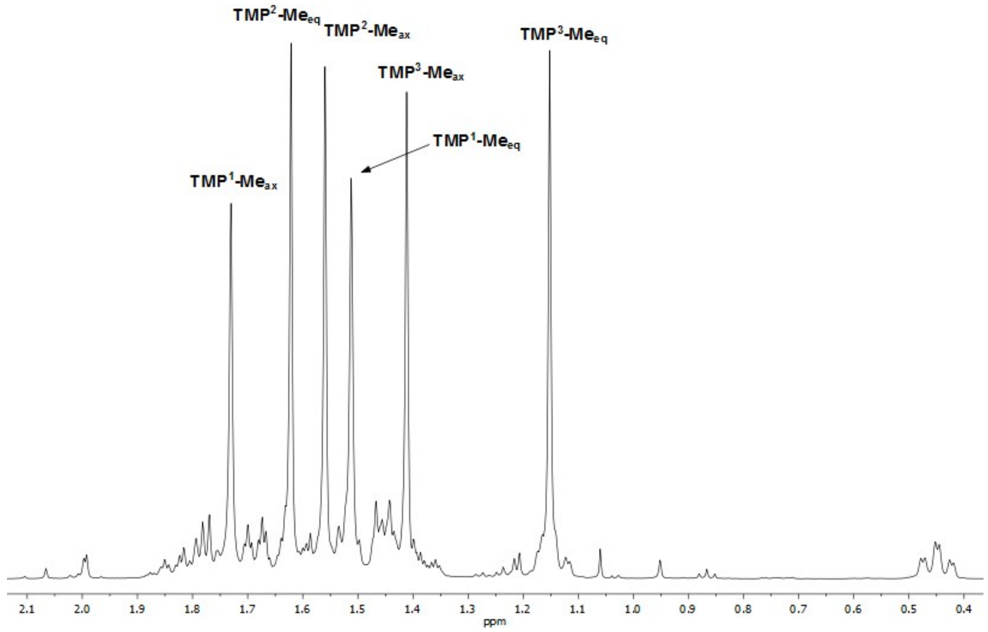

Figure S3e ^{1}H NMR spectra of **3** in C_6D_6 (at 298 K); expansions of aliphatic with TMP-Me groups annotated.

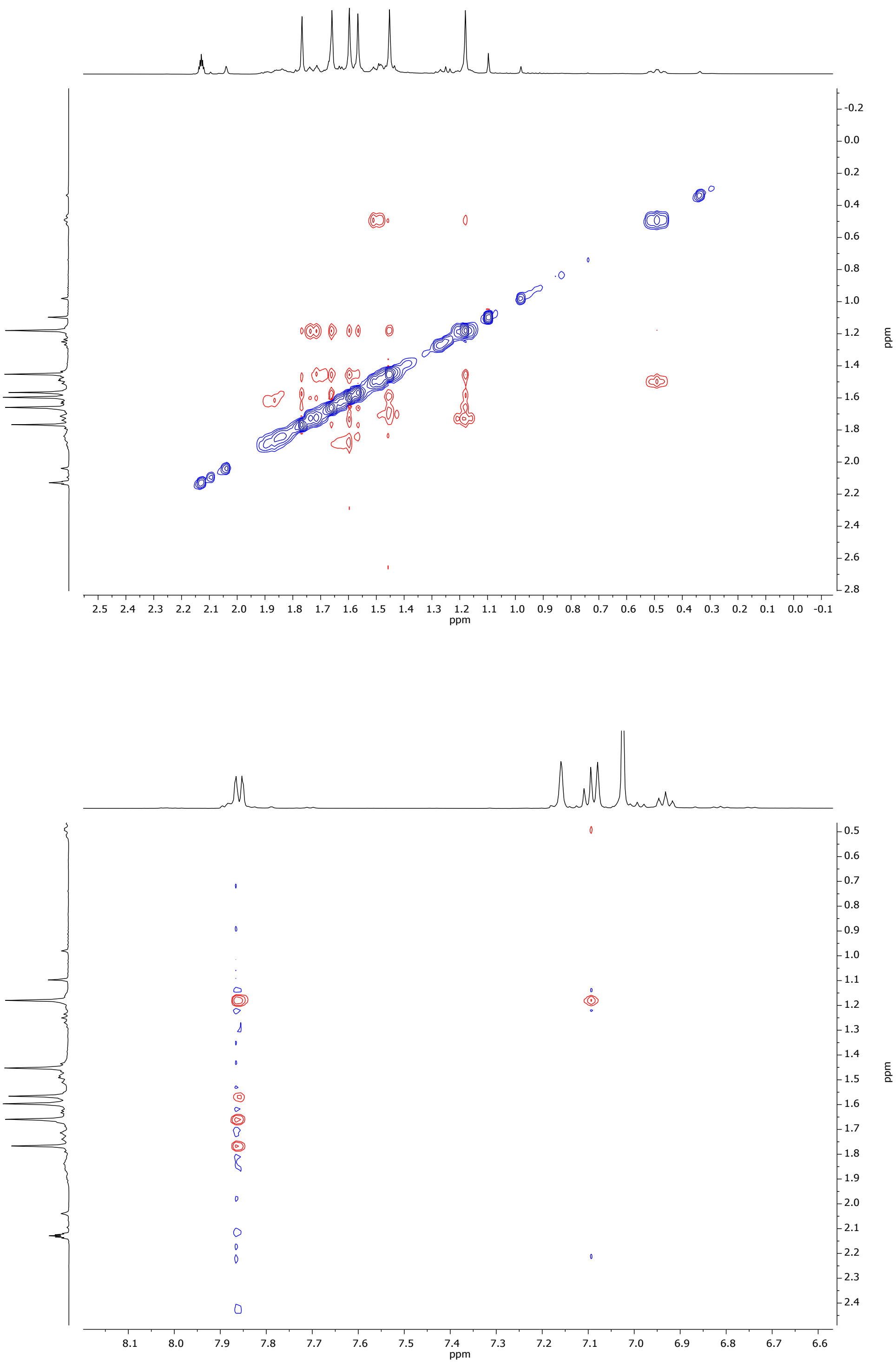

Figure S3f $^{1}H,^{1}H$-NOESY spectra of **3** (500 MHz, 263 K, C_7D_8, $\tau = 0.6$ s)**.** The observation of NOEs to the *o*-Ph hydrogens from both axial and equatorial Me-groups of TMP^1 but only the equatorial groups of TMP^2 and TMP^3 is consistent with TMP^1 lying *endo*.

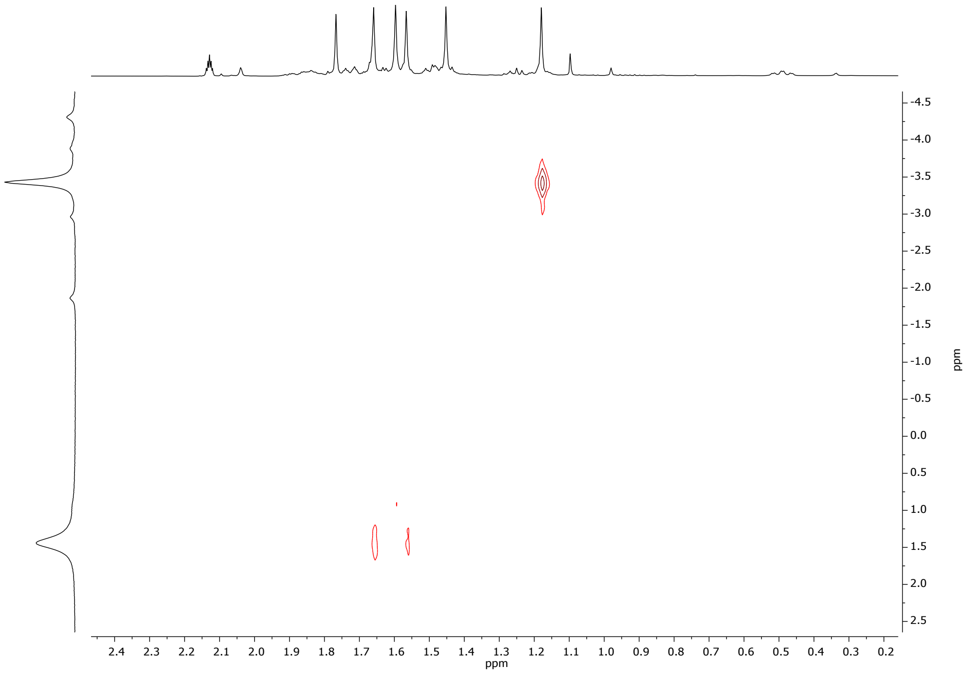

Figure S3g $^{1}H,^{7}Li$-HOESY spectrum of **3** (500 MHz, 263 K, C_7D_8; $\tau = 0.05$ s).

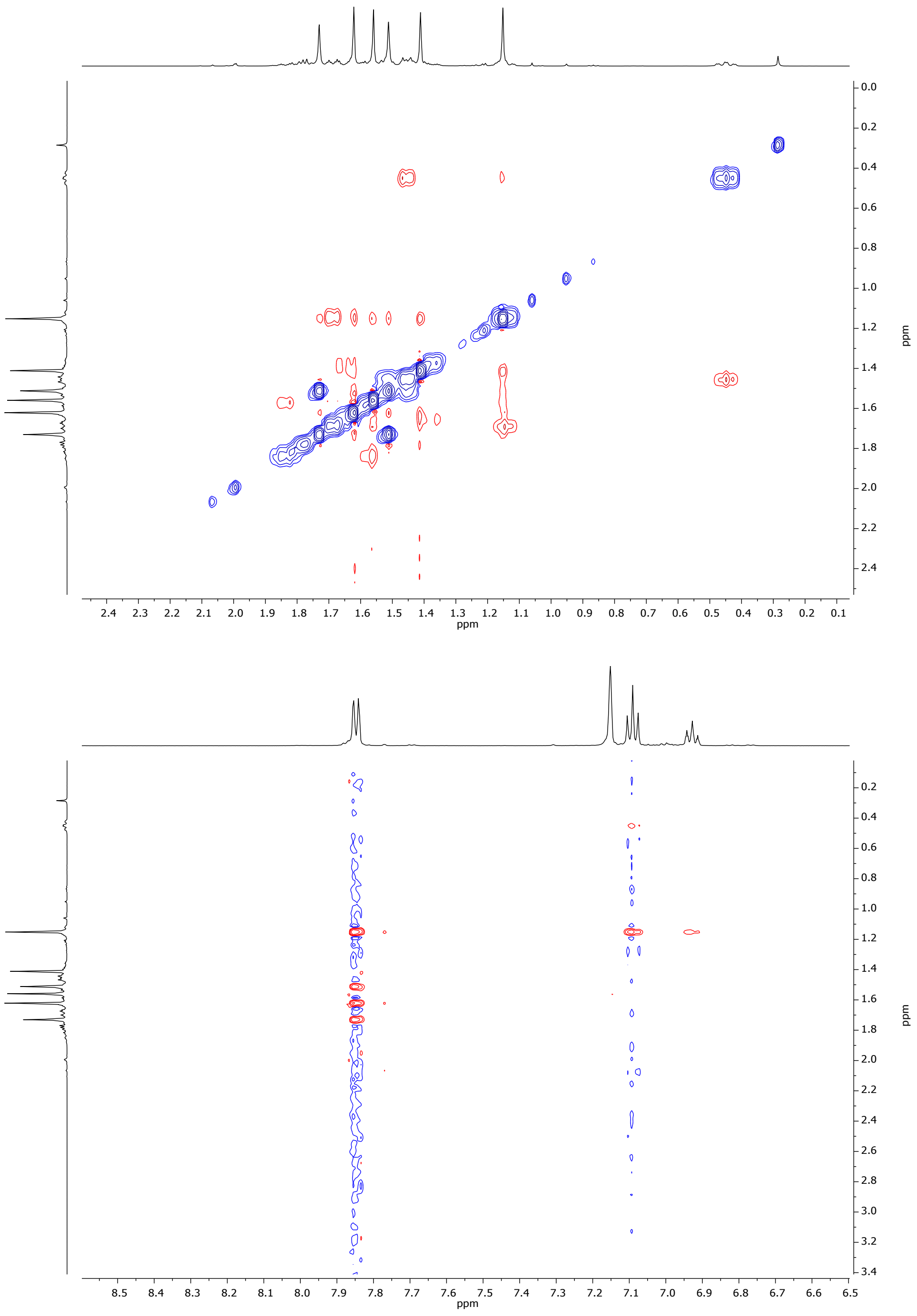

Figure S3h $^{1}H,^{1}H$-NOESY spectra of **3** (500 MHz, 298 K, C_6D_6, τ = 0.6 s).

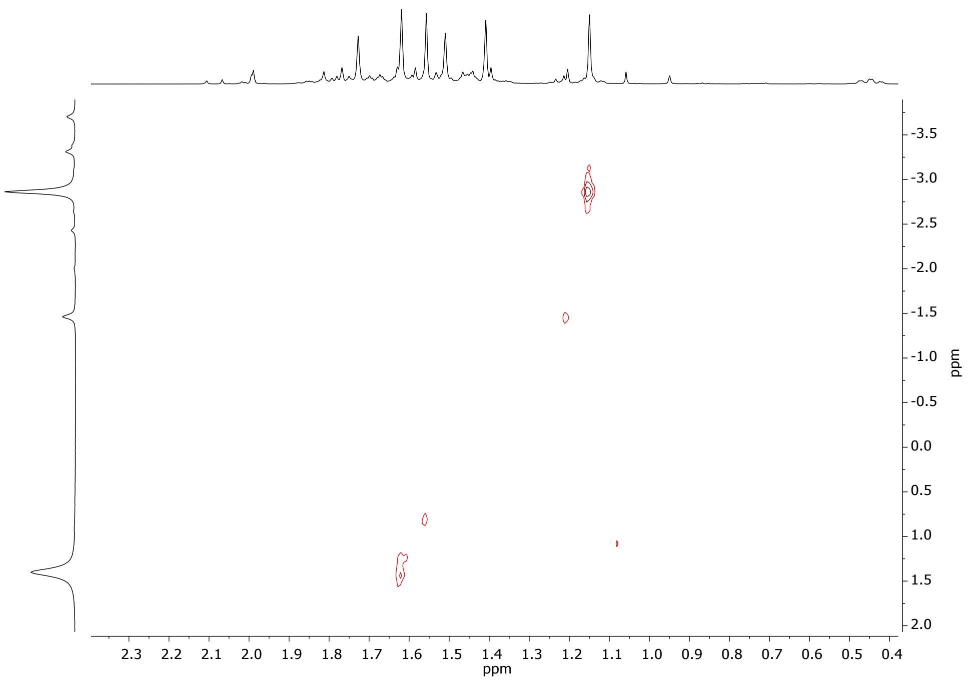

Figure S3i $^{1}H,^{7}Li$-HOESY spectrum of **3** (500 MHz, 298 K, C_6D_6, τ = 0.05 s).

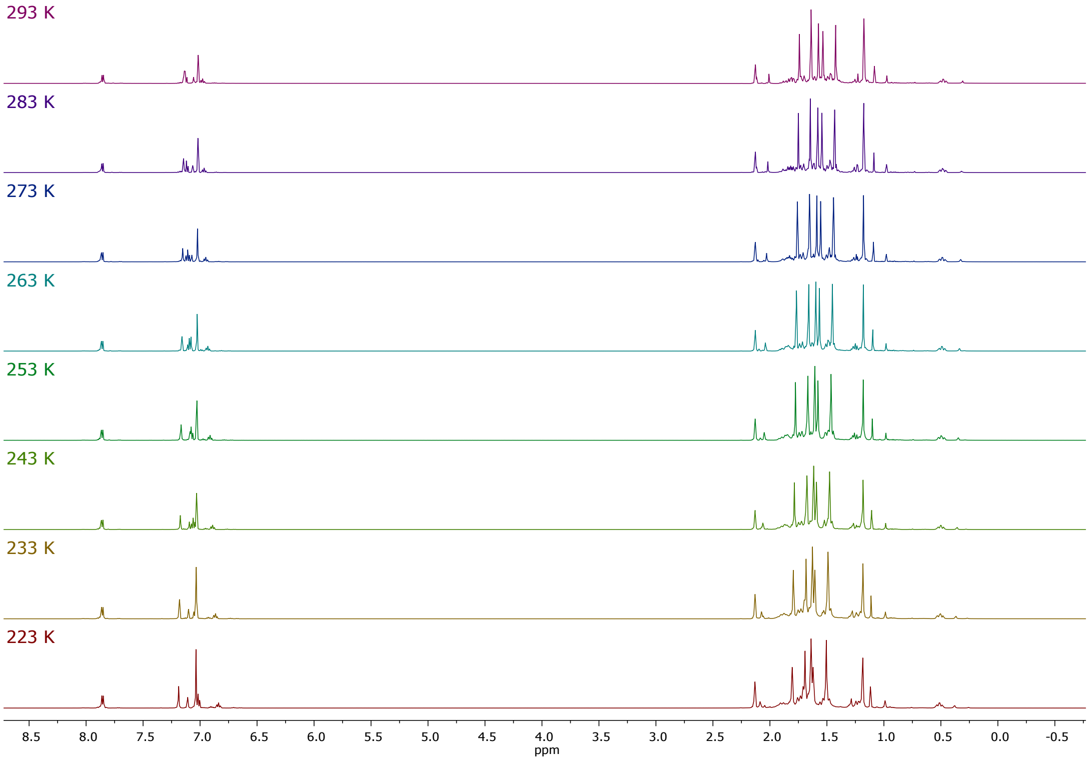

Figure S3j Variable temperature ^{1}H NMR spectra of **3** (500 MHz, T (specified), C_7D_8).

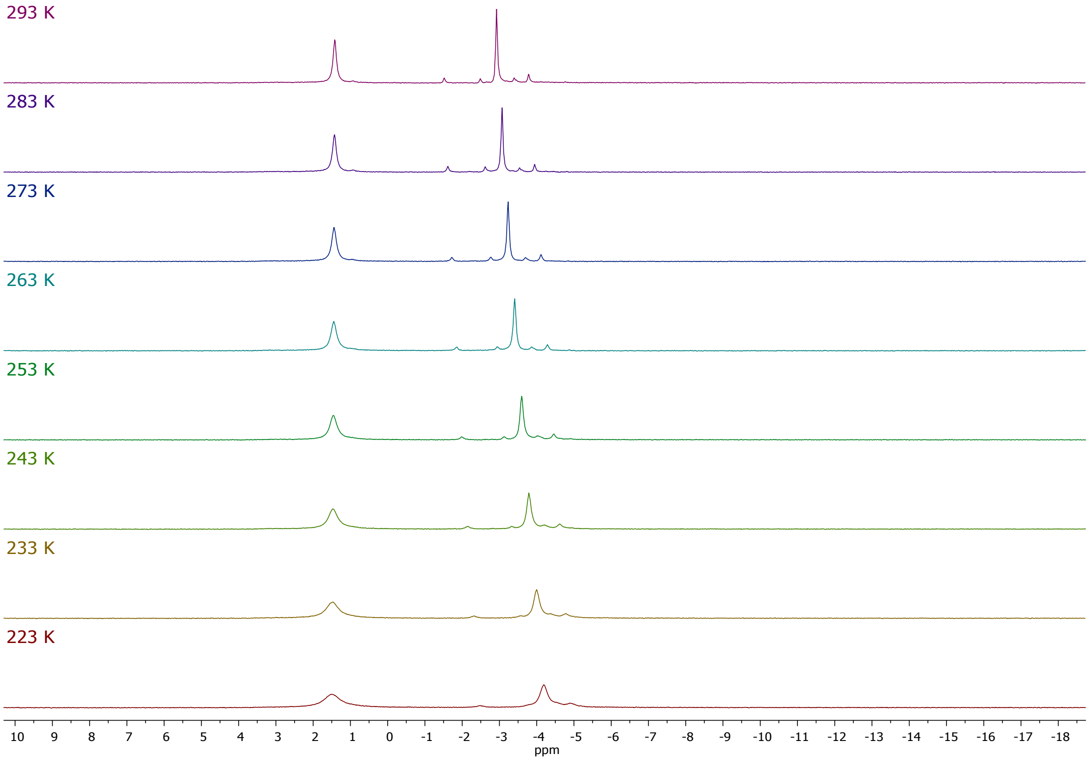

Figure S3k Variable temperature ^{7}Li NMR spectra of **3** (194 MHz, T (specified), C_7D_8).

Additional characterisation of 4

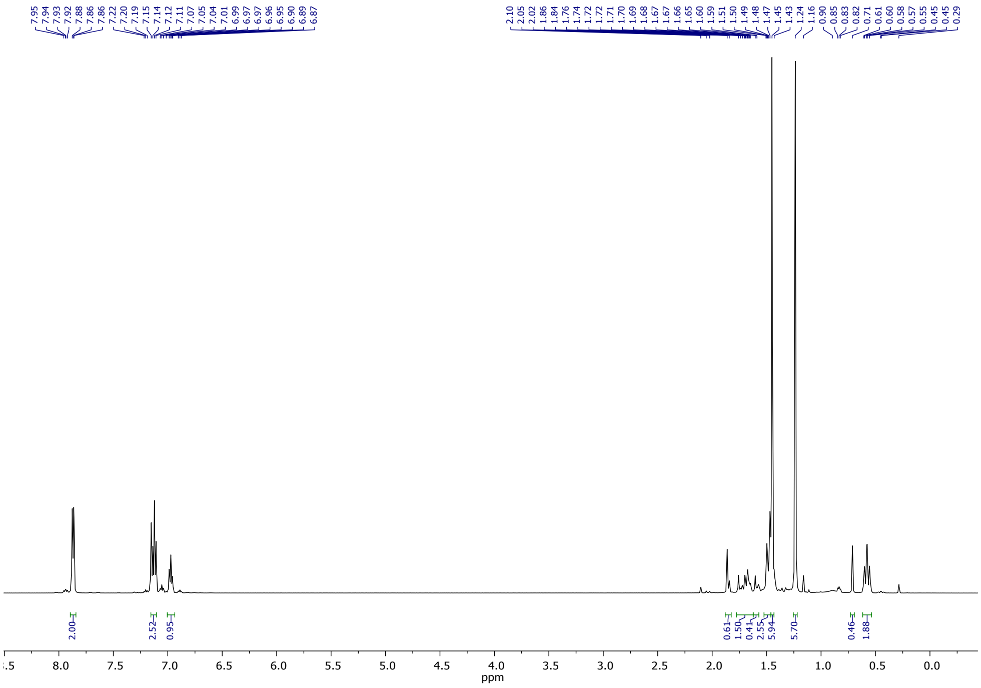

Figure S4a ^{1}H NMR spectrum of **4** (500 MHz, 298 K, C_6D_6).

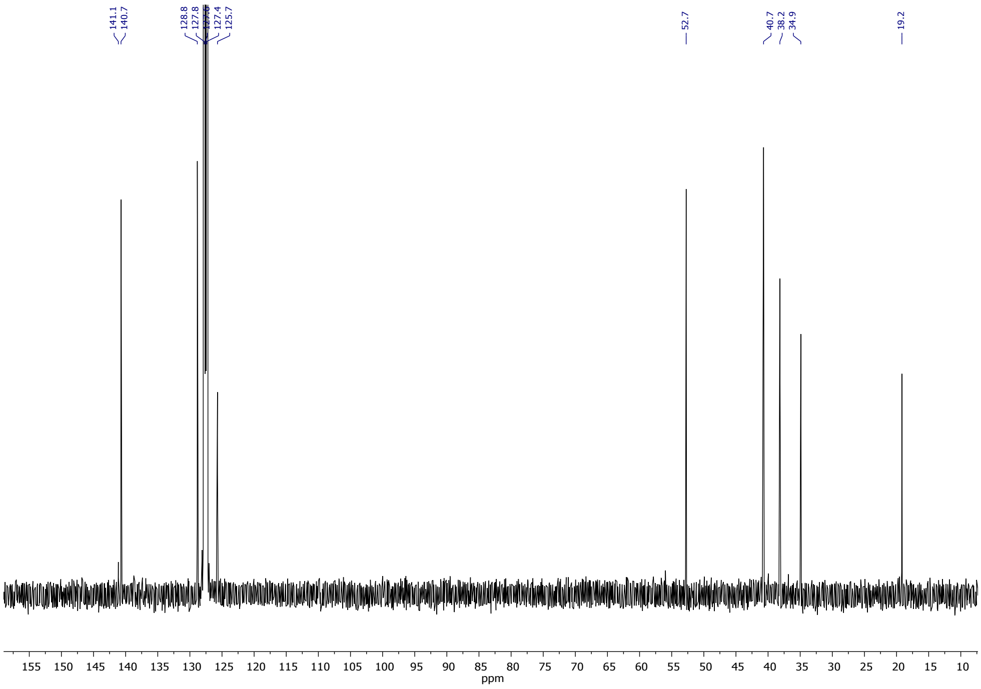

Figure S4b ^{13}C NMR spectrum **4** (125 MHz, 298 K, C_6D_6).

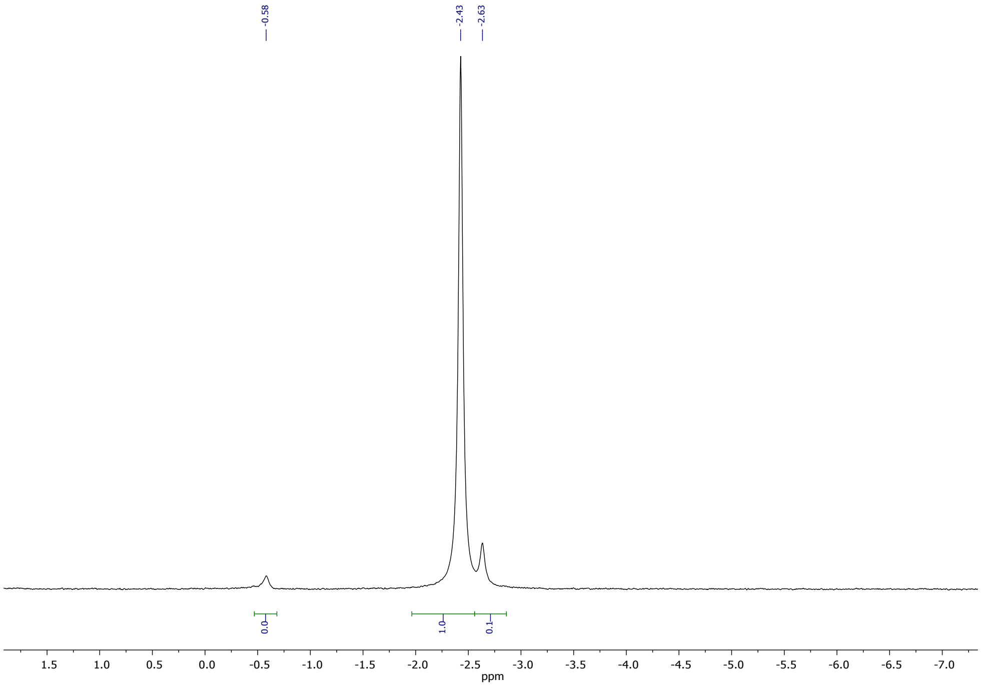

Figure S4c ^{7}Li NMR spectrum of **4** (194 MHz, 298 K, C_6D_6).

Additional characterisation of 5

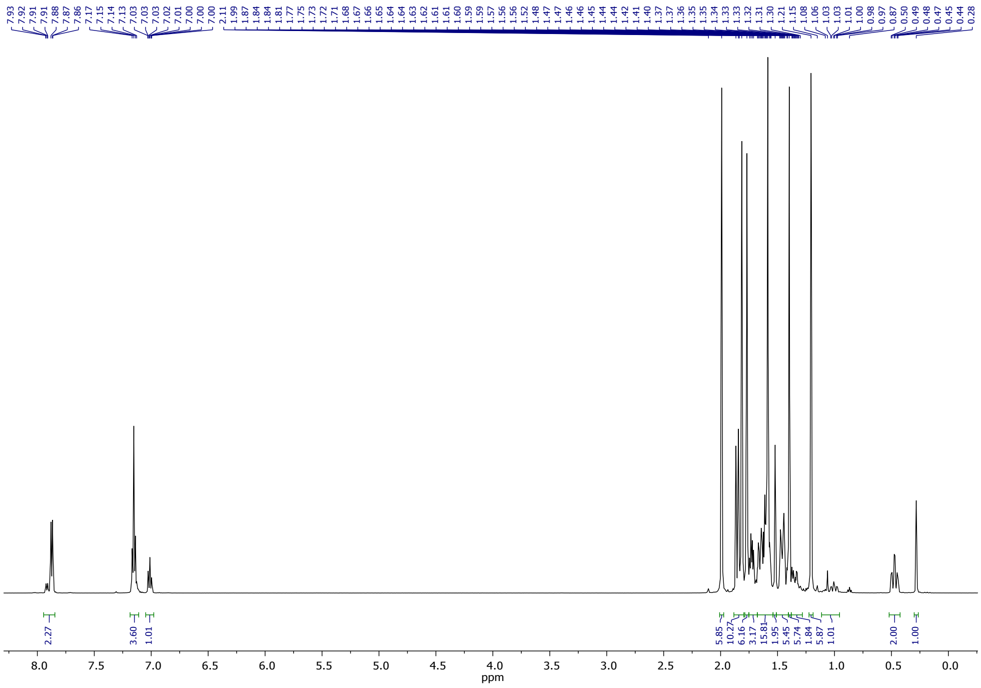

Figure S5a ^{1}H NMR spectrum of **5** (500 MHz, 298 K, C_6D_6).

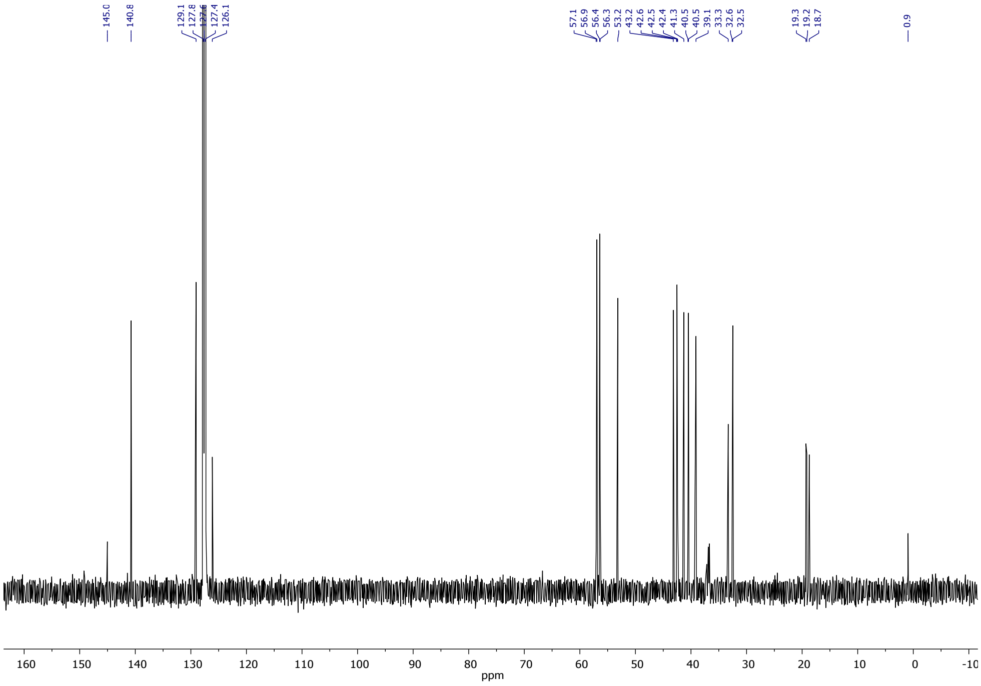

Figure S5b ^{13}C NMR spectrum of **5** (125 MHz, 298 K, C_6D_6).

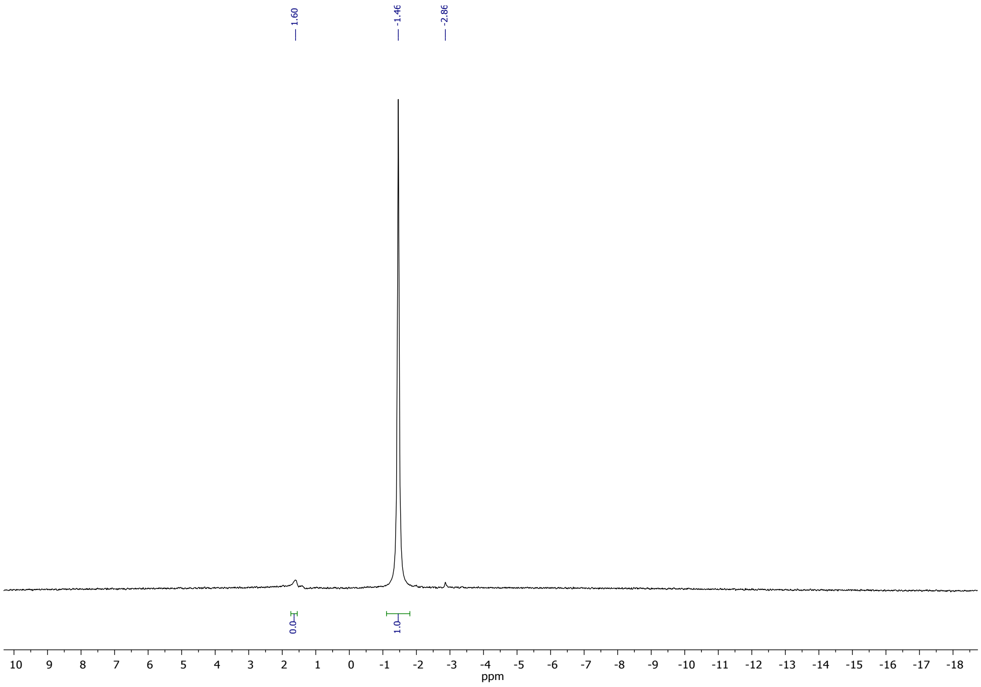

Figure S5c ^{7}Li NMR spectrum of **5** (194 MHz, 298 K, C_6D_6).

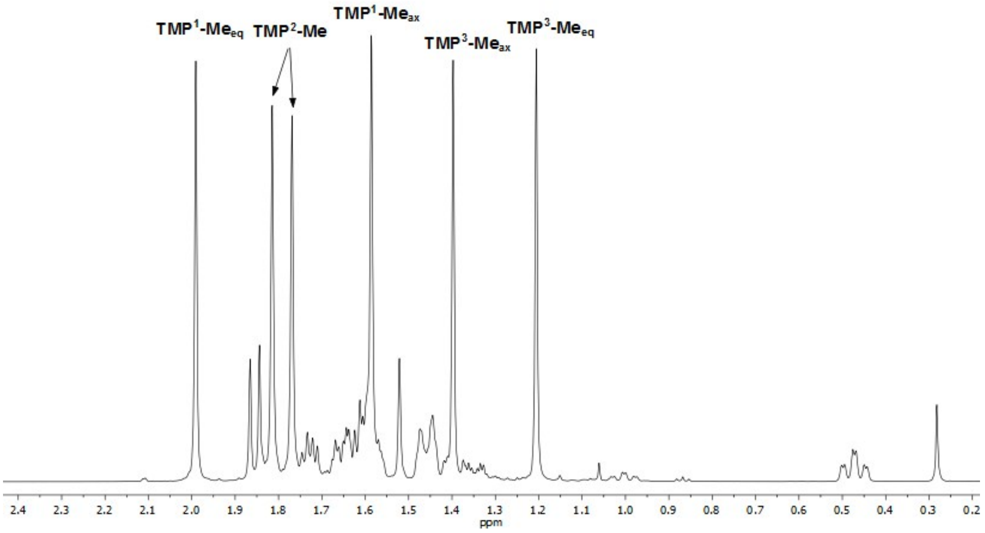

Figure S5d ^{1}H NMR spectrum of **5** (500 MHz, 298 K, C_6D_6); expansion of aliphatic region.

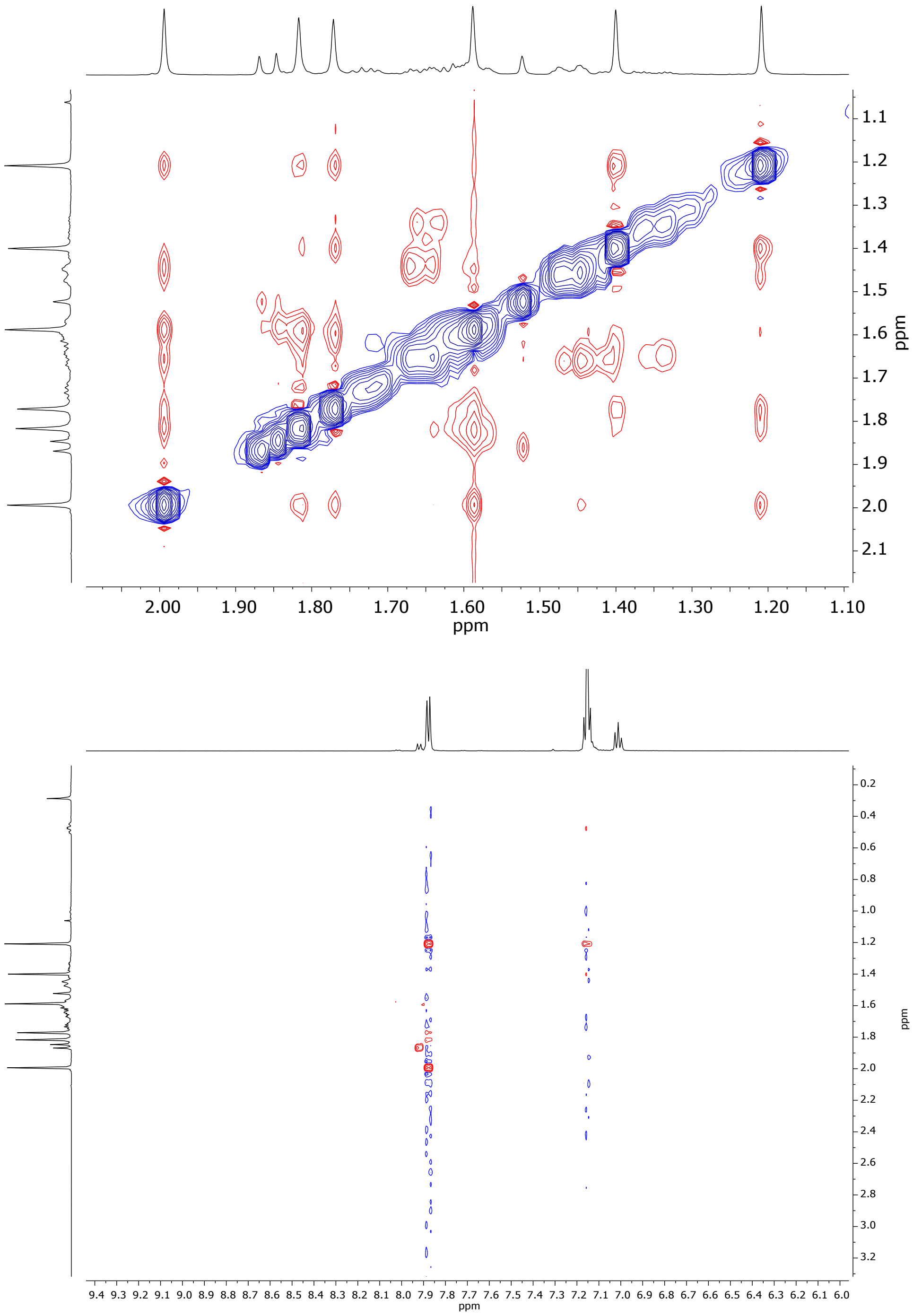

Figure S5e ^{1}H,^{1}H-NOESY spectra of **5** (500 MHz, 298 K, C_6D_6, τ = 0.6 s).

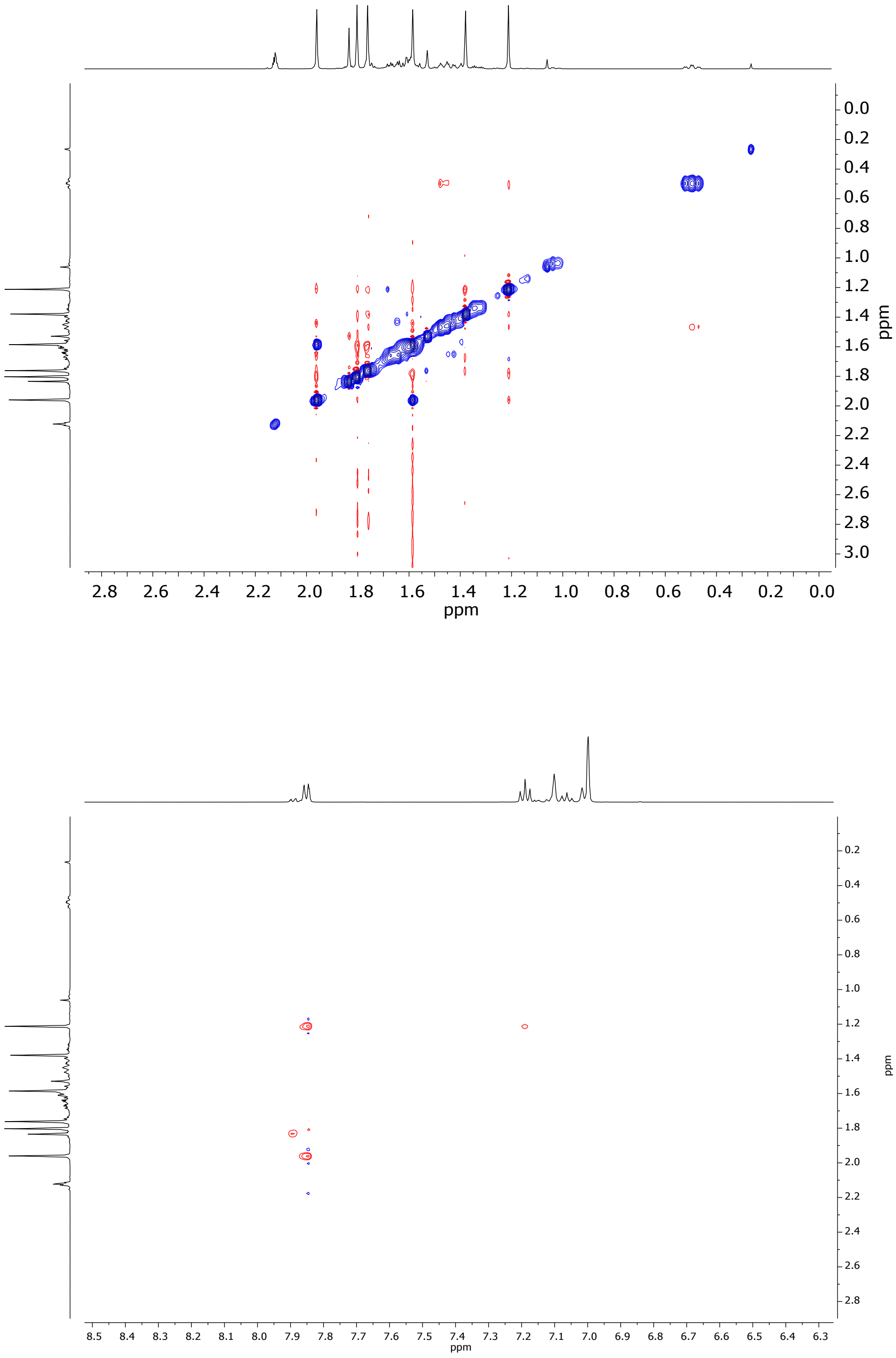

Figure S5f ^{1}H,^{1}H-NOESY spectra of **5** (500 MHz, 353 K, C_7D_8, τ = 0.6 s).

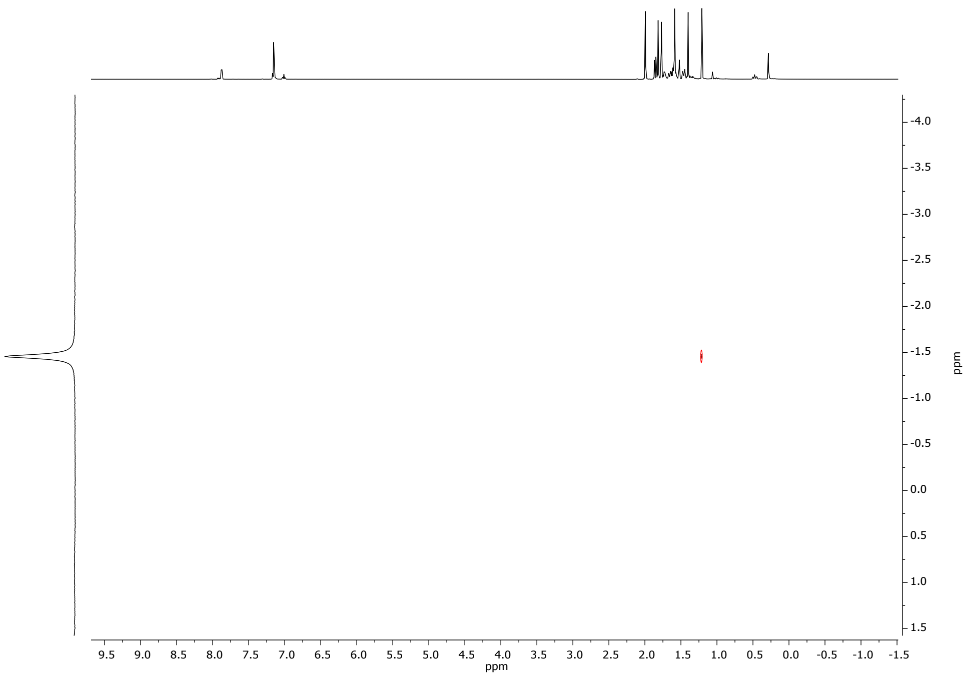

Figure S5g $^{1}H,^{7}Li$-HOESY spectrum of **5** (500 MHz, 298 K, C_6D_6, $\tau = 0.1$).

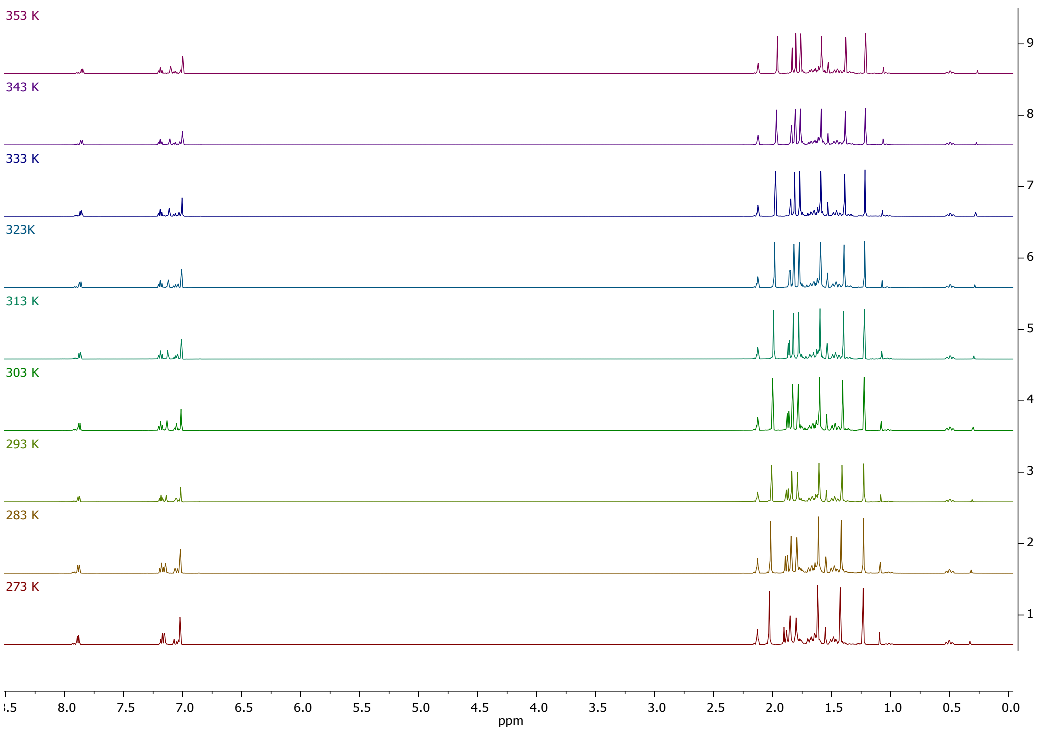

Figure S5h Variable temperature ^{1}H NMR spectra of **5** (500 MHz, T (specified), C_7D_8).

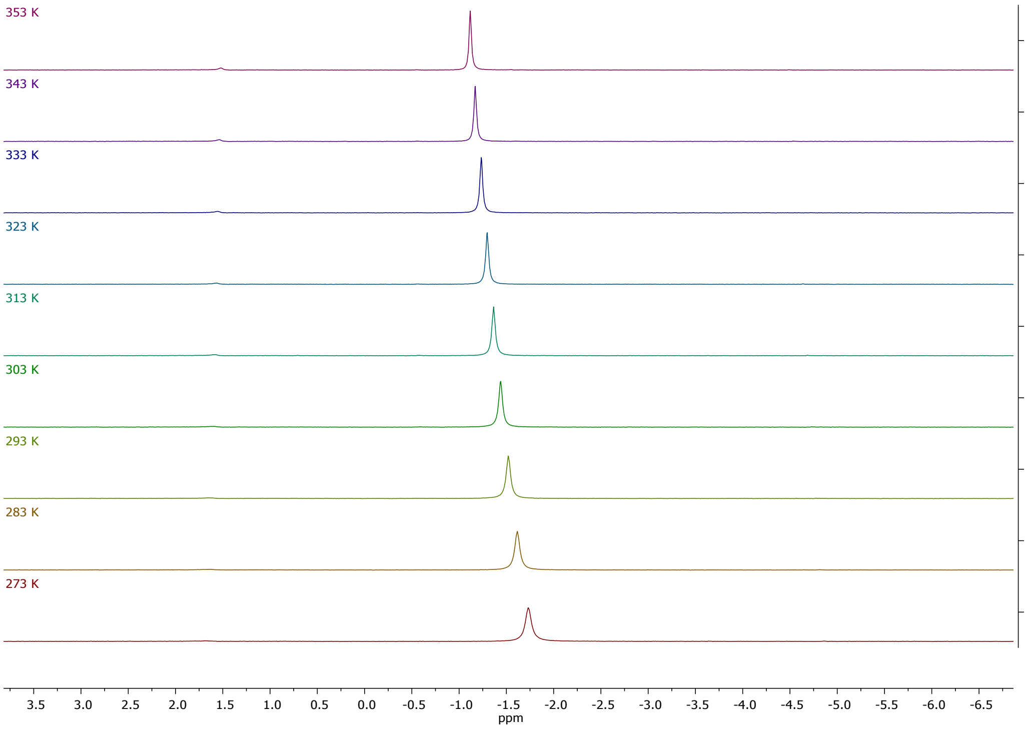

Figure S5i Variable temperature ^{7}Li NMR spectra of **5** (194 MHz, T (specified), C_7D_8).

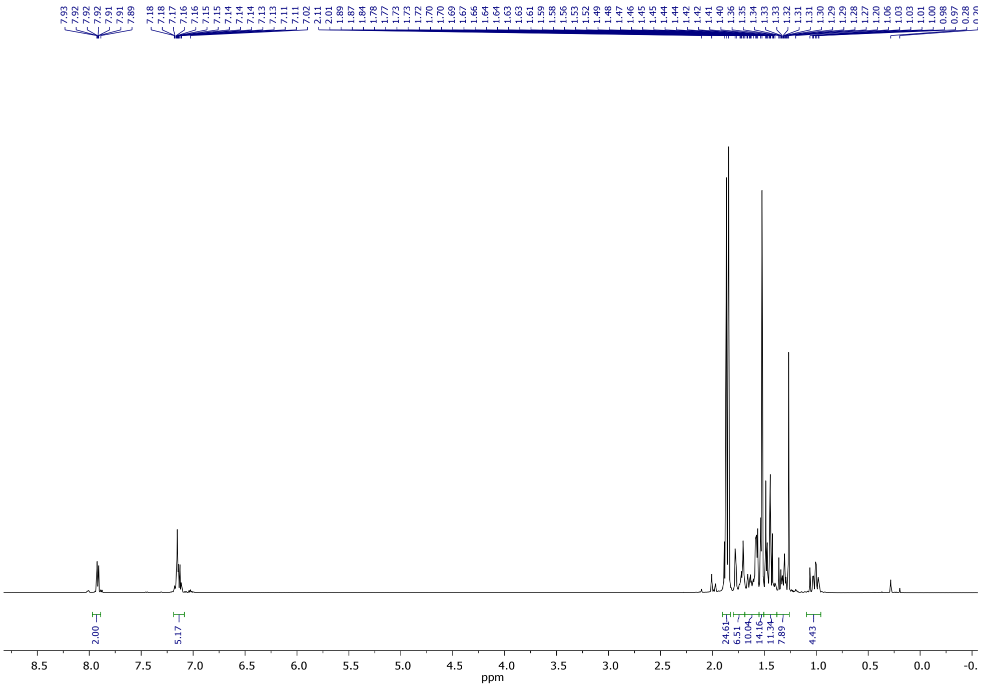

Figure S5j ^{1}H NMR spectrum (500 MHz, 298 K, C_6D_6) on a 1:1 mixture of **5** and ^{t}BuOCu, heated to 50°C for 24 h (*cf.* Fig. S6a).

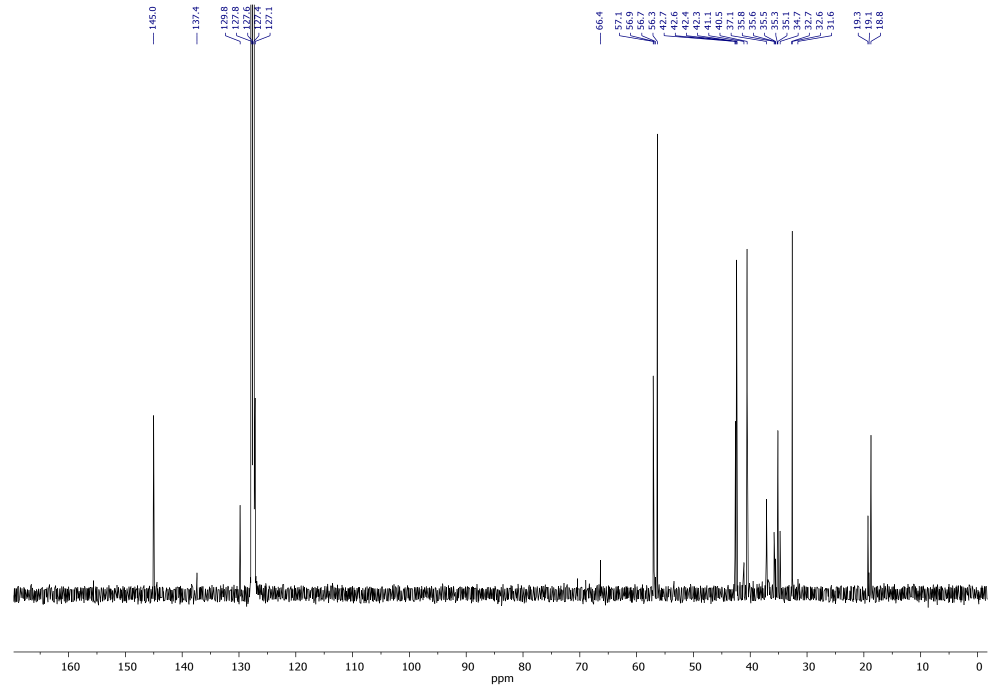

Figure S5k ^{13}C NMR spectrum (125 MHz, 298 K, C_6D_6) on a 1:1 mixture of **5** and ^{t}BuOCu, heated to 50°C for 24 h (*cf*. Fig. S6b).

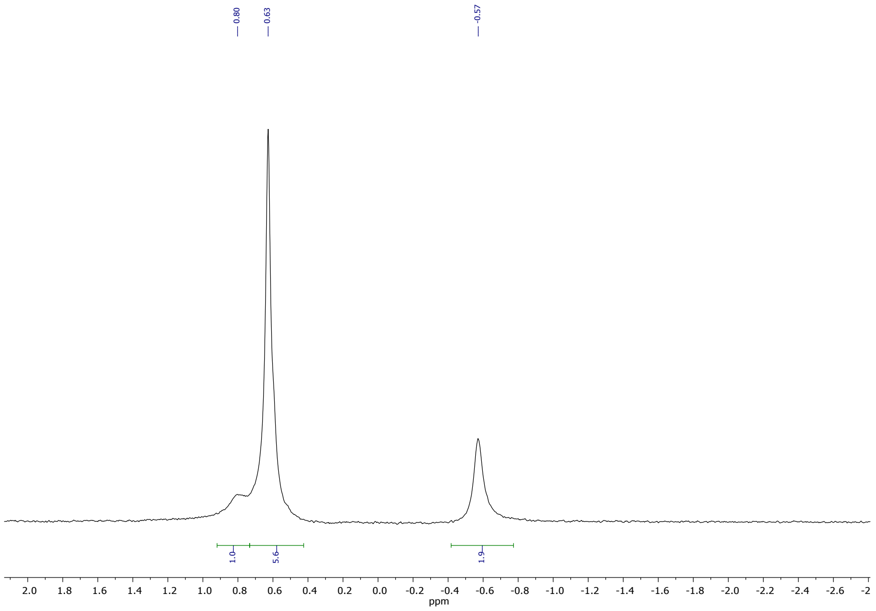

Figure S5l ^{7}Li NMR spectrum (125 MHz, 298 K, C_6D_6) on a 1:1 mixture of **5** and ^{t}BuOCu, heated to 50°C for 24 h. The major resonance at δ 0.63 ppm is due to ^{t}BuOLi.[1]

[1] J. F. Allan, R. Nassar, E. Specht, A. Beatty, N. Calin and K. Henderson, *J. Am. Chem. Soc.*, 2004, **126**, 484-485.

Additional Characterisation of 6

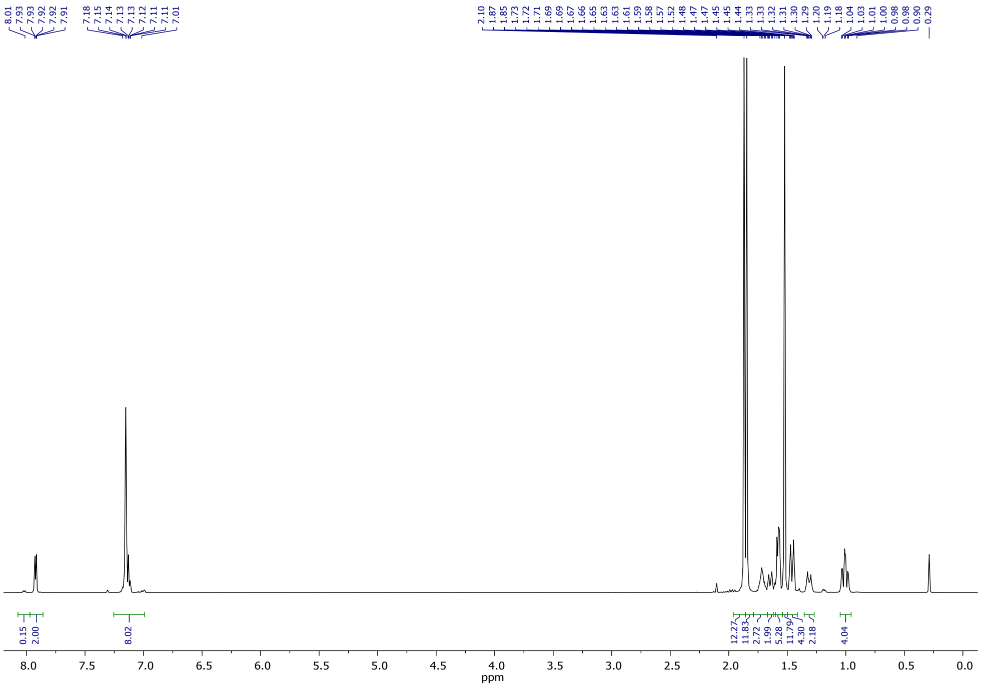

Figure S6a. ^{1}H NMR spectrum of **6** (500 MHz, 298 K, C_6D_6).

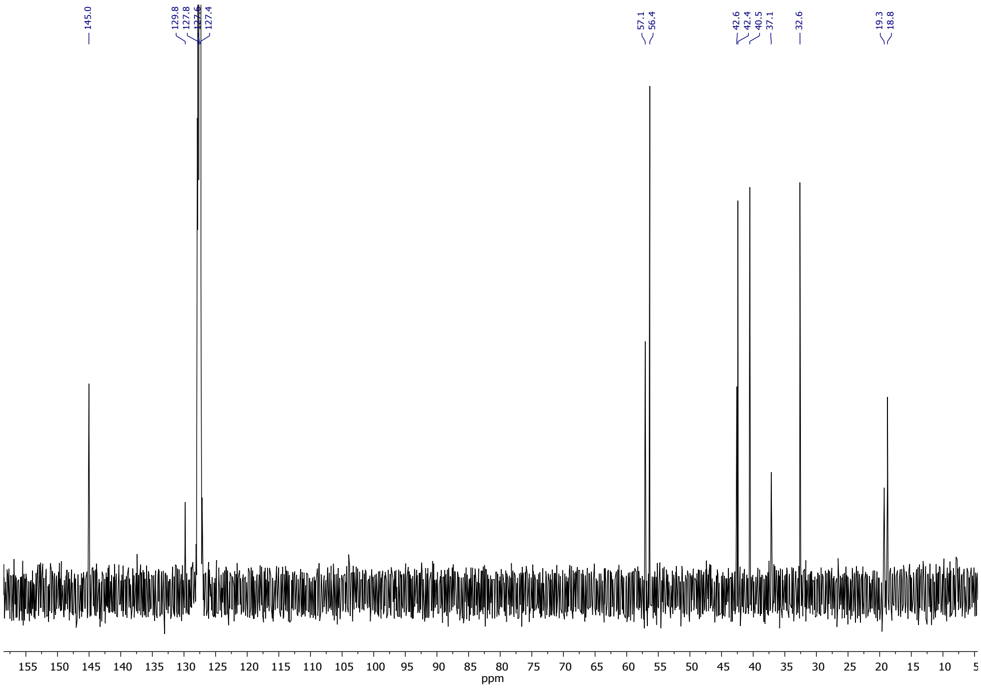

Figure S6b. ^{13}C NMR spectrum of **6** (125 MHz, 298 K, C_6D_6).

Additional characterisation of 7

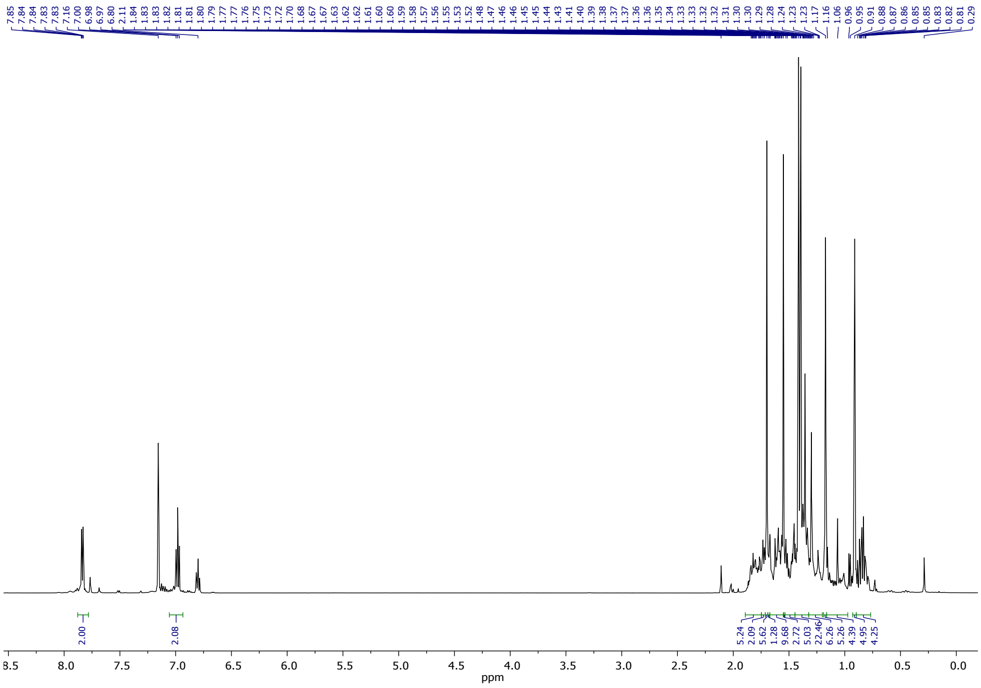

Figure S7a ^{1}H NMR spectrum of **7** (500 MHz, 298 K, C_6D_6).

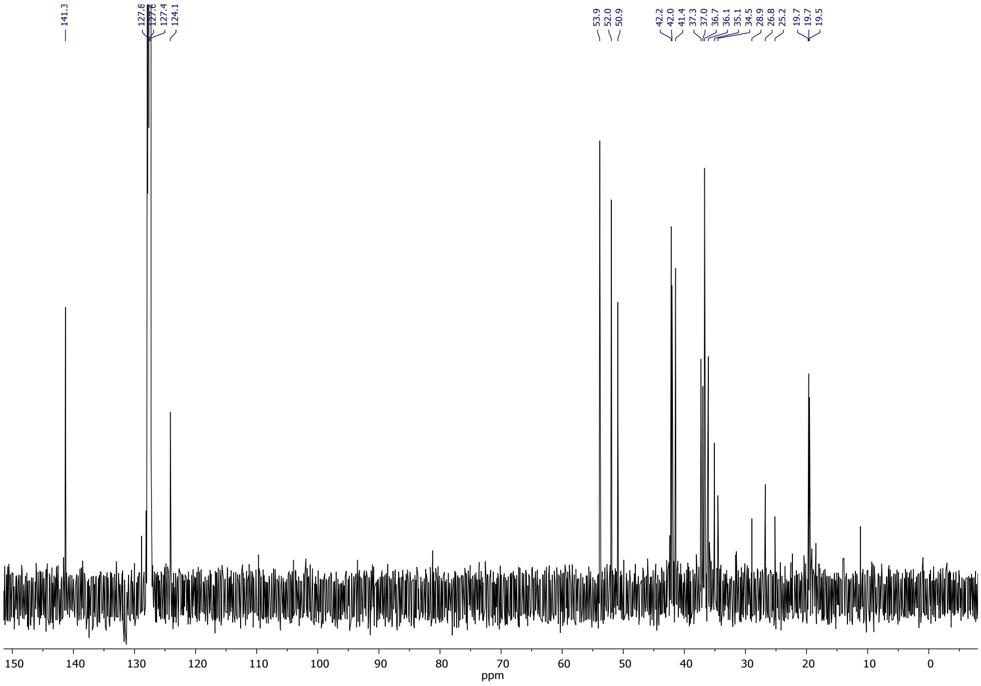

Figure S7b ^{13}C NMR spectrum of **7** (125 MHz, 298 K, C_6D_6).

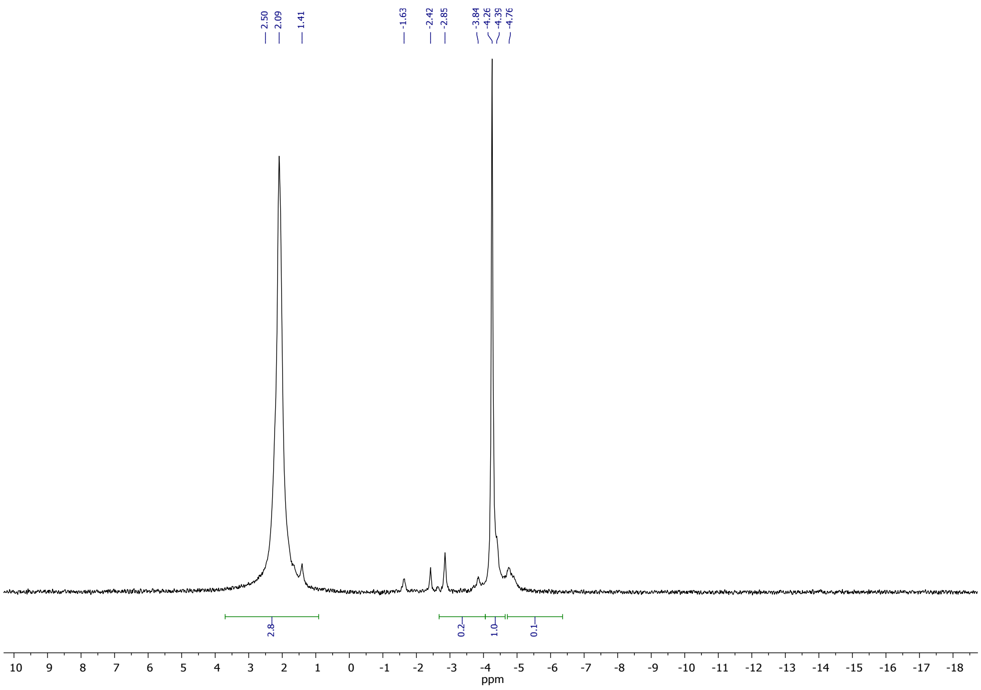

Figure S7c ^{7}Li NMR spectrum of **7** (194 MHz, 298 K, C_6D_6).

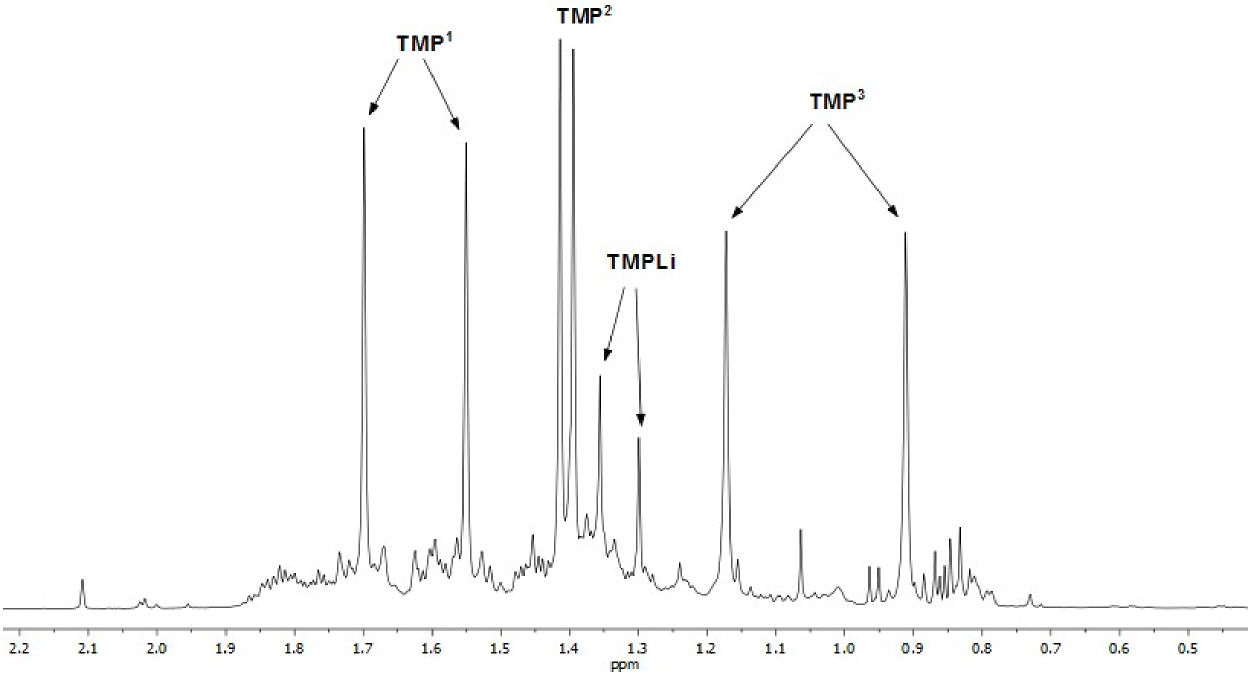

Figure S7d ^{1}H NMR spectrum of **7** (500 MHz, 298 K, C_6D_6).

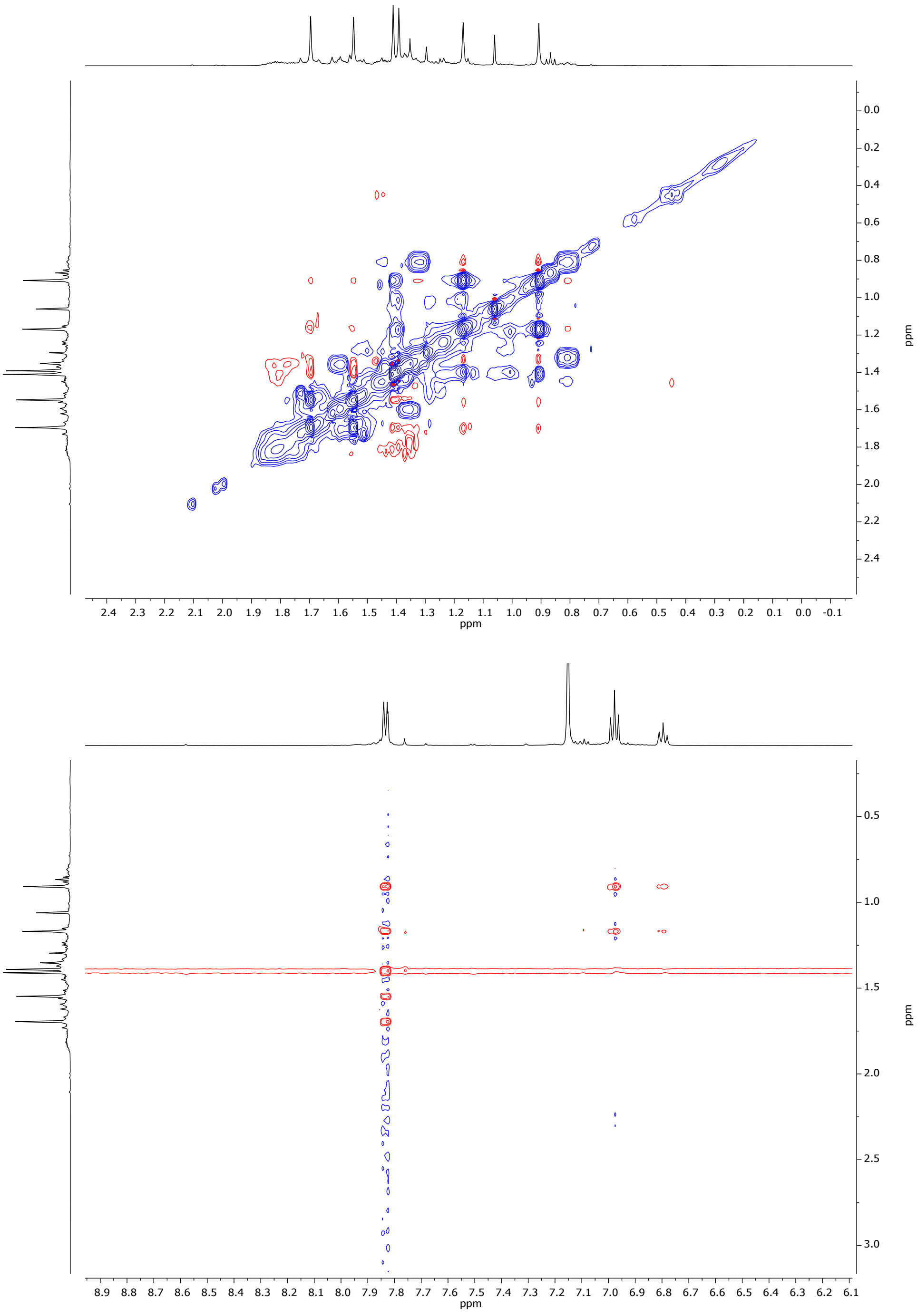

Figure S7e $^{1}H,^{1}H$-NOESY spectrum of **7** (500 MHz, 298 K, C_6D_6, $\tau = 0.6$ s).

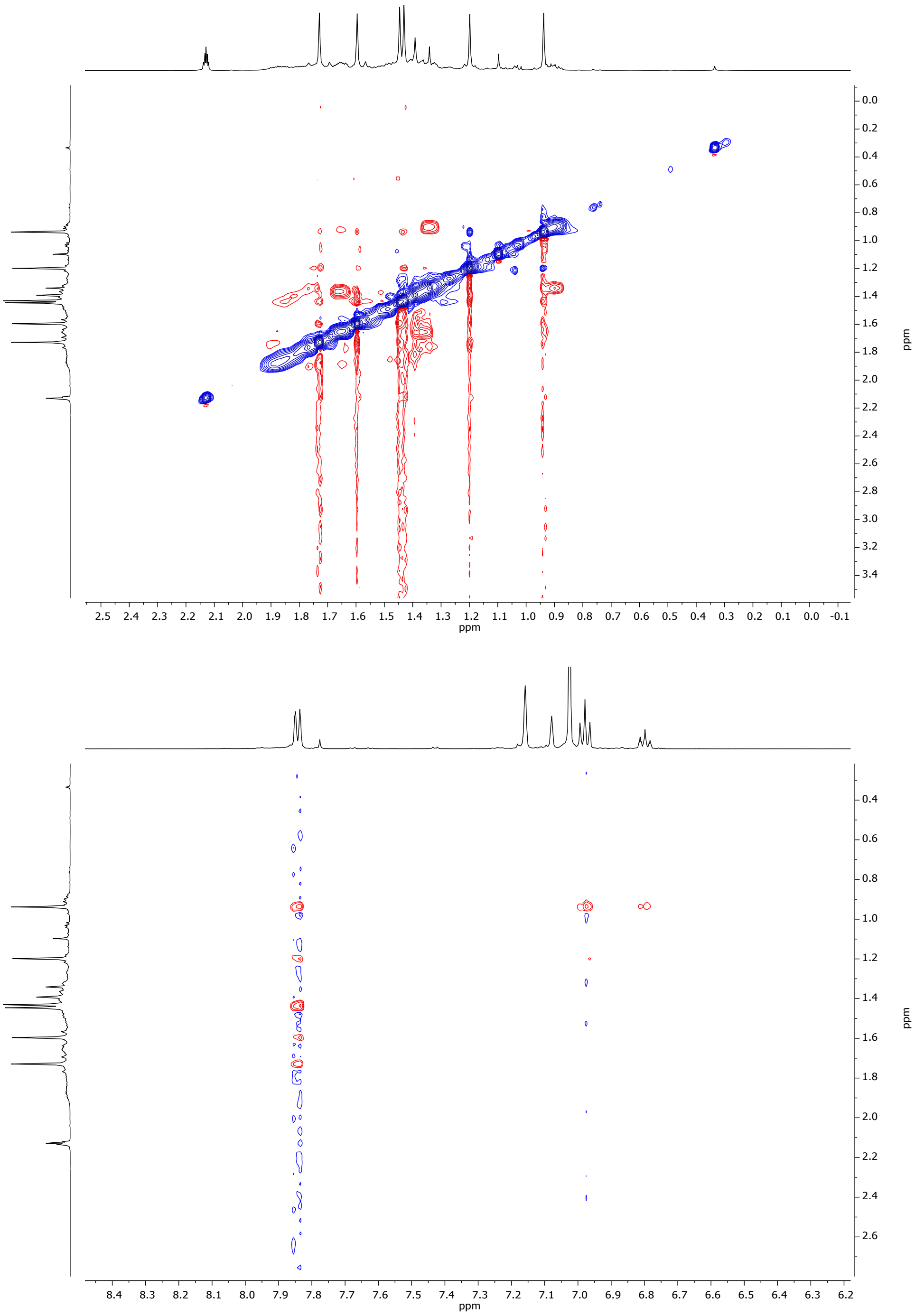

Figure S7f ^{1}H,^{1}H-NOESY spectra of **7** (500 MHz, 263 K, C_7D_8, $\tau = 0.6$ s).

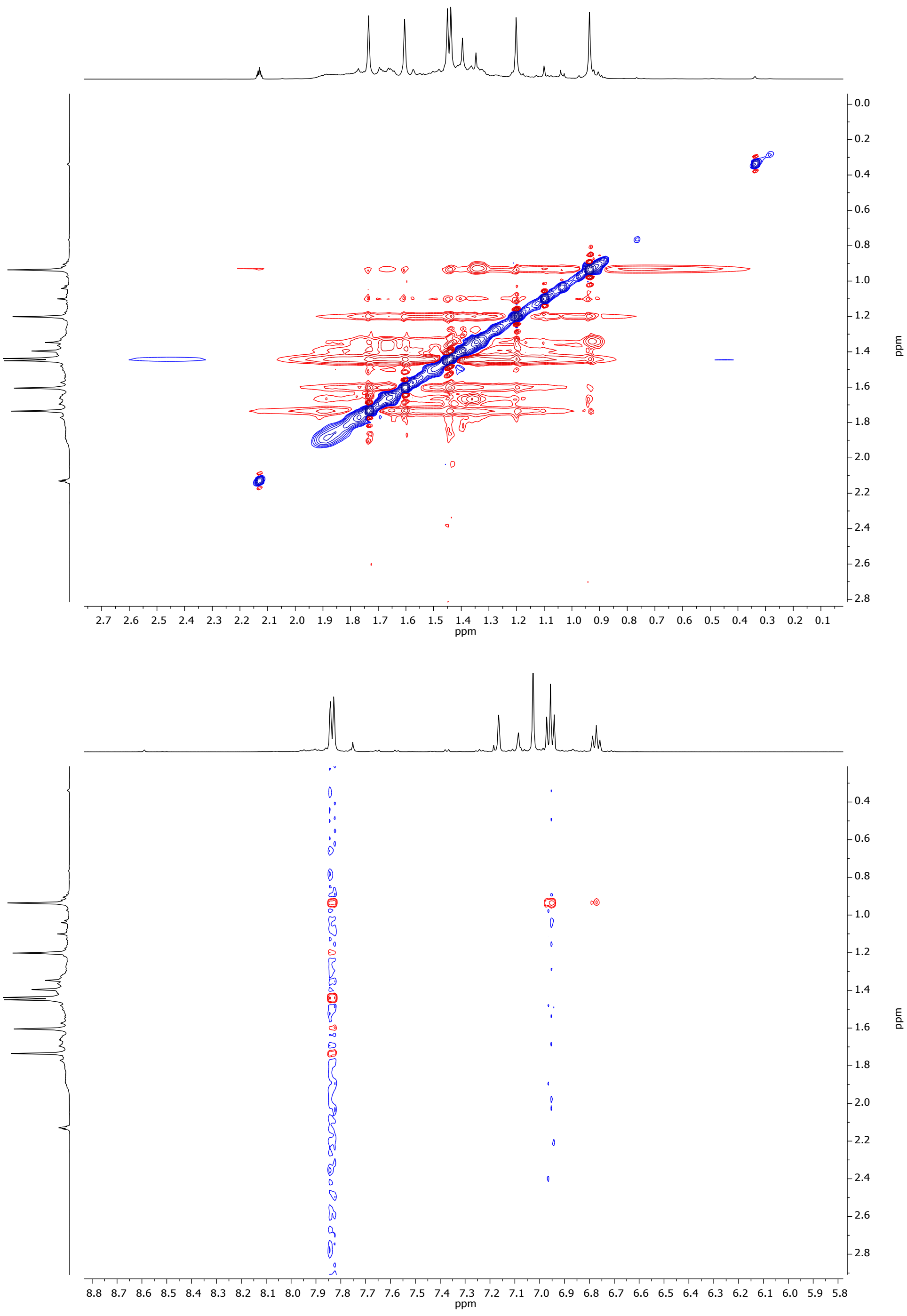

Figure S7g ^{1}H,^{1}H-NOESY spectrum of **7** (500 MHz, 253 K, C_7D_8, $\tau = 0.6$ s).

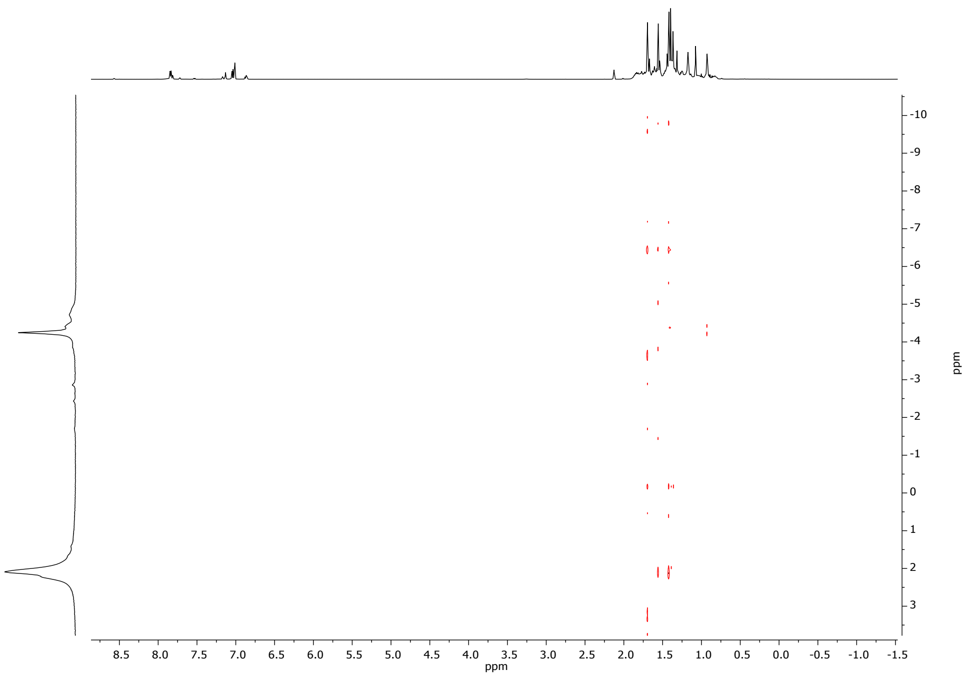

Figure S7h $^{1}H,^{7}Li$-HOESY spectrum of **7** (500 MHz, 298 K, C_6D_6, $\tau = 0.05$ s).

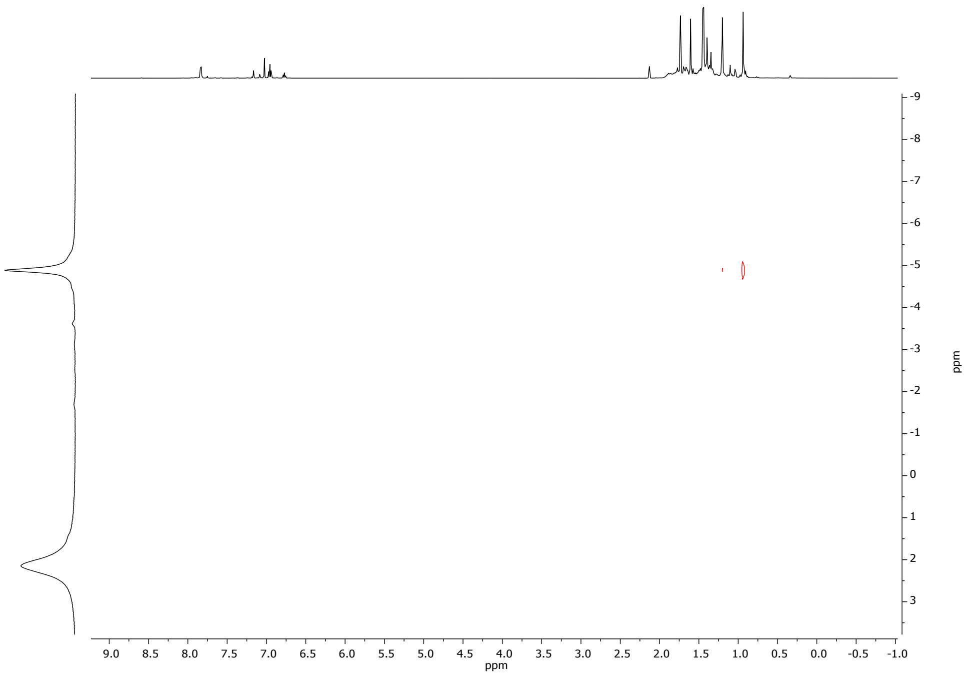

Figure S7i $^{1}H,^{7}Li$-HOESY spectrum of **7** (500 MHz, 253 K, C_7D_8).

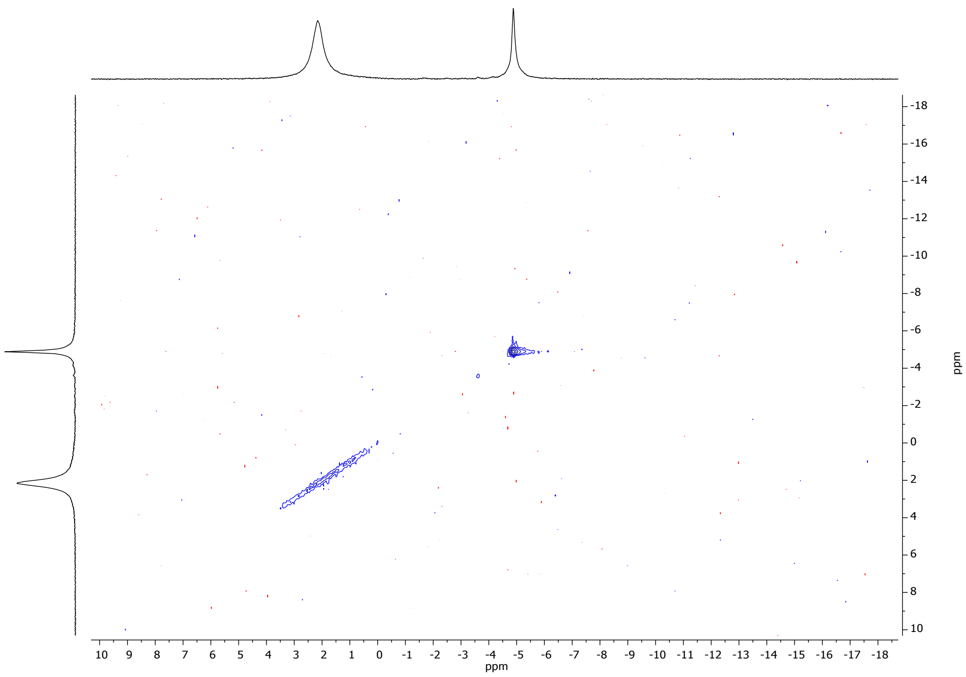

Figure S7j $^{7}Li,^{7}Li$-NOESY spectrum of **7** (194 MHz, 253 K, C_7D_8).

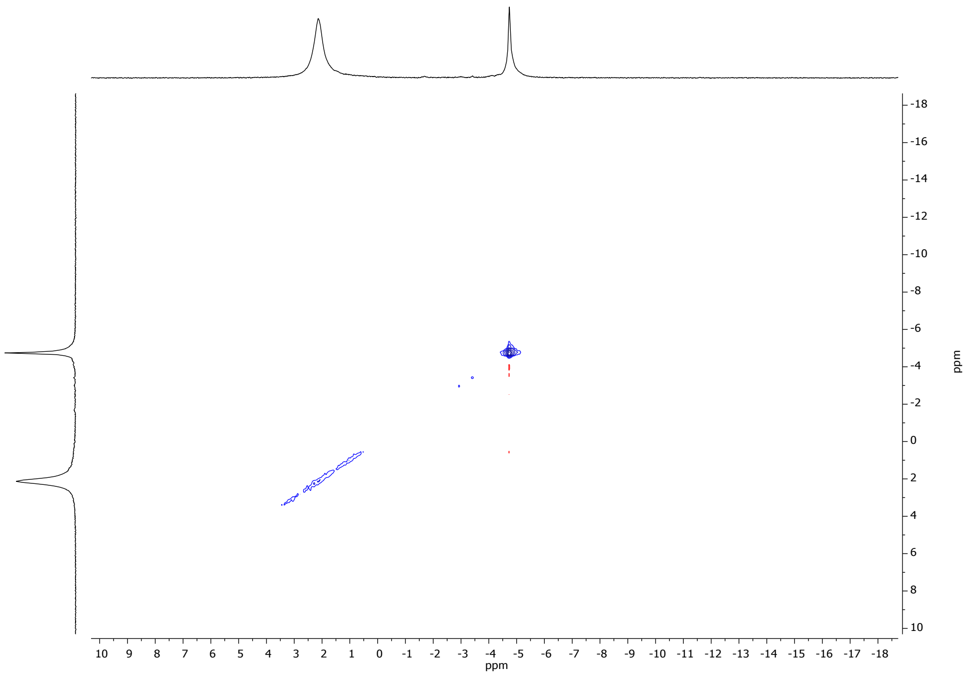

Figure S7k $^{7}Li,^{7}Li$-NOESY spectrum of **7** (194 MHz, 263 K, C_7D_8).

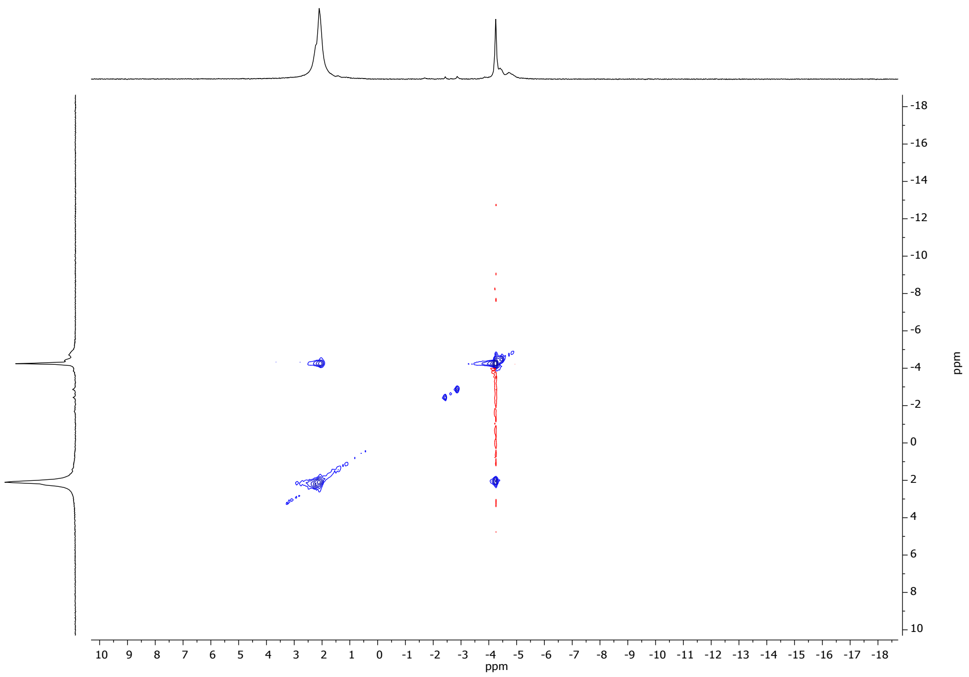

Figure S7l ^{7}Li,^{7}Li-NOESY spectrum of **7** (194 MHz, 298 K, C_7D_8).

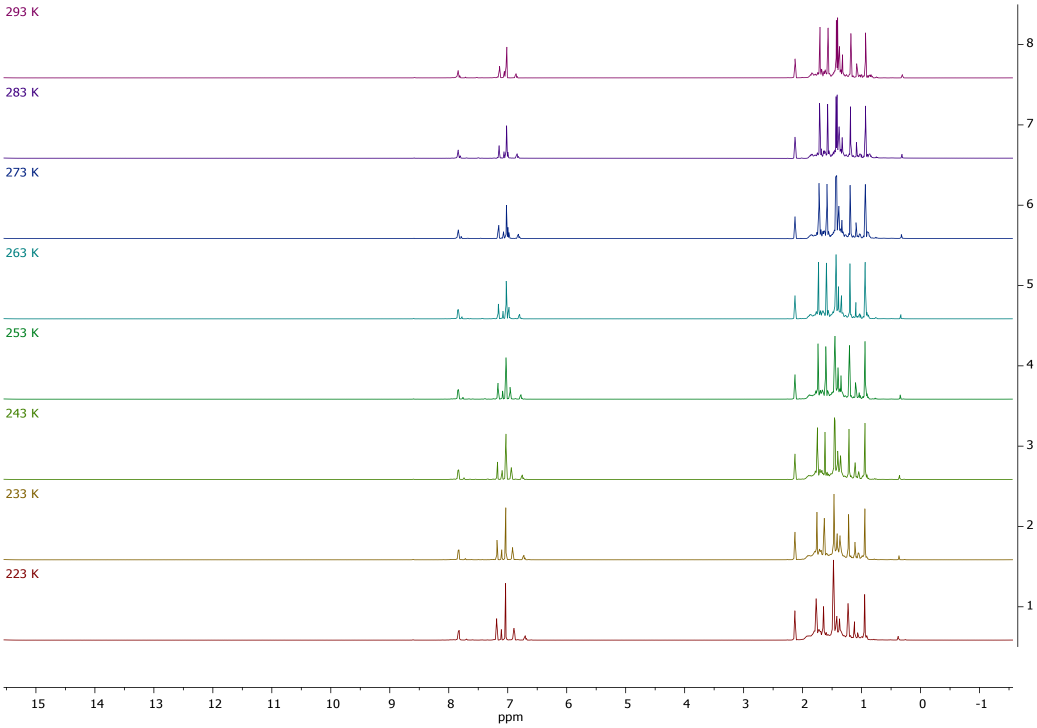

Figure S7m Variable temperature ^{1}H NMR spectra of **7** (500 MHz, T (specified), C_7D_8).

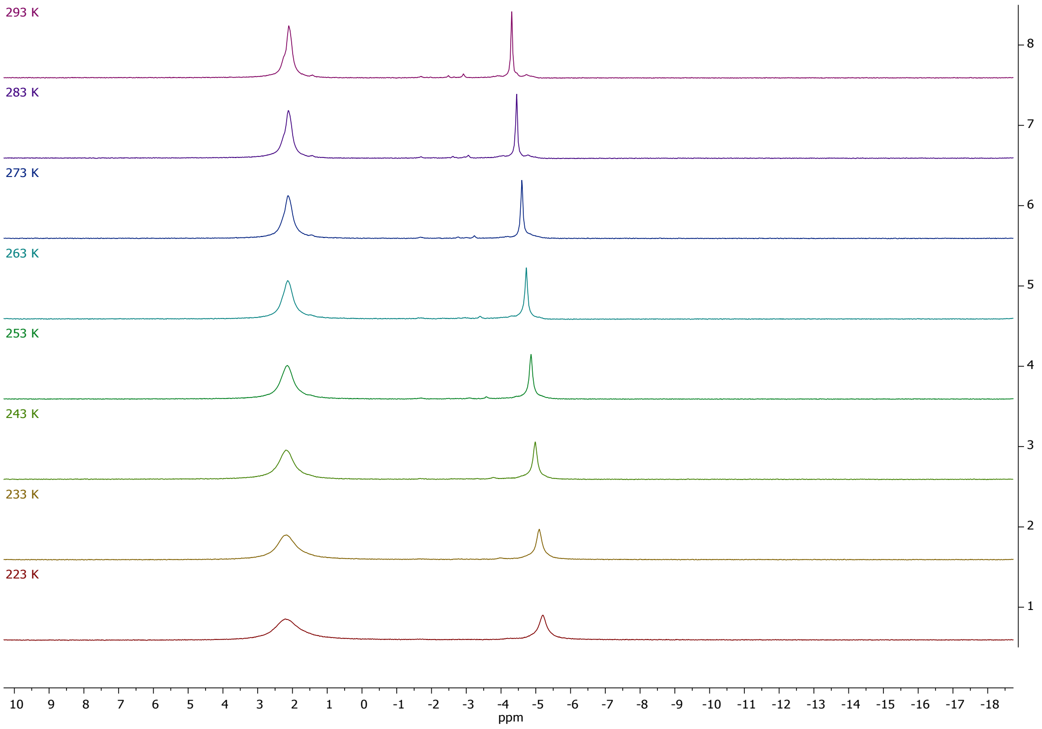

Figure S7n Variable temperature ^{7}Li NMR spectra of **7** (194 MHz, T (specified), C_7D_8).

DFT calculations

Computational Details

All calculations were carried with the Gaussian 16 program package.[2] The molecular structures and harmonic vibrational frequencies were obtained using the hybrid density functional method based on M06 functional.[3] We used Ahlrichs' SVP[4] all–electron basis set for Cu atom and 6–31+G* for the other atoms. Geometry optimization and vibrational analysis were performed at the same level. All the optimizations were calculated without any symmetry assumptions, and characterized by normal coordinate analysis at the same level of theory (number of imaginary frequencies, NIMAG, 0 for minima).

---

[2] Gaussian 16, Revision B.01, M. J. Frisch, G. W. Trucks, H. B. Schlegel, G. E. Scuseria, M. A. Robb, J. R. Cheeseman, G. Scalmani, V. Barone, G. A. Petersson, H. Nakatsuji, X. Li, M. Caricato, A. V. Marenich, J. Bloino, B. G. Janesko, R. Gomperts, B. Mennucci, H. P. Hratchian, J. V. Ortiz, A. F. Izmaylov, J. L. Sonnenberg, D. Williams-Young, F. Ding, F. Lipparini, F. Egidi, J. Goings, B. Peng, A. Petrone, T. Henderson, D. Ranasinghe, V. G. Zakrzewski, J. Gao, N. Rega, G. Zheng, W. Liang, M. Hada, M. Ehara, K. Toyota, R. Fukuda, J. Hasegawa, M. Ishida, T. Nakajima, Y. Honda, O. Kitao, H. Nakai, T. Vreven, K. Throssell, J. A. Montgomery, Jr., J. E. Peralta, F. Ogliaro, M. J. Bearpark, J. J. Heyd, E. N. Brothers, K. N. Kudin, V. N. Staroverov, T. A. Keith, R. Kobayashi, J. Normand, K. Raghavachari, A. P. Rendell, J. C. Burant, S. S. Iyengar, J. Tomasi, M. Cossi, J. M. Millam, M. Klene, C. Adamo, R. Cammi, J. W. Ochterski, R. L. Martin, K. Morokuma, O. Farkas, J. B. Foresman, and D. J. Fox, Gaussian, Inc., Wallingford CT, 2016.

[3] (a) Y. Zhao, D. G. Truhlar, *Theor. Chem. Acc.,* 2008, **120**, 215-241; (b) Y. Zhao, D. G. Truhlar, *Acc. Chem. Res.,* 2008, **41**, 157-167.

[4] A. Schäfer, H. Horn, R. Ahlrichs, *J. Chem. Phys.,* 1992, **97***,* 2571-2577.

Structure optimisation for 1

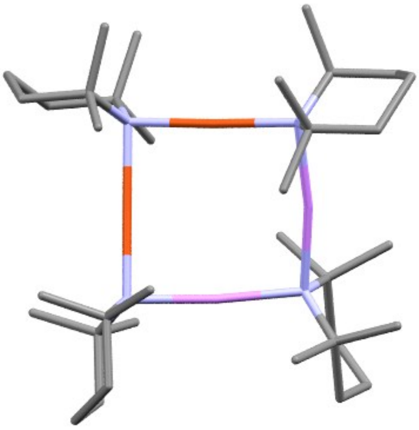

Figure S8 Optimised DFT molecular structure of **1**. ΔG = −3092241.394 (ΔE = −3092843.02) kcal.mol^{-1}.

Structure optimisation for 2

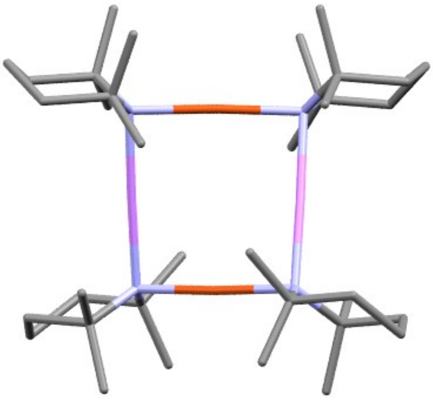

Figure S9 Optimised DFT structure of **2**. ΔG = −3092243.858 (ΔE = −3092845.838) kcal.mol^{-1}.

Structure optimisation for 3

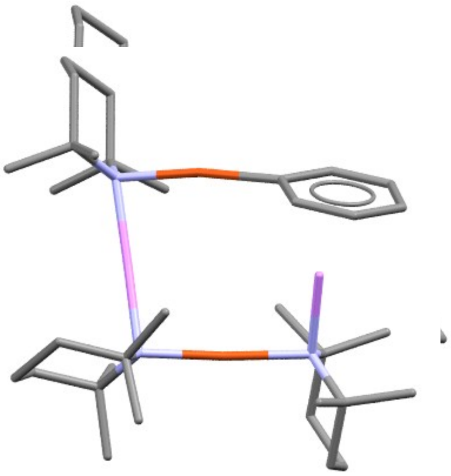

a) ΔG = −2981404.318

(ΔE = −2981901.61) kcal.mol^{-1}

b) ΔG = −2981401.793

(ΔE = −2981898.945) kcal.mol^{-1}

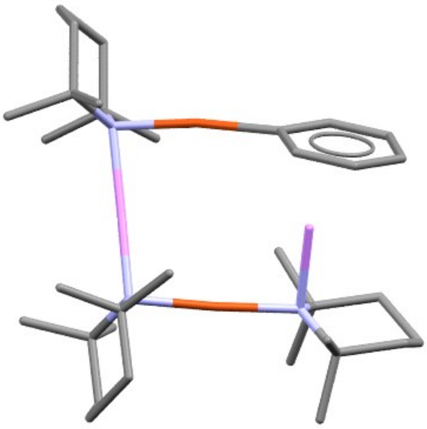

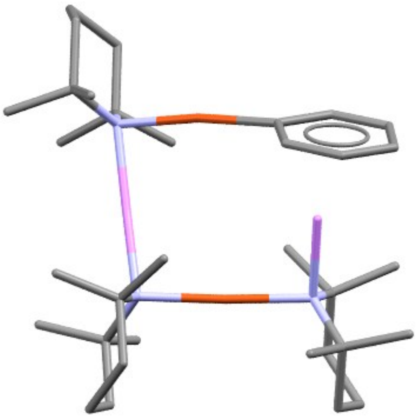

c) ΔG = −2981402.107

(ΔE = −2981898.932) kcal.mol^{-1}

d) ΔG = −2981399.815

(ΔE = −2981896.846) kcal.mol^{-1}

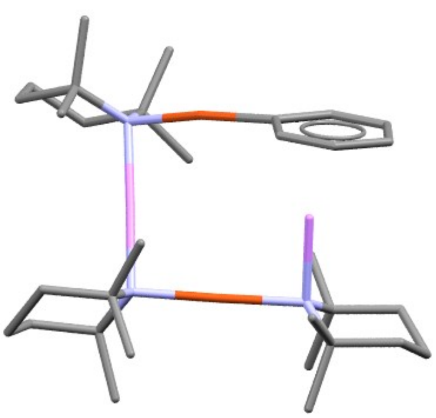

e) ΔG = −2981405.177 (ΔE = −2981901.73) kcal.mol^{-1}

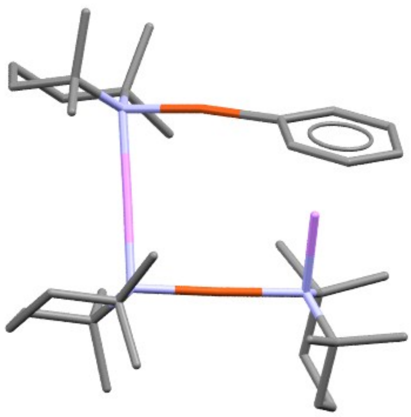

f) ΔG = −2981401.234 (ΔE = −2981898.267) kcal.mol^{-1}

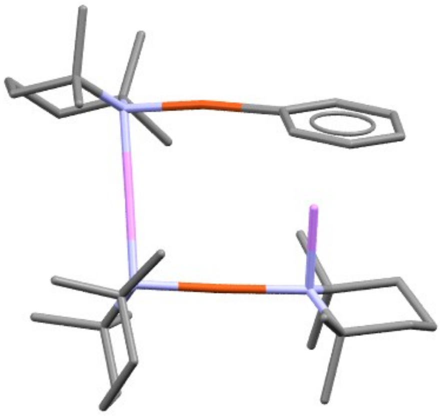

g) ΔG = −2981402.123 (ΔE = −2981899.13) kcal.mol^{-1}

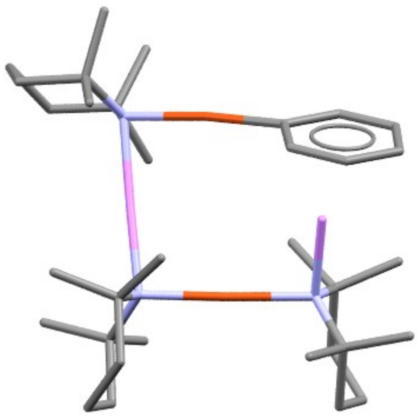

h) ΔG = −2981399.696 (ΔE = −2981897.131) kcal.mol^{-1}

Figure S10 Optimised DFT molecular structures for compound **3**.

Structure optimisation for 4

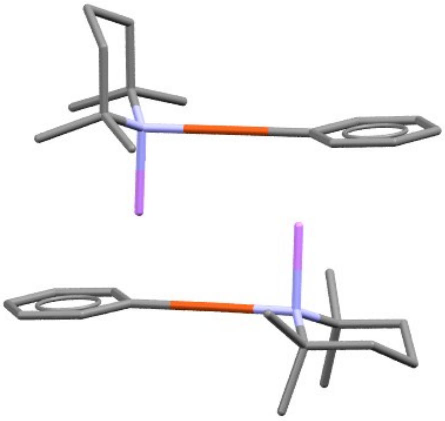

a) ΔG = −2870563.837

(ΔE = −2870955.133) kcal.mol^{-1}

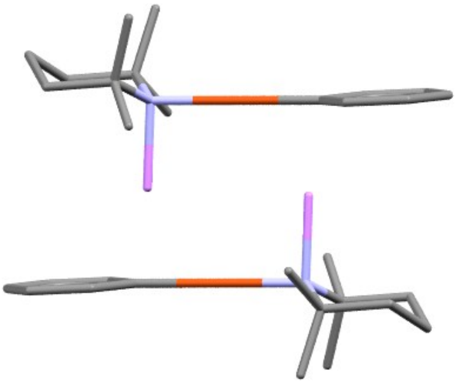

b) ΔG = −2870562.799

(ΔE = −2870952.95) kcal.mol^{-1}

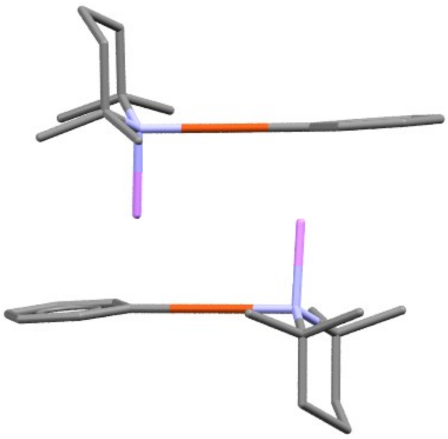

c) $\Delta G = -2870559.828$

($\Delta E = -2870951.038$) kcal.mol^{-1}

Figure S11 Optimised DFT molecular structures for **4**.

Structure optimisation for 5

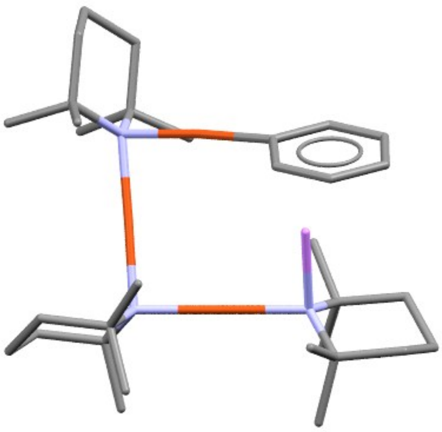

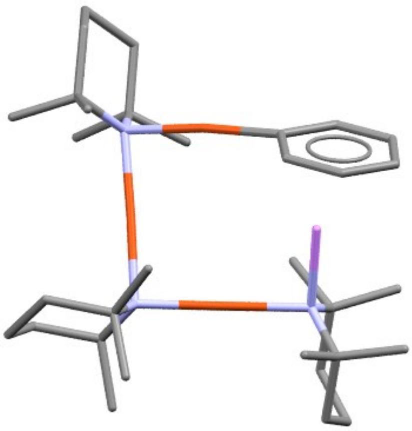

a) $\Delta G = -4005959.452$
($\Delta E = -4006456.917$) kcal.mol^{-1}

b) $\Delta G = -4005956.506$
($\Delta E = -4006454.084$) kcal.mol^{-1}

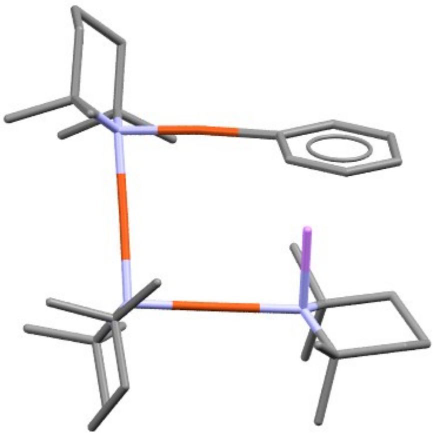

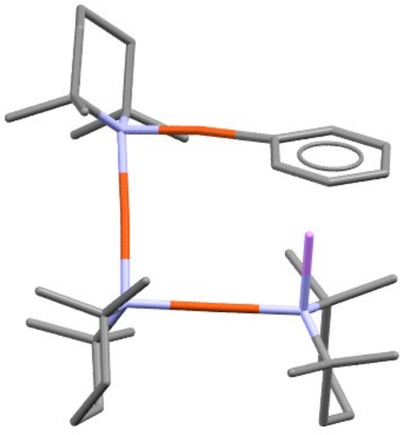

c) ΔG = −4005960.181 (ΔE = −4006456.712) kcal.mol-1

d) ΔG = −4005956.953 (ΔE = −4006454.234) kcal.mol-1

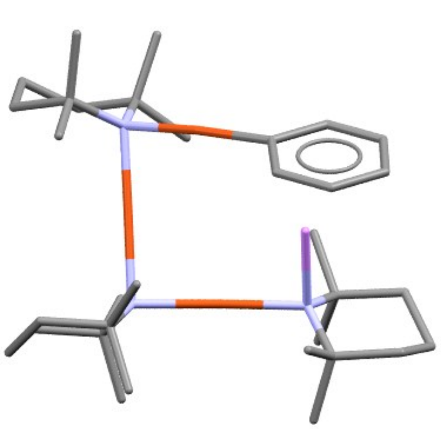

e) ΔG = −4005957.071 (ΔE = −4006455.214) kcal.mol-1

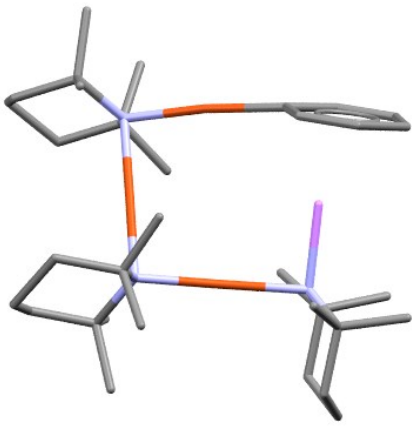

f) ΔG = −4005953.908 (ΔE = −4006451.922) kcal.mol-1

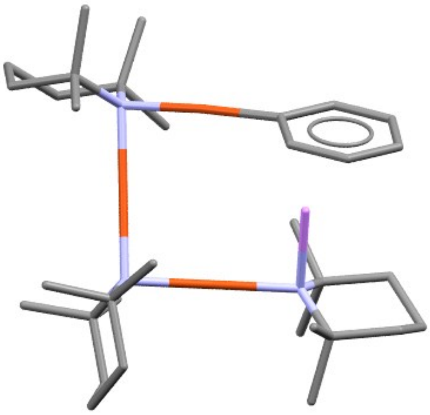

g) $\Delta G = -4005957.471$

($\Delta E = -4006455.071$) kcal.mol^{-1}

h) $\Delta G = -4005954.173$

($\Delta E = -4006452.146$) kcal.mol^{-1}

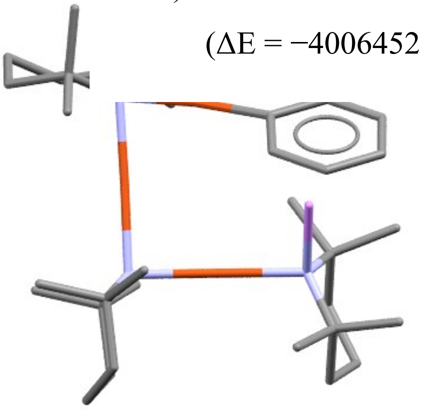

Figure S12 Optimised DFT structures for **5**.

Structure optimisation for 7

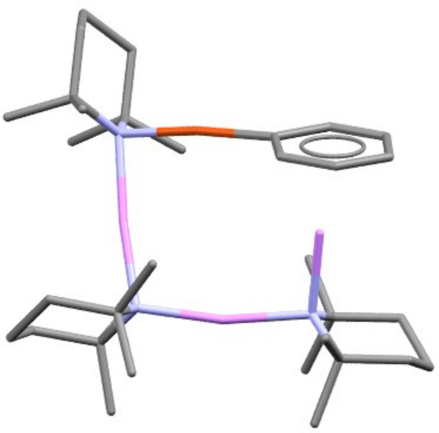

a) $\Delta G = -1956844.943$ ($\Delta E = -1957341.434$) kcal.mol^{-1}

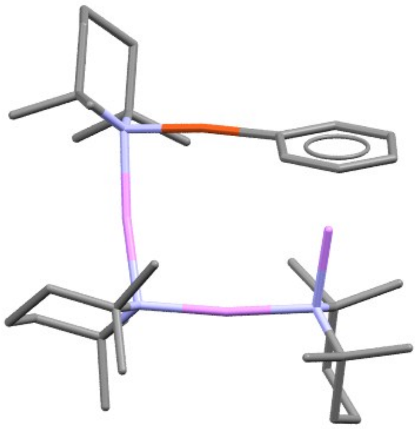

b) $\Delta G = -1956844.752$ ($\Delta E = -1957341.321$) kcal.mol^{-1}

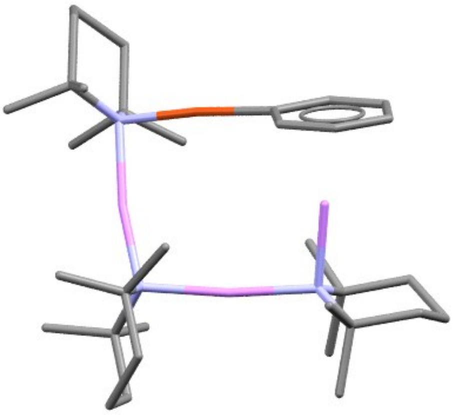

c) $\Delta G = -1956845.05$ ($\Delta E = -1957341.157$) kcal.mol^{-1}

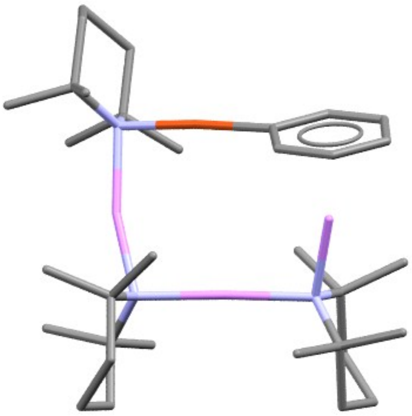

d) $\Delta G = -1956846.453$ ($\Delta E = -1957341.731$) kcal.mol^{-1}

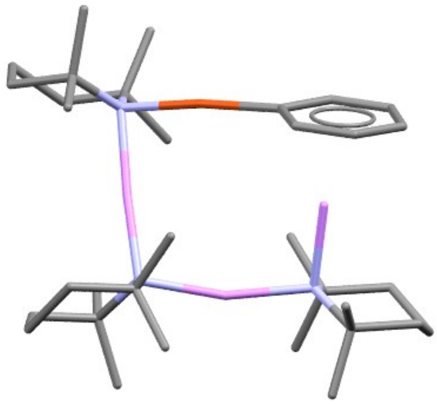

e) ΔG = −1956845.618

(ΔE = −1957341.988) kcal.mol^{-1}

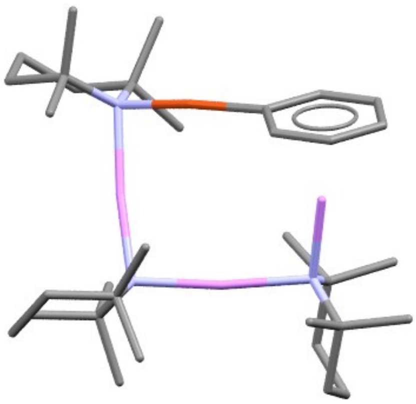

f) ΔG = −1956844.613

(ΔE = −1957341.015) kcal.mol^{-1}

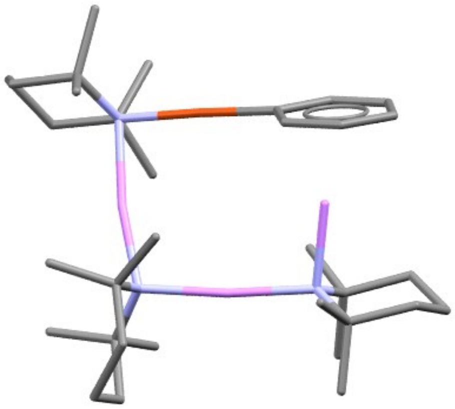

g) ΔG = −1956844.796

(ΔE = −1957340.762) kcal.mol^{-1}

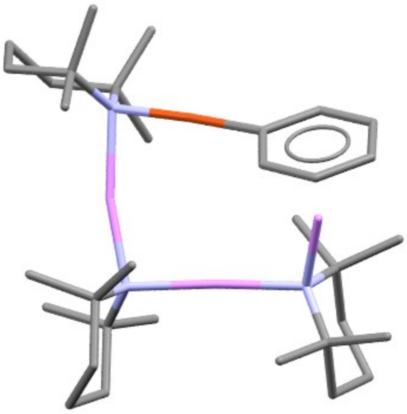

h) ΔG = −1956844.493

(ΔE = −1957342.161) kcal.mol^{-1}

Figure S13 Optimised DFT molecular structures for **7**.

Structure optimization for 1

Cartesian Coordinates

Cu	1.15735600	-1.40851300	-0.01417400
Cu	-1.54245500	-0.92731000	0.04196600
N	2.74640700	-0.37312900	-0.44572900
N	-0.39905200	-2.49677700	0.43749400
C	3.01830300	-0.26236200	-1.90401300
N	-2.65729000	0.61709200	-0.42059100
C	3.90414500	-0.76637300	0.40151000
N	0.55465700	2.84413200	0.39641300
C	-0.62224500	-3.61146700	-0.54063300
C	-0.47724800	-2.89920200	1.87963800
C	-3.71468600	0.94982500	0.57186500
C	-1.93364700	-4.34815100	-0.22348100
H	-2.04800500	-5.19817100	-0.91604800
H	-2.77305800	-3.65906800	-0.42642900
C	-1.79270200	-3.64298200	2.15891200
H	-2.62508300	-2.92483900	2.05095900
H	-1.80008000	-3.97937800	3.20879800
C	1.82182000	0.43807800	-2.54755000
H	1.67313400	1.45812300	-2.15295000
H	0.89222700	-0.12842700	-2.38864000
H	1.96405500	0.55128500	-3.63174400
C	-3.11534300	0.53183200	-1.83605600
C	4.25470000	0.61613000	-2.16391700
H	4.46704000	0.63794700	-3.24546700
H	4.01369200	1.65578200	-1.87224800
C	-0.75710000	-3.02714500	-1.94317300
H	-1.61458800	-2.34721200	-2.01568700
H	-0.91441200	-3.83074600	-2.67656500
H	0.14647600	-2.47436100	-2.24302600
C	0.52281900	4.06659500	-0.43060900
C	5.12954100	0.11377500	0.09964300
H	4.91638200	1.14036000	0.45007200
H	5.98863100	-0.24488000	0.68999200
C	-0.46260000	-1.64282500	2.74200600
H	-1.26222100	-0.94879500	2.44984200
H	0.49486800	-1.10930800	2.66241900
H	-0.61481900	-1.90227500	3.79938400
C	-4.54154500	2.16906300	0.12013000
H	-5.35424700	2.34122800	0.84462400
H	-3.88989900	3.06177300	0.16462200
C	3.18135900	-1.60033000	-2.64912800
H	2.35935300	-2.28614500	-2.39874800
H	4.11922400	-2.11574900	-2.41857200
H	3.16432500	-1.43806800	-3.73777400
C	-2.02726400	-4.81442200	1.21958600
H	-1.29634500	-5.61595000	1.41334100
H	-3.01477900	-5.25959500	1.40942400
C	-4.65279800	-0.21966800	0.91791600
H	-4.06405900	-1.10172600	1.21526500
H	-5.29359500	-0.52067400	0.08267000
H	-5.31356100	0.04440200	1.75809300
C	0.63223300	1.76167200	2.55648900
H	-0.45505200	1.61064700	2.58799300
H	0.99560000	1.78584200	3.59475000
H	1.06800200	0.86447800	2.08780400
C	0.98809600	3.03976000	1.79655700
C	4.30805300	-2.25000400	0.29694100
H	4.80714000	-2.50344600	-0.64433900
H	3.42236900	-2.89643800	0.38592500
H	5.00238100	-2.51627800	1.10845900

C	5.47539800	0.16982600	-1.37835600
H	6.31161800	0.86373100	-1.54817400
H	5.82440700	-0.81342200	-1.73254500
C	-0.19593300	5.23008700	0.27959800
H	-1.26924800	4.97284900	0.35300600
H	-0.13261300	6.14090400	-0.33924300
C	-3.04601600	1.36654400	1.88079000
H	-2.39020000	2.24424700	1.74705900
H	-2.45413900	0.55188500	2.31926200
H	-3.80062100	1.65857200	2.62512400
C	0.32703100	5.49162100	1.68146200
H	1.35931000	5.87637200	1.64239300
H	-0.26583900	6.27948100	2.16916300
C	0.70758900	-3.75070500	2.37515100
H	0.65323500	-4.80397100	2.08370100
H	0.74066300	-3.73202300	3.47440400
H	1.66153600	-3.34931000	2.00584500
C	-3.93691600	1.76927900	-2.23941800
H	-3.26826300	2.64834500	-2.25605700
H	-4.30195700	1.63553600	-3.27109700
C	3.53312900	-0.53073600	1.86484400
H	3.26254600	0.51786900	2.05296400
H	4.38242100	-0.76903400	2.52141900
H	2.68820200	-1.16377700	2.17664600
C	-3.94545500	-0.72359000	-2.18409100
H	-3.53670800	-1.62051600	-1.69940600
H	-3.94260100	-0.89565000	-3.27139600
H	-4.99593800	-0.64422500	-1.88138600
C	-1.87805000	0.51080200	-2.73183100
H	-1.24249400	1.38976900	-2.55697100
H	-2.16537300	0.52273700	-3.79341400
H	-1.26656900	-0.38563900	-2.56576400
C	1.90584200	4.53968200	-0.92541200
H	2.55321900	4.90552900	-0.12149900
H	1.80704700	5.35641800	-1.65763700
H	2.43562000	3.71288900	-1.42613000
C	-5.08685400	2.04963700	-1.28967400
H	-5.84462500	1.25187400	-1.34562000
H	-5.60483900	2.97630300	-1.57753200
C	2.51275100	3.26143700	1.97963100
H	3.11673100	2.58658900	1.34942900
H	2.81363700	3.08017100	3.02307300
H	2.83006000	4.28001500	1.73028300
C	-0.28351400	3.76562600	-1.69416400
H	-1.32581800	3.48581600	-1.45958100
H	0.17393000	2.95509600	-2.28125000
H	-0.34441000	4.64787300	-2.34767500
C	0.26126100	4.20596900	2.48939200
H	-0.79933800	3.92619500	2.61923900
H	0.67441100	4.35412800	3.50165600
C	0.53960100	-4.61829600	-0.62303400
H	1.50039400	-4.09358100	-0.72859200
H	0.41088100	-5.26139400	-1.50589400
H	0.61760700	-5.28334100	0.24126000
Li	-1.08089200	1.80960500	-0.01748000
Li	1.78266600	1.32709200	-0.01234600

Structure optimization for 2

Cartesian Coordinates

C	3.85513200	-1.98789400	2.25497500
C	2.37050000	-2.10600700	1.86908600
C	3.04833600	-2.37081700	-0.53972800
C	4.52220600	-2.24508200	-0.11394300
C	4.77842100	-2.72655400	1.30257900
H	4.13248300	-0.91854700	2.25714200
H	3.98942800	-2.34637800	3.28870900
H	5.15610000	-2.78670700	-0.83526800
H	4.81201400	-1.17951700	-0.17923400
H	4.62156700	-3.81474900	1.37604900
H	5.82922900	-2.55549000	1.57886800
N	2.15135100	-1.71567500	0.44951300
C	1.86770400	-3.51387100	2.24503300
H	1.86942400	-3.63894100	3.33883900
H	0.83629900	-3.66370900	1.89350900
H	2.47668600	-4.32408400	1.83101400
C	1.59616600	-1.13530600	2.75550600
H	0.51282500	-1.22277300	2.59547200
H	1.79195800	-1.33711100	3.81876600
H	1.88359900	-0.09301100	2.56287200
C	2.72592300	-3.84778400	-0.83752900
H	1.64832000	-3.97649400	-1.01892800
H	3.26358000	-4.18696500	-1.73625300
H	3.00443300	-4.52718100	-0.02512600
C	2.91245200	-1.62952900	-1.86809100
H	3.58505000	-2.05593200	-2.62584700
H	1.88712600	-1.69238500	-2.26369000
H	3.18947500	-0.56550600	-1.77387000
C	-3.37128400	-2.03646800	-2.28033400
C	-1.86936900	-2.11234200	-1.96160500
C	-2.43483400	-2.73040600	0.42011400
C	-3.92833800	-2.65016100	0.06592200
C	-4.21712200	-2.93878900	-1.39758100
H	-3.70548700	-0.99334300	-2.14191900
H	-3.52671900	-2.27751000	-3.34474000
H	-4.48937700	-3.34167100	0.71609400
H	-4.28281500	-1.63146400	0.30608000
H	-4.01938800	-3.99794300	-1.62816500
H	-5.28482800	-2.78254500	-1.60989400
N	-1.62869900	-1.86378500	-0.50211300
C	-1.30042100	-3.44480800	-2.48304200
H	-1.30524400	-3.44093900	-3.58301300
H	-0.25905700	-3.57633300	-2.15575800
H	-1.86269200	-4.32840700	-2.16918200
C	-1.16380900	-1.03234400	-2.77311200
H	-0.07238100	-1.09349800	-2.65674000
H	-1.39174500	-1.14816600	-3.84251700
H	-1.47693800	-0.02553900	-2.47310300
C	-1.98709500	-4.20520100	0.45574200
H	-0.89012100	-4.28100500	0.46594400
H	-2.36597200	-4.68788300	1.36837600
H	-2.35346500	-4.80133300	-0.38569800
C	-2.28494400	-2.21893100	1.84935800
H	-2.91216000	-2.81136800	2.53051100
H	-1.24670900	-2.29749600	2.20405200
H	-2.60506500	-1.17127100	1.93611600
C	-3.32425100	1.82535100	2.51070500
C	-1.94527100	2.18359700	1.93443300
C	-2.98630900	2.63779600	-0.29861300
C	-4.36393500	2.27504200	0.28200900

C	-4.46255000	2.52303800	1.78003000
H	-3.46510900	0.73368900	2.42521400
H	-3.35092900	2.06320500	3.58767500
H	-5.15147700	2.83155000	-0.25358900
H	-4.54373900	1.20239600	0.08501500
H	-4.44539000	3.60505700	1.98917400
H	-5.43025000	2.16392200	2.15993500
N	-1.89133800	1.96931300	0.45902500
C	-1.56643600	3.61280400	2.38386200
H	-1.28960800	3.60981300	3.44835700
H	-0.69810000	3.99302300	1.82162800
H	-2.37052600	4.34617000	2.27268100
C	-0.89808400	1.28047100	2.57890400
H	0.12332200	1.55939600	2.26607400
H	-0.92582100	1.35872300	3.67557300
H	-1.04736600	0.22755300	2.31269000
C	-2.84992600	4.17205200	-0.38113700
H	-1.82615100	4.45980700	-0.66459000
H	-3.52695500	4.56798800	-1.15216600
H	-3.09462300	4.69571300	0.54715500
C	-2.95315000	2.15885100	-1.74719000
H	-3.74347300	2.64931300	-2.33335800
H	-1.99038600	2.39241900	-2.22933600
H	-3.12175600	1.07533100	-1.81670300
C	3.24839200	2.75026100	-2.48796200
C	1.86636000	2.65136200	-1.81997300
C	2.93097700	2.93668400	0.42199600
C	4.30651800	3.03588500	-0.26409400
C	4.22619700	3.58580400	-1.67825500
H	3.65746400	1.72892500	-2.58865300
H	3.14211400	3.14903400	-3.51102500
H	4.99037800	3.64129300	0.35433100
H	4.73969300	2.01915300	-0.31089500
H	3.91404900	4.64306100	-1.66423400
H	5.22011200	3.57434900	-2.14948900
N	1.94693600	2.21393100	-0.40841700
C	1.12041700	3.98959900	-2.04595900
H	0.90069500	4.13750100	-3.11452200
H	0.14988800	4.01337500	-1.51975500
H	1.68303200	4.86660800	-1.70988600
C	1.06093000	1.59642500	-2.57403800
H	0.05659800	1.46146300	-2.13998400
H	0.91789000	1.86835100	-3.63050300
H	1.56026400	0.61874400	-2.54871500
C	2.48468700	4.34251800	0.87618000
H	1.48254800	4.29632900	1.33282000
H	3.17116400	4.74881700	1.63538000
H	2.44274300	5.07269100	0.06129300
C	3.13385800	2.13997200	1.70971600
H	3.86156200	2.62909400	2.37318900
H	2.19155400	2.04396700	2.27206000
H	3.53138100	1.12941900	1.51042900
Li	2.10712200	0.27189600	0.01500400
Cu	0.26639700	-1.78634200	-0.03259000
Cu	-1.76464000	0.05662700	-0.01353100
Li	0.01247800	2.30805900	0.00996900

Structure optimization for 3

3-a)

Cartesian Coordinates

C -4.13088600 -0.52598900 -0.60288600

C -3.26798600 -0.92743500 1.72606100

C -4.14773300 -2.18433900 1.82724400

C -5.39301800 -2.10124400 0.95780000

C -5.00940200 -1.78242900 -0.47900200

H -3.54470300 -3.05091400 1.50179000

H -4.42053400 -2.36352100 2.88111400

H -5.94731700 -3.05041600 0.99939000

H -6.08382500 -1.33619400 1.34867800

H -4.44246700 -2.63536300 -0.89320500

H -5.91108100 -1.66688300 -1.10383800

N -2.96035600 -0.59003300 0.31100900

Cu -1.57520500 -1.76803400 -0.39667300

C -0.23830600 2.56130200 -1.68330300

C -0.36060000 3.24410000 0.74267400

C -1.67228200 3.99389800 0.44676600

C -1.77730800 4.47055400 -0.99316700

C -1.55921100 3.30580600 -1.94676200

H -2.51958500 3.31158000 0.65329200

H -1.78200000 4.83815800 1.14703400

H -2.76280300 4.92596700 -1.16979500

H -1.04040100 5.26657100 -1.18626700

H -2.39790700 2.59254600 -1.83398200

H -1.58691500 3.64888500 -2.99415200

N -0.14544700 2.16975400 -0.25562500

C 3.07356800 -0.49311300 1.84344200

C 4.06617500 0.19968000 -0.37353500

C 5.18135200 -0.84155200 -0.17331700

C 5.47978500 -1.12202500 1.28980500

C 4.20019600 -1.52826400 2.00208100

H 4.86609800 -1.78620400 -0.65503500

H 6.08987600 -0.50979000 -0.70234600

H 6.23419200 -1.91756800 1.37839600

H 5.92242800 -0.23418900 1.76930200

H 3.85206400 -2.49030500 1.58163700

H 4.38676700 -1.70523300 3.07407900

N 2.86015800 -0.16502800 0.41172500

Cu 1.36878500 1.03765000 0.11456100

Li 1.85675000 -1.57867500 -0.48345200

C 2.39459900 -3.93721500 -1.94970100

C 1.85736300 -4.25845400 -0.70181300

C 0.65670900 -3.67339200 -0.28461600

C -0.06102700 -2.74216400 -1.07092500

C 0.50959100 -2.46649900 -2.33520800

C 1.70464300 -3.04641000 -2.77381700

H 3.33764000 -4.37491900 -2.27527700

H 2.38115400 -4.95668600 -0.04751600

H 0.27587500 -3.94323200 0.70430400

H 0.01414700 -1.75351800 -3.00064000

H 2.10896800 -2.78841800 -3.75367300

C 3.68860600 0.17721000 -1.85596900

H 3.27404400 -0.79781000 -2.16436500

H 2.94988100 0.95570200 -2.09782600

H 4.57330800 0.35508600 -2.48452000

C 4.61924900 1.60896700 -0.09428600

H 5.11103600 1.69716900 0.88042500

H 5.36277400 1.88943300 -0.85623300

H 3.80720200 2.35089100 -0.12427500

C 3.36295100 0.71646700 2.75155700

H 3.27930400 0.43033000 3.81127600

H 4.36536400 1.13539800 2.61225000

H 2.64005800 1.52337200 2.56098500

C 1.78670400 -1.12707800 2.37085900

H 0.94576300 -0.41850100 2.32640700

H 1.50096400 -2.02465900 1.79723300

H 1.90372100 -1.44230500 3.41789900

C 0.79694400 4.24891200 0.88016800

H 0.90061700 4.91411200 0.01640800

H 0.64981800 4.88808400 1.76433600

H 1.75071000 3.71399100 1.00425100

C -0.51211900 2.58235000 2.11274900

H 0.40707500 2.05410600 2.40956900

H -0.73430500 3.33010400 2.88771900

H -1.34106700 1.85548200 2.13399900

C 0.93059000 3.41255100 -2.21710600

H 0.92940900 3.41190600 -3.31815200

H 0.89037800 4.46056300 -1.90057400

H 1.89327800 3.00231900 -1.87934300

C -0.23731900 1.27903700 -2.52049600

H 0.75431800 0.80159100 -2.52215400

H -0.95532400 0.53120900 -2.14791300

H -0.50416700 1.49598000 -3.56589200

C -3.62593100 -0.45860800 -2.04512500

H -3.08226000 0.47907700 -2.23782700

H -2.95531900 -1.29952400 -2.28728600

H -4.47019400 -0.49312400 -2.74918400

C -4.99606900 0.73588000 -0.41695700

H -4.36193800 1.63540600 -0.37018000

H -5.68119200 0.85598800 -1.26962900

H -5.61464900 0.72057500 0.48606200

C -3.92734700 0.22143200 2.51596100

H -4.98550500 0.36836000 2.27753300

H -3.87149100 0.02210800 3.59653100

H -3.41394200 1.17617300 2.32629600

C -1.95080300 -1.22233700 2.44564300

H -1.41034100 -2.06194200 1.97978200

H -1.28047800 -0.34734300 2.44001800

H -2.13143900 -1.48432900 3.49842100

Li -1.54657000 0.79712800 0.10586800

3-b)

Cartesian Coordinates

C -4.13431000 -0.35482200 -0.58531900

C -3.29138600 -0.72716600 1.75873000

C -4.29644600 -1.87847300 1.92527800

C -5.53679400 -1.70635200 1.06258600

C -5.13417700 -1.50711400 -0.38996400

H -3.79136700 -2.81711300 1.63523800

H -4.57166300 -1.97982900 2.98875500

H -6.18933100 -2.58672000 1.15744200

H -6.13639500 -0.85149000 1.41631000

H -4.65933300 -2.43470000 -0.75655700

H -6.02341200 -1.33490600 -1.01951900

N -2.96853500 -0.48302200 0.32826200

Cu -1.66614600 -1.76596100 -0.34103400

C -0.13724100 2.47336200 -1.75469100

C -0.16013600 3.22370300 0.64535000

C -1.48746900 3.96192200 0.38712000

C -1.67190400 4.38626100 -1.06140500

C -1.47471400 3.19822000 -1.99079600

H -2.32103200 3.28848100 0.66661100

H -1.56312000 4.83266000 1.05898600

H -2.67495600 4.81532400 -1.20372100

H -0.96385100 5.19157700 -1.31438500

H -2.30200900 2.48085200 -1.83184700

H -1.54326900 3.51387500 -3.04514600

N 0.00201800 2.11310100 -0.32236500

C 4.10603100 -0.07012700 -0.38937100

C 3.03352800 -0.68914400 1.79956600

C 3.74820200 0.49065000 2.47797700

C 5.04838100 0.87010100 1.78064400

C 4.81742900 1.10820900 0.29383800

H 3.06853500 1.36165000 2.46008200

H 3.93419700 0.25625100 3.53981800

H 5.47405000 1.77206200 2.24428700

H 5.80233600 0.07906700 1.92566800

H 4.18746600 2.00753300 0.16769500

H 5.77332400 1.31683000 -0.21584900

N 2.86526700 -0.44136500 0.34210700

Cu 1.45385200 0.88854800 0.03396000

Li 1.84150500 -1.81448500 -0.54533000

C 2.21785300 -4.10350900 -1.86267300

C 1.67933000 -4.38203900 -0.60480400

C 0.50018200 -3.75102000 -0.19097200

C -0.19998600 -2.82223800 -0.99606700

C 0.36728600 -2.59507600 -2.27227200

C 1.54703600 -3.21182900 -2.70336700

H 3.14709000 -4.57328700 -2.18295100

H 2.18947300 -5.07801600 0.06243600

H 0.12617400 -3.97969600 0.81073500

H -0.11530000 -1.88791200 -2.95286800

H 1.95283500 -2.98603500 -3.69042400

C 1.64371900 -0.84234500 2.41591000

H 1.02744300 0.05279100 2.25209900

H 1.10514100 -1.70182300 1.98195200

H 1.70972200 -1.00833700 3.50116500

C 3.76834000 -2.00224600 2.14023700

H 3.61108600 -2.25479700 3.19923100

H 3.37646500 -2.83872300 1.54047000

H 4.85058200 -1.96195100 1.98599500

C 5.09925000 -1.23513700 -0.58158200

H 5.84195400 -0.97186900 -1.34914400

H 5.66090600 -1.49743400 0.31984400

H 4.57814100 -2.14316800 -0.92459700

C 3.71428100 0.35479800 -1.80402900

H 3.22383500 -0.46956300 -2.35063100

H 3.02490400 1.21041700 -1.79376700

H 4.60123100 0.64839100 -2.38374600

C 1.00249800 4.23131200 0.68657600

H 0.90668600 4.89144900 1.56241400

H 1.96065900 3.69627600 0.76883100

H 1.05605100 4.87542300 -0.19700200

C -0.23984500 2.62730500 2.05065100

H 0.72287200 2.19475900 2.35969200

H -0.50712000 3.40077600 2.78539300

H -1.00596400 1.83810200 2.13015900

C 0.99864400 3.33335300 -2.34727800

H 1.01249100 3.23984500 -3.44407900

H	0.89838200	4.40159100	-2.12468800
H	1.97647100	3.00832300	-1.96629800
C	-0.14834000	1.17245700	-2.56246800
H	0.85290500	0.71784200	-2.60196300
H	-0.83082600	0.41878200	-2.13674900
H	-0.47081400	1.35960100	-3.59805700
C	-3.63763600	-0.42340900	-2.03083300
H	-2.98945600	0.43079800	-2.27836900
H	-3.07218700	-1.34928200	-2.22659800
H	-4.48689600	-0.39868300	-2.72940700
C	-4.86950200	0.99487000	-0.46658300
H	-5.55170000	1.13354900	-1.31881700
H	-5.47372600	1.09039800	0.44140700
H	-4.15213200	1.83110100	-0.47631900
C	-3.81515900	0.51903300	2.50049800
H	-4.84587800	0.78232500	2.24250100
H	-3.79232900	0.35442000	3.58813800
H	-3.18944900	1.39882800	2.28495200
C	-2.00470800	-1.13198400	2.48002100
H	-1.57539900	-2.05353200	2.05529400
H	-1.23424800	-0.34604500	2.41898200
H	-2.19717200	-1.31430200	3.54748800
Li	-1.46524700	0.80850700	0.08516500

3-c)

Cartesian Coordinates

C	-4.15773700	-0.34530500	-0.68452800
C	-3.39556800	-0.72702500	1.68766100
C	-4.41655400	-1.86857600	1.81772500
C	-5.62331900	-1.68605600	0.90982400
C	-5.16849600	-1.49311700	-0.52898300
H	-3.90906100	-2.81104000	1.54496500
H	-4.73168600	-1.96875500	2.87010100
H	-6.28703000	-2.56009500	0.98290300
H	-6.22663200	-0.82496000	1.24157400
H	-4.68446400	-2.42342200	-0.87577700
H	-6.03377400	-1.31792800	-1.19036300
N	-3.02897400	-0.48258800	0.27043500
Cu	-1.68934300	-1.73957500	-0.37155500
C	-0.22654300	3.27065100	0.74722800
C	-0.15795400	2.61316300	-1.67901700
C	0.97239500	3.62640000	-1.92125500
C	0.93754500	4.78854600	-0.93554600
C	0.89995100	4.28742300	0.50317100
H	1.93802300	3.09999600	-1.81648100
H	0.92703200	4.00078800	-2.95813700
H	1.81564200	5.43370600	-1.08536900
H	0.06326100	5.42953800	-1.13555200
H	1.86099200	3.79416400	0.73734400
H	0.80122100	5.13503000	1.20235600
N	-0.18260400	2.17561600	-0.25753600
C	2.93069500	-0.65412000	1.85874000
C	4.01591500	-0.04162800	-0.32944600
C	4.98409300	-1.22703400	-0.15797800
C	5.22756200	-1.60056400	1.29485300
C	3.90098200	-1.84028300	1.99719800

H	4.55491200	-2.10314900	-0.68102500
H	5.93556400	-0.99896000	-0.66605600
H	5.85876200	-2.49938200	1.35519500
H	5.79296400	-0.80366800	1.80401800
H	3.42274100	-2.73745900	1.56004400
H	4.05908700	-2.06206800	3.06562400
N	2.76416300	-0.28383800	0.42998000
Cu	1.32757800	0.98245600	0.10544500
Li	1.71699000	-1.66228600	-0.47399500
C	2.19208600	-4.06282400	-1.92425100
C	1.65467800	-4.35915100	-0.67038800
C	0.47904300	-3.73068200	-0.24517400
C	-0.21496000	-2.78179000	-1.03164100
C	0.35357400	-2.53317200	-2.30293800
C	1.52610500	-3.15340200	-2.74758500
H	3.11722600	-4.53373400	-2.25484700
H	2.16027900	-5.07129400	-0.01672800
H	0.09969600	-3.97963100	0.74985800
H	-0.12410600	-1.80929300	-2.96965700
H	1.93080200	-2.91392100	-3.73195500
C	3.67342900	0.04522700	-1.81761500
H	3.08827900	-0.82204500	-2.16887100
H	3.10485300	0.95697900	-2.04897800
H	4.59246400	0.07005100	-2.42116000
C	4.73642700	1.27519800	0.01017700
H	5.22279300	1.26668600	0.99113400
H	5.51799600	1.48839200	-0.73530400
H	4.02088800	2.11101000	-0.00037600
C	3.39725400	0.48471100	2.78729700
H	3.18523600	0.23210400	3.83736600
H	4.47216200	0.68616600	2.72239200
H	2.87374000	1.42184400	2.55221000
C	1.56938000	-1.10767200	2.38578400
H	1.65687300	-1.49460000	3.41159800
H	0.85095300	-0.27484600	2.40437800
H	1.13156100	-1.91488400	1.77530500
C	-1.48926900	3.20580300	-2.18828200
H	-1.67287400	4.23435500	-1.86338700
H	-1.49798300	3.21822600	-3.28817200
H	-2.34775300	2.59830100	-1.86210600
C	0.08606500	1.37865800	-2.54909600
H	-0.72074200	0.63638400	-2.42559800
H	0.12521000	1.65120800	-3.61387000
H	1.03147300	0.87946400	-2.29613700
C	-1.58242900	4.00215200	0.82470200
H	-1.63405900	4.60182600	1.74539800
H	-1.76916200	4.68702500	-0.00732700
H	-2.41786500	3.28383100	0.85580700
C	-0.03183000	2.65092300	2.13061100
H	-0.83603300	1.93219900	2.36691800
H	0.92732600	2.11957300	2.20752200
H	-0.04755900	3.42397200	2.91231100
C	-3.59600100	-0.40691400	-2.10636400
H	-2.90771500	0.43086600	-2.30392600
H	-3.05065300	-1.34668500	-2.29070800
H	-4.40692800	-0.34122400	-2.84653700
C	-4.89332100	1.00637100	-0.58773200
H	-4.17525000	1.84065200	-0.54545600
H	-5.52818800	1.16010300	-1.47318000
H	-5.54646500	1.09137900	0.28703900
C	-3.91964300	0.52657900	2.41523700
H	-4.92003500	0.83563900	2.09671500
H	-3.97059300	0.34747600	3.49971300
H	-3.24391100	1.38230300	2.25347000
C	-2.13270200	-1.14113300	2.44353700
H	-1.69418000	-2.06343500	2.02979500

H -1.36131300 -0.35428000 2.39939800

H -2.35338800 -1.32137100 3.50591700

Li -1.58453200 0.84724600 0.06937700

3-d)

Cartesian Coordinates

C -4.21072800 -0.10523000 -0.58984000

C -3.37843700 -0.46248500 1.76084800

C -4.43666500 -1.55924800 1.95835100

C -5.66910000 -1.34811600 1.09229000

C -5.26284100 -1.20457700 -0.36657300

H -3.97717900 -2.52732600 1.69023800

H -4.71469400 -1.62213000 3.02398900

H -6.36368800 -2.19281600 1.20999200

H -6.22459400 -0.45670100 1.42728400

H -4.83411200 -2.16257200 -0.71082400

H -6.14537500 -1.00561200 -0.99780400

N -3.05169400 -0.26948400 0.32537400

Cu -1.82188900 -1.62288300 -0.34715700

C 0.16106100 3.28215300 0.65016600

C -0.07274800 2.48368000 -1.72122900

C 1.04364300 3.44723800 -2.15502900

C 1.15505800 4.66456000 -1.24699400

C 1.28501000 4.23265800 0.20666600

H 2.00304700 2.90312700 -2.13565900

H 0.87998500 3.75751100 -3.20103200

H 2.02357800 5.27286300 -1.53919200

H 0.27841500 5.32078700 -1.37301400

H 2.24656000 3.70309400 0.33545600

H 1.31535400 5.11277300 0.87094100

N 0.03202000 2.12890900 -0.27860900

C 4.04831500 -0.30895100 -0.29386200

C 2.83359900 -1.04238600 1.78406100

C 3.66912500 -0.05311900 2.61194400

C 5.02911800 0.23482100 1.99290200

C 4.86406600 0.68423700 0.54871500

H 3.10912300 0.89482100 2.68995300

H 3.78302000 -0.43548100 3.64029400

H 5.55057400 1.01033100 2.57291400

H 5.67310900 -0.65836700 2.04502500

H 4.33502800 1.65452900 0.53712700

H 5.84779100 0.85239700 0.07894900

N 2.74878200 -0.62963900 0.35459800

Cu 1.41582100 0.78370700 0.05857100

Li 1.66246600 -1.89899000 -0.62611800

C 1.84902400 -4.18465400 -2.03728200

C 1.31657900 -4.47433700 -0.77961000

C 0.19893000 -3.77352600 -0.31144900

C -0.44635000 -2.76282300 -1.06112600

C 0.10731300 -2.52974800 -2.34270500

C 1.22909000 -3.21224200 -2.82597800

H 2.73332700 -4.70886000 -2.39780300

H 1.78604500 -5.23409400 -0.15324400

H -0.16768200 -4.01242600 0.69076600

H -0.33696600 -1.76506500 -2.98593500

H 1.62821700 -2.97762400 -3.81364500

C	1.41677500	-1.06765600	2.35343800
H	0.94091600	-0.07928000	2.28903300
H	0.77562100	-1.78242800	1.81107600
H	1.42163500	-1.36740300	3.41189100
C	3.38417000	-2.46889500	1.99679600
H	4.46744500	-2.55120300	1.86598100
H	3.16409900	-2.80715200	3.02011100
H	2.91120200	-3.18566000	1.30683200
C	4.90802100	-1.54928900	-0.61351000
H	5.73179900	-1.27487100	-1.28902600
H	5.36347900	-2.01576500	0.26487000
H	4.30802000	-2.32018100	-1.12412000
C	3.76669100	0.34222800	-1.64612800
H	3.18864500	-0.32822200	-2.30461600
H	3.20047100	1.27789600	-1.52988400
H	4.70546800	0.58303200	-2.16568700
C	-1.43385100	3.09467000	-2.12448700
H	-1.57903500	3.01264600	-3.21184700
H	-2.27475700	2.57000100	-1.64527200
H	-1.53127800	4.15632000	-1.87623900
C	0.05462100	1.19322800	-2.53276100
H	-0.72633400	0.46471000	-2.25414800
H	-0.04796200	1.39377000	-3.60945800
H	1.02827700	0.70872900	-2.37322200
C	-1.14871900	4.07365000	0.84091900
H	-1.05457400	4.76614100	1.69040400
H	-1.43873300	4.67372000	-0.02623300
H	-1.98781000	3.39460700	1.06611300
C	0.51406300	2.74979800	2.03768300
H	-0.25823600	2.05656700	2.41232800
H	1.47690500	2.21711300	2.03178300
H	0.59488600	3.57315100	2.76214700
C	-3.71156300	-0.22884600	-2.03147200
H	-2.99116000	0.56580800	-2.28022300
H	-3.22276300	-1.19996300	-2.21307300
H	-4.54998900	-0.14287400	-2.73821200
C	-4.88664700	1.27770000	-0.49768700
H	-4.13461400	2.08132800	-0.47265600
H	-5.52770300	1.44869800	-1.37540600
H	-5.52440100	1.39754300	0.38462900
C	-3.82808000	0.82869400	2.47203700
H	-4.81960200	1.17823300	2.16886400
H	-3.86114000	0.67469400	3.56106500
H	-3.11713400	1.64817800	2.27716800
C	-2.10630500	-0.90385100	2.48322800
H	-1.71611200	-1.85064300	2.07692700
H	-1.30732300	-0.14757700	2.39516200
H	-2.29456600	-1.04984700	3.55704800
Li	-1.50370600	0.95519400	0.13165500

3-e)

Cartesian Coordinates

C	-3.17506900	-1.35670300	1.63641800
C	-4.11491600	-0.67164700	-0.59524300
C	-5.14896300	0.20596300	0.12965900
C	-5.49314600	-0.30907000	1.51756500

C -4.22104800 -0.47177400 2.33380000

H -4.72967800 1.22485400 0.22661500

H -6.05594000 0.29633900 -0.49081500

H -6.18228100 0.38518100 2.02071000

H -6.03092100 -1.26852600 1.44837200

H -3.77760600 0.52679600 2.50054100

H -4.44634200 -0.88522500 3.33080600

N -2.91958000 -0.87948500 0.25499400

Cu -1.49507500 -1.88905900 -0.57666900

C -0.19496600 2.62185200 -1.69428900

C -0.56629800 3.18395300 0.73676200

C -1.79192000 4.02033200 0.32857400

C -1.68046100 4.59715800 -1.07199100

C -1.42157900 3.47441900 -2.06334800

H -2.68563500 3.36892000 0.36715200

H -1.95624800 4.81801700 1.07147800

H -2.60402300 5.13237700 -1.33711900

H -0.87553500 5.34830000 -1.11520300

H -2.31185800 2.82332100 -2.09781400

H -1.29110900 3.87434600 -3.08239400

N -0.27424900 2.14810300 -0.28649500

C 2.96678900 -0.44999600 1.91677500

C 4.01672300 0.34492000 -0.23989100

C 5.18722600 -0.62406400 0.00450500

C 5.43458400 -0.90355700 1.47670600

C 4.15206700 -1.40997700 2.11364500

H 4.95857500 -1.58157000 -0.49984700

H 6.09461800 -0.22582200 -0.47852200

H 6.23836800 -1.64520800 1.59366400

H 5.78726000 0.00567000 1.98958900

H 3.89240300 -2.38322800 1.65675500

H 4.29404500 -1.59843500 3.19036700

N 2.79780700 -0.10145000 0.48330800

Cu 1.25749200 1.03951700 0.15120100

Li 1.91016800 -1.53576900 -0.49887700

C 2.63126700 -3.82043200 -2.01324800

C 2.03808900 -4.20198500 -0.80837400

C 0.79139800 -3.67960700 -0.44648000

C 0.08091100 -2.75544700 -1.24843200

C 0.71055700 -2.41603600 -2.46760100

C 1.95207500 -2.93406600 -2.85097800

H 3.60912800 -4.20897500 -2.29565600

H 2.55290700 -4.89800400 -0.14469700

H 0.36441200 -3.99494600 0.51025300

H 0.22398500 -1.70438700 -3.13997900

H 2.40109000 -2.62968800 -3.79747800

C 3.71581600 0.30890200 -1.73965400

H 3.40355400 -0.69507700 -2.07382800

H 2.92674100 1.02240800 -2.01709300

H 4.61243700 0.56678600 -2.32172500

C 4.46942400 1.78155600 0.07984300

H 4.92367800 1.88057800 1.07195500

H 5.21854600 2.12258500 -0.65121200

H 3.61574900 2.47406100 0.03760500

C 3.12322100 0.75499000 2.86145300

H 3.05707400 0.43170000 3.91176000

H 4.07831100 1.27817800 2.74576700

H 2.32234700 1.48801700 2.68418600

C 1.70458900 -1.18255700 2.36564500

H 0.82076300 -0.53235900 2.28985500

H 1.51354200 -2.08541100 1.76083800

H 1.78822200 -1.51166400 3.41177200

C 0.61235900 4.11569500 1.07083700

H 0.89160400 4.77499200 0.24270800

H 0.36681200 4.75905100 1.92983500

H 1.50258100 3.52534400 1.33609600

C -0.92692500 2.46731800 2.03757200

H -0.10805500 1.81432300 2.37954300

H -1.13564300 3.18812700 2.84096900

H -1.83866900 1.85491700 1.93020100

C 1.07223600 3.42623500 -2.05246300

H 1.19571500 3.47460100 -3.14541700

H 1.04795200 4.46075900 -1.69211800

H 1.97150300 2.95450700 -1.63284700

C -0.18977200 1.38872900 -2.59981700

H 0.72516700 0.79168100 -2.46175400

H -1.04836300 0.73015400 -2.40213300

H -0.23901300 1.68207700 -3.65891500

C -1.86948600 -1.24564100 2.42087000

H -1.10697800 -1.93483200 2.02417500

H -1.45944800 -0.22550800 2.38673800

H -2.02453600 -1.49670700 3.48060400

C -3.59813500 -2.83545100 1.74869400

H -3.54583900 -3.16771700 2.79716000

H -4.61917000 -3.02976300 1.40418800

H -2.92130700 -3.47231000 1.15817000

C -4.78933900 -1.96100400 -1.10216500

H -5.32648300 -2.51057500 -0.32231200

H -5.52207200 -1.72628500 -1.88971200

H -4.03701300 -2.63994600 -1.53083100

C -3.67088200 0.08307700 -1.84544200

H -3.20688500 1.04927400 -1.58897400

H -2.95065700 -0.50659300 -2.43563700

H -4.52796300 0.30936200 -2.49597500

Li -1.58027500 0.63950400 0.03421900

3-f)

Cartesian Coordinates

C -3.24932900 -1.10352700 1.69485400

C -4.11979300 -0.52174600 -0.59146200

C -5.07818900 0.49678000 0.04843400

C -5.46379100 0.13292200 1.47335700

C -4.21224000 -0.07575700 2.31158600

H -4.57681300 1.48258400 0.05969600

H -5.97543400 0.60773100 -0.58284700

H -6.08759400 0.92572800 1.91193400

H -6.08597700 -0.77665800 1.48098300

H -3.68136100 0.88971700 2.40224300

H -4.47632900 -0.38583000 3.33626600

N -2.94794900 -0.75228200 0.28572700

Cu -1.59426500 -1.90182700 -0.47220800

C -0.05330700 2.53843900 -1.78310900

C -0.33864700 3.19669500 0.62428800

C -1.55574700 4.05027000 0.22179100

C -1.48885900 4.56337000 -1.20573300

C -1.27438400 3.39793300 -2.15743100

H -2.46397000 3.42659300 0.32761900

H -1.66901200 4.88488100 0.93303000

H -2.41610300 5.09688700 -1.46143500

H -0.67995100 5.30434500 -1.30794600

H -2.17729400 2.76393400 -2.14683700

H -1.16009200 3.75594100 -3.19412000

N	-0.11769800	2.10695000	-0.36059600
C	4.04487500	0.01563900	-0.27056700
C	2.93566000	-0.61157700	1.89204600
C	3.54758600	0.62594800	2.56706800
C	4.83965100	1.08271600	1.89935900
C	4.65423200	1.25853500	0.39642400
H	2.80831200	1.44539100	2.51652900
H	3.72057800	0.42348800	3.63773100
H	5.17984900	2.02741600	2.34830500
H	5.64419800	0.35564500	2.09765600
H	3.97780500	2.11298500	0.21396000
H	5.61657100	1.50961100	-0.08122500
N	2.79701700	-0.39803300	0.42663300
Cu	1.34125200	0.88782800	0.07162700
Li	1.86671800	-1.79805900	-0.51029300
C	2.42870200	-4.03950400	-1.90496700
C	1.83571100	-4.38128400	-0.68781900
C	0.61366500	-3.80704700	-0.31792300
C	-0.07528500	-2.87425700	-1.12882100
C	0.55082100	-2.57883600	-2.36225000
C	1.77201500	-3.14144100	-2.74959100
H	3.38988200	-4.46531700	-2.19058400
H	2.33497100	-5.08270300	-0.01811000
H	0.19279600	-4.08451500	0.65273400
H	0.08161500	-1.86170400	-3.04117500
H	2.22187200	-2.86610500	-3.70448200
C	1.54018700	-0.84822100	2.46234600
H	0.86406900	-0.01061200	2.24111500
H	1.08828400	-1.76031400	2.03733200
H	1.57184800	-0.97322100	3.55464400
C	3.74179800	-1.86753600	2.28375400
H	3.54399000	-2.12308100	3.33515700
H	3.44245600	-2.73249400	1.67175700
H	4.82550200	-1.75188400	2.19022700
C	5.11444600	-1.09192400	-0.37630500
H	5.85610400	-0.82020600	-1.14187600
H	5.67164100	-1.26788700	0.54827100
H	4.66183300	-2.04882300	-0.68025900
C	3.68892300	0.35357100	-1.71734100
H	3.30016800	-0.53077500	-2.25255100
H	2.92912700	1.14254100	-1.77692000
H	4.57491500	0.70211500	-2.26718500
C	0.88034200	4.10344800	0.87065600
H	1.14803500	4.71964900	0.00657400
H	0.68885600	4.78781500	1.71149900
H	1.75975100	3.49286500	1.12635500
C	-0.67551700	2.56858000	1.97494400
H	0.15214500	1.95260800	2.35671300
H	-0.88154800	3.34587800	2.72466500
H	-1.58212700	1.94328100	1.92346400
C	1.21418900	3.32702100	-2.18710700
H	1.39852300	3.22600900	-3.26760900
H	1.13600000	4.40155500	-1.98354100
H	2.10249700	2.96181500	-1.65647600
C	-0.08341900	1.27844000	-2.65104600
H	0.83188900	0.67947700	-2.52810600
H	-0.93825300	0.63093700	-2.40463000
H	-0.16860500	1.54127300	-3.71593600
C	-1.94119300	-1.06033100	2.48149100
H	-1.25158200	-1.85214700	2.14812400
H	-1.42527400	-0.09558700	2.37236200
H	-2.12401000	-1.20914400	3.55597400
C	-3.80832000	-2.52465500	1.91132000
H	-3.77287200	-2.78755700	2.97990900
H	-4.84782800	-2.64542900	1.58960900
H	-3.20173200	-3.26045300	1.36154100

C	-4.89890600	-1.78916900	-0.99364700
H	-5.49475800	-2.21526900	-0.18017500
H	-5.59640600	-1.56402200	-1.81514400
H	-4.20443200	-2.56788900	-1.34254700
C	-3.61248500	0.08227900	-1.89818600
H	-3.06661900	1.02229400	-1.72190300
H	-2.94458100	-0.61367100	-2.43187700
H	-4.44733400	0.32530100	-2.57124200
Li	-1.51363400	0.67803200	0.00522200

3-g)

Cartesian Coordinates

C	-3.31550800	-0.98986500	1.66704700
C	-4.14497100	-0.66249000	-0.68693400
C	-5.09030900	0.44360900	-0.18815600
C	-5.49652800	0.26205800	1.26682800
C	-4.26228300	0.12318300	2.14581200
H	-4.57150600	1.41558800	-0.29230800
H	-5.98036400	0.49376500	-0.83712600
H	-6.10518200	1.11570300	1.59962700
H	-6.14150600	-0.62501900	1.37431900
H	-3.70708500	1.08076500	2.13374000
H	-4.55002900	-0.05754300	3.19485400
N	-2.99544800	-0.80467400	0.23403500
Cu	-1.58396000	-1.94507000	-0.41406400
C	-0.41177900	3.26906600	0.66057400
C	-0.27987000	2.54593700	-1.73632500
C	0.88739600	3.51954700	-1.97294600
C	0.86568000	4.70607900	-1.01673400
C	0.75129000	4.24893400	0.43204900
H	1.83490900	2.96860200	-1.83760800
H	0.87480000	3.87101800	-3.01895300
H	1.77517200	5.31049000	-1.14936300
H	0.02765400	5.37761600	-1.26504800
H	1.68765700	3.74137200	0.72753300
H	0.64357900	5.12098200	1.09913100
N	-0.35138400	2.13750400	-0.30469000
C	2.91169600	-0.48040700	1.89665500
C	3.95223800	0.16882300	-0.30542700
C	5.02591100	-0.91432300	-0.09179800
C	5.29417800	-1.21632600	1.37263600
C	3.99043800	-1.56365900	2.07189700
H	4.68450400	-1.84371100	-0.58650200
H	5.95419100	-0.61367500	-0.60489700
H	6.00925900	-2.04705600	1.46452300
H	5.77467100	-0.35298900	1.86022900
H	3.60483600	-2.51300600	1.65415500
H	4.15802900	-1.74242000	3.14686200
N	2.72167100	-0.15957100	0.45860900
Cu	1.20640000	1.00965000	0.10824300
Li	1.78545300	-1.62970200	-0.42014800
C	2.47694700	-4.04468500	-1.80686900
C	1.91499700	-4.34474700	-0.56474900
C	0.68845700	-3.78117300	-0.19639500
C	-0.03414000	-2.89623300	-1.03079200
C	0.56344400	-2.63982800	-2.28667700

C	1.78663900	-3.19715000	-2.67475600
H	3.44007500	-4.46513100	-2.09416000
H	2.43972500	-5.00878900	0.12351100
H	0.28812000	-4.03044300	0.79048300
H	0.06711500	-1.96146200	-2.98640500
H	2.21123300	-2.95575000	-3.65024400
C	3.61227100	0.16905800	-1.79691900
H	3.12930200	-0.76931800	-2.11948300
H	2.94832900	1.00336500	-2.06276900
H	4.52712200	0.27372500	-2.39821600
C	4.54754500	1.55652600	-0.00931000
H	5.32020200	1.80975200	-0.75157000
H	3.76257700	2.32549100	-0.06261400
H	5.01482100	1.62882400	0.97821200
C	3.25694100	0.72018000	2.79925500
H	3.07806700	0.46859000	3.85574300
H	4.30374000	1.03425900	2.72214800
H	2.63459500	1.59050200	2.54840000
C	1.59632700	-1.05322200	2.42318400
H	0.79334400	-0.30197400	2.40223100
H	1.25633700	-1.92311400	1.83597700
H	1.70652200	-1.39476900	3.46281100
C	-1.57444400	3.18227700	-2.29175300
H	-1.65658400	4.25590700	-2.09241500
H	-1.61182600	3.06513800	-3.38476600
H	-2.47234100	2.70331100	-1.87662600
C	-0.04941500	1.28804900	-2.57733000
H	-0.90310200	0.59447800	-2.51182900
H	0.08062200	1.54827100	-3.63804600
H	0.84668900	0.73900100	-2.25545600
C	-1.74924600	4.03610500	0.65370000
H	-1.82011000	4.66898000	1.55080000
H	-1.88495700	4.69485500	-0.20875900
H	-2.59929100	3.33604600	0.67643000
C	-0.29386100	2.70638400	2.07579100
H	-1.17547900	2.10037300	2.33952300
H	0.60036800	2.07760100	2.19531600
H	-0.22966400	3.52033800	2.81239100
C	-2.01069700	-0.87449000	2.45350800
H	-1.33148800	-1.71198700	2.22721200
H	-1.47400400	0.06129200	2.23038700
H	-2.20126600	-0.88028400	3.53686800
C	-3.89872000	-2.36641900	2.04056500
H	-3.30544800	-3.16802000	1.57609500
H	-3.86800300	-2.50911800	3.13186700
H	-4.94050300	-2.50181000	1.73241200
C	-4.94150300	-1.95594800	-0.94331700
H	-5.56116000	-2.26504000	-0.09521000
H	-5.61815200	-1.82397500	-1.80157600
H	-4.25539000	-2.78371000	-1.17566600
C	-3.60044100	-0.23376300	-2.04843200
H	-3.02428300	0.70158900	-1.97946400
H	-2.94575700	-1.00806800	-2.48144100
H	-4.41874300	-0.05040300	-2.75983200
Li	-1.65600900	0.67598000	0.00161800

3-h)

Cartesian Coordinates

C -3.37596800 -0.49732000 1.76266100

C -4.20201800 -0.60329700 -0.61656500

C -5.24479600 0.47587900 -0.28552000

C -5.67313300 0.44702300 1.17274900

C -4.44854300 0.56348800 2.06483200

H -4.80326000 1.46519300 -0.50629200

H -6.11595400 0.36287000 -0.95170200

H -6.37315900 1.26935900 1.38176000

H -6.22482200 -0.48092400 1.39415300

H -4.00037600 1.56243900 1.90982200

H -4.73006500 0.50866700 3.12945200

N -3.05078100 -0.52334100 0.31676100

Cu -1.68483300 -1.78864500 -0.21582100

C 0.07118600 3.31284100 0.54512800

C -0.29581400 2.39538400 -1.76200900

C 0.74198200 3.38152900 -2.32277200

C 0.85249200 4.64960700 -1.48983200

C 1.10563400 4.29443200 -0.03356200

H 1.72574400 2.88235400 -2.34182400

H 0.49154500 3.62290900 -3.36989700

H 1.66698700 5.28231000 -1.87220900

H -0.06267600 5.25683200 -1.58293100

H 2.10076800 3.82007400 0.04439800

H 1.14015100 5.20488400 0.58796400

N -0.08141000 2.10849000 -0.31532500

C 4.04156200 -0.12619800 -0.32623000

C 2.89552800 -0.85059400 1.79677200

C 3.69755000 0.20220900 2.57818400

C 5.03139600 0.53387800 1.92485200

C 4.82005200 0.93138800 0.47151500

H 3.09455800 1.12403700 2.63945600

H 3.84791300 -0.14288000 3.61496900

H 5.52605000 1.34985000 2.47184100

H 5.71732700 -0.32659700 1.99067600

H 4.24300300 1.87328200 0.44124100

H 5.78607300 1.13415500 -0.02076700

N 2.77188700 -0.49292300 0.35550800

Cu 1.36112600 0.83096300 0.01875300

Li 1.78207000 -1.88223600 -0.54652800

C 2.07604100 -4.22945900 -1.88436600

C 1.57751400 -4.50738300 -0.61041000

C 0.43884500 -3.84278500 -0.14093300

C -0.25947000 -2.87968900 -0.90464200

C 0.26214600 -2.65687000 -2.20164700

C 1.40315100 -3.30522800 -2.68712100

H 2.97573800 -4.72550400 -2.24658400

H 2.08944800 -5.22941200 0.02715200

H 0.09846900 -4.06959200 0.87288500

H -0.22230600 -1.92653200 -2.85572500

H 1.77566300 -3.08070800 -3.68741600

C 1.48934500 -0.92136500 2.39163500

H 0.96377100 0.03967600 2.29962100

H 0.87546400 -1.68902700 1.88896600

H 1.52866600 -1.18024600 3.46002900

C 3.51654100 -2.24163100 2.04750700

H 3.33702200 -2.55178900 3.08753700

H 3.06235200 -3.00628900 1.39707800

H 4.59903400 -2.27794400 1.89144100

C 4.95830400 -1.33043200 -0.62572200

H 5.75869300 -1.03440100 -1.32003000

H 5.44758600 -1.75033400 0.25815400

H 4.39195500 -2.14417200 -1.10813600

C 3.70143200 0.46863600 -1.69172100

H 3.14494300 -0.25003100 -2.31707600

H 3.09015000 1.37794100 -1.59210200

H 4.61667800 0.73955200 -2.23794200

C	-1.70581900	2.94680800	-2.08802100
H	-1.78461900	4.03223600	-1.96253400
H	-1.97030500	2.73312900	-3.13414100
H	-2.48587700	2.49799600	-1.45401800
C	-0.15747300	1.07848000	-2.52956000
H	-0.87469800	0.31613100	-2.18016300
H	-0.34264700	1.22979500	-3.60333900
H	0.85119900	0.65553800	-2.41724500
C	-1.25824000	4.04543500	0.81118600
H	-1.12037700	4.82089400	1.57937400
H	-1.67720800	4.54023100	-0.06989400
H	-2.01842500	3.34244800	1.18931700
C	0.58254900	2.88461200	1.91950300
H	-0.09685600	2.16583100	2.40127600
H	1.57759000	2.41977200	1.85050100
H	0.67103300	3.75522500	2.58583500
C	-2.10988800	-0.08807000	2.51494800
H	-1.29118500	-0.80387800	2.34188400
H	-1.76454800	0.91899000	2.22267100
H	-2.28945600	-0.04149100	3.59848200
C	-3.79769200	-1.85319300	2.35844500
H	-3.07232200	-2.63216800	2.07784400
H	-3.83011000	-1.79899400	3.45769900
H	-4.78589000	-2.18630500	2.02531300
C	-4.89387200	-1.98034700	-0.67707000
H	-5.48135300	-2.21746300	0.21595100
H	-5.58438800	-2.02236900	-1.53352400
H	-4.14766600	-2.77885500	-0.80601000
C	-3.68783700	-0.33723100	-2.03131700
H	-3.17917100	0.63384400	-2.10084100
H	-2.98237700	-1.11940500	-2.35752200
H	-4.52001300	-0.32357500	-2.75031200
Li	-1.57204500	0.82104700	0.06533400

Structure optimization for 4

4-a)

Cartesian Coordinates

Cu -0.74109500 1.89403000 0.00005900

Li -1.65665800 -0.73887500 -0.00015000

N -2.45387800 1.01366100 -0.00048600

C -3.19354300 1.19226800 -1.27051400

C -3.47997300 2.65677100 -1.64460900

H -3.86341200 2.72614900 -2.67430000

H -2.55719200 3.25384400 -1.58503400

H -4.22193900 3.13020800 -0.99207800

C -2.31870200 0.62269300 -2.39140400

H -2.83441700 0.68226300 -3.36075900

H -2.07784000 -0.44301500 -2.23017200

H -1.36971700 1.17685600 -2.48236800

C -4.50743600 0.39372200 -1.24552300

H -5.08626300 0.60717000 -2.15905900

H -4.26417900 -0.68552800 -1.27436700

C -5.33782400 0.66818700 -0.00129100

H -6.23953100 0.03862500 -0.00157900

H -5.69743100 1.70994600 -0.00131200

C -4.50810500 0.39354800 1.24334600

H -4.26481700 -0.68571800 1.27213200

H -5.08742600 0.60680500 2.15661100

C -3.19426000 1.19213300 1.26915000

C -3.48094700 2.65659100 1.64321600

H -3.86497900 2.72586100 2.67269300

H -4.22255300 3.13006400 0.99030700

H -2.55815100 3.25369800 1.58423000

C -2.31999700 0.62249000 2.39047500

H -2.83621300 0.68203300 3.35956800

H -1.37103600 1.17661700 2.48194600

H -2.07908600 -0.44320500 2.22934800

C 0.97472600 2.77928200 0.00081000

C 1.63256100 3.15039600 -1.19264600

H 1.17779500 2.89892200 -2.15594700

C 2.84681000 3.84039000 -1.20082500

H 3.31587800 4.10835500 -2.14819300

C 3.46111100 4.18267800 0.00215800

H 4.41237300 4.71395300 0.00267000

C 2.84589700 3.83969500 1.20447600

H 3.31425600 4.10710300 2.15235400

C 1.63165300 3.14972300 1.19497900

H 1.17615500 2.89769500 2.15780800

Cu 0.74106600 -1.89393100 -0.00012300

Li 1.65672200 0.73896500 0.00007200

N 2.45388500 -1.01360000 -0.00051100

C 3.19418200 -1.19230400 1.26913900

C 3.48069200 -2.65683600 1.64304600

H 3.86482200 -2.72625600 2.67247700

H 2.55779600 -3.25380100 1.58410900

H 4.22215000 -3.13036800 0.99001300

C 2.31993500 -0.62270900 2.39050100

H 2.83607500 -0.68249000 3.35961600

H 2.07919100 0.44306900 2.22954700

H 1.37088700 -1.17670700 2.48182400

C 4.50809900 -0.39383000 1.24349500

H 5.08735200 -0.60726000 2.15676500

H 4.26490600 0.68543500 1.27240000

C 5.33786800 -0.66838800 -0.00113000

H 6.23961900 -0.03888800 -0.00129200

H 5.69740600 -1.71017100 -0.00126000

C 4.50757100 -0.39370200 -1.24537600

H 4.26442800 0.68558900 -1.27410300

H 5.08642100 -0.60709600 -2.15890700

C 3.19359900 -1.19210900 -1.27052600

C 3.47992700 -2.65658700 -1.64479700

H 3.86339000 -2.72586300 -2.67448600

H 4.22184300 -3.13015500 -0.99231000

H 2.55710300 -3.25360500 -1.58532400

C 2.31884600 -0.62231400 -2.39137100

H 2.83458900 -0.68179300 -3.36072000

H 1.36982100 -1.17639900 -2.48244000

H 2.07806900 0.44337200 -2.22998200

C -0.97473800 -2.77922100 0.00065200

C -1.63157400 -3.14966400 1.19487200

H -1.17604600 -2.89757900 2.15765200

C -2.84576300 -3.83972900 1.20445900

H -3.31404100 -4.10716000 2.15236900

C -3.46103800 -4.18276200 0.00218800

H -4.41227000 -4.71409100 0.00277000

C -2.84684200 -3.84044800 -1.20084200

H -3.31596700 -4.10844000 -2.14817700

C -1.63261900 -3.15040700 -1.19275300

H -1.17791600 -2.89893300 -2.15610500

4-b)

Cartesian Coordinates

Cu -0.91773500 -1.75379900 0.02098700

Li -1.62515800 0.96969900 -0.01628900

N -2.55155500 -0.71021000 -0.04837000

C -3.27211700 -0.91447300 -1.32937200

C -4.18574700 0.26377200 -1.72052600

H -4.51265500 0.16221800 -2.76607700

H -3.65232000 1.22552300 -1.63630800

H -5.09197000 0.33864100 -1.11121900

C -2.22704500 -1.01540100 -2.44315700

H -2.70972800 -1.14259300 -3.42308300

H -1.55082600 -1.87190300 -2.29071900

H -1.60773000 -0.10316700 -2.49621400

C -4.08574300 -2.21735600 -1.30561600

H -4.68280300 -2.30256800 -2.22902900

H -3.37650900 -3.06397800 -1.30447100

C -4.97876000 -2.32729600 -0.07712700

H -5.48663300 -3.30267300 -0.06758400

H -5.78230000 -1.57360600 -0.11854700

C -4.15711700 -2.15633700 1.19340200

H -3.45113900 -3.00176600 1.27438400

H -4.80594200 -2.19591400 2.08443100

C -3.34221300 -0.85408300 1.19962600

C -4.27205900 0.34290500 1.48240800

H -4.65821800 0.29168200 2.51128000

H -5.14150300 0.38971200 0.81920000

H -3.73293000 1.30091500 1.38508700

C -2.36039900 -0.90412300 2.37345500

H -2.89746400 -0.98309700 3.32988000

H	-1.73894700	0.00760400	2.42098600
H	-1.68209900	-1.76902300	2.29905900
C	0.71402600	-2.78828900	0.09550600
C	1.30283800	-3.17501600	1.31975000
H	0.85457800	-2.83955400	2.26014000
C	2.43940000	-3.98343600	1.38885300
H	2.85486700	-4.25908600	2.35878900
C	3.04468400	-4.43507700	0.21780700
H	3.93551500	-5.06074400	0.26440900
C	2.49859500	-4.07821800	-1.01336300
H	2.96050300	-4.42870000	-1.93703400
C	1.36103400	-3.26942000	-1.06417700
H	0.95895000	-3.01203600	-2.04905200
Cu	0.89768900	1.86235700	-0.00803500
Li	1.57367800	-0.82328800	0.02007700
N	2.53225000	0.84481600	-0.04746300
C	3.24371000	0.94345100	-1.34198800
C	3.62552800	2.37556100	-1.75514400
H	3.97947800	2.39715900	-2.79744200
H	2.75177000	3.04072200	-1.67936800
H	4.42106000	2.80450600	-1.13584500
C	2.29470000	0.42070700	-2.42443200
H	2.78581300	0.41557800	-3.40829600
H	1.97186000	-0.61747300	-2.22960000
H	1.39228700	1.04908200	-2.50431800
C	4.49588100	0.05082500	-1.33989400
H	5.05989800	0.20540500	-2.27433200
H	4.17380100	-1.00754300	-1.33970100
C	5.38222100	0.28492900	-0.12657000
H	6.23514400	-0.40916600	-0.14130500
H	5.81650900	1.29737200	-0.15677900
C	4.57256100	0.09245900	1.14613200
H	4.24981100	-0.96464200	1.20040100
H	5.19333600	0.27548900	2.03850400
C	3.32344100	0.98780600	1.19616800
C	3.72874600	2.43295300	1.53424100
H	4.15423000	2.48946600	2.54808900
H	4.47745800	2.84231100	0.84729000
H	2.84953900	3.09437600	1.49797000
C	2.44514700	0.50463200	2.35442700
H	2.99436300	0.54396900	3.30624700
H	1.54322300	1.12959600	2.46291100
H	2.12174600	-0.54310100	2.22350900
C	-0.72358200	2.90735100	0.05569500
C	-1.38635500	3.36201400	-1.10577500
H	-1.00136500	3.07649100	-2.09005500
C	-2.51864900	4.17866500	-1.05526100
H	-2.99577500	4.50780800	-1.97905000
C	-3.04087500	4.56954600	0.17588000
H	-3.92725000	5.20142600	0.22186100
C	-2.41946500	4.14243200	1.34787200
H	-2.81894300	4.44300200	2.31705500
C	-1.29064400	3.32331600	1.28068700
H	-0.83025700	3.00382300	2.22076200

4-c)

Cartesian Coordinates

Cu	-1.09032300	1.69440800	-0.02934000
Li	-1.52351100	-1.06675100	-0.03203900
N	-2.61315700	0.50283300	0.13171900
C	-3.25416200	0.59888800	1.46620300
C	-4.02471200	-0.67147200	1.87745300
H	-4.28233800	-0.63167600	2.94629900
H	-3.41208000	-1.57495500	1.72120300
H	-4.96226000	-0.81409700	1.33091700
C	-2.14496400	0.76314500	2.50740400
H	-2.56524700	0.82154300	3.52198400
H	-1.55856100	1.68015200	2.33552300
H	-1.44773100	-0.09263400	2.48811000
C	-4.18437900	1.81847800	1.54722300
H	-4.71505500	1.82092900	2.51406000
H	-3.55898600	2.72854100	1.52341800
C	-5.17421100	1.87508700	0.39151500
H	-5.77187800	2.79622800	0.45254100
H	-5.89691700	1.04578600	0.46482800
C	-4.43807700	1.82055500	-0.94016500
H	-3.82550900	2.73375300	-1.04233800
H	-5.15290600	1.82064900	-1.78009500
C	-3.50351100	0.60616700	-1.05202600
C	-4.32931900	-0.66887300	-1.31423500
H	-4.79855600	-0.62234000	-2.30820700
H	-5.13464900	-0.82672000	-0.58996200
H	-3.69130500	-1.56927400	-1.29817400
C	-2.62035500	0.79217700	-2.28913100
H	-3.23129000	0.83796500	-3.20243900
H	-1.91071600	-0.04537100	-2.41177500
H	-2.03458600	1.72338800	-2.23318400
C	0.41927800	2.88230000	-0.24024200
C	0.90915500	3.23460200	-1.51778000
H	0.45850800	2.78465400	-2.40785900

C	1.94878800	4.14858900	-1.70134800
H	2.28874400	4.39354100	-2.70818700
C	2.55717000	4.74243300	-0.59714400
H	3.37431300	5.45014200	-0.73307400
C	2.11245900	4.41817000	0.68271400
H	2.58111800	4.87545200	1.55490900
C	1.06854500	3.50457800	0.84892000
H	0.74554700	3.27550800	1.86951100
Cu	1.09033700	-1.69444800	-0.02933800
Li	1.52350300	1.06667800	-0.03254500
N	2.61316000	-0.50285800	0.13167500
C	3.50356900	-0.60627600	-1.05202200
C	4.32934300	0.66876800	-1.31431900
H	4.79862200	0.62216100	-2.30826800
H	3.69130400	1.56915600	-1.29836700
H	5.13463600	0.82671200	-0.59002600
C	2.62047100	-0.79244000	-2.28914600
H	3.23144600	-0.83829800	-3.20242500
H	2.03472700	-1.72366200	-2.23313100
H	1.91080600	0.04507000	-2.41191700
C	4.43817700	-1.82061700	-0.94001000
H	5.15303900	-1.82076400	-1.77991100
H	3.82564700	-2.73384800	-1.04211800
C	5.17425700	-1.87498900	0.39170600
H	5.77196200	-2.79609800	0.45284600
H	5.89692300	-1.04564900	0.46497200
C	4.18437100	-1.81831300	1.54736400
H	3.55901700	-2.72840400	1.52361400
H	4.71500400	-1.82065300	2.51422500
C	3.25410600	-0.59876700	1.46619500
C	4.02458200	0.67166300	1.87736500
H	4.28217600	0.63196800	2.94622200
H	4.96214300	0.81428400	1.33084700

H	3.41190800	1.57509700	1.72101400	C	-2.11242200	-4.41823100	0.68275000
C	2.14487300	-0.76298400	2.50736500	H	-2.58103200	-4.87556200	1.55494500
H	2.56512100	-0.82128000	3.52196500	C	-2.55720900	-4.74241400	-0.59710100
H	1.44760700	0.09276100	2.48796400	H	-3.37436700	-5.45010700	-0.73302700
H	1.55852300	-1.68003600	2.33553900	C	-1.94888500	-4.14851000	-1.70130600
C	-0.41927700	-2.88232300	-0.24021700	H	-2.28890500	-4.39339600	-2.70813800
C	-1.06848400	-3.50466200	0.84894800	C	-0.90923500	-3.23454100	-1.51774600
H	-0.74541500	-3.27566400	1.86953300	H	-0.45864700	-2.78453200	-2.40782300

Structure optimization for 5

5-a)

Cartesian Coordinates

C	3.92505600	-0.78826000	0.63215600
C	3.06234300	-1.10258500	-1.72692000
C	3.86449600	-2.40961700	-1.82770000
C	5.10218000	-2.41704900	-0.94618900
C	4.71938400	-2.09714400	0.48895700
H	3.20505700	-3.24184400	-1.52384100
H	4.13611700	-2.58648400	-2.88166600
H	5.59588300	-3.39858700	-0.99564100
H	5.84396400	-1.69099700	-1.31674100
H	4.09558500	-2.91865600	0.88357400
H	5.61711000	-2.04533600	1.12682700
N	2.75756400	-0.78434400	-0.29979900
Cu	1.28636300	-1.88874900	0.38522900
Cu	1.46920600	0.66639000	-0.03531000
C	0.24600900	2.56894000	1.70657500
C	0.43720600	3.22742400	-0.73256700
C	1.75251600	3.95684400	-0.41596400
C	1.83890400	4.43857800	1.02492800
C	1.56883600	3.29642200	1.99445900
H	2.58771600	3.26054000	-0.61142400
H	1.87841500	4.80180100	-1.11286900
H	2.83289000	4.86781300	1.21818200
H	1.12339500	5.25941800	1.19428200
H	2.39329500	2.56493400	1.92678300
H	1.56161000	3.66704900	3.03295200
N	0.19594400	2.14533500	0.27127900
C	-3.13859700	-0.39763800	-1.85703900
C	-4.08626800	0.32021400	0.37177100
C	-5.23752100	-0.68214600	0.17754400
C	-5.56185400	-0.94030300	-1.28404300
C	-4.30512900	-1.38833300	-2.01088400
H	-4.94799700	-1.64021100	0.64894300
H	-6.12804400	-0.32389300	0.71973800
H	-6.34612800	-1.70664700	-1.37047300
H	-5.97546800	-0.03288900	-1.75276700
H	-3.98953600	-2.36519500	-1.59900200
H	-4.50715700	-1.55187000	-3.08217400
N	-2.90114900	-0.08178400	-0.42645200
Cu	-1.34807600	1.04016800	-0.12856700
Li	-1.94349900	-1.54080400	0.43471500
C	-2.70382100	-4.01438500	1.92911500
C	-2.17863500	-4.33181200	0.67564200
C	-0.96654000	-3.76988800	0.26152300
C	-0.22549600	-2.86540200	1.05728000
C	-0.78472600	-2.59314000	2.32744100
C	-1.99144300	-3.15117200	2.76228400
H	-3.65550100	-4.43474800	2.25236900
H	-2.71995200	-5.01041900	0.01498800
H	-0.59390100	-4.03657500	-0.73154300
H	-0.27038500	-1.89977500	2.99958000
H	-2.38634100	-2.89746000	3.74711800
C	-3.69698200	0.27878100	1.85047300
H	-3.29734100	-0.70592700	2.14794800
H	-2.94343800	1.04238700	2.09260000
H	-4.57342700	0.46772800	2.48737200
C	-4.59657300	1.74744200	0.10255200

H	-5.31201100	2.05421800	0.88102400
H	-3.75921100	2.46077900	0.11177600
H	-5.10811600	1.85162800	-0.86051800
C	-3.38467100	0.82637100	-2.75756400
H	-3.33784400	0.53913900	-3.81922200
H	-4.36084100	1.29598500	-2.59540800
H	-2.61674100	1.59410100	-2.58106900
C	-1.88164500	-1.08093200	-2.39279900
H	-1.00993800	-0.41285300	-2.33338700
H	-1.63940900	-2.00015200	-1.83264700
H	-2.00973200	-1.37504600	-3.44464100
C	-0.71193000	4.24210700	-0.86047400
H	-0.79488300	4.93027700	-0.01430900
H	-0.56366800	4.85905000	-1.75894000
H	-1.67558100	3.72212100	-0.96682600
C	0.57320700	2.58522400	-2.11118800
H	-0.35776800	2.08002200	-2.41217400
H	0.80041800	3.34832300	-2.86941700
H	1.38439800	1.84316000	-2.13329800
C	-0.92864400	3.45703100	2.15989200
H	-0.99781800	3.44359500	3.25774100
H	-0.83151200	4.50698900	1.86613400
H	-1.88188200	3.08474000	1.75929300
C	0.17424500	1.31857800	2.58440500
H	-0.82059600	0.85102900	2.53714200
H	0.90824600	0.55946400	2.28150700
H	0.36970700	1.57973700	3.63494400
C	3.42073900	-0.72334200	2.07366700
H	2.97274300	0.25381900	2.30216800
H	2.67239200	-1.50560800	2.28209700
H	4.25846500	-0.86998900	2.77081300
C	4.86293300	0.41931200	0.46426700
H	4.28124200	1.35215400	0.42383900
H	5.54702000	0.48491300	1.32348100
H	5.48574200	0.37483300	-0.43476000
C	3.80689400	0.01586700	-2.47995800
H	4.87780400	0.05762800	-2.25613100
H	3.71759500	-0.13832700	-3.56544000
H	3.38173500	1.00123400	-2.24310000
C	1.74463400	-1.31419900	-2.46924100
H	1.13511700	-2.10126300	-1.99666400
H	1.14510300	-0.39261300	-2.49282200
H	1.93544000	-1.61645200	-3.50923300

5-b)

Cartesian Coordinates

C	-3.92369522	-0.71945876	-0.66238945
C	-3.05799044	-1.21220073	1.66696386
C	-3.92300174	-2.48221937	1.69763909
C	-5.16630813	-2.37890025	0.83040423
C	-4.77664489	-1.99658235	-0.58743688
H	-3.30719753	-3.32510369	1.33703814
H	-4.19379785	-2.70952959	2.74202181
H	-5.70665041	-3.33686611	0.82838778
H	-5.86937240	-1.64073954	1.24937252
H	-4.19312455	-2.82235961	-1.03155652

H -5.67375072 -1.86757692 -1.21516457
N -2.75189933 -0.81990921 0.25901839
Cu -1.32013782 -1.92228522 -0.50183873
Cu -1.41535697 0.60133533 0.06372792
C -0.19001829 2.56137781 -1.60058265
C -0.33200149 3.10678498 0.86188053
C -1.66739593 3.82737301 0.61196549
C -1.81195092 4.35954054 -0.80652038
C -1.53486667 3.26906276 -1.83248011
H -2.48489191 3.11212900 0.81272366
H -1.78182490 4.64641983 1.34115361
H -2.82411571 4.76426300 -0.95346116
H -1.12822857 5.20974720 -0.96140655
H -2.34112410 2.51640900 -1.78751431
H -1.55370462 3.68890449 -2.85216400
N -0.10947506 2.06627183 -0.18939179
C 4.15189915 0.21084280 -0.33706620
C 3.13822124 -0.62420741 1.80623628
C 3.73659241 0.57830623 2.55363150
C 4.99477237 1.11942346 1.88639484
C 4.74554067 1.41359574 0.41249958
H 2.97642457 1.37955272 2.58223381
H 3.94436733 0.30263151 3.60141970
H 5.33003080 2.03207813 2.40058984
H 5.82173242 0.39855486 1.99428565
H 4.03275560 2.25409593 0.32942567
H 5.67832377 1.73960472 -0.07814069
N 2.94871160 -0.31279398 0.36367741
Cu 1.41030212 0.88744187 0.12483358
Li 2.06836012 -1.71307757 -0.62123509
C 2.70650824 -3.87528567 -2.15949068
C 2.16944570 -4.30812941 -0.94561984
C 0.94037892 -3.80516089 -0.50297018
C 0.18972755 -2.85462179 -1.23360230
C 0.75797893 -2.47102727 -2.47150915
C 1.98569016 -2.96042233 -2.93051156
H 3.67333818 -4.24457431 -2.49954798
H 2.71833226 -5.02532966 -0.33388639
H 0.56677706 -4.15260446 0.46411538
H 0.23750929 -1.73643024 -3.09275196
H 2.39003880 -2.61557049 -3.88322402
C 1.76807830 -0.94122931 2.40053581
H 1.06462019 -0.10783266 2.26492848
H 1.32214024 -1.83084320 1.92483982
H 1.84261030 -1.14539085 3.47875499
C 3.99297389 -1.87917989 2.07955545
H 3.84948503 -2.20903906 3.11907596
H 3.68941966 -2.71133586 1.42513285
H 5.06804493 -1.72663418 1.94589531
C 5.25178626 -0.84405229 -0.57899118
H 5.97019514 -0.47141567 -1.32404292
H 5.83094121 -1.09967246 0.31297906
H 4.82129783 -1.77774137 -0.97463583
C 3.72906461 0.66749072 -1.73239227
H 3.32320419 -0.17107422 -2.32506262
H 2.96106476 1.45103420 -1.68700753
H 4.58693216 1.07349755 -2.28750675
C 0.80731971 4.13190029 0.99296058
H 0.68142403 4.70644999 1.92253402
H 1.77937018 3.61928383 1.04520652
H 0.85346335 4.85705913 0.17548781
C -0.41776080 2.42170544 2.22366519
H 0.54952239 1.98703969 2.51636491
H -0.70220305 3.14896985 2.99779433
H -1.16970999 1.61878929 2.22908181
C 0.94794688 3.51293755 -2.02567265

H	1.06849769	3.47918446	-3.11850059
H	0.76323819	4.56095109	-1.76678484
H	1.90547590	3.22379366	-1.57334517
C	-0.09923955	1.35711943	-2.53974706
H	0.91540088	0.93209313	-2.55021644
H	-0.79040399	0.55383472	-2.24769170
H	-0.34389997	1.65930816	-3.56900434
C	-3.42170490	-0.59730411	-2.10123368
H	-2.91766284	0.36387463	-2.27513311
H	-2.71960960	-1.40727441	-2.35899042
H	-4.26836201	-0.65485961	-2.80068237
C	-4.80713261	0.51624156	-0.41960451
H	-4.18642223	1.41873660	-0.32022208
H	-5.48611655	0.66561646	-1.27234561
H	-5.43339751	0.44293220	0.47510981
C	-3.73614015	-0.10680583	2.49621770
H	-4.79714246	0.02828456	2.26281689
H	-3.67592888	-0.35004702	3.56734756
H	-3.23827231	0.86079242	2.34191408
C	-1.74593072	-1.53756444	2.37781508
H	-1.19110692	-2.33209105	1.85293154
H	-1.09079330	-0.65690186	2.44313364
H	-1.94221144	-1.88645870	3.40212361

5-c)

Cartesian Coordinates

C	-4.00196000	0.51299500	0.68618400
C	-3.21919600	0.87771500	-1.69801000
C	-4.14794000	2.09789400	-1.79295800
C	-5.36217600	1.99477300	-0.88478000
C	-4.91813300	1.73903700	0.54604800
H	-3.56689100	2.99310800	-1.50900000
H	-4.45633600	2.23773700	-2.84218300
H	-5.95294400	2.92097900	-0.93690000
H	-6.03404600	1.19199500	-1.22965600
H	-4.36515300	2.62264000	0.91060600
H	-5.79113900	1.61447000	1.20775400
N	-2.86306100	0.60114900	-0.27589600
Cu	-1.45725400	1.80377100	0.37862600
Cu	-1.49979300	-0.76964500	-0.02968700
C	-0.21576100	-3.25511000	-0.72839700
C	-0.09879400	-2.58028200	1.71001700
C	1.07345000	-3.55375800	1.91047600
C	1.05028400	-4.72082900	0.93308900
C	0.95536700	-4.22390700	-0.50242600
H	2.01783700	-2.99673000	1.77904200
H	1.06771400	-3.92224500	2.94978600
H	1.95469300	-5.33375000	1.05949000
H	0.20456500	-5.39024300	1.15908000
H	1.89173500	-3.69903800	-0.76514900
H	0.86595200	-5.07421200	-1.19851800
N	-0.16785600	-2.14962000	0.27801100
C	3.00831800	0.63590800	-1.87823500
C	4.05914100	-0.01697500	0.31664000
C	5.08235800	1.12033700	0.13910400
C	5.34756100	1.47210000	-1.31486900

C 4.03434200 1.77390900 -2.01747500

H 4.69342500 2.01948100 0.65403300

H 6.01999600 0.85062700 0.65245500

H 6.02236000 2.33839200 -1.37891200

H 5.87316100 0.64564100 -1.81960500

H 3.59973100 2.69258100 -1.57966500

H 4.20153400 1.98747600 -3.08615000

N 2.82180900 0.27375400 -0.44988900

Cu 1.33715200 -0.93901100 -0.12774600

Li 1.80136300 1.67508000 0.43863000

C 2.39835600 4.15729100 1.93530100

C 1.85911000 4.45340300 0.68259500

C 0.68253500 3.82565700 0.25987200

C -0.00881100 2.87538800 1.04694600

C 0.56029000 2.62894600 2.31832200

C 1.73305100 3.24995000 2.76067700

H 3.32424200 4.62801400 2.26401200

H 2.36310700 5.16598200 0.02816900

H 0.30017400 4.07543700 -0.73390000

H 0.08360000 1.90485800 2.98569200

H 2.13862200 3.01206900 3.74511700

C 3.70910700 -0.07145400 1.80453800

H 3.16515100 0.82710400 2.14351200

H 3.09802000 -0.95234400 2.04562000

H 4.62456300 -0.13220500 2.41098200

C 4.72009000 -1.36802900 -0.00899500

H 5.48761800 -1.61141100 0.74183600

H 3.96723100 -2.17029400 0.00338700

H 5.21075400 -1.38907200 -0.98768100

C 3.41875800 -0.52545400 -2.80438600

H 3.24172800 -0.25597600 -3.85672500

H 4.47734600 -0.79468700 -2.72062200

H 2.83288400 -1.42857800 -2.58336400

C 1.67057900 1.15465100 -2.40297100

H 0.90581900 0.36470000 -2.39475300

H 1.28543400 1.99757500 -1.80469200

H 1.76823000 1.51654200 -3.43688400

C -1.39030000 -3.21955100 2.25400600

H -1.53289200 -4.26088900 1.94923600

H -1.36968300 -3.21402900 3.35378700

H -2.27520700 -2.65263000 1.93265400

C 0.14089400 -1.34349700 2.57641200

H -0.72325400 -0.66321000 2.55257900

H 0.31038000 -1.63773100 3.62258000

H 1.01838100 -0.77340300 2.24169400

C -1.54385100 -4.03155700 -0.75750800

H -1.59781600 -4.63888000 -1.67300100

H -1.67797800 -4.71720700 0.08424200

H -2.39673700 -3.33682900 -0.76479800

C -0.06781400 -2.64651900 -2.12105700

H -0.92310600 -1.99890700 -2.36858700

H 0.84920400 -2.04549500 -2.20518800

H -0.01764000 -3.43870200 -2.88190000

C -3.44644300 0.51725200 2.11081300

H -2.86877400 -0.39500000 2.32047500

H -2.79457200 1.38742900 2.29230200

H -4.27040900 0.55982500 2.83790300

C -4.83036500 -0.77764900 0.56052100

H -5.46430300 -0.90598000 1.45046000

H -5.49762600 -0.79120900 -0.30728300

H -4.16907700 -1.65348000 0.48689000

C -3.85148300 -0.31936100 -2.42894900

H -4.88390400 -0.52383600 -2.12852000

H -3.86601500 -0.13349700 -3.51319500

H -3.26620700 -1.23340600 -2.25086400

C -1.93987300 1.21027500 -2.46236600

H	-1.40852700	2.06429800	-2.01190400
H	-1.25015200	0.35345700	-2.47570700
H	-2.17234900	1.47085500	-3.50519700

5-d)

Cartesian Coordinates

C	4.02606100	-0.26050400	0.61993400
C	3.24624300	-0.70206100	-1.75283300
C	4.29017500	-1.82456600	-1.86002600
C	5.50361600	-1.59731800	-0.97508500
C	5.05661100	-1.38994500	0.46192100
H	3.80615900	-2.77134000	-1.56171700
H	4.59078700	-1.93879400	-2.91452300
H	6.18568600	-2.45775900	-1.03857300
H	6.08269700	-0.72924500	-1.32985600
H	4.59935200	-2.32526000	0.83005000
H	5.92131600	-1.18058800	1.11310800
N	2.88539100	-0.45327200	-0.32650000
Cu	1.58968000	-1.75366200	0.36139400
Cu	1.40812300	0.80059800	-0.05708100
C	-0.05707900	3.21234600	-0.66570100
C	-0.00952300	2.49554600	1.76253700
C	-1.15917500	3.47309900	2.05602100
C	-1.19464800	4.65737400	1.10254000
C	-1.21499100	4.16710200	-0.33611300
H	-2.11569200	2.93033500	1.97789000
H	-1.07977000	3.81716700	3.10054800
H	-2.08197400	5.27520600	1.30383700
H	-0.32823600	5.31707500	1.27069900
H	-2.16373400	3.62970600	-0.51536400
H	-1.19592500	5.01750700	-1.03734200
N	-0.01177200	2.08654000	0.31977800
C	-4.12097000	-0.23561400	0.25938900
C	-2.91114500	-0.99547400	-1.80835200
C	-3.68639900	0.04208000	-2.63534100
C	-5.04170700	0.38383300	-2.03296200
C	-4.88211800	0.80320800	-0.57888400
H	-3.08198700	0.96433200	-2.68983200
H	-3.80154200	-0.32008000	-3.67085000
H	-5.51763400	1.19098700	-2.60889600
H	-5.72474600	-0.47798300	-2.10955600
H	-4.31466400	1.75070300	-0.54212100
H	-5.86605000	1.00354800	-0.12224100
N	-2.82491600	-0.59717900	-0.37475100
Cu	-1.42344500	0.75125800	-0.06033600
Li	-1.81889400	-1.91952500	0.61261400
C	-2.13730100	-4.20580400	2.06674800
C	-1.60066400	-4.53726700	0.82128300
C	-0.45627200	-3.88186100	0.35255400
C	0.21014700	-2.87512900	1.08852700
C	-0.34935000	-2.59880800	2.35920200
C	-1.49597000	-3.23787500	2.84345200
H	-3.04158000	-4.69472100	2.42729800
H	-2.08719800	-5.29428000	0.20465100
H	-0.08564100	-4.15227200	-0.64006700
H	0.10943400	-1.83152200	2.98925400

H -1.89875900 -2.97114800 3.82149200

C -1.49015000 -1.08762700 -2.35676600

H -0.96110600 -0.12947600 -2.26673400

H -0.89776900 -1.84503700 -1.81700700

H -1.49412500 -1.36882800 -3.42029400

C -3.52660400 -2.39216500 -2.04202900

H -3.29768500 -2.73529900 -3.06172200

H -3.10563500 -3.13481700 -1.34568700

H -4.61541800 -2.42320700 -1.93893300

C -5.03622600 -1.44494000 0.54464400

H -5.85196400 -1.14877200 1.22064100

H -5.50618800 -1.87111800 -0.34628100

H -4.47565800 -2.25303300 1.04235800

C -3.83608800 0.37808600 1.62723600

H -3.29698200 -0.32731800 2.28235800

H -3.23074800 1.29050600 1.53667100

H -4.77240100 0.64854000 2.13637200

C 1.30960600 3.12959800 2.24819500

H 2.18087700 2.59402600 1.84763700

H 1.41674400 4.18491900 1.97713000

H 1.36235400 3.08185500 3.34591900

C -0.21367300 1.24440500 2.61703100

H 0.61983800 0.53594900 2.49647200

H -0.27771100 1.51415600 3.68131200

H -1.13971100 0.71700000 2.34874700

C 1.25899900 4.00134900 -0.77691900

H 1.21632500 4.67944200 -1.64200200

H 1.48348500 4.61669600 0.09867700

H 2.10692700 3.31686500 -0.92838000

C -0.31413400 2.63689600 -2.05560300

H 0.48352900 1.94034200 -2.35729200

H -1.27356900 2.10024000 -2.09655200

H -0.35397600 3.44288800 -2.80230700

C 3.49786900 -0.32965500 2.05359400

H 2.82715900 0.51120900 2.28106900

H 2.94872500 -1.26715500 2.23928000

H 4.33419300 -0.28598800 2.76644700

C 4.72569000 1.10374700 0.48670000

H 3.98480800 1.91294500 0.41149800

H 5.34878000 1.29640100 1.37272600

H 5.38510200 1.17612700 -0.38458100

C 3.74376700 0.54805600 -2.49770000

H 4.74063400 0.87803900 -2.18929800

H 3.79299600 0.34795000 -3.57844100

H 3.05226400 1.38904300 -2.34243600

C 1.99685300 -1.16623000 -2.49595500

H 1.56723200 -2.07050000 -2.03507700

H 1.22081300 -0.38710600 -2.49825400

H 2.23824200 -1.40402000 -3.54222800

5-e)

Cartesian Coordinates

C -2.93310300 1.41850300 -1.68580100

C -3.91284100 1.03477300 0.61014500

C -4.95752200 0.09481200 -0.01046100

C -5.26843300 0.42411100 -1.46270700

C	-3.98672900	0.47245000	-2.28136800
H	-4.56431000	-0.93648600	0.04590300
H	-5.87739900	0.11706500	0.59704300
H	-5.95472300	-0.32665400	-1.88111900
H	-5.80220900	1.38591500	-1.53000700
H	-3.55649000	-0.54372300	-2.32813900
H	-4.20172300	0.77236900	-3.32043500
N	-2.69279800	1.08461500	-0.24915700
Cu	-1.17701100	2.03535600	0.50270400
Cu	-1.51232700	-0.49507500	0.00052800
C	-0.34640500	-2.48730300	1.73929800
C	-0.66777000	-3.15688300	-0.68173800
C	-1.97317800	-3.86575300	-0.28756100
C	-1.98110900	-4.35387900	1.15195500
C	-1.65729100	-3.20638000	2.09661300
H	-2.80488600	-3.15132700	-0.42512200
H	-2.15702700	-4.70156600	-0.98243300
H	-2.96430000	-4.77925300	1.40066200
H	-1.25976700	-5.17688500	1.28054400
H	-2.48462200	-2.47770900	2.06245200
H	-1.59863400	-3.56551900	3.13741200
N	-0.34960000	-2.07067900	0.29819500
C	3.12668400	0.23924400	-1.90130900
C	4.04879600	-0.55472000	0.31243000
C	5.28063600	0.34072500	0.09035300
C	5.59971400	0.56368700	-1.37787100
C	4.37394800	1.12128600	-2.08050700
H	5.08692800	1.32263500	0.56136600
H	6.14518300	-0.09371300	0.61864200
H	6.44784300	1.25619100	-1.48235600
H	5.92086900	-0.37835700	-1.85082500
H	4.15526100	2.12275800	-1.66497700
H	4.56736000	1.26505100	-3.15617400
N	2.88662100	-0.05542500	-0.46619500
Cu	1.25252300	-1.05757800	-0.14938000
Li	2.06369000	1.47175200	0.42156600
C	3.02692800	3.84096800	1.85990000
C	2.45604000	4.19971900	0.63772200
C	1.18280400	3.73154300	0.29611400
C	0.42283000	2.88382000	1.13614900
C	1.03301000	2.56477500	2.37086400
C	2.30041700	3.03193800	2.73458300
H	4.02445800	4.18792200	2.12725700
H	3.00837600	4.83637200	-0.05482500
H	0.77349000	4.02747200	-0.67429500
H	0.50870500	1.90878600	3.07156200
H	2.73184100	2.74670200	3.69518500
C	3.69703400	-0.46980700	1.79888900
H	3.40309600	0.55054300	2.09826500
H	2.87864000	-1.15402300	2.06468500
H	4.56504500	-0.74019200	2.41766600
C	4.42891800	-2.02190900	0.04245000
H	4.90688800	-2.17422900	-0.93140700
H	5.13384300	-2.38452400	0.80635200
H	3.53547500	-2.66240800	0.07560700
C	3.24325900	-1.00119800	-2.80498800
H	3.21124300	-0.70938700	-3.86596000
H	4.17140100	-1.56300100	-2.65465700
H	2.40679500	-1.69123300	-2.61940800
C	1.92767000	1.03331600	-2.41479100
H	1.00201300	0.44420500	-2.34515300
H	1.77696000	1.96748000	-1.84671200
H	2.06510600	1.32091800	-3.46733000
C	0.46041900	-4.18639000	-0.86448400
H	0.62457900	-4.82753300	0.00592000
H	0.22715100	-4.84912800	-1.71083100

H	1.41036500	-3.67886200	-1.08955000
C	-0.88260100	-2.53209900	-2.05711500
H	0.01109000	-1.98626100	-2.39742700
H	-1.10206400	-3.31115500	-2.80115600
H	-1.73128400	-1.83306300	-2.05169800
C	0.83544300	-3.39100200	2.14757200
H	0.97396000	-3.34745500	3.23802400
H	0.69256300	-4.44675900	1.89492000
H	1.77174900	-3.06052200	1.67767200
C	-0.22622500	-1.23406000	2.60760900
H	0.75676400	-0.75423400	2.48690200
H	-0.98960200	-0.48503300	2.36064400
H	-0.34106200	-1.49567700	3.66971800
C	-1.62581000	1.23782800	-2.45162500
H	-0.87794000	1.98271900	-2.13614400
H	-1.19427800	0.24041600	-2.29447000
H	-1.79159500	1.36571500	-3.53135100
C	-3.34382400	2.88207100	-1.94221800
H	-2.71544500	3.56698400	-1.35429200
H	-3.20105100	3.12255700	-3.00620500
H	-4.39021000	3.10215000	-1.71151600
C	-4.53836500	2.41247000	0.89500700
H	-5.06973200	2.84672300	0.04384000
H	-5.26944400	2.32584300	1.71226900
H	-3.76345400	3.12652200	1.20936700
C	-3.53301200	0.47590100	1.97712000
H	-3.10906900	-0.53380300	1.89230200
H	-2.79707200	1.12068300	2.48395100
H	-4.42064600	0.40842400	2.62217500

5-f)

Cartesian Coordinates

C	-3.02025900	-1.18689000	1.73726100
C	-3.95268200	-0.84894200	-0.58381300
C	-4.91358800	0.21356500	-0.02900300
C	-5.25367300	0.00164400	1.43913700
C	-3.98478400	-0.11734000	2.27149500
H	-4.43345400	1.20197600	-0.14646900
H	-5.83190500	0.23291700	-0.63906900
H	-5.86843900	0.83609700	1.80733600
H	-5.87474800	-0.90072600	1.55931600
H	-3.46307000	0.85624100	2.26736500
H	-4.23203600	-0.33878000	3.32308000
N	-2.74487300	-0.94888000	0.28824500
Cu	-1.30309800	-2.03640600	-0.41386300
Cu	-1.45316900	0.53602100	-0.01710800
C	-0.20445300	2.40952600	-1.81868300
C	-0.42705600	3.15677500	0.58122900
C	-1.72251700	3.89748800	0.20974000
C	-1.77375100	4.33533900	-1.24485500
C	-1.50622000	3.15269800	-2.16360700
H	-2.57156600	3.21893500	0.40890800
H	-1.84915700	4.76454700	0.87871000
H	-2.75657100	4.77325900	-1.47260200
H	-1.04195300	5.13900200	-1.42549500
H	-2.35220800	2.44948100	-2.08953100

H -1.46374600 3.48193800 -3.21523500

N -0.18702300 2.02417800 -0.36871200

C 4.10368300 0.21501100 -0.33748700

C 3.11578200 -0.42565900 1.88032600

C 3.68654500 0.85737400 2.50444000

C 4.92586700 1.36623400 1.77826200

C 4.66720500 1.50272600 0.28262700

H 2.90416900 1.63615200 2.46470900

H 3.90894200 0.68837800 3.57170400

H 5.23399900 2.33581900 2.19625500

H 5.77486600 0.68554700 1.95491600

H 3.93380700 2.31233700 0.11670300

H 5.59134300 1.80018500 -0.24151100

N 2.91130300 -0.25854700 0.41625100

Cu 1.34659200 0.88944500 0.07064900

Li 2.06285500 -1.75918600 -0.43824400

C 2.79970900 -4.04467900 -1.78381900

C 2.22483800 -4.37897700 -0.55629100

C 0.97411400 -3.85878100 -0.20310900

C 0.23879300 -2.98744000 -1.04091200

C 0.84847600 -2.69947000 -2.28456600

C 2.09708700 -3.20994100 -2.65567600

H 3.78224200 -4.42787900 -2.05691600

H 2.75974700 -5.03234600 0.13423200

H 0.56750400 -4.12842300 0.77561400

H 0.34309400 -2.02753300 -2.98386100

H 2.53260300 -2.94193800 -3.61933100

C 1.75686400 -0.71720100 2.50967900

H 1.03225100 0.08088400 2.29680700

H 1.33421600 -1.65955900 2.12260700

H 1.83798500 -0.81478800 3.60217000

C 4.00113100 -1.62857400 2.26787600

H 3.86358800 -1.86376400 3.33358300

H 3.71889300 -2.52491600 1.69361500

H 5.07253100 -1.46342800 2.12065700

C 5.22960000 -0.83234700 -0.46774400

H 5.93245900 -0.52781100 -1.25718600

H 5.82186700 -0.96894600 0.44148300

H 4.82218100 -1.81585300 -0.75085300

C 3.67290100 0.50762000 -1.77338300

H 3.29961100 -0.40287500 -2.27448800

H 2.87874700 1.26358900 -1.81334400

H 4.51927900 0.88183800 -2.36711200

C 0.74230300 4.15168600 0.68020200

H 0.88347500 4.76663100 -0.21282600

H 0.57277300 4.84115800 1.52028800

H 1.68457000 3.61670800 0.87120900

C -0.61249900 2.60257100 1.99008500

H 0.28840700 2.07820800 2.34203900

H -0.81623600 3.42092900 2.69537600

H -1.46189200 1.90693900 2.04049300

C 0.98339200 3.28747200 -2.27416100

H 1.17711600 3.12452400 -3.34455000

H 0.80274500 4.36136900 -2.15347600

H 1.90248900 3.04522300 -1.72661700

C -0.13217100 1.13363400 -2.65966900

H 0.85305600 0.65140200 -2.57571800

H -0.88684200 0.39537700 -2.35738700

H -0.29659300 1.37043100 -3.72126800

C -1.70413100 -1.09124800 2.50295100

H -1.03271900 -1.92222800 2.23407700

H -1.17424100 -0.15349400 2.29430300

H -1.88439100 -1.14227300 3.58671500

C -3.56600700 -2.59092100 2.06551900

H -3.43451200 -2.79299100 3.13884400

H -4.63135400 -2.72081900 1.85414500

H -3.01237200 -3.35984200 1.50751700

C -4.69417200 -2.18262000 -0.79027300

H -5.27451100 -2.51165700 0.07590000

H -5.40317500 -2.08573500 -1.62557900

H -3.98174600 -2.98099200 -1.04331300

C -3.52015300 -0.41232400 -1.97951100

H -3.00602300 0.55709100 -1.95463600

H -2.84374200 -1.15095500 -2.43946500

H -4.39623100 -0.30508600 -2.63488500

5-g)

Cartesian Coordinates

C 3.12060800 -1.16142100 -1.68135000

C 3.95783900 -0.86913400 0.68573000

C 4.96257100 0.18069700 0.18773600

C 5.36297600 -0.02381400 -1.26655500

C 4.13121000 -0.10731900 -2.15732500

H 4.49826300 1.17778800 0.29508800

H 5.85327000 0.17459900 0.83775600

H 6.01194800 0.80008900 -1.59773300

H 5.96854500 -0.93894100 -1.36865700

H 3.62678800 0.87573400 -2.16454200

H 4.42245600 -0.32151800 -3.19907800

N 2.79098200 -0.93850300 -0.24298600

Cu 1.29995500 -1.99303300 0.40973400

Cu 1.55807700 0.58722000 0.00724500

C 0.47521800 3.21336200 -0.64666400

C 0.27950900 2.48982100 1.76626000

C -0.85829400 3.50295000 1.97191600

C -0.77672800 4.68985200 1.02195800

C -0.65903700 4.22670800 -0.42289900

H -1.82129800 2.98559800 1.81796700

H -0.85192000 3.84892700 3.01909500

H -1.66562800 5.32664500 1.14091600

H 0.08189500 5.32899900 1.28385400

H -1.60817400 3.75146400 -0.72989100

H -0.51210300 5.09165700 -1.09065800

N 0.34939200 2.08336100 0.32597500

C -3.02394900 -0.39319600 -1.91642300

C -4.02277500 0.29224100 0.29459300

C -5.15148800 -0.73185700 0.07470500

C -5.44243800 -1.00096300 -1.39143700

C -4.16184300 -1.41290400 -2.09747800

H -4.85477000 -1.68328500 0.55572600

H -6.05994300 -0.38974700 0.59718900

H -6.20301300 -1.78944800 -1.49000100

H -5.87492600 -0.10639100 -1.86757400

H -3.82882800 -2.38515000 -1.68777300

H -4.34061300 -1.57311200 -3.17353600

N -2.81357800 -0.09033700 -0.47769500

Cu -1.23615900 0.99243600 -0.12212400

Li -1.90684600 -1.58476600 0.37834800

C -2.75731800 -4.07750600 1.82414200

C -2.20415500 -4.38289000 0.57987700

C -0.97486300 -3.83026200 0.20535400

C -0.24220800 -2.94976800 1.03546400

C	-0.83171700	-2.68708800	2.29375000
C	-2.05661300	-3.23546800	2.68823700
H	-3.72225500	-4.49036100	2.11629600
H	-2.73709800	-5.04393600	-0.10494200
H	-0.57962900	-4.08482200	-0.78237700
H	-0.32669800	-2.01043600	2.98929700
H	-2.47455200	-2.99051600	3.66574500
C	-3.67883000	0.25821700	1.78449900
H	-3.23958100	-0.70641700	2.09198600
H	-2.97666400	1.05727400	2.05972400
H	-4.58625600	0.39719100	2.39011800
C	-4.54906300	1.71075700	0.01314600
H	-5.29886800	1.99922400	0.76592700
H	-3.72523700	2.43819200	0.06006200
H	-5.02412100	1.81235800	-0.96832900
C	-3.29993800	0.83138500	-2.80984300
H	-3.15233800	0.57417500	-3.86978800
H	-4.32201100	1.21467700	-2.71600300
H	-2.61709500	1.65656900	-2.56328200
C	-1.74405700	-1.03844400	-2.44497500
H	-0.89271500	-0.34441700	-2.39446500
H	-1.47157400	-1.94443400	-1.87663700
H	-1.86371400	-1.34711500	-3.49370600
C	1.58371900	3.08194500	2.33876200
H	1.59197100	2.96948400	3.43283100
H	2.46785000	2.56561700	1.94237500
H	1.70643800	4.15094900	2.13575000
C	-0.02093400	1.24803800	2.60712500
H	0.82382700	0.54463200	2.60921000
H	-0.22137200	1.53560200	3.64979500
H	-0.90086500	0.70599100	2.23375900
C	1.83676400	3.92916800	-0.61784700
H	1.93817700	4.56312600	-1.51109300
H	1.98326500	4.57892600	0.24971400
H	2.65610700	3.19525200	-0.63300200
C	0.34118900	2.66351400	-2.06467700
H	1.21015800	2.04850600	-2.34026500
H	-0.56417100	2.05016400	-2.18243700
H	0.27961800	3.49005400	-2.78728700
C	1.84067900	-1.01093000	-2.49817400
H	1.12185300	-1.81111700	-2.25974200
H	1.34804900	-0.04767300	-2.30504100
H	2.06141100	-1.06607600	-3.57416300
C	3.64007300	-2.57449600	-2.00691200
H	3.02494500	-3.33515500	-1.50465900
H	3.57516400	-2.74964900	-3.09100800
H	4.68334200	-2.74480600	-1.72554200
C	4.66572800	-2.21755000	0.91104400
H	5.28515700	-2.54289000	0.07046800
H	5.33191600	-2.14423200	1.78322200
H	3.92898500	-3.00792900	1.11460900
C	3.46122600	-0.44004300	2.06373200
H	2.95067300	0.53168400	2.02364700
H	2.76091900	-1.18045800	2.48401000
H	4.30532200	-0.34211600	2.76136600

5-h)

Cartesian Coordinates

C	3.18746300	-0.93686800	-1.74021500
C	3.99065200	-0.66674500	0.63791400
C	4.90321700	0.48453700	0.18934600
C	5.32236800	0.37512000	-1.27016900
C	4.10583800	0.21624800	-2.17174500
H	4.35585800	1.43310800	0.33510800
H	5.79040000	0.52723800	0.84282800
H	5.89514200	1.26665500	-1.56490800
H	6.00853600	-0.47666000	-1.40524500
H	3.51687000	1.15097500	-2.15025400
H	4.41934800	0.06430800	-3.21801100
N	2.83362200	-0.79208100	-0.29806300
Cu	1.43232500	-1.96522400	0.34577200
Cu	1.47639600	0.62715600	-0.00415700
C	0.19794100	3.18760300	-0.58070500
C	0.17247100	2.42316700	1.82545600
C	-0.93859100	3.44323200	2.12642400
C	-0.91196200	4.64713700	1.19842300
C	-0.92089200	4.18915200	-0.25019100
H	-1.91699600	2.94522800	2.02843800
H	-0.85465700	3.76187200	3.17880300
H	-1.77940100	5.29367400	1.39641700
H	-0.02536600	5.27088400	1.39569300
H	-1.88914500	3.70136700	-0.46511100
H	-0.84546700	5.05360100	-0.93003000
N	0.16609500	2.03572500	0.37580300
C	-4.10098900	0.03203400	0.24250500
C	-2.95223800	-0.74334200	-1.85247400
C	-3.61567100	0.40082900	-2.63501600
C	-4.93390200	0.85053000	-2.01980100
C	-4.74876400	1.18103100	-0.54533100
H	-2.92252800	1.25995000	-2.64994300
H	-3.76197200	0.09704900	-3.68529100
H	-5.31991600	1.72871300	-2.55785200
H	-5.70049400	0.06775400	-2.14153400
H	-4.09193600	2.06544400	-0.45710800
H	-5.71367000	1.45394400	-0.08557400
N	-2.83795900	-0.41538500	-0.40348700
Cu	-1.33468700	0.80983700	-0.04232800
Li	-1.95721000	-1.84787300	0.53760400
C	-2.50822800	-4.14893900	1.92093600
C	-1.97444000	-4.48490500	0.67545300
C	-0.77093500	-3.91139000	0.24966900
C	-0.04164600	-2.98482000	1.03018700
C	-0.60462700	-2.69957600	2.29729900
C	-1.80813700	-3.25968200	2.73970900
H	-3.45628500	-4.57358100	2.24901100
H	-2.50770500	-5.18052700	0.02605700
H	-0.40107200	-4.18089000	-0.74340100
H	-0.10070800	-1.98831200	2.95774700
H	-2.20949800	-2.99058000	3.71767200
C	-1.54314800	-0.95699700	-2.39818000
H	-0.91070700	-0.07164100	-2.25193300
H	-1.04498000	-1.80481000	-1.89804600
H	-1.56596900	-1.17755200	-3.47565200
C	-3.69948900	-2.06198600	-2.14785100
H	-3.49192900	-2.38506000	-3.17860500
H	-3.36079100	-2.86869300	-1.47821500
H	-4.78733900	-1.99072900	-2.05596300
C	-5.13133100	-1.09662400	0.45802000
H	-5.91514900	-0.76144000	1.15323900
H	-5.64003200	-1.42046300	-0.45432500
H	-4.65477900	-1.98426600	0.90492500
C	-3.77222800	0.53889100	1.64385500
H	-3.31196700	-0.25162600	2.26132200
H	-3.07939700	1.39015900	1.60878100

H	-4.68267900	0.87188200	2.16259400
C	1.51059900	3.00825200	2.33057600
H	1.61262600	2.83102200	3.41128600
H	2.37159100	2.54547400	1.83238200
H	1.59199200	4.09134200	2.18553100
C	-0.09667500	1.17025100	2.65976700
H	0.71370800	0.43377700	2.55899800
H	-0.18252500	1.43315400	3.72427600
H	-1.03268100	0.67847000	2.35941300
C	1.55382800	3.91126700	-0.64290800
H	1.55854600	4.62140400	-1.48304700
H	1.79789600	4.48061100	0.25811100
H	2.36533600	3.18742500	-0.81107300
C	-0.05805300	2.67766000	-1.99547000
H	0.74122300	2.00320400	-2.33513100
H	-1.01687400	2.14347600	-2.06406100
H	-0.09867700	3.52102300	-2.69966300
C	1.90508100	-0.86768400	-2.56177500
H	1.25547400	-1.73307000	-2.35699100
H	1.33140700	0.04224000	-2.33881800
H	2.13567600	-0.86521200	-3.63701100
C	3.82784800	-2.28816300	-2.11027900
H	3.28410800	-3.11488100	-1.63107800
H	3.77381900	-2.43454000	-3.19918900
H	4.88338300	-2.37439400	-1.83655500
C	4.81123400	-1.95885300	0.80815500
H	5.46956600	-2.18662900	-0.03478200
H	5.45586100	-1.87133300	1.69508400
H	4.14432000	-2.81965500	0.95975700
C	3.46058600	-0.34575000	2.03300300
H	2.85369800	0.56842300	2.03504400
H	2.84152000	-1.17003900	2.42382600
H	4.29464700	-0.19148000	2.73261700

Structure optimization for 7

7-a)

Cartesian Coordinates

C	4.01225100	-0.56508800	0.64861900
C	3.20166500	-0.86244200	-1.71574700
C	4.03253300	-2.15100400	-1.83079800
C	5.25619400	-2.14130000	-0.92739100
C	4.84555100	-1.85036900	0.50774300
H	3.38721800	-3.00131700	-1.54612500
H	4.32644900	-2.31146800	-2.88202200
H	5.77510500	-3.10959000	-0.98298100
H	5.98597000	-1.39244400	-1.27673900
H	4.23532600	-2.69285100	0.87960200
H	5.73322900	-1.78934000	1.15972400
N	2.86791400	-0.55255900	-0.30038900
Cu	1.41868200	-1.69101700	0.33919700
C	0.12603600	2.54183400	1.64446900
C	0.38990800	3.35266600	-0.70995600
C	1.69977300	4.06084300	-0.31273200
C	1.73639900	4.44914600	1.15890300
C	1.44221400	3.23856700	2.03485300
H	2.54483800	3.37302700	-0.51467900
H	1.86673700	4.94376600	-0.95255400
H	2.71903300	4.87243000	1.41441400
H	1.00580200	5.24990100	1.35835000
H	2.27291500	2.51438700	1.92649600
H	1.42260600	3.52416100	3.10032700
N	0.08501700	2.23540800	0.20149800
C	-3.17542600	-0.24371500	-1.80945700
C	-4.24280300	0.32771400	0.38705600
C	-5.43030700	-0.58625100	0.03310800
C	-5.65056800	-0.71173400	-1.46683200
C	-4.36392600	-1.16201000	-2.14214100
H	-5.22375800	-1.59358400	0.44166300
H	-6.34521200	-0.22710600	0.53387900
H	-6.46002200	-1.42727200	-1.67373700
H	-5.98819600	0.24990000	-1.88681800
H	-4.12076900	-2.18274900	-1.79207800
H	-4.49734500	-1.22875600	-3.23513800
N	-3.02256300	-0.04661800	-0.35328000
Li	-2.17933500	-1.57394000	0.47031600
C	-2.57309800	-3.89219800	1.81373900
C	-2.03302900	-4.17580300	0.55733400
C	-0.83788800	-3.56864500	0.15557200
C	-0.12746100	-2.65116900	0.96451200
C	-0.70028900	-2.41504300	2.23585400
C	-1.88988300	-3.01685300	2.66036200
H	-3.51146700	-4.34747400	2.12851700
H	-2.55055700	-4.86158700	-0.11480800
H	-0.45524200	-3.80960200	-0.83996200
H	-0.21030000	-1.71813600	2.92191100
H	-2.29569700	-2.78752200	3.64667000
C	-3.94883400	0.14758700	1.87839700
H	-3.67437700	-0.89262200	2.12319300
H	-3.12729700	0.80403900	2.20823200
H	-4.82880100	0.39230800	2.49103500
C	-4.66945400	1.80225900	0.22282000
H	-5.44608800	2.07170800	0.95535600
H	-3.81388900	2.47756400	0.39277100

H -5.07313200 2.03094400 -0.76946300

C -3.30725100 1.05828100 -2.63126300

H -3.21094100 0.85322500 -3.70867400

H -4.26400100 1.57096900 -2.48618100

H -2.51263800 1.77809400 -2.37035000

C -1.89773100 -0.92182900 -2.30797500

H -1.00483500 -0.31132900 -2.08857200

H -1.74509400 -1.90824500 -1.84050000

H -1.92162100 -1.08246800 -3.39578400

C -0.73350900 4.39993900 -0.85791500

H -0.87121400 5.02001100 0.03442300

H -0.52508500 5.08369500 -1.69556300

H -1.69657700 3.90639200 -1.06611500

C 0.58507800 2.75373800 -2.10440700

H -0.32259600 2.22827000 -2.44576900

H 0.81850300 3.52882200 -2.84876100

H 1.41730000 2.03082800 -2.13330400

C -1.05551000 3.38115500 2.18048000

H -1.10058500 3.32990900 3.27970400

H -0.99723200 4.44197200 1.91380500

H -2.01487600 2.99974800 1.79413300

C 0.03892400 1.20372500 2.38476900

H -0.95111000 0.73639700 2.24492800

H 0.78595900 0.47488700 2.03127800

H 0.18794000 1.33015000 3.46782400

C 3.46910900 -0.52717700 2.07851800

H 2.94298200 0.41730100 2.28660400

H 2.77236500 -1.35804800 2.27726900

H 4.29305700 -0.60455500 2.80298100

C 4.92851200 0.66826200 0.53018500

H 5.60579500 0.72419300 1.39565000

H 5.55780100 0.66852600 -0.36541700

H 4.33047700 1.59351900 0.51628500

C 3.93101200 0.27905000 -2.45389800

H 4.98917000 0.36859400 -2.18784300

H 3.89154500 0.11627300 -3.54132100

H 3.45884200 1.25122700 -2.24562000

C 1.89388000 -1.08114600 -2.47907000

H 1.30315700 -1.90565900 -2.04834400

H 1.26375300 -0.17621800 -2.47261600

H 2.09383700 -1.32724300 -3.53224000

Li 1.54100400 0.92239000 -0.11632500

Li -1.55965700 1.26154300 -0.16222400

7-b)

Cartesian Coordinates

C -3.96485400 -0.51555600 -0.64210500

C -3.17519200 -0.76290800 1.73948900

C -4.09532200 -1.98346900 1.90360600

C -5.31726100 -1.92363000 1.00146700

C -4.88210900 -1.73419200 -0.44251300

H -3.51244400 -2.88695000 1.65018900

H -4.39427200 -2.08276400 2.96078700

H -5.90804800 -2.84610400 1.10119100

H -5.98714500 -1.10533800 1.31298200

H -4.32754000 -2.63339300 -0.76524700

H -5.75947200 -1.64713700 -1.10524700

N -2.82508400 -0.52366000 0.31404200

Cu -1.40148600 -1.70753600 -0.28814400

C -0.11631700 2.50086900 -1.71527300

C -0.28747800 3.35316200 0.62348600

C -1.66573400 3.96471200 0.29864700

C -1.83333800 4.30244400 -1.17665500

C -1.49776600 3.09757000 -2.04582700

H -2.45111100 3.23674000 0.58617000

H -1.84391400 4.85715600 0.92200100

H -2.86352100 4.63534900 -1.37364600

H -1.18838300 5.15506200 -1.44362800

H -2.27097300 2.32108200 -1.88735300

H -1.55338600 3.36284900 -3.11547100

N 0.01696300 2.22179900 -0.27123100

C 4.27631100 0.16591100 -0.38076000

C 3.12663500 -0.42488500 1.76771800

C 3.79189000 0.78429800 2.44898100

C 5.11959600 1.15812200 1.80570100

C 4.93426200 1.37313400 0.31033900

H 3.10767400 1.65077200 2.36763700

H 3.91989000 0.59087000 3.52742800

H 5.52534200 2.06737600 2.27310400

H 5.86758200 0.36924100 1.98846800

H 4.28283500 2.25509800 0.15884900

H 5.89748700 1.61038600 -0.17214100

N 3.03692600 -0.24286200 0.30486400

Li 2.25796600 -1.79211300 -0.50848200

C 2.52295900 -4.02403100 -1.77387400

C 1.98568100 -4.28877300 -0.51180100

C 0.81423300 -3.64309400 -0.09832700

C 0.11902000 -2.71290500 -0.90630800

C 0.68567800 -2.49903200 -2.18509600

C 1.85798100 -3.12985300 -2.61711800

H 3.44526500 -4.50645100 -2.09519000

H 2.49107100 -4.98537000 0.15833700

H 0.44190700 -3.86176200 0.90632600

H 0.20722400 -1.79337300 -2.87033000

H 2.26236300 -2.91359900 -3.60684100

C 1.69272600 -0.54018200 2.28993500

H 1.09792000 0.36177800 2.07053600

H 1.16786000 -1.39941400 1.83930900

H 1.66583100 -0.67174700 3.38191300

C 3.83822800 -1.71709800 2.22529500

H 3.68334000 -1.88700400 3.30213000

H 3.43302200 -2.59499200 1.69497300

H 4.92027600 -1.70331000 2.05622800

C 5.32508200 -0.95521300 -0.55113700

H 6.11987300 -0.64539000 -1.24742200

H 5.81524000 -1.24110700 0.38558800

H 4.85997700 -1.86408600 -0.96884700

C 3.88950900 0.58525300 -1.79966900

H 3.42656500 -0.25010200 -2.35322100

H 3.17392100 1.42267200 -1.79656800

H 4.76612900 0.91525500 -2.37602100

C 0.77373200 4.47180000 0.65381000

H 0.79779100 5.07777200 -0.25804500

H 0.58856400 5.16064100 1.49289500

H 1.77656100 4.03857000 0.79060400

C -0.34451300 2.81107800 2.05253600

H 0.64353600 2.45226500 2.38149900

H -0.66537700 3.58845600 2.76135200

H -1.05484500 1.97340300 2.15684700

C 0.96778500 3.41729500 -2.32740500

H 1.01208500 3.28632300 -3.41993800

H 0.79270900 4.48339800 -2.14507800

H	1.96076300	3.17685200	-1.91897400	H	-5.43361000	0.86074800	0.29859400
C	0.02343500	1.15777700	-2.44063200	C	-3.81528800	0.45482600	2.43596000
H	1.05856300	0.77998300	-2.38628000	H	-4.84762600	0.64567000	2.12591200
H	-0.62795400	0.37784000	-2.01172400	H	-3.83312400	0.30609000	3.52603900
H	-0.22876200	1.24800200	-3.50817100	H	-3.23917300	1.37224800	2.23795000
C	-3.41653400	-0.59397200	-2.06908100	C	-1.88658800	-1.05318100	2.51052000
H	-2.81454100	0.29050600	-2.32591800	H	-1.37324900	-1.94580800	2.11826500
H	-2.78929000	-1.48795500	-2.21835900	H	-1.17818700	-0.21021300	2.45899500
H	-4.24300200	-0.64440800	-2.79318500	H	-2.10157100	-1.23124500	3.57444200
C	-4.80409000	0.77674400	-0.59320400	Li	-1.47785100	0.93779000	0.10188500
H	-4.15434700	1.66635100	-0.62389800	Li	1.58614000	1.08984300	0.02682500
H	-5.47576700	0.82913300	-1.46327500				

7-c)

Cartesian Coordinates

C	4.09621700	-0.37066700	0.72665000	C	-1.16527600	3.53217100	1.88198400
C	3.35147100	-0.64547300	-1.66742500	C	-1.11936000	4.75588800	0.97652200
C	4.29747300	-1.84823800	-1.80995900	C	-0.98871900	4.33604800	-0.48154100
C	5.49781700	-1.76542600	-0.87881100	H	-2.10137900	2.97720800	1.67547300
C	5.03361600	-1.57728600	0.55782500	H	-1.21426300	3.83357800	2.94223100
H	3.72515800	-2.76170600	-1.56878400	H	-2.02486600	5.36464600	1.11620400
H	4.62414500	-1.94398600	-2.85924200	H	-0.27632400	5.40669100	1.26048000
H	6.10525200	-2.67848500	-0.96338300	H	-1.91734700	3.81377300	-0.78381700
H	6.16076900	-0.93752200	-1.17991300	H	-0.90721500	5.22155000	-1.13440400
H	4.48504300	-2.48200100	0.87480000	N	0.17380900	2.24961800	0.22010700
H	5.89797200	-1.47452800	1.23534700	C	-3.04170000	-0.44670600	-1.82964000
N	2.97868200	-0.41116700	-0.25017800	C	-4.17874900	0.06391900	0.33733800
Cu	1.55642500	-1.60352900	0.33689900	C	-5.17821600	-1.07949800	0.07144700
C	0.19983800	3.38589200	-0.71713900	C	-5.37371700	-1.36855200	-1.40952300
C	0.02714300	2.58572600	1.64882100	C	-4.02933200	-1.60024800	-2.08424000

H	-4.79459000	-1.99631500	0.56104600
H	-6.14488000	-0.85673800	0.55395200
H	-6.02312600	-2.24650300	-1.54262100
H	-5.90350400	-0.53156900	-1.89249000
H	-3.58755100	-2.53520500	-1.68770400
H	-4.15957200	-1.75646900	-3.16865900
N	-2.90971600	-0.15155800	-0.38613700
Li	-2.05477400	-1.68416800	0.45837900
C	-2.32100800	-4.02585600	1.77675200
C	-1.76254800	-4.27049600	0.52001100
C	-0.60444500	-3.59228200	0.12399400
C	0.05130600	-2.64027600	0.93972600
C	-0.53713600	-2.44536900	2.21103600
C	-1.69193500	-3.11634300	2.62917700
H	-3.23182500	-4.53676800	2.08693600
H	-2.23773600	-4.98158200	-0.15710700
H	-0.20772200	-3.80490400	-0.87260100
H	-0.08989700	-1.72563400	2.90271800
H	-2.11224700	-2.91634800	3.61578400
C	-3.87548400	0.06542200	1.83815500
H	-3.35494500	-0.85037500	2.16819900
H	-3.25148700	0.92668100	2.12087900
H	-4.80351700	0.12712900	2.42521700
C	-4.86307500	1.41844700	0.06166000
H	-5.67424600	1.59946400	0.78407900
H	-4.13689800	2.24032100	0.16416600
H	-5.30322400	1.49130400	-0.93827000
C	-3.44669200	0.75144300	-2.71783400
H	-3.21851900	0.54512600	-3.77495700
H	-4.51417200	0.99181100	-2.66626200
H	-2.89769700	1.66146400	-2.43076200
C	-1.66171000	-0.88589600	-2.32597300
H	-0.91997300	-0.07375600	-2.23686800

H	-1.27050600	-1.74999100	-1.76517700
H	-1.68877500	-1.17455200	-3.38692200
C	1.28061200	3.18764400	2.32173500
H	1.47424000	4.22688100	2.03517200
H	1.17945700	3.17176800	3.41815800
H	2.18135400	2.60571500	2.06739000
C	-0.24188600	1.27126900	2.38737400
H	0.60607900	0.57328500	2.28111500
H	-0.40547300	1.43668900	3.46282900
H	-1.13625500	0.75255800	2.00658500
C	1.51102000	4.20170500	-0.72072500
H	1.54662700	4.88138800	-1.58634800
H	1.64576900	4.81791400	0.17472800
H	2.38461200	3.53266900	-0.79266400
C	0.07625000	2.80638400	-2.12725800
H	0.92873800	2.14633500	-2.36421800
H	-0.84602700	2.21601500	-2.25138600
H	0.05408900	3.59829000	-2.89003500
C	3.50531200	-0.42925700	2.13699600
H	2.86235400	0.44291800	2.34118200
H	2.90259600	-1.33881400	2.29125500
H	4.30468300	-0.42644700	2.89245000
C	4.91698400	0.93361800	0.67815900
H	4.25353500	1.81339200	0.66888800
H	5.55932200	1.01442800	1.56781500
H	5.57442400	1.00957800	-0.19402300
C	3.97044600	0.58659600	-2.35704100
H	4.98581600	0.81746200	-2.01993200
H	4.02295800	0.43099700	-3.44506100
H	3.35382300	1.48366100	-2.18246500
C	2.07576200	-0.95497400	-2.45204200
H	1.56810300	-1.85330900	-2.06569600
H	1.35993800	-0.11700700	-2.40208100

H 2.30189600 -1.12744100 -3.51461300

Li 1.67696200 1.04905900 -0.04492600

Li -1.41899300 1.12957500 -0.18361800

7-d)

Cartesian Coordinates

C 4.11413500 -0.27982200 0.59840100

C 3.24525400 -0.51027900 -1.75718100

C 4.17340800 -1.71692900 -1.96897900

C 5.42287100 -1.65505100 -1.10467100

C 5.03516100 -1.48768300 0.35653100

H 3.60882500 -2.63001800 -1.70861100

H 4.43975100 -1.79981400 -3.03619700

H 6.01918900 -2.57004900 -1.23522600

H 6.07362300 -0.82643400 -1.42927400

H 4.50118300 -2.39637900 0.68706900

H 5.93413900 -1.39885300 0.98952500

N 2.94423400 -0.29402300 -0.31886000

Cu 1.56689300 -1.50602400 0.33868200

C -0.15329400 3.35207800 -0.68662400

C 0.16144300 2.56734200 1.66880100

C -0.87736600 3.59608000 2.14876100

C -0.94769000 4.81622500 1.24256100

C -1.19171500 4.37566300 -0.19244900

H -1.86940700 3.11170800 2.16215800

H -0.65990100 3.89237200 3.18906500

H -1.75068000 5.49048500 1.57523000

H -0.01758800 5.40406800 1.31113600

H -2.19052100 3.90469300 -0.24771300

H -1.21673000 5.24572400 -0.87019000

N -0.00781900 2.21815600 0.24345600

C -4.15791700 -0.04479800 0.31482600

C -3.02281700 -0.91059700 -1.74824000

C -3.86067900 0.05648400 -2.60199300

C -5.20347300 0.38123800 -1.96698400

C -4.98209600 0.91509500 -0.56081100

H -3.29389700 0.99718800 -2.72120900

H -3.99427600 -0.35801500 -3.61541200

H -5.74375400 1.12154900 -2.57526700

H -5.84694000 -0.51385700 -1.94324000

H -4.43296700 1.87183400 -0.63299100

H -5.94375000 1.14028000 -0.06965800

N -2.90544500 -0.46423600 -0.34282100

Li -2.10426000 -1.94611500 0.60087700

C -2.09595000 -4.10242100 2.03324300

C -1.53221000 -4.40210000 0.79091200

C -0.44586500 -3.65849200 0.31477700

C 0.13816800 -2.59304400 1.03903900

C -0.44642600 -2.34876000 2.30446600

C -1.53765700 -3.07358500 2.79661900

H -2.95499100 -4.66213600 2.40120600

H -1.95280900 -5.20429100 0.18308100

H -0.05357800 -3.91031600 -0.67448700

H -0.04968600 -1.54512100 2.93075600

H -1.96160500 -2.82840700 3.77127300

C -1.60735900 -0.94428500 -2.32511800

H	-1.15265000	0.05840600	-2.32989200
H	-0.95033500	-1.60939000	-1.73989200
H	-1.59768700	-1.30237200	-3.36543100
C	-3.58690600	-2.33912500	-1.93221400
H	-4.64769000	-2.42450200	-1.67263900
H	-3.48362800	-2.66740100	-2.97814100
H	-3.03753200	-3.07182000	-1.31634100
C	-5.06346000	-1.20985000	0.77443800
H	-5.88328000	-0.84503900	1.41280700
H	-5.52283600	-1.75772000	-0.05556900
H	-4.49154300	-1.93942700	1.37460800
C	-3.77425100	0.70741900	1.58870000
H	-3.19360600	0.06613300	2.27355200
H	-3.17084200	1.60141400	1.36043500
H	-4.66301200	1.05273400	2.13707400
C	1.56531700	3.09482500	2.05362900
H	1.74619100	4.12357300	1.72311400
H	1.70286600	3.08058400	3.14608600
H	2.37125100	2.47530200	1.62668700
C	-0.04147600	1.27587200	2.46426500
H	0.66406300	0.49110300	2.13898400
H	0.10751200	1.43146000	3.54337000
H	-1.06054400	0.87948700	2.33129100
C	1.16666700	4.08397200	-1.01448700
H	1.03054100	4.79908600	-1.84075800
H	1.57603900	4.64477600	-0.16738300
H	1.94085000	3.36547500	-1.33684800
C	-0.65624800	2.79079800	-2.01644600
H	0.05198500	2.05306100	-2.43120300
H	-1.63853600	2.30127100	-1.90366700
H	-0.78236100	3.58344100	-2.76840200
C	3.60528600	-0.37067000	2.03973600
H	2.98256300	0.49641400	2.30874200
H	3.00708000	-1.28138100	2.20553400
H	4.45000400	-0.39713900	2.74374200
C	4.94405900	1.01817300	0.52871900
H	4.29002200	1.90408000	0.52528200
H	5.60465900	1.09706000	1.40497400
H	5.58690000	1.08219200	-0.35563300
C	3.83555800	0.72950700	-2.45681500
H	4.85294000	0.97509100	-2.13801100
H	3.86718900	0.57720000	-3.54611300
H	3.20978200	1.61791100	-2.26898700
C	1.93057100	-0.79980500	-2.48021800
H	1.44416100	-1.70735300	-2.08917800
H	1.21661500	0.03518500	-2.37132600
H	2.09711000	-0.94404400	-3.55791100
Li	1.61155400	1.15619300	-0.13159800
Li	-1.42156200	0.85442900	-0.07906800

7-e)

Cartesian Coordinates

C	3.11475900	-1.32186300	-1.62355200
C	4.01307000	-0.68348500	0.64215000
C	5.12095300	0.12883300	-0.04903700
C	5.48523000	-0.41424100	-1.42071300

C	4.23567300	-0.50062600	-2.28143400
H	4.76561100	1.16925400	-0.16687100
H	6.00798100	0.17144800	0.60445600
H	6.23424800	0.23338700	-1.89980700
H	5.95868400	-1.40517500	-1.32884800
H	3.85888900	0.52261600	-2.46117100
H	4.47009700	-0.92684200	-3.27088900
N	2.83909700	-0.83673600	-0.24885800
Cu	1.35081200	-1.79437500	0.53433200
C	0.04959300	2.59595900	1.62645800
C	0.64019200	3.29709000	-0.70352700
C	1.79428200	4.14649900	-0.13976800
C	1.53093900	4.63196100	1.27731300
C	1.20630100	3.44818900	2.17665100
H	2.70810300	3.52325100	-0.12980800
H	2.00308100	4.99584400	-0.81204300
H	2.40781500	5.17278000	1.66266000
H	0.70408000	5.36095800	1.28498200
H	2.10384500	2.81077300	2.25892600
H	0.97032300	3.78780300	3.19944300
N	0.25974300	2.20790300	0.21578000
C	-3.00725600	-0.17835100	-1.89033200
C	-4.23555100	0.43658100	0.20732300
C	-5.36625100	-0.53228900	-0.18748900
C	-5.48406900	-0.71663700	-1.69292400
C	-4.14257300	-1.14541400	-2.26791300
H	-5.15549500	-1.51742700	0.27043300
H	-6.32330900	-0.18858700	0.24001900
H	-6.25545900	-1.46555200	-1.92551200
H	-5.82280700	0.21822500	-2.16862100
H	-3.89182900	-2.14668800	-1.86961500
H	-4.20160000	-1.25144800	-3.36429300
N	-2.95672100	0.07578000	-0.43577000

Li	-2.21082600	-1.45000000	0.49869000
C	-2.81124900	-3.71104400	1.91608900
C	-2.22169500	-4.06573000	0.70078800
C	-0.97702600	-3.53355000	0.34561500
C	-0.26311500	-2.62644300	1.16408600
C	-0.89024300	-2.31336200	2.39170400
C	-2.12988700	-2.84074900	2.76921200
H	-3.78712900	-4.10755800	2.19426500
H	-2.73778600	-4.74832800	0.02437600
H	-0.55298100	-3.82977700	-0.61850300
H	-0.40189400	-1.61754000	3.07903400
H	-2.57567800	-2.55623900	3.72334900
C	-4.03586700	0.32061800	1.72118400
H	-3.76415300	-0.70460200	2.02559700
H	-3.24624600	1.00013000	2.07913400
H	-4.95809400	0.57152600	2.26521100
C	-4.70719200	1.88426200	-0.04839300
H	-5.08077500	2.04524700	-1.06577200
H	-5.52375100	2.15766900	0.63771900
H	-3.88664600	2.60065700	0.11606500
C	-3.11336400	1.08860000	-2.76724900
H	-2.95591700	0.84437700	-3.82924700
H	-4.08278100	1.59213400	-2.69259500
H	-2.34147400	1.82762600	-2.49069300
C	-1.67940400	-0.83208500	-2.27242100
H	-0.82516700	-0.17732100	-2.02743700
H	-1.52277000	-1.78846700	-1.74649200
H	-1.62168700	-1.04407500	-3.35047200
C	-0.52080400	4.22634000	-1.11885800
H	-0.88729400	4.85672200	-0.30149200
H	-0.21679600	4.90056000	-1.93463000
H	-1.37735000	3.63726100	-1.48867200
C	1.13390400	2.64881500	-1.99834600

H	0.35902500	1.99977800	-2.44064000
H	1.40153400	3.40377900	-2.75172800
H	2.04171700	2.04120600	-1.83633500
C	-1.27306900	3.34802000	1.91181000
H	-1.47885800	3.37768400	2.99320800
H	-1.26977800	4.38498300	1.55916900
H	-2.13737000	2.85163000	1.43998700
C	-0.02646600	1.30211900	2.44006100
H	-0.87201700	0.67036400	2.11519000
H	0.88852900	0.69993200	2.33599300
H	-0.16773200	1.50635900	3.51204300
C	1.84675900	-1.12574400	-2.45265300
H	1.02501700	-1.75786500	-2.07960500
H	1.50978100	-0.07809600	-2.43648300
H	2.02012200	-1.39343900	-3.50548000
C	3.45170300	-2.82286900	-1.72808700
H	2.70747300	-3.42102700	-1.17938100
H	3.43537100	-3.14587300	-2.78068600
H	4.43907400	-3.08024300	-1.33070800
C	4.59744100	-2.00311200	1.18155400
H	5.12257400	-2.59103100	0.42145400
H	5.32067500	-1.80266900	1.98725200
H	3.79554700	-2.63325700	1.59473900
C	3.56637500	0.10763300	1.86984600
H	3.16050700	1.09461300	1.59121000
H	2.79856600	-0.43906800	2.44102500
H	4.41207400	0.29595600	2.54685300
Li	1.57807200	0.74475600	-0.06285300
Li	-1.46346200	1.35070400	-0.21177800

7-f)

Cartesian Coordinates

C	-3.17902900	-1.11814700	1.63498400
C	-4.02708900	-0.49475400	-0.65413500
C	-5.09980600	0.38767700	0.00596300
C	-5.49973100	-0.09981900	1.38849400
C	-4.26222300	-0.22524900	2.26179900
H	-4.69574400	1.41263700	0.09936000
H	-5.97897900	0.45529200	-0.65593000
H	-6.21873500	0.59692100	1.84412300
H	-6.02185000	-1.06786600	1.31917600
H	-3.83577300	0.78241800	2.41778100
H	-4.52487800	-0.61283700	3.25999200
N	-2.86831000	-0.67967200	0.25145800
Cu	-1.42935300	-1.73846900	-0.49343600
C	0.12428800	2.53529100	-1.69302600
C	-0.39376600	3.29479100	0.62478600
C	-1.52960700	4.17508100	0.06688200
C	-1.29066500	4.62178400	-1.36648800
C	-1.01226100	3.41306000	-2.24780100
H	-2.46636400	3.58624600	0.09627000
H	-1.69066800	5.04594500	0.72461000
H	-2.16453600	5.17356700	-1.74329000
H	-0.44986400	5.33320100	-1.40789200
H	-1.92961100	2.80306600	-2.31507700
H	-0.77260400	3.72883100	-3.27767100

N	-0.08666500	2.16680800	-0.27647900
C	4.25714500	0.08973800	-0.25857700
C	3.00117400	-0.37404300	1.85333500
C	3.53323900	0.93645000	2.46090600
C	4.87127500	1.35361800	1.86468700
C	4.78940700	1.40239700	0.34421200
H	2.79369500	1.73860300	2.26667500
H	3.60638400	0.84856500	3.55817400
H	5.17195300	2.33518500	2.25964900
H	5.66029800	0.65146000	2.17992400
H	4.10621700	2.22324600	0.05317600
H	5.77315000	1.65162100	-0.08847100
N	2.98882000	-0.31251500	0.37822100
Li	2.24374300	-1.86559400	-0.44377100
C	2.56236500	-4.10479000	-1.67337100
C	1.89187300	-4.35346500	-0.47297600
C	0.70329000	-3.67389600	-0.18206600
C	0.12206700	-2.72409100	-1.05604100
C	0.82550000	-2.52043600	-2.26568100
C	2.01515600	-3.18947700	-2.57639500
H	3.49708500	-4.61545800	-1.90155300
H	2.30431500	-5.06557100	0.24273200
H	0.21870800	-3.88585500	0.77561700
H	0.44391000	-1.79647500	-2.99035400
H	2.52658700	-2.98459100	-3.51785100
C	1.55111300	-0.56327800	2.30057000
H	0.88765800	0.24508300	1.95053700
H	1.14003000	-1.51201700	1.91674000
H	1.45976800	-0.58287600	3.39685200
C	3.77068700	-1.56581000	2.46342800
H	3.52374900	-1.68024500	3.53032600
H	3.49415200	-2.50461100	1.95709000
H	4.85903700	-1.46574900	2.39879100
C	5.37752800	-0.97377200	-0.22333900
H	6.18770500	-0.70464200	-0.91861600
H	5.83581800	-1.10033200	0.76288500
H	4.98886700	-1.95741800	-0.53402700
C	3.96939900	0.31836300	-1.74360400
H	3.63621000	-0.61295000	-2.23429300
H	3.18783400	1.07427900	-1.90192400
H	4.86753800	0.66305100	-2.27658900
C	0.81702000	4.18092800	0.98326300
H	1.20094600	4.75665800	0.13464000
H	0.55794000	4.90243600	1.77387400
H	1.64532300	3.55998900	1.36224700
C	-0.88794700	2.71874900	1.95285800
H	-0.12783600	2.07334900	2.42068800
H	-1.12634100	3.51886800	2.66850600
H	-1.81584800	2.13196800	1.83346900
C	1.46033800	3.26242800	-1.99574000
H	1.74315700	3.13838900	-3.05272200
H	1.41450700	4.34126800	-1.80711200
H	2.28955200	2.87391200	-1.38480500
C	0.15166400	1.23043700	-2.49514000
H	1.00340600	0.58677300	-2.21262500
H	-0.76516100	0.64036100	-2.34344600
H	0.24701100	1.42511900	-3.57376900
C	-1.91031700	-0.96840400	2.47283500
H	-1.11989900	-1.65126700	2.12327100
H	-1.51539000	0.05774700	2.43715900
H	-2.10832600	-1.20290700	3.52916600
C	-3.59321700	-2.59743000	1.77168200
H	-2.87937000	-3.24526500	1.23901800
H	-3.59557500	-2.89687400	2.83132400
H	-4.59158700	-2.81363800	1.37728900
C	-4.67006700	-1.79865500	-1.16437100

H	-5.22863700	-2.34068100	-0.39413000
H	-5.37681200	-1.58469000	-1.98110800
H	-3.89650700	-2.47705400	-1.55461200
C	-3.53358800	0.24161800	-1.89768500
H	-3.08989500	1.21784400	-1.64102900
H	-2.78307800	-0.35155000	-2.44487500
H	-4.36293400	0.44709000	-2.58972000
Li	-1.54102200	0.84156900	0.04225500
Li	1.51172000	1.01879200	0.04622300

7-g)

Cartesian Coordinates

C	3.27324100	-0.82747100	-1.67810000
C	4.03421400	-0.92282200	0.72380200
C	5.23006000	-0.02748900	0.36367500
C	5.67329700	-0.19821000	-1.08072300
C	4.49541100	0.04616000	-2.00969600
H	4.93516800	1.02647900	0.52218300
H	6.06449500	-0.22957100	1.05536300
H	6.49020400	0.50034600	-1.31428500
H	6.08642400	-1.20742000	-1.23973400
H	4.19712300	1.10710400	-1.92071100
H	4.78564800	-0.10989800	-3.06184100
N	2.92498000	-0.72143000	-0.24110800
Cu	1.37349300	-1.75261400	0.27874300
C	0.29152100	3.41716200	-0.70234800
C	0.44736000	2.56931700	1.64286300
C	-0.54301000	3.65297200	2.10558400
C	-0.47761000	4.89974600	1.23663800
C	-0.67341400	4.51990700	-0.22212400
H	-1.56723300	3.23838600	2.05087700
H	-0.36166200	3.90273600	3.16500300
H	-1.24721800	5.62171100	1.54741500
H	0.48663900	5.41589300	1.37288400
H	-1.70782000	4.14655700	-0.34586300
H	-0.58495900	5.40644700	-0.87236500
N	0.30257500	2.25613900	0.20732300
C	-3.16209200	-0.31034300	-1.82495300
C	-4.11298700	0.36619900	0.38530100
C	-5.15218300	-0.76908400	0.28308200
C	-5.46287200	-1.16330000	-1.15437500
C	-4.17932900	-1.46444700	-1.91738900
H	-4.75528000	-1.65741000	0.81468000
H	-6.07636000	-0.48768700	0.81569200
H	-6.13008500	-2.03786600	-1.17151100
H	-6.01967800	-0.35532400	-1.65552400
H	-3.71908900	-2.38134200	-1.49721300
H	-4.40091600	-1.69403200	-2.97349800
N	-2.91164600	0.06766900	-0.41796900
Li	-2.17370000	-1.55134600	0.38320200
C	-2.64777000	-3.92068400	1.71297500
C	-2.10159100	-4.20577100	0.45972000
C	-0.90569300	-3.59843800	0.06208700
C	-0.19877500	-2.68216900	0.87568800
C	-0.77778600	-2.44299800	2.14360800
C	-1.97126400	-3.04274800	2.56165200

H	-3.58787300	-4.37554100	2.02306200
H	-2.61615000	-4.89201900	-0.21419000
H	-0.51894600	-3.83656900	-0.93268700
H	-0.29246800	-1.74345000	2.83052900
H	-2.38365800	-2.81149800	3.54474800
C	-3.67815500	0.46731500	1.84931200
H	-3.14410400	-0.43373400	2.19730700
H	-3.01302600	1.33150300	2.00611200
H	-4.54550100	0.59949700	2.51256500
C	-4.79069900	1.71455900	0.06880700
H	-5.52188000	1.97578000	0.84973500
H	-4.03730000	2.51702400	0.03262900
H	-5.32646200	1.72256000	-0.88604400
C	-3.63295900	0.82780600	-2.75620700
H	-3.50362100	0.53947100	-3.81083600
H	-4.68885500	1.08907400	-2.62841800
H	-3.04759200	1.74201600	-2.58554900
C	-1.82822800	-0.79083400	-2.40422200
H	-1.09434400	0.03193800	-2.44617600
H	-1.37818500	-1.60641000	-1.81317300
H	-1.94973800	-1.16707000	-3.43050300
C	1.86800800	3.00911100	2.07792200
H	2.06867300	4.06773000	1.87613200
H	2.01546900	2.85747500	3.15849300
H	2.65273400	2.43835900	1.55651400
C	0.12847300	1.28293800	2.41074700
H	0.81276700	0.45926300	2.14331700
H	0.20648000	1.42998400	3.49829000
H	-0.89593100	0.93645800	2.20089700
C	1.68283900	4.02694600	-0.97010400
H	1.63823300	4.78500900	-1.76774900
H	2.12105300	4.50925600	-0.08952600
H	2.38635900	3.24459300	-1.30073200
C	-0.22678400	2.94174200	-2.06114600
H	0.40356400	2.14495400	-2.48271400
H	-1.25896600	2.55867600	-1.99379300
H	-0.25196600	3.76515400	-2.78975100
C	2.09253300	-0.28181300	-2.48112000
H	1.17580200	-0.86554100	-2.29701400
H	1.89026500	0.77525100	-2.23913600
H	2.29775000	-0.31380200	-3.56075300
C	3.50139500	-2.26205700	-2.19051500
H	3.55733300	-2.27405700	-3.29012200
H	4.42532600	-2.71632500	-1.81842300
H	2.66596400	-2.91153200	-1.88620300
C	4.51234400	-2.38151500	0.87054900
H	5.07388700	-2.74951300	0.00572200
H	5.17374400	-2.47930400	1.74518500
H	3.65362600	-3.05317600	1.02029800
C	3.53760300	-0.51076300	2.10942000
H	3.17878900	0.52804600	2.12140300
H	2.71592900	-1.16143100	2.45208800
H	4.34728600	-0.58359900	2.85010700
Li	1.71959200	0.88029700	-0.04573800
Li	-1.34360500	1.23638000	-0.17643300

7-h)

Cartesian Coordinates

C	3.23516500	-0.54921100	-1.76186900
C	4.07407700	-0.73716800	0.60919200
C	5.22636500	0.21777500	0.26050200
C	5.62958000	0.13722100	-1.20302000
C	4.41454200	0.38586500	-2.08102200
H	4.90224500	1.25097100	0.48323700
H	6.08819000	0.00940100	0.91579100
H	6.41394000	0.87591000	-1.42432000
H	6.07175800	-0.84688900	-1.42741600
H	4.08089100	1.42868900	-1.92467700
H	4.67567700	0.29795800	-3.14857200
N	2.92681800	-0.53182600	-0.31168400
Cu	1.44571800	-1.65326900	0.23684500
C	-0.08575700	3.35068000	-0.58564600
C	0.36655300	2.50462200	1.72074300
C	-0.60732500	3.54731600	2.29871500
C	-0.70355300	4.79278800	1.43137200
C	-1.05283800	4.39410900	0.00710200
H	-1.60910200	3.08911300	2.37019300
H	-0.30727900	3.80768200	3.32824400
H	-1.46503500	5.47775000	1.83284700
H	0.24367500	5.35623000	1.45158800
H	-2.06883800	3.95761100	0.00816900
H	-1.09460800	5.28042400	-0.64825900
N	0.09370600	2.18833500	0.30428900
C	-4.11101800	0.13936000	0.33150900
C	-3.07131100	-0.76333300	-1.76930700
C	-3.87617300	0.25763800	-2.59171000
C	-5.18334500	0.64742400	-1.91953500
C	-4.89923700	1.15419200	-0.51437300
H	-3.26262800	1.16694500	-2.71657600
H	-4.05782000	-0.13799400	-3.60517200
H	-5.69747000	1.42207400	-2.50734400
H	-5.87363300	-0.21182400	-1.88749700
H	-4.29918600	2.07942800	-0.59093700
H	-5.83487100	1.42810400	0.00170100
N	-2.90227200	-0.34590600	-0.36099600
Li	-2.20188300	-1.90459400	0.52649300
C	-2.30539000	-4.10408500	1.94468800
C	-1.76786100	-4.42253300	0.69554500
C	-0.65677400	-3.72333500	0.20999900
C	-0.02395600	-2.68454200	0.93123300
C	-0.58463700	-2.41891900	2.20327800
C	-1.69868200	-3.10121900	2.70518900
H	-3.18280600	-4.62936900	2.31992500
H	-2.22761300	-5.20469600	0.09007300
H	-0.28419600	-3.98685900	-0.78354100
H	-0.15077300	-1.63182600	2.82592400
H	-2.10281800	-2.84181400	3.68454800
C	-1.67135600	-0.86291800	-2.37868800
H	-1.16036600	0.11220700	-2.36729100
H	-1.04081200	-1.58142800	-1.82601100
H	-1.70713300	-1.19391800	-3.42728300
C	-3.71557800	-2.15714600	-1.95954300
H	-3.65983200	-2.47290600	-3.01290500
H	-3.18918800	-2.93043600	-1.37342800
H	-4.77179300	-2.19038700	-1.66992300
C	-5.07088900	-0.97604400	0.80308300
H	-5.85623200	-0.57156300	1.46058300
H	-5.57589700	-1.49074000	-0.02179700

H	-4.52915300	-1.74121500	1.38717100
C	-3.64815300	0.85407100	1.60175100
H	-3.08426400	0.17239100	2.26146800
H	-3.00214100	1.71646500	1.36671900
H	-4.49874600	1.24221100	2.18113400
C	1.80555400	3.00592100	2.01355800
H	1.93871600	4.06950000	1.78423600
H	2.06083600	2.87492700	3.07658900
H	2.57080400	2.46725500	1.43071900
C	0.17508900	1.20382700	2.50573200
H	0.82483700	0.39524200	2.12757400
H	0.40203500	1.33289600	3.57464500
H	-0.86565900	0.85106000	2.42987600
C	1.23025100	4.04676400	-0.99366200
H	1.05438700	4.80742200	-1.77049400
H	1.73180000	4.54815900	-0.15857700
H	1.94092000	3.31399400	-1.41207300
C	-0.71791900	2.85286800	-1.88644300
H	-0.08513400	2.10022300	-2.38179900
H	-1.71184800	2.40765000	-1.70953200
H	-0.86856300	3.67705300	-2.59899500
C	2.01398400	-0.00737200	-2.50542900
H	1.12484100	-0.63153800	-2.32453800
H	1.77922800	1.03144200	-2.21500000
H	2.18961400	0.01547200	-3.59043100
C	3.49743500	-1.94676200	-2.35345700
H	2.68704900	-2.63580400	-2.06888100
H	3.53318500	-1.90069500	-3.45299200
H	4.44082600	-2.39087000	-2.02023300
C	4.61062000	-2.18209300	0.66722600
H	5.16283600	-2.48267300	-0.22931700
H	5.29847400	-2.29833700	1.51887600
H	3.78275900	-2.89424100	0.80323300
C	3.60950300	-0.41238300	2.02929800
H	3.21196800	0.60923800	2.10491600
H	2.82580600	-1.11085500	2.36679200
H	4.44721500	-0.49022900	2.73762000
Li	1.65494300	0.99738800	-0.04448200
Li	-1.36454800	0.88598200	-0.05348600

In situ deprotometallation using TMPCu and TMPLi

Control reaction 1: *In situ* study of TMPLi in C_6D_6

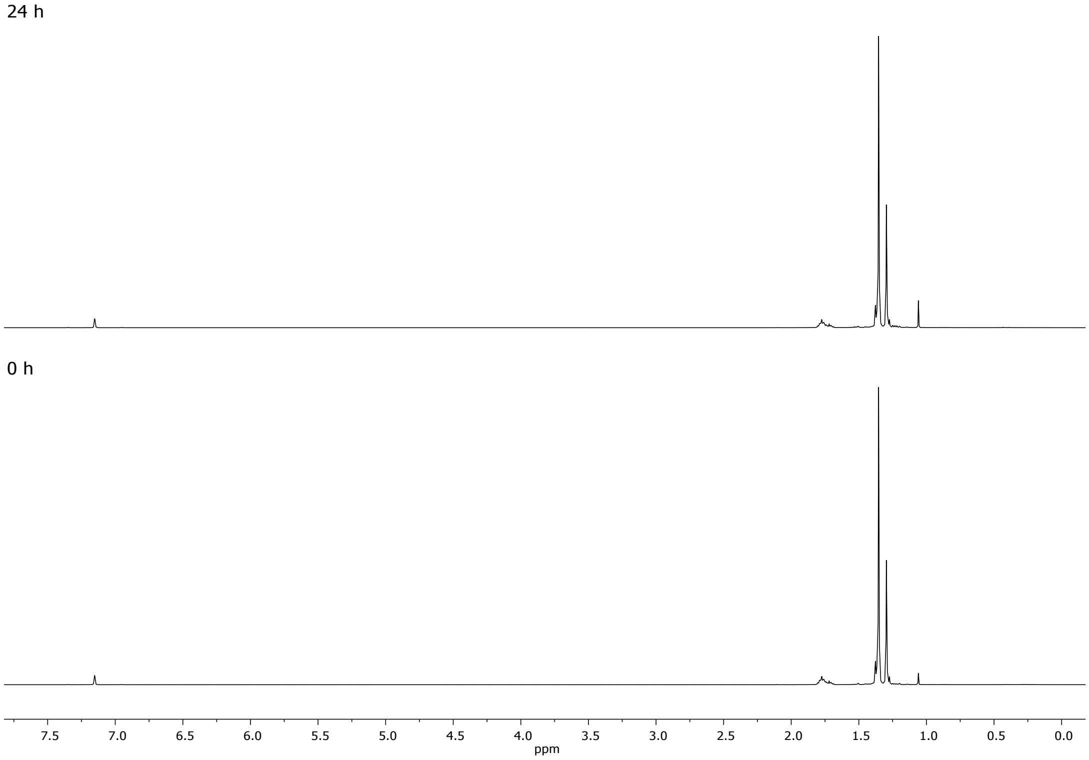

Figure S14a ^{1}H NMR spectrum of TMPLi before and after heating to 50°C for *ca.* 24 h.

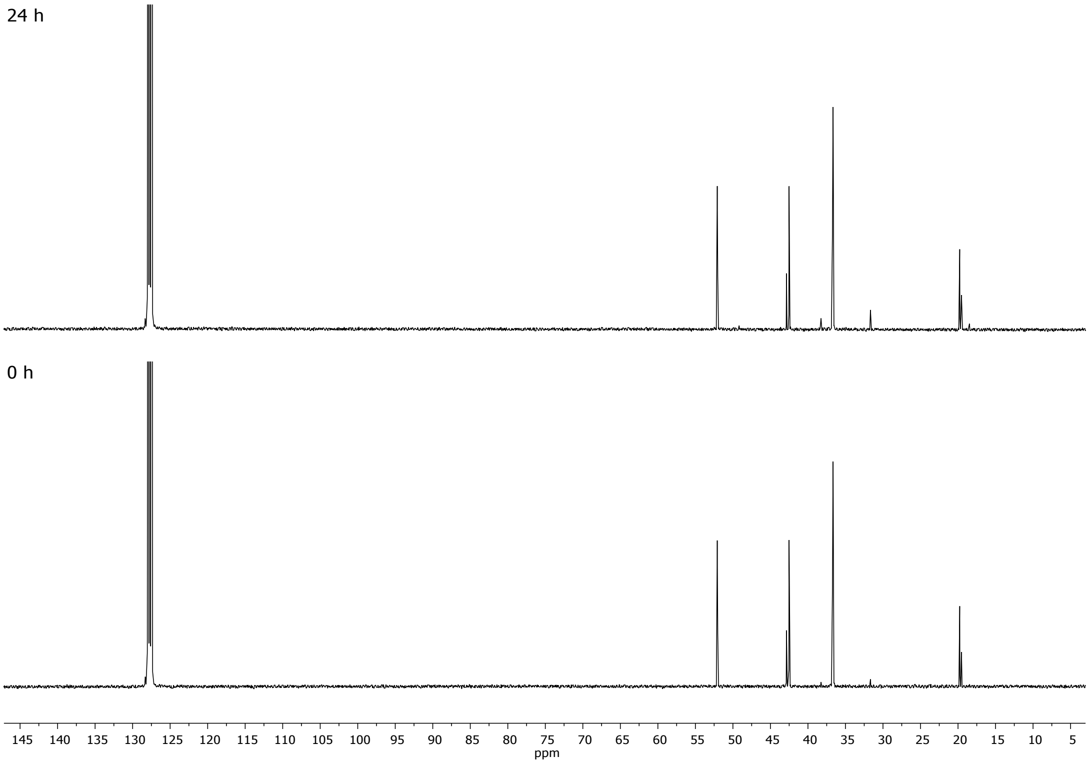

Figure S14b ^{13}C NMR spectrum of TMPLi before and after heating to 50°C for *ca.* 24 h.

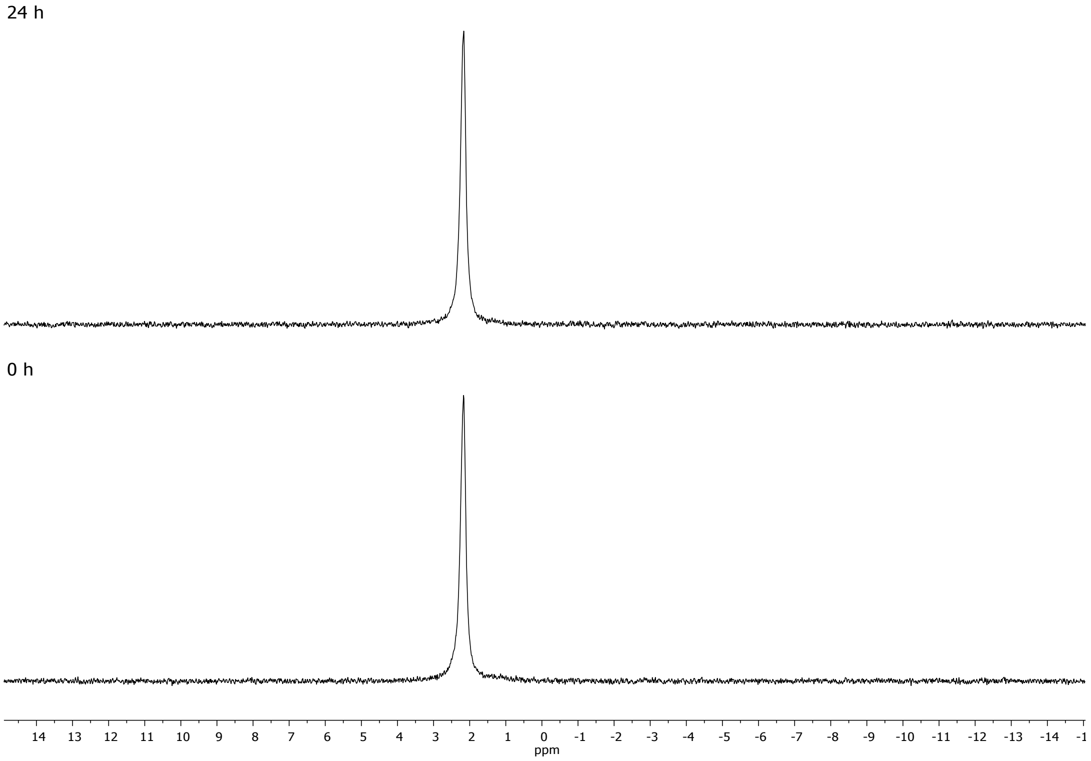

Figure S14c ^{7}Li NMR spectrum of TMPLi after heating to 50°C for *ca.* 24 h.

Control reaction 2: *In situ* study of TMPCu in C_6D_6

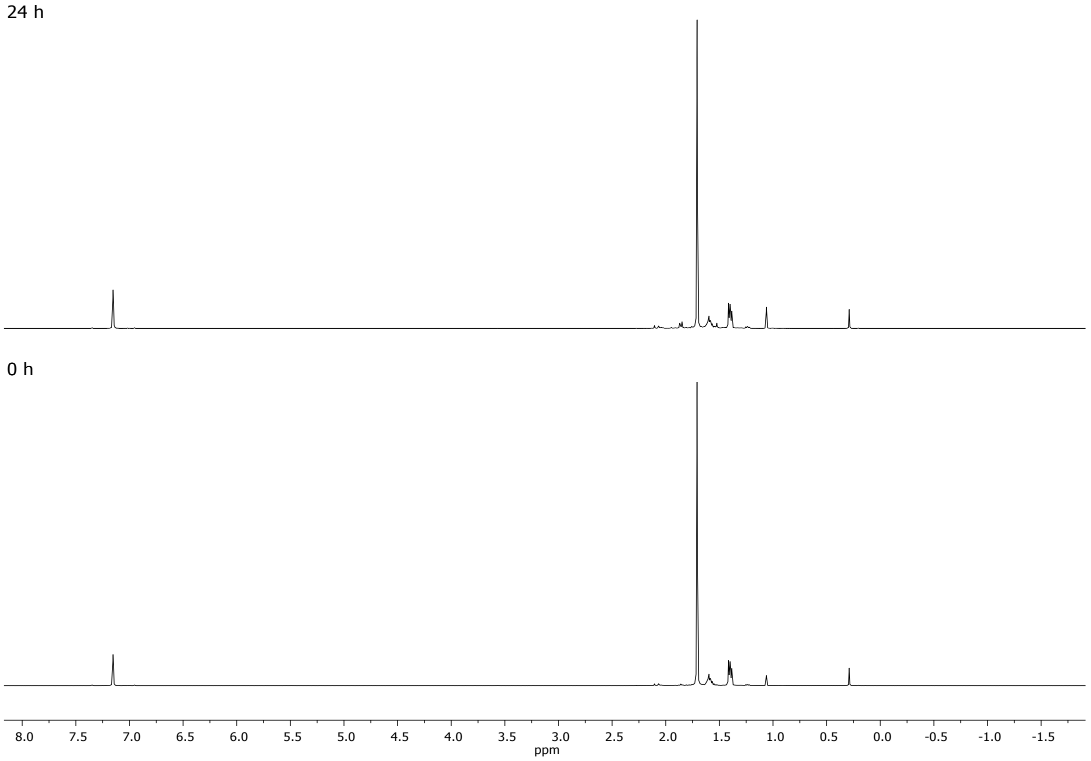

Figure S15a ^{1}H NMR spectrum of TMPCu before and after heating to 50°C for 24 h. In contrast to the reactivity shown by a mixture of TMPCu and TMPLi (main manuscript Figure 9 and below Figure S17) only a vanishingly small amount of **6** has formed as evidenced by the signals at δ 1.87, 1.85, 1.52 ppm.

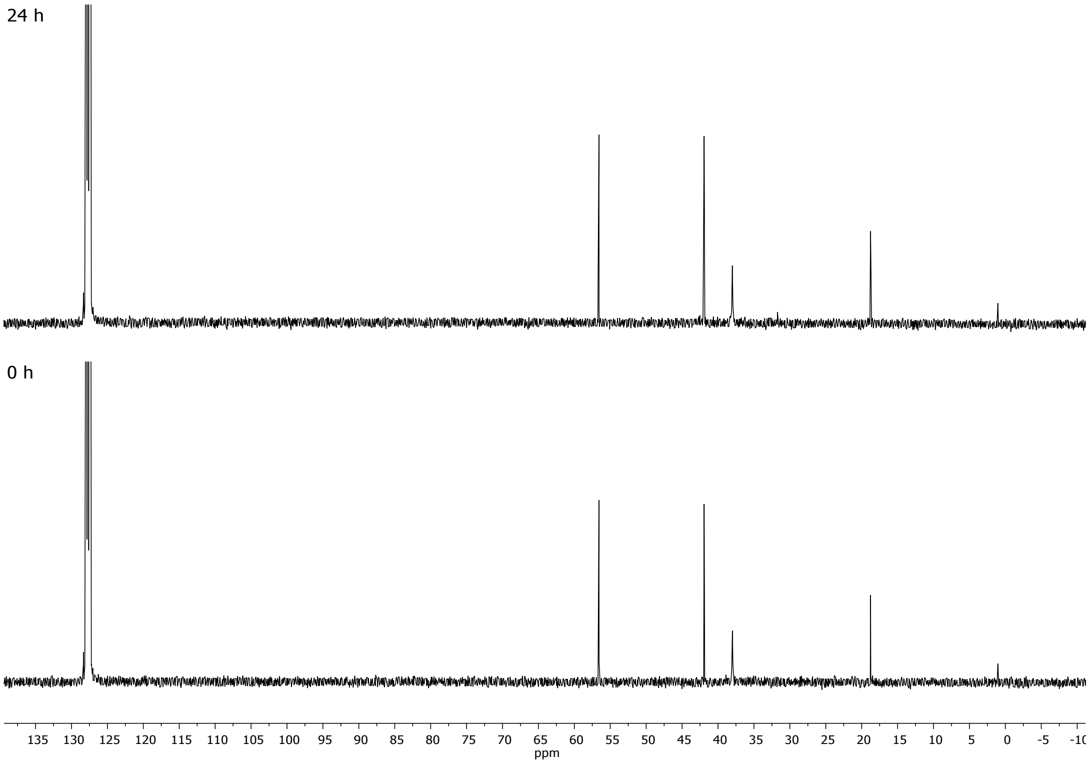

Figure S15b ^{13}C NMR spectrum of TMPCu before and after heating to 50°C for 24 h.

Control reaction 3: *In situ* study of TMPCu(μ-TMP)Li **2** in C_6D_6

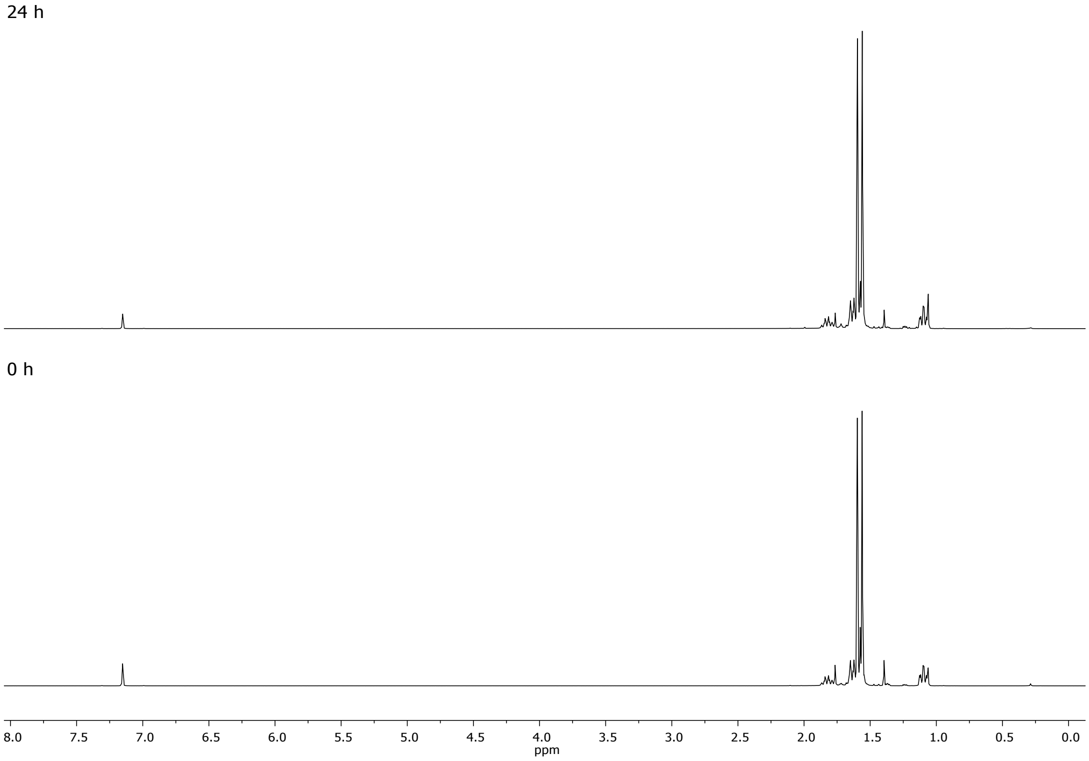

Figure S16a ^{1}H NMR spectrum of **2** before and after heating to 50°C for 24 h.

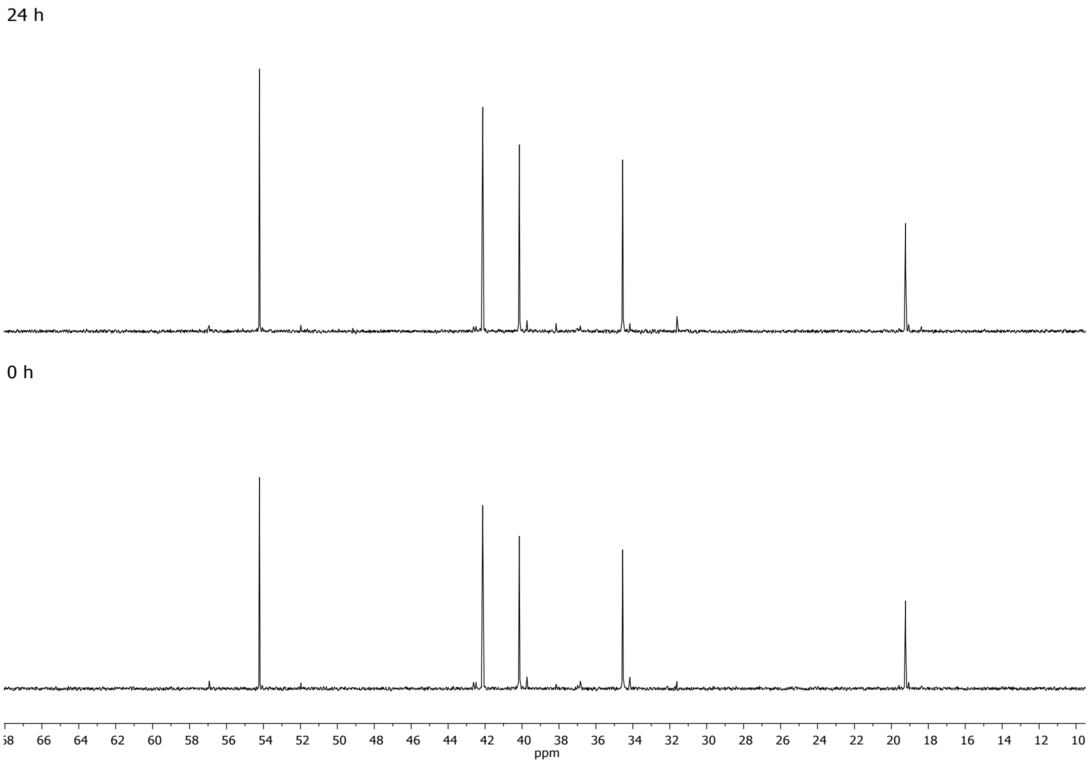

Figure S16b ^{13}C NMR spectrum of **2** before and after heating to 50°C for 24 h.

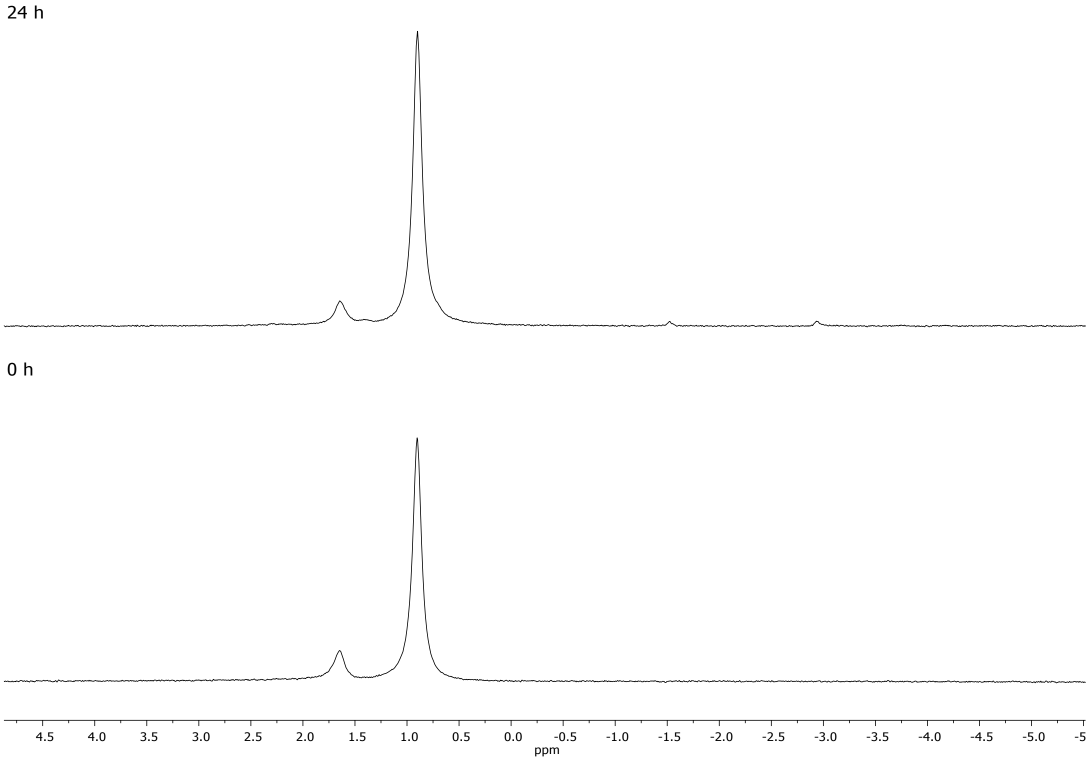

Figure S16c ^{7}Li NMR spectrum of **2** before and after heating to 50°C 24 h.

In situ reaction of TMPLi and TMPCu with C_6D_6

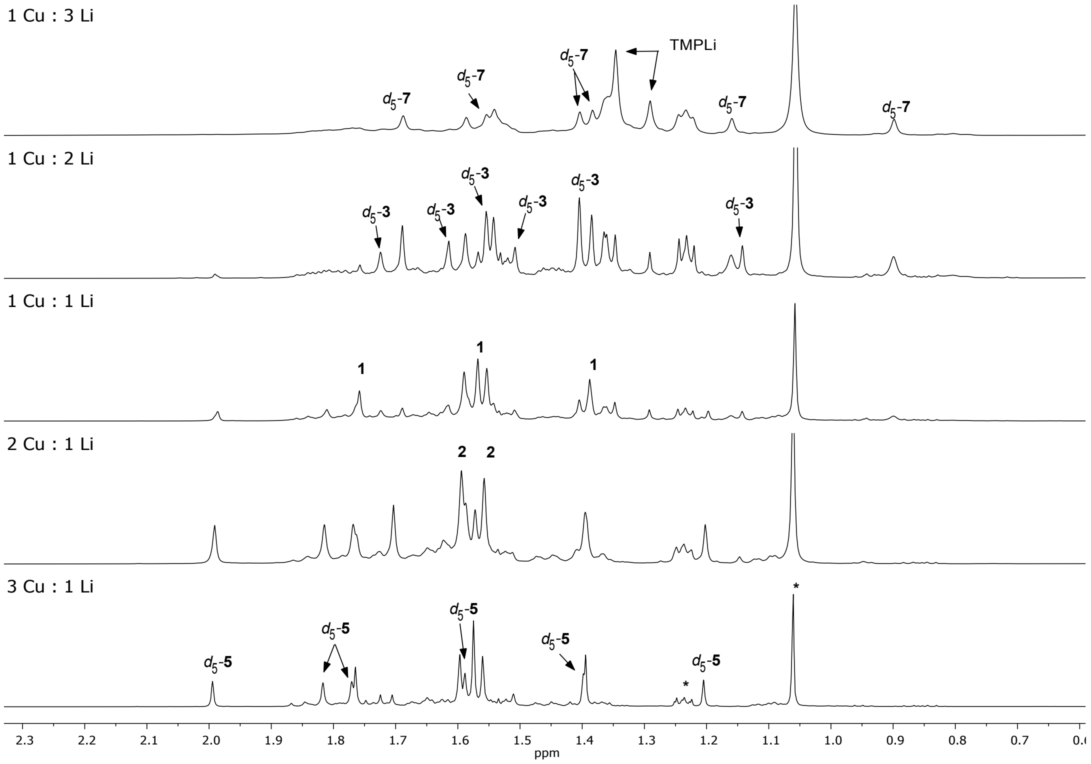

Figure S17a ^{1}H NMR spectra (500 MHz, 298 K, C_6D_6) of reaction mixtures containing TMPLi and TMPCu in the molar ratios specified and heated to 50°C for *ca*. 24 h. * = TMPH.

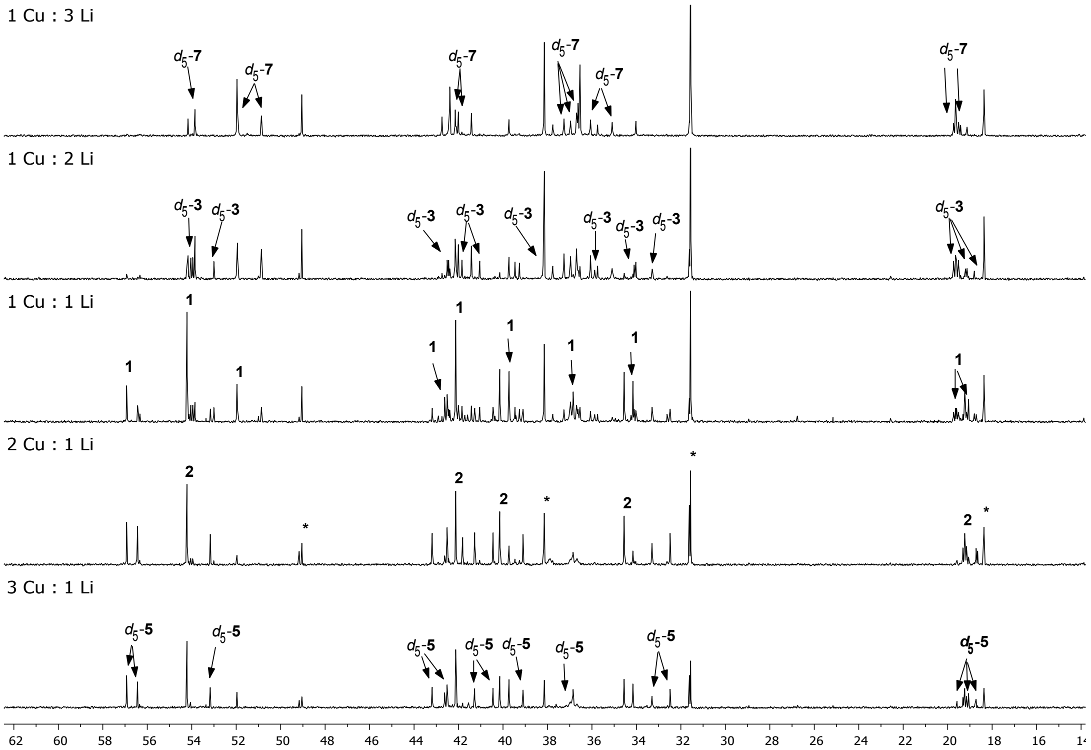

Figure S17b ^{13}C NMR spectra (125 MHz, 298 K, C_6D_6) of reaction mixtures containing TMPLi and TMPCu in the molar ratios specified and heated to 50°C for *ca*. 24 h. * = TMPH.

1:1 reaction of TMPLi and TMPCu with benzene

Experimental

TMPCu (203 mg, 1 mmol) and TMPLi (147 mg, 1 mmol) were combined in C_6H_6 (6 mL) and heated to 50°C for *ca.* 5 days, after which time the volatiles were removed *in vacuo* to leave a white residue.

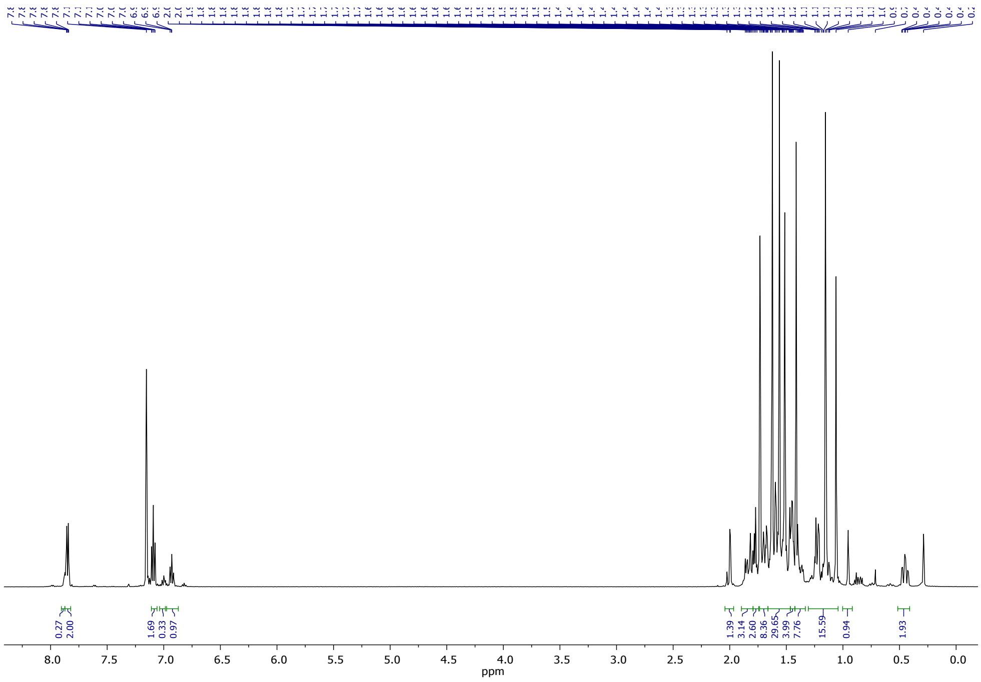

Figure S18a ^{1}H NMR spectrum (500 MHz, 298 K, C_6D_6) of an aliquot from the reaction mixture containing TMPCu and TMPLi (1:1) in C_6H_6, which was heated to 50°C for *ca.* 5 days.

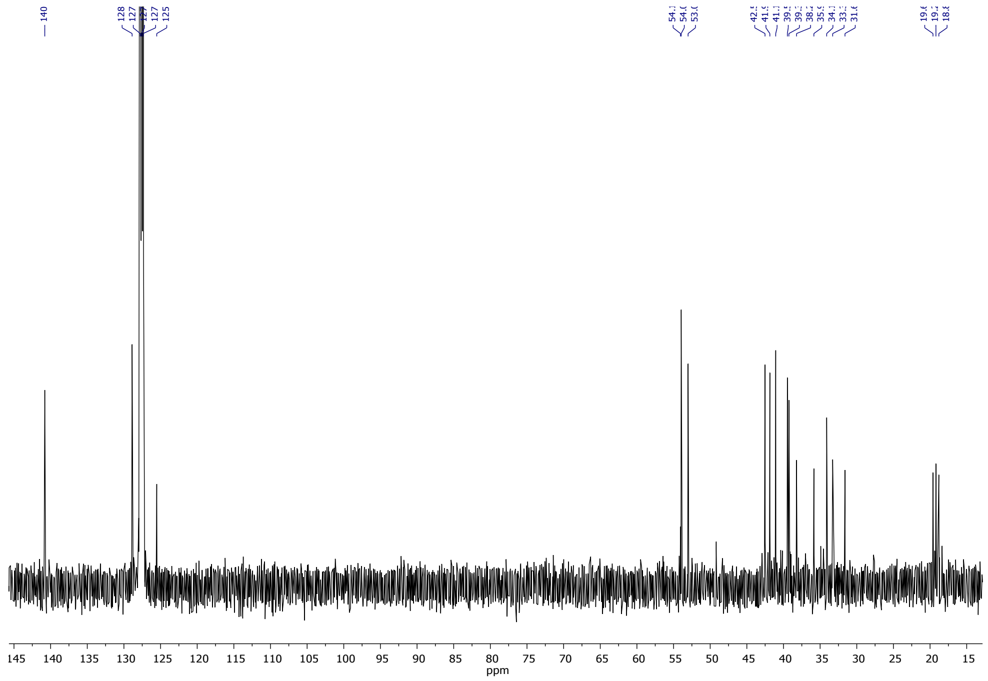

Figure S18b ^{13}C NMR spectrum (125 MHz, 298 K, C_6D_6) of an aliquot from the reaction mixture containing TMPCu and TMPLi (1:1) in C_6H_6, which was heated to 50°C for *ca.* 5 days.

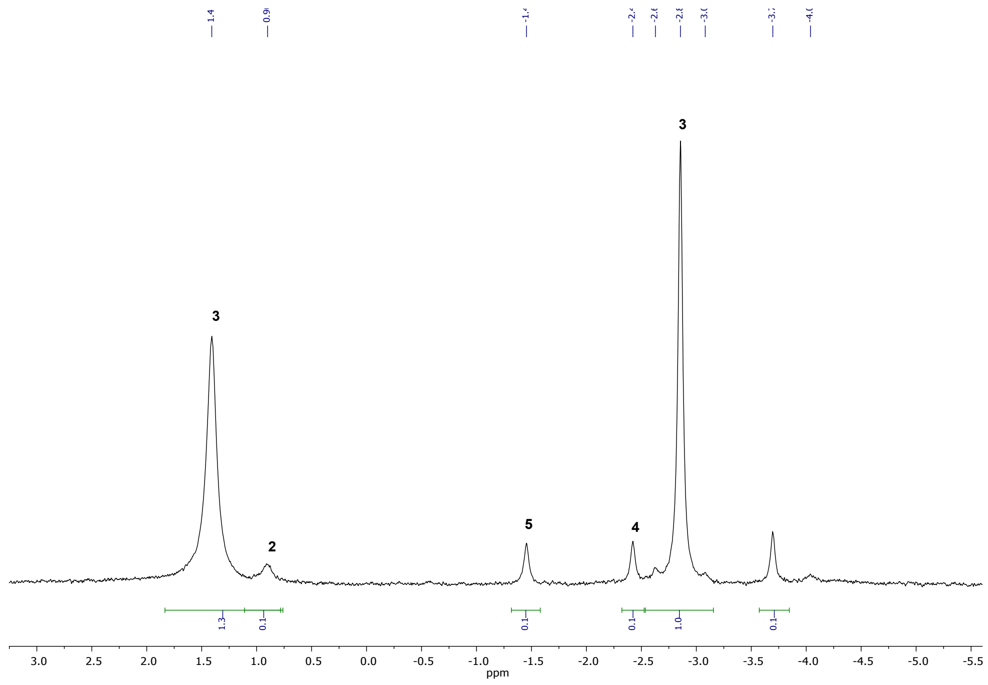

Figure S18c ^{7}Li NMR spectrum (194 MHz, C_6D_6) of an aliquot from the reaction mixture containing TMPCu and TMPLi (1:1) in C_6H_6, which was heated to 50°C for *ca.* 5 days.
